# Hamiltonian formulation of general relativity and post-Newtonian dynamics of compact binaries

**DOI:** 10.1007/s41114-018-0016-5

**Published:** 2018-08-31

**Authors:** Gerhard Schäfer, Piotr Jaranowski

**Affiliations:** 1 Friedrich-Schiller-Universität Jena, Jena, Germany; 2 University of Białystok, Białystok, Poland

**Keywords:** General relativity, Classical spin and gravity, Hamiltonian formalism, Compact binary systems, Canonical equations of motion, Radiation reaction and emission, Analytical and dimensional regularization

## Abstract

Hamiltonian formalisms provide powerful tools for the computation of approximate analytic solutions of the Einstein field equations. The post-Newtonian computations of the explicit analytic dynamics and motion of compact binaries are discussed within the most often applied Arnowitt–Deser–Misner formalism. The obtention of autonomous Hamiltonians is achieved by the transition to Routhians. Order reduction of higher derivative Hamiltonians results in standard Hamiltonians. Tetrad representation of general relativity is introduced for the tackling of compact binaries with spinning components. Configurations are treated where the absolute values of the spin vectors can be considered constant. Compact objects are modeled by use of Dirac delta functions and their derivatives. Consistency is achieved through transition to *d*-dimensional space and application of dimensional regularization. At the fourth post-Newtonian level, tail contributions to the binding energy show up. The conservative spin-dependent dynamics finds explicit presentation in Hamiltonian form through next-to-next-to-leading-order spin–orbit and spin1–spin2 couplings and to leading-order in the cubic and quartic in spin interactions. The radiation reaction dynamics is presented explicitly through the third-and-half post-Newtonian order for spinless objects, and, for spinning bodies, to leading-order in the spin–orbit and spin1–spin2 couplings. The most important historical issues get pointed out.

## Introduction

Before entering the very subject of the article, namely the Hamiltonian treatment of the dynamics of compact binary systems within general relativity (GR) theory, some historical insight will be supplied. The reader may find additional history, e.g., in Damour (1983a, 1987b), Futamase and Itoh (2007), Blanchet (2014), and Porto (2016).

### Early history (1916–1960)

The problem of motion of many-body systems is an important issue in GR (see, e.g., Damour 1983a, 1987b). Earliest computations were performed by Droste, de Sitter, and Lorentz in the years 1916–1917, at the first post-Newtonian (1PN) order of approximation of the Einstein field equations, i.e., at the order $n=1$, where $(1/c^2)^n$ corresponds to the *n*th post-Newtonian (PN) order with $n=0$ being the Newtonian level. Already in the very first paper, where Droste calculated the 1PN gravitational field for a many-body system (Droste 1916), there occurred a flaw in the definition of the rest mass *m* of a self-gravitating body of volume *V* (we follow the Dutch version; the English version contains an additional misprint), reading, in the rest frame of the body, indicated in the following by $\dot{=}$,

1.1 $$\begin{aligned} m\quad {\mathop {=}\limits ^{{\text {Droste 1916}}}} \int _V \mathrm {d}^3 x\,\varrho ~\dot{=} \int _V \mathrm {d}^3 x\,\varrho _*\left( 1-\frac{3U}{c^2}\right) , \end{aligned}$$

where the “Newtonian” mass density $\varrho _*=\sqrt{-g}\varrho u^0/c$ [$g=\det (g_{\mu \nu })$, $u^0$ is the time component of the four-velocity field $u^{\mu }$, $u^{\mu }u_{\mu }=-c^2$] fulfills the metric-free continuity equation

1.2 $$\begin{aligned} \partial _t \varrho _* + \mathrm{div}(\varrho _*\mathbf{v}) = 0, \end{aligned}$$

where $\mathbf{v} = (v^i)$ is the Newtonian velocity field (with $v^i = cu^i/u^0$). The Newtonian potential *U* is defined by

1.3 $$\begin{aligned} \varDelta U = -4\pi G \varrho _*, \end{aligned}$$

with the usual boundary condition for *U* at infinity: $\lim _{|\mathbf {r}|\rightarrow \infty }U(\mathbf {r},t)=0$. Let us stress again that the definition (1.1) is *not* correct. The correct expression for the rest mass contrarily reads, at the 1PN level,

1.4 $$\begin{aligned} m ~\dot{=} \int _V \mathrm {d}^3x\, \varrho _*\left( 1 + \frac{1}{c^2}\left( \varPi -\frac{U}{2}\right) \right) , \end{aligned}$$

with specific internal energy $\varPi$. For pressureless (dust-like) matter, the correct 1PN expression is given by

1.5 $$\begin{aligned} m = \int _V \mathrm {d}^3x\, \varrho _* ~\dot{=} \int _V \mathrm {d}^3x \sqrt{\det (g_{ij})}\,\varrho = \int _V \mathrm {d}V \varrho , \end{aligned}$$

where $$\mathrm {d}V\equiv \sqrt{\det (g_{ij})}\,\mathrm {d}^3x$$.

The error in question slept into second of two sequential papers by de Sitter (1916a, b, 1917) when calculating the 1PN equations of motion for a many-body system. Luckily, that error had no influence on the de Sitter precession of the Moon orbit around the Earth in the gravitational field of the Sun. The error became identified (at least for dusty matter) by Eddington and Clark (1938). On the other side, Levi-Civita (1937b) used the correct rest mass formula for dusty bodies. Einstein criticized the calculations by Levi-Civita because he was missing pressure for stabilizing the bodies. Hereupon, Levi-Civita argued with the “effacing principle”, inaugurated by Brillouin, that the internal structure should have no influence on the external motion. The 1PN gravitational field was obtained correctly by Levi-Civita but errors occurred in the equations of motion including self-acceleration and wrong periastron advance (Levi-Civita 1937a; Damour and Schäfer 1988). Full clarification was achieved by Eddington and Clark (1938), letting aside the unstable interior of their dusty balls. Interestingly, in a 1917 paper by Lorentz and Droste (in Dutch), the correct 1PN Lagrangian of a self-gravitating many-body system of fluid balls was obtained but never properly recognized. Only in 1937, for the edition of the collected works by Lorentz, it became translated into English (Lorentz and Droste 1937). A full-fledged calculation made by Einstein et al. (1938)—posed in the spirit of Hermann Weyl by making use of surface integrals around field singularities—convincingly achieved the 1PN equations of motion, nowadays called Einstein–Infeld–Hoffmann (EIH) equations of motion. Some further refining work by Einstein and Infeld appeared in the 1940s. Fichtenholz (1950) computed the Lagrangian and Hamiltonian out of the EIH equations. A consistent fluid ball derivation of the EIH equations has been achieved by Fock (1939) and Petrova (1949) (delayed by World War II), and Papapetrou (1951a) (see also Fock 1959).

In the 1950s, Infeld and Plebański rederived the EIH equations of motion with the aid of Dirac $\delta$-functions as field sources by postulating the properties of Infeld’s “good” $\delta$-function (Infeld 1954, 1957; Infeld and Plebański 1960; see Sect. 4.2 of our review for more details). Also in the 1950s, the Dirac $\delta$-function became applied to the post-Newtonian problem of motion of spinning bodies by Tulczyjew (1959), based on the seminal work by Mathisson (1937, 2010), with the formulation of a general relativistic gravitational skeleton structure of extended bodies. Equations of motion for spinning test particles had been obtained before by Papapetrou (1951b) and Corinaldesi and Papapetrou (1951). Further in the 1950s, another approach to the equations-of-motion problem, called fast-motion or post-Minkowskian (PM) approximation, which is particularly useful for the treatment of high-speed scattering problems, was developed and elaborated by Bertotti (1956) and Kerr (1959a, b, c), at the 1PM level. First results at the 2PM level were obtained by Bertotti and Plebański (1960).

### History on Hamiltonian results

Hamiltonian frameworks are powerful tools in theoretical physics because of their capacity of full-fledged structural exploration and efficient application of mathematical theories (see, e.g., Holm 1985; Alexander 1987; Vinti 1998). Most importantly, Hamiltonians generate the time evolution of all quantities in a physical theory. For closed systems, the total Hamiltonian is conserved in time. Together with the other conserved quantities, total linear momentum and total angular momentum, which are given by very simple universal expressions, and the boost vector, which is connected with the Hamiltonian density and the total linear momentum, the total Hamiltonian is one of the generators of the globally operating Poincaré or inhomogeneous Lorentz group. A natural ingredient of a Hamiltonian formalism is the ($3+1$)-splitting of spacetime in space and time. Consequently Hamiltonian formalisms allow transparent treatments of both initial value problems and Newtonian limits. Finally, for solving equations of motion, particularly in approximation schemes, Hamiltonian frameworks naturally fit into the powerful Lie-transform technique based on action-angle variables (Hori 1966; Kinoshita 1978; Vinti 1998; Tessmer et al. 2013).

Additionally we refer to an important offspring of the Hamiltonian framework, the effective-one-body (EOB) approach, which will find its presentation in an upcoming *Living Reviews* article by Thibault Damour. References in the present article referring to EOB are particularly Buonanno and Damour (1999, 2000), Damour et al. (2000a, 2008b, 2015), Damour (2001, 2016).

The focus of the present article is on the Hamiltonian formalism of GR as developed by Arnowitt, Deser, and Misner (ADM) (Arnowitt et al. 1959, 1960a, b), with its Routhian modification (Jaranowski and Schäfer 1998, 2000c) (where the matter is treated in Hamiltonian form and the field in the Lagrangian one) and classical-spin generalization (Steinhoff and Schäfer 2009a; Steinhoff 2011), and with application to the problem of motion of binary systems with compact components including proper rotation (spin) and rotational deformation (quadratic in the spin variables); for other approaches to the problem of motion in GR, see the reviews by Futamase and Itoh (2007), Blanchet (2014), and Porto (2016). The review article by Arnowitt et al. (1962) gives a thorough account of the ADM formalism (see also Regge and Teitelboim 1974 for the discussion about asymptotics). In this formalism, the final Hamiltonian, nowadays called ADM Hamiltonian, is given in form of a volume integral of the divergence of a vector over three-dimensional spacelike hypersurface, which can also naturally be represented as surface integral at flat spatial infinity $i^0$.

It is also interesting to give insight into other Hamiltonian formulations of GR, because those are closely related to the ADM approach but differently posed. Slightly ahead of ADM, Dirac (1958, 1959) had developed a Hamiltonian formalism for GR, and slightly afterwards, Schwinger (1963a, b). Schwinger’s approach starts from tetrad representation of GR and ends up with a different set of canonical variables and, related herewith, different coordinate conditions. Dirac has developed his approach with some loose ends toward the final Hamiltonian (see Sect. 2.1 below and also, e.g., Deser 2004), but the coordinate conditions introduced by him—nowadays called Dirac gauge—are often used, mainly in numerical relativity. A subtle problem in all Hamiltonian formulations of GR is the correct treatment of surface terms at spacelike infinity which appear in the asymptotically flat spacetimes. In 1967, this problem has been clearly addressed by De Witt (1967) and later, in 1974, full clarification has been achieved by Regge and Teitelboim (1974). For a short comparison of the three canonical formalisms in question, the Dirac, ADM, and Schwinger ones, see Schäfer (2014).

The first authors who had given the Hamiltonian as two-dimensional surface integral at $i^0$ on three-dimensional spacelike hypersurfaces were ADM. Of course, the representation of the total energy as surface integral was known before, particularly through the Landau–Lifshitz gravitational stress-energy-pseudotensor approach. Schwinger followed the spirit of ADM. He was fully aware of the correctness of his specific calculations modulo surface terms only which finally became fixed by asymptotic Lorentz invariance considerations. He presented the Hamiltonian (as well as the other generators of the Lorentz group) as two-dimensional surface integrals. Only one application of the Schwinger approach by somebody else than Schwinger himself is known to the authors. It is the paper by Kibble in 1963 in which the Dirac spin-1/2 field found a canonical treatment within GR (Kibble 1963). This paper played a crucial role in the implementation of classical spin into the ADM framework by Steinhoff and Schäfer (2009a) and Steinhoff (2011) (details can be found in Sect. 7 of the present article).

The ADM formalism is the most often used Hamiltonian framework in the analytical treatment of the problem of motion of gravitating compact objects. The main reason for this is surely the very well adapted coordinate conditions for explicit calculations introduced by Arnowitt et al. (1960c) (generalized isotropic coordinates; nowadays, for short, often called ADMTT coordinates, albeit the other coordinates introduced by Arnowitt et al. 1962, are ADMTT too), though also in Schwinger’s approach similar efficient coordinate conditions could have been introduced (Schäfer 2014). Already Kimura (1961) started application of the ADM formalism to gravitating point masses at the 1PN level. In 1974, that research activity culminated in a 2PN Hamiltonian for binary point masses obtained by Hiida and Okamura (1972), Ohta et al. (1974a, b). However, one coefficient of their Hamiltonian was not correctly calculated and the Hamiltonian as such was not clearly identified, i.e., it was not clear to which coordinate system it referred to. In 1985, full clarification has been achieved in a paper by Damour and Schäfer (1985) relying on the observation by Schäfer (1984) that the perturbative use of the equations of motion on the action level implies that coordinate transformations have been applied; also see Barker and O’Connell (1984, 1986). In addition, Damour and Schäfer (1985) showed how to correctly compute the delicate integral ($U^\text {TT}$) which had been incorrectly evaluated by Hiida and Okamura (1972), Ohta et al. (1974a, b), and made contact with the first fully correct calculation of the 2PN dynamics of binary systems (in harmonic coordinates) by Damour and Deruelle (1981) and Damour (1982) in 1981–1982.

In Schäfer (1983b), the leading-order 2.5PN radiation reaction force for *n*-body systems was derived by using the ADM formalism. The same force expression had already been obtained earlier by Schäfer (1982) within coordinate conditions closely related to the ADM ones—actually identical with the ADM conditions through 1PN and at 2.5PN order—and then again by Schäfer (1983a), as quoted in Poisson and Will (2014), based on a different approach but in coordinates identical to the ADM ones at 2.5PN order. The 2PN Hamiltonian shown by Schäfer (1982) and taken from Ohta et al. (1974b), apart from the erroneous coefficient mentioned above, is the ADM one as discussed above (the factor 7 in the static part therein has to be replaced by 5), and in the definition of the reaction force in the centre-of-mass system, a misprinted factor 2 is missing, i.e. $2\mathbf{F}=\mathbf{F}_1-\mathbf{F}_2$. The detailed calculations were presented in Schäfer (1985, 1986), a further ADM-based derivation by use of a PM approximation scheme has been performed. At 2PN level, the genuine 3-body potential was derived by Schäfer (1987). However, in the reduction of a 4-body potential derived by Ohta et al. (1973, 1974a, b) to three bodies made by Schäfer (1987) some combinatorical shortcomings slept in, which were identified and corrected by Lousto and Nakano (2008), and later by Galaviz and Brügmann (2011) in different form. The *n*-body 3.5PN non-autonomous radiation reaction Hamiltonian^1^ was obtained by the authors in Jaranowski and Schäfer (1997), confirming energy balance results in Blanchet and Schäfer (1989), and the equations of motion out of it were derived by Königsdörffer et al. (2003).

Additionally within the ADM formalism, for the first time in 2001, the conservative 3PN dynamics for compact binaries has been fully obtained by Damour and the authors, by also for the first time making extensive use of the dimensional regularization technique (Damour et al. 2001) (for an earlier mentioning of application of dimensional regularization to classical point particles, see Damour 1980, 1983a; and for an earlier *n*-body static result, i.e. a result valid for vanishing particle momenta and vanishing reduced canonical variables of the gravitational field, not based on dimensional regularization, see Kimura and Toiya 1972). Only by performing all calculations in a *d*-dimensional space the regularization has worked out fully consistently in the limit $d\rightarrow 3$ (later on, a *d*-dimensional Riesz kernel calculation has been performed too, Damour et al. 2008a). In purely 3-dimensional space computations two coefficients, denoted by $\omega _{\mathrm{kinetic}}$ and $\omega _{\mathrm{static}}$, could not be determined by analytical three-dimensional regularization. The coefficient $\omega _{\mathrm{kinetic}}$ was shown to be fixable by insisting on global Lorentz invariance and became thus calculable with the aid of the Poincaré algebra (with value 24/41) (Damour et al. 2000c, d). The first evaluation of the value of $\omega _{\mathrm{static}}$ (namely $\omega _{\mathrm{static}}=0$) was obtained by Jaranowski and Schäfer (1999, 2000b) by assuming a matching with the Brill–Lindquist initial-value configuration of two black holes. The correctness of this value (and thereby the usefulness of considering that the Brill–Lindquist initial-value data represent a relevant configuration of two black holes) was later confirmed by dimensional regularization (Damour et al. 2001). Explicit analytical solutions for the motion of compact binaries through 2PN order were derived by Damour and Schäfer (1988) and Schäfer and Wex (1993b, c), and through 3PN order by Memmesheimer et al. (2005), extending the seminal 1PN post-Keplerian parametrization proposed by Damour and Deruelle (1985).

Quite recently, the 4PN binary dynamics has been successfully derived, using dimensional regularization and sophisticated far-zone matching (Jaranowski and Schäfer 2012, 2013, 2015; Damour et al. 2014). Let us remark in this respect that the linear in *G* (Newtonian gravitational constant) part can be deduced to all PN orders from the 1PM Hamiltonian derived by Ledvinka et al. (2008). For the first time, the contributions to 4PN Hamiltonian were obtained by the authors in Jaranowski and Schäfer (2012) through $G^2$ order, including additionally all log-terms at 4PN going up to the order $G^5$. Also the related energy along circular orbits was obtained as function of orbital frequency. The application of the Poincaré algebra by Jaranowski and Schäfer (2012) clearly needed the noncentre-of-mass Hamiltonian, though only the centre-of-mass one was published. By Jaranowski and Schäfer (2013), all terms became calculated with the exception of terms in the reduced Hamiltonian linear in $\nu \equiv m_1m_2/(m_1+m_2)^2$ (where $m_1$ and $m_2$ denote the masses of binary system components) and of the orders $G^3$, $G^4$, and $G^5$. Those terms are just adding up to the log-terms mentioned above. However, taking a numerical self-force solution for circular orbits in the Schwarzschild metric into account, already the innermost (or last) stable circular orbit could be determined numerically through 4PN order by Jaranowski and Schäfer (2013). The complete 4PN analytic conservative Hamiltonian has been given for the first time by Damour et al. (2014), based on Jaranowski and Schäfer (2015), together with the results of Le Tiec et al. (2012) and Bini and Damour (2013). Applications of it for bound and unbound orbits were performed by Damour et al. (2015) and Bini and Damour (2017).

For spinning bodies, counting spin as 0.5PN effect, the 1.5PN spin–orbit and 2PN spin–spin Hamiltonians were derived by Barker and O’Connell (1975, 1979), where the given quadrupole-moment-dependent part can be regarded as representing spin-squared terms for extended bodies (notice the presence of the tensor product of two unit vectors pointing each to the spin direction in the quadrupole-moment-dependent Hamiltonians). For an observationally important application of the spin–orbit dynamics, see Damour and Schäfer (1988). In 2008, the 2.5PN spin–orbit Hamiltonian was successfully calculated by Damour et al. (2008c), and the 3PN spin1–spin2 and spin1–spin1 binary black-hole Hamiltonians by Steinhoff et al. (2008a, b, c). The 3PN spin1–spin1 Hamiltonian for binary neutron stars was obtained by Hergt et al. (2010). The 3.5PN spin–orbit and 4PN spin1–spin2 Hamiltonians were obtained by Hartung and Steinhoff (2011a, b) (also see Hartung et al. 2013; Levi and Steinhoff 2014). The 4PN spin1–spin1 Hamiltonian was presented in Levi and Steinhoff (2016a). Based on the Dirac approach, the Hamiltonian of a spinning test-particle in the Kerr metric has been obtained by Barausse et al. (2009, 2012). The canonical Hamiltonian for an extended test body in curved spacetime, to quadratic order in spin, was derived by Vines et al. (2016). Finally, the radiation-reaction Hamiltonians from the leading-order spin–orbit and spin1–spin2 couplings have been derived by Steinhoff and Wang (2010) and Wang et al. (2011).

### More recent history on non-Hamiltonian results

At the 2PN level of the equations of motion, the Polish school founded by Infeld succeeded in getting many expressions whereby the most advanced result was obtained by Ryteń in her MSc thesis from 1961 using as model for the source of the gravitational field Infeld’s “good $\delta$-function”. Using the same source model as applied by Fock and Petrova, Kopeikin (1985) and Grishchuk and Kopeikin (1986) derived the 2PN and 2.5PN equations of motion for compact binaries. However, already in 1982, Damour and Deruelle had obtained the 2PN and 2.5PN equations of motion for compact binaries, using analytic regularization techniques [Damour 1982, 1983a, b (for another such derivation see Blanchet et al. 1998)]. Also Ohta and Kimura (1988) should be mentioned for a Fokker action derivation of the 2PN dynamics. Regarding the coordinate conditions used in the papers quoted in the present subsection, treating spinless particles, all are based on the harmonic gauge with the exceptions of the ones with a Hamiltonian background and those by Ryteń or Ohta and Kimura.

Using the technique of Einstein, Infeld, and Hoffmann (EIH), Itoh and Futamase (2003) and Itoh (2004) succeeded in deriving the 3PN equations of motion for compact binaries, and Blanchet et al. (2004) derived the same 3PN equations of motion based on dimensional regularization. The extended Hadamard regularization, developed and applied before (Blanchet and Faye 2000a, b, 2001a, b), is incompatible^2^ with distribution theory and with the method of dimensional regularization (Jaranowski and Schäfer 2015). The 3.5PN equations of motion were derived within several independent approaches: by Pati and Will (2002) using the method of direct integration of the relaxed Einstein equations (DIRE) developed by Pati and Will (2000), by Nissanke and Blanchet (2005) applying Hadamard self-field regularization, by Itoh (2009) using the EIH technique, and by Galley and Leibovich (2012) within the effective field theory (EFT) approach. Radiation recoil effects, starting at 3.5PN order, have been discussed by Bekenstein (1973), Fitchett (1983), Junker and Schäfer (1992), Kidder (1995), and Blanchet et al. (2005).

Bernard et al. (2016) calculated the 4PN Fokker action for binary point-mass systems and found a nonlocal-in-time Lagrangian inequivalent to the Hamiltonian obtained by Damour et al. (2014). On the one hand, the local part of the result of Bernard et al. (2016) differed from the local part of the Hamiltonian of Damour et al. (2014) only in a few terms. On the other hand, though the nonlocal-in-time part of the action in Bernard et al. (2016) was the same as the one in Damour et al. (2014, 2015), Bernard et al. (2016) advocated to treat it (notably for deriving the conserved energy, and deriving its link with the orbital frequency) in a way which was inequivalent to the one in Damour et al. (2014, 2015). It was then shown by Damour et al. (2016) that: (i) the treatment of the nonlocal-in-time part in Bernard et al. (2016) was not correct, and that (ii) the difference in local-in-time terms was composed of a combination of gauge terms and of a new ambiguity structure which could be fixed either by matching to Damour et al. (2014, 2015) or by using the results of self-force calculations in the Schwarzschild metric. In their recent articles (Bernard et al. 2017a, b) Blanchet and collaborators have recognized that the criticisms of Damour et al. (2016) were founded, and, after correcting their previous claims and using results on periastron precession first derived by Damour et al. (2015, 2016), have obtained full equivalence with the earlier derived ADM results. Let us also mention that Marchand et al. (2018) has presented a self-contained calculation of the full 4PN dynamics (not making any use of self-force results), which confirms again the correctness of the 4PN dynamics first obtained by Damour et al. (2014). The computation of Marchand et al. (2018) can be viewed as a 4PN analog of the 3PN derivation presented in Damour et al. (2001), in which the power of dimensional regularization in post-Newtonian calculations has been established for the first time. An application of the 4PN dynamics for bound orbits was performed by Bernard et al. (2017b).

The application of EFT approach to PN calculations, devised by Goldberger and Rothstein (2006a, b), has also resulted in PN equations of motion for spinless particles up to the 3PN order (Gilmore and Ross 2008; Kol and Smolkin 2009; Foffa and Sturani 2011). At the 4PN level, Foffa and Sturani (2013a) calculated a quadratic in *G* higher-order Lagrangian, the published version of which was found in agreement with Jaranowski and Schäfer (2012). The quintic in *G* part of the 4PN Lagrangian was derived within the EFT approach by Foffa et al. (2017) (with its 2016 arXiv version corrected by Damour and Jaranowski 2017). Galley et al. (2016) got the 4PN nonlocal-in-time tail part. Recently, Porto and Rothstein (2017) and Porto (2017) performed a deeper analysis of IR divergences in PN expansions.

The 1.5PN spin–orbit dynamics was derived in Lagrangian form by Tulczyjew (1959) and Damour (1982). The 2PN spin–spin equations of motion were derived by D’Eath (1975a, b), and Thorne and Hartle (1985), respectively, for rotating black holes. The 2.5PN spin–orbit dynamics was successfully tackled by Tagoshi et al. (2001), and by Faye et al. (2006), using harmonic coordinates approach. Within the EFT approach, Porto (2010) and Levi (2010a) succeeded in determining the same coupling (also see Perrodin 2011). The 3PN spin1–spin2 dynamics was successfully tackled by Porto and Rothstein (2008b, 2010b) (based on Porto 2006; Porto and Rothstein 2006) and by Levi (2010b), and the 3PN spin1–spin1 one, again by Porto and Rothstein (2008a), but given in 2010 only in fully correct form (Porto and Rothstein 2010a). For the 3PN spin1–spin1 dynamics, also see Bohé et al. (2015). The most advanced results for spinning binaries can be found in Levi (2012), Marsat et al. (2013), Bohé et al. (2013), Marsat (2015), and Levi and Steinhoff (2016a, b, c), reaching 3.5PN and 4PN levels (also see Steinhoff 2017). Finally, the radiation-reaction dynamics of the leading-order spin–orbit and spin1–spin2 couplings have been obtained by Wang and Will (2007) and Zeng and Will (2007), based on the DIRE method (Will 2005) (see also Maia et al. 2017a, b, where the EFT method became applied).

### Notation and conventions

In this article, Latin indices from the mid alphabet are running from 1 to 3 (or *d* for an arbitrary number of space dimensions), Greek indices are running from 0 to 3 (or *d* for arbitrary space dimensions), whereby $x^0 = ct$. We denote by $\mathbf {x}=(x^i)$ ($i\in \{1,\ldots ,d\}$) a point in the *d*-dimensional Euclidean space ${\mathbb {R}}^d$ endowed with a standard Euclidean metric defining a scalar product (denoted by a dot). For any spatial *d*-dimensional vector $\mathbf {w}=(w^i)$ we define $|\mathbf{w}|\equiv \sqrt{\mathbf{w}\cdot \mathbf{w}}\equiv \sqrt{\delta _{ij}w^iw^j}$, so $|\cdot |$ stands here for the Euclidean length of a vector, $\delta _{ij}=\delta ^i_j$ denotes Kronecker delta. The partial differentiation with respect to $x^\mu$ is denoted by $\partial _\mu$ or by a comma, i.e., $\partial _\mu \phi \equiv \phi _{,\mu }$, and the partial derivative with respect to time coordinates *t* is denoted by $\partial _t$ or by an overdot, $\partial _t\phi \equiv {\dot{\phi }}$. The covariant differentiation is generally denoted by $\nabla$, but we may also write $\nabla _\alpha (\cdot )\equiv (\cdot )_{||\alpha }$ for spacetime or $\nabla _i(\cdot )\equiv (\cdot )_{;i}$ for space variables, respectively. The signature of the $(d+1)$-dimensional metric $g_{\mu \nu }$ is $+(d-1)$. The Einstein summation convention is adopted. The speed of light is denoted by *c* and *G* is the Newtonian gravitational constant.

We use the notion of a *tensor density*. The components of a tensor density of weight *w*, *k* times contravariant and *l* times covariant, transform, when one changes one coordinate system to another, by the law [see, e.g., p. 501 in Misner et al. (1973) or, for more general case, Sects. 3.7–3.9 and 4.5 in Plebański and Krasiński (2006), where however definition of the density weight differs by sign from the convention used by us]

1.6 $$\begin{aligned} {\mathcal {T}}^{\alpha '_1\ldots \alpha '_k}_{\beta '_1\ldots \beta '_l} = \left( \frac{\partial x'}{\partial x}\right) ^{-w} {x^{\alpha '_1}}_{,\alpha _1}\ldots {x^{\alpha '_k}}_{,\alpha _k} {x^{\beta _1}}_{,\beta '_1}\ldots {x^{\beta _l}}_{,\beta '_l} {\mathcal {T}}^{\alpha _1\ldots \alpha _k}_{\beta _1\ldots \beta _l}, \end{aligned}$$

where $({\partial x'}/{\partial x})$ is the Jacobian of the transformation $x\rightarrow x'(x)$. E.g., determinant of the metric $g\equiv \det (g_{\mu \nu })$ is a scalar density of weight $+2$. The covariant derivative of the tensor density of weight *w*, *k* times contravariant and *l* times covariant, is computed according to the rule

1.7 $$\begin{aligned} \nabla _\gamma {\mathcal {T}}^{\alpha _1\ldots \alpha _k}_{\beta _1\ldots \beta _l}&= \partial _\gamma {\mathcal {T}}^{\alpha _1\ldots \alpha _k}_{\beta _1\ldots \beta _l} - w \varGamma ^\rho _{\rho \gamma } {\mathcal {T}}^{\alpha _1\ldots \alpha _k}_{\beta _1\ldots \beta _l} \nonumber \\&\quad + \sum _{i=1}^k \varGamma ^{\alpha _i}_{\rho _i\gamma } {\mathcal {T}}^{\alpha _1\ldots \rho _i\ldots \alpha _k}_{\beta _1\ldots \beta _l} - \sum _{j=1}^l \varGamma ^{\rho _j}_{\beta _j\gamma } {\mathcal {T}}^{\alpha _1\ldots \alpha _k}_{\beta _1\ldots \rho _j\ldots \beta _l}. \end{aligned}$$

For the often used case when ${\mathcal {T}}^{\alpha _1\ldots \alpha _k}_{\beta _1\ldots \beta _l}=|g|^{w/2}T^{\alpha _1\ldots \alpha _k}_{\beta _1\ldots \beta _l}$ (where $T^{\alpha _1\ldots \alpha _k}_{\beta _1\ldots \beta _l}$ is a tensor *k* times contravariant and *l* times covariant), Eq. (1.7) implies that the covariant derivative of ${\mathcal {T}}^{\alpha _1\ldots \alpha _k}_{\beta _1\ldots \beta _l}$ can be computed by means of the rule,

1.8 $$\begin{aligned} \nabla _{\gamma }{\mathcal {T}}^{\alpha _1\ldots \alpha _k}_{\beta _1\ldots \beta _l} = T^{\alpha _1\ldots \alpha _k}_{\beta _1\ldots \beta _l} \nabla _{\gamma }|g|^{w/2} + |g|^{w/2} \nabla _{\gamma } T^{\alpha _1\ldots \alpha _k}_{\beta _1\ldots \beta _l} = |g|^{w/2} \nabla _{\gamma } T^{\alpha _1\ldots \alpha _k}_{\beta _1\ldots \beta _l}, \end{aligned}$$

because

1.9 $$\begin{aligned} \nabla _{\gamma }|g|^{w/2} = \partial _{\gamma }|g|^{w/2} - w \varGamma ^{\rho }_{\rho \gamma }|g|^{w/2} = 0. \end{aligned}$$

Letters *a*, *b* ($a,b=1,2$) are particle labels, so ${\mathbf {x}}_a=(x_a^i)\in {\mathbb {R}}^d$ denotes the position of the *a*th point mass. We also define $\mathbf{r}_a\equiv \mathbf {x}-{\mathbf {x}}_a$, $r_a\equiv |\mathbf{r}_a|$, ${\mathbf {n}}_a\equiv \mathbf{r}_a/r_a$; and for $a\ne b$, $\mathbf{r}_{ab}\equiv {\mathbf {x}}_a-\mathbf {x}_b$, $r_{ab}\equiv |\mathbf{r}_{ab}|$, ${\mathbf {n}}_{ab}\equiv \mathbf{r}_{ab}/r_{ab}$. The linear momentum vector of the *a*th particle is denoted by ${\mathbf {p}}_a=(p_{ai})$, and $m_a$ denotes its mass parameter. We abbreviate Dirac delta distribution $\delta (\mathbf {x}-{\mathbf {x}}_a)$ by $\delta _a$ (both in *d* and in 3 dimensions); it fulfills the condition $\int \mathrm {d}^dx\,\delta _a=1$.

Thinking in terms of dimensions of space, *d* has to be an integer, but whenever integrals within dimensional regularization get performed, we allow *d* to become an arbitrary complex number [like in the analytic continuation of factorial $n!=\varGamma (n+1)$ to $\varGamma (z)$].

## Hamiltonian formalisms of GR

The presented Hamiltonian formalisms do all rely on a ($3+1$) splitting of spacetime metric $g_{\mu \nu }$ in the following form:

2.1 $$\begin{aligned} \mathrm {d}s^2 = g_{\mu \nu }\mathrm {d}x^{\mu }\mathrm {d}x^{\nu } = -(Nc\,\mathrm {d}t)^2 + \gamma _{ij}(\mathrm {d}x^i + N^ic\,\mathrm {d}t)(\mathrm {d}x^j + N^jc\,\mathrm {d}t), \end{aligned}$$

where

2.2 $$\begin{aligned} \gamma _{ij} \equiv g_{ij}, \quad N \equiv (-g^{00})^{-1/2}, \quad N^i = \gamma ^{ij}N_j \quad \text {with}\quad N_i \equiv g_{0i}, \end{aligned}$$

here $\gamma ^{ij}$ is the inverse metric of $\gamma _{ij}$ ($\gamma _{ik}\gamma ^{kj}=\delta _i^j$), $\gamma \equiv \mathrm{det}(\gamma _{ij})$; lowering and raising of spatial indices is with $\gamma _{ij}$. The splitting (2.1), and the associated explicit $3+1$ decomposition of Einstein’s equations, was first introduced by Fourès-Bruhat (1956). The notations *N* and $N^i$ are due to Arnowitt et al. (1962) and their names, respectively “lapse” and “shift” functions, are due to Wheeler (1964). Let us note the useful relation between the determinants $g\equiv \det (g_{\mu \nu })$ and $\gamma$:

2.3 $$\begin{aligned} g = -N^2 \gamma . \end{aligned}$$

We restrict ourselves to consider only *asymptotically flat spacetimes* and we employ *quasi-Cartesian coordinate systems* $(t,x^i)$ which are characterized by the following asymptotic spacelike behaviour (i.e., in the limit $r\rightarrow \infty$ with $r\equiv \sqrt{x^ix^i}$ and *t* = const) of the metric coefficients:

2.4 $$\begin{aligned} N&= 1+O(1/r), \quad N^i=O(1/r), \quad \gamma _{ij}=\delta _{ij}+O(1/r), \end{aligned}$$

2.5 $$\begin{aligned} N_{,i}&= O(1/r^2), \quad N^i_{,j}=O(1/r^2), \quad \gamma _{ij,k}=O(1/r^2). \end{aligned}$$

De Witt (1967) and later, in a more refined way, Regge and Teitelboim (1974) explicitly showed that the Hamiltonian which generates all Einsteinian field equations can be put into the form,

2.6 $$\begin{aligned} H[\gamma _{ij},\pi ^{ij},N,N^i;q^A,\pi _A]&= \int \mathrm {d}^3x\, (N{{\mathcal {H}}} - c N^i {\mathcal {H}}_i) \nonumber \\&\quad + \frac{c^4}{16\pi G}\oint _{i^0} \mathrm {d}S_i\,\partial _j (\gamma _{ij} - \delta _{ij} \gamma _{kk}), \end{aligned}$$

wherein *N* and $N^i$ operate as Lagrangian multipliers and where $\mathcal {H}$ and ${\mathcal {H}}_i$ are Hamiltonian and momentum densities, respectively; $i^0$ denotes spacelike flat infinity. They depend on matter canonical variables $q^A,\pi _A$ (through matter Hamiltonian density ${\mathcal {H}}_{\text {m}}$ and matter momentum density ${\mathcal {H}}_{\text {m}i}$) and read

2.7 $$\begin{aligned} {\mathcal {H}}\equiv & {} \frac{c^4}{16\pi G}\left[ -\gamma ^{1/2} R + \frac{1}{\gamma ^{1/2}}\left( \gamma _{ik}\gamma _{jl}\pi ^{ij}\pi ^{kl}-\frac{1}{2}\pi ^2\right) \right] + {\mathcal {H}}_{\text {m}}, \end{aligned}$$

2.8 $$\begin{aligned} {\mathcal {H}}_i\equiv & {} \frac{c^3}{8\pi G}\gamma _{ij}\nabla _k\pi ^{jk} + {\mathcal {H}}_{\text {m}i}, \end{aligned}$$

where *R* is the intrinsic curvature scalar of the spacelike hypersurfaces of constant-in-time slices $t=x^0/c$ = const; the ADM canonical field momentum is given by the density $\displaystyle \frac{c^3}{16\pi G}\pi ^{ij}$, where

2.9 $$\begin{aligned} \pi _{ij} \equiv -\gamma ^{1/2} (K_{ij}-K\gamma _{ij}), \end{aligned}$$

with $K\equiv \gamma ^{ij}K_{ij}$, where $K_{ij}=-N \varGamma ^0_{ij}$ is the extrinsic curvature of *t* = const slices, $\varGamma ^0_{ij}$ denote Christoffel symbols; $\pi \equiv \gamma _{ij}\pi ^{ij}$; $\nabla _k$ denotes the three-dimensional covariant derivative (with respect to $\gamma _{ij}$). The given densities are densities of weight one with respect to three-dimensional coordinate transformations. Let us note the useful formula for the density of the three-dimensional scalar curvature of the surface *t* = const:

2.10 $$\begin{aligned} \sqrt{\gamma } R&= \frac{1}{4} \sqrt{\gamma } \Big (\big (\gamma ^{ij}\gamma ^{lm}-\gamma ^{il}\gamma ^{jm}\big )\gamma ^{kn} + 2\big (\gamma ^{il}\gamma ^{km}-\gamma ^{ik}\gamma ^{lm}\big )\gamma ^{jn}\Big )\gamma _{ij,k}\gamma _{lm,n} \nonumber \\&\quad + \partial _i\big (\gamma ^{-1/2}\partial _j (\gamma \gamma ^{ij})\big ). \end{aligned}$$

The matter densities ${\mathcal {H}}_{\text {m}}$ and ${\mathcal {H}}_{\text {m}i}$ are computed from components of the matter energy-momentum tensor $T^{\mu \nu }$ by means of formulae

2.11 $$\begin{aligned} {\mathcal {H}}_{\text {m}}&= \sqrt{\gamma }\,T^{\mu \nu }n_\mu n_\nu = \sqrt{\gamma }\, N^2 T^{00}, \end{aligned}$$

2.12 $$\begin{aligned} {\mathcal {H}}_{\text {m}i}&= -\sqrt{\gamma }\,T^{\mu }_i n_\mu = \sqrt{\gamma }\, N T^0_i, \end{aligned}$$

where $n_\mu =(-N,0,0,0)$ is the timelike unit covector orthogonal to the spacelike hypersurfaces *t* = const. Opposite to what the right-hand sides of Eqs. (2.11)–(2.12) seem to suggest, the matter densities must be independent on lapse *N* and shift $N^i$ and expressible in terms of the dynamical matter and field variables $q^A$, $\pi _A$, $\gamma _{ij}$ only ($\pi ^{ij}$ does not show up for matter which is minimally coupled to the gravitational field). The variation of (2.6) with respect to *N* and $N^i$ yields the *constraint equations*

2.13 $$\begin{aligned} {\mathcal {H}} = 0 \quad \text {and} \quad {\mathcal {H}}_i = 0. \end{aligned}$$

The most often applied Hamiltonian formalism employs the following coordinate choice made by ADM (which we call ADMTT gauge),

2.14 $$\begin{aligned} \pi ^{ii} = 0, \qquad 3\partial _j\gamma _{ij} - \partial _i\gamma _{jj} = 0 \quad \text {or} \quad \gamma _{ij} = \psi \delta _{ij} + h_{ij}^{\mathrm{TT}}, \end{aligned}$$

where the TT piece $h_{ij}^{\mathrm{TT}}$ is transverse and traceless, i.e., it satisfies $\partial _jh_{ij}^{\mathrm{TT}}=0$ and $h_{ii}^{\mathrm{TT}}=0$. The TT piece of any field function can be computed by means of the TT projection operator defined as follows

2.15 $$\begin{aligned} \delta ^{\mathrm{TT}kl}_{ij} \equiv \frac{1}{2}(P_{il}P_{jk} + P_{ik}P_{jl} - P_{kl}P_{ij}), \quad P_{ij} \equiv \delta _{ij} - \partial _i\partial _j\varDelta ^{-1}, \end{aligned}$$

where $\varDelta ^{-1}$ denotes the inverse of the flat space Laplacian, which is taken without homogeneous solutions for source terms decaying fast enough at infinity (in 3-dimensional or, if not, then in generalized *d*-dimensional space). The nonlocality of the TT-operator $\delta ^{\mathrm{TT}kl}_{ij}$ is just the gravitational analogue of the well-known nonlocality of the Coulomb gauge in the electrodynamics.

Taking into account its gauge condition as given in Eq. (2.14), the field momentum $\displaystyle \frac{c^3}{16\pi G}\pi ^{ij}$ can be split into its longitudinal and TT parts, respectively,

2.16 $$\begin{aligned} \pi ^{ij} = {\tilde{\pi }}^{ij} + \pi ^{ij}_{\mathrm{TT}}, \end{aligned}$$

where the TT part $\pi ^{ij}_{\mathrm{TT}}$ fulfills the conditions $\partial _j\pi ^{ij}_{\mathrm{TT}}=0$ and $\pi ^{ii}_{\mathrm{TT}}=0$ and where the longitudinal part ${\tilde{\pi }}^{ij}$ can be expressed in terms of a vectorial function $V^i$,

2.17 $$\begin{aligned} {\tilde{\pi }}^{ij} = \partial _i V^j +\partial _j V^i - \frac{2}{3}\delta ^{ij}\partial _k V^k. \end{aligned}$$

It is also convenient to parametrize the field function $\psi$ from Eq. (2.14) in the following way

2.18 $$\begin{aligned} \psi = \left( 1 + \frac{1}{8}\phi \right) ^4. \end{aligned}$$

The independent field variables are $\pi ^{ij}_{\mathrm{TT}}$ and $h_{ij}^{\mathrm{TT}}$. Already Kimura (1961) used just this presentation for applications. The Poisson bracket for the independent degrees of freedom reads

2.19 $$\begin{aligned}&\{F(\mathbf {x}),G(\mathbf {y})\} \equiv \frac{16\pi G}{c^3} \nonumber \\&\quad \times \int \mathrm {d}^3z\, \left( \frac{\delta F(\mathbf {x})}{\delta h^{\mathrm{TT}}_{ij}(\mathbf {z})}\bigg (\delta ^{\mathrm{TT}kl}_{ij}(\mathbf {z})\frac{\delta G(\mathbf {y})}{\delta \pi ^{kl}_{\mathrm{TT}}(\mathbf {z})}\bigg ) - \frac{\delta G(\mathbf {y})}{\delta h^{\mathrm{TT}}_{ij}(\mathbf {z})}\bigg (\delta ^{\mathrm{TT}kl}_{ij}(\mathbf {z})\frac{\delta F(\mathbf {x})}{\delta \pi ^{kl}_{\mathrm{TT}}(\mathbf {z})}\bigg )\right) , \end{aligned}$$

where $\delta F(\mathbf {x})/(\delta f(\mathbf {z}))$ denotes the functional (or Frèchet) derivative. ADM gave the Hamiltonian in fully reduced form, i.e., after having applied (four) constraint equations (2.13) and (four) coordinate conditions (2.14). It reads

2.20 $$\begin{aligned} H_\text {red}[h_{ij}^{\mathrm{TT}},\pi ^{ij}_{\mathrm{TT}};q^A,\pi _A]&= \frac{c^4}{16\pi G}\oint _{i^0} \mathrm {d}S_i\, \partial _j (\gamma _{ij} - \delta _{ij} \gamma _{kk}) \nonumber \\&= \frac{c^4}{16\pi G}\int \mathrm {d}^3x\, \partial _i\partial _j (\gamma _{ij} - \delta _{ij} \gamma _{kk}). \end{aligned}$$

The reduced Hamiltonian generates the field equations of the two remaining metric coefficients (eight metric coefficients are determined by the four constraint equations and four coordinate conditions combined with four otherwise degenerate field equations for the lapse and shift functions). By making use of (2.18) the reduced Hamiltonian (2.20) can be written as

2.21 $$\begin{aligned} H_\text {red}[h_{ij}^{\mathrm{TT}},\pi ^{ij}_{\mathrm{TT}};q^A,\pi _A] = -\frac{c^4}{16\pi G} \int \mathrm {d}^3x\, \varDelta \phi [h_{ij}^{\mathrm{TT}},\pi ^{ij}_{\mathrm{TT}};q^A,\pi _A]. \end{aligned}$$

### Hamiltonian formalisms of Dirac and Schwinger

Dirac had chosen the following coordinate system, called “maximal slicing” because of the field momentum condition,

2.22 $$\begin{aligned} \pi \equiv \gamma _{ij}\pi ^{ij} = 0, \quad \partial _j(\gamma ^{1/3}\gamma ^{ij}) = 0. \end{aligned}$$

The reason for calling the condition $\pi =2K\gamma ^{1/2}=0$ “maximal slicing” is because the congruence of the timelike unit vectors $n^{\mu }$ normal to the $t =$ const hypersurfaces (slices)—as such irrotational—is free of expansion (notice that $\nabla _\mu n^{\mu }=-K$). Hereof it immediately follows that a finite volume in any slice gets unchanged by a small timelike deformation of the slice which vanishes on the boundary of the volume, i.e. an extremum principle holds (see, e.g., York 1979). The corresponding independent field variables are (no implementation of the three differential conditions!)

2.23 $$\begin{aligned} {\tilde{\pi }}^{ij} = \Big (\pi ^{ij}-\frac{1}{3}\gamma ^{ij}\pi \Big )\gamma ^{1/3}, \quad \tilde{g}_{ij} = \gamma ^{-1/3}\gamma _{ij}, \end{aligned}$$

with the algebraic properties $\gamma _{ij}{\tilde{\pi }}^{ij}=0$ and $\det (\tilde{g}_{ij})=1$. To leading order linear in the metric functions, the Dirac gauge coincides with the ADM gauge. The reduction of the Dirac form of dynamics to the independent tilded degrees of freedom has been performed by Regge and Teitelboim (1974), including a fully satisfactory derivation of the Hamiltonian introduced by Dirac. The Poisson bracket for the Dirac variables reads

2.24 $$\begin{aligned} \{F,G\}&= \int \mathrm {d}^3z\,{\tilde{\delta }}^{kl}_{ij}(\mathbf {z}) \left( \frac{\delta F}{\delta \tilde{g}_{ij}(\mathbf {z})}\frac{\delta G}{\delta {\tilde{\pi }}^{kl}(\mathbf {z})} - \frac{\delta G}{\delta \tilde{g}_{ij}(\mathbf {z})}\frac{\delta F}{\delta {\tilde{\pi }}^{kl}(\mathbf {z})}\right) \nonumber \\&\quad + \frac{1}{3}\int \mathrm {d}^3z\,\big ({\tilde{\pi }}^{ij}(\mathbf {z})\tilde{g}^{kl}(\mathbf {z}) - {\tilde{\pi }}^{kl}(\mathbf {z})\tilde{g}^{ij}(\mathbf {z})\big ) \frac{\delta F}{\delta {\tilde{\pi }}^{ij}(\mathbf {z})}\frac{\delta G}{\delta {\tilde{\pi }}^{kl}(\mathbf {z})}, \end{aligned}$$

with

2.25 $$\begin{aligned} {\tilde{\delta }}^{kl}_{ij} \equiv \frac{1}{2}(\delta ^k_i\delta ^l_j + \delta ^l_i\delta ^k_j) - \frac{1}{3}\tilde{g}_{ij}\tilde{g}^{kl}, \quad \tilde{g}_{ij}\tilde{g}^{jl} = \delta ^l_i. \end{aligned}$$

The Hamiltonian proposed by Dirac results from the expression

2.26 $$\begin{aligned} H_{\mathrm{D}} = -\frac{c^4}{16\pi G} \int \mathrm {d}^3x ~\partial _i(\gamma ^{-1/2}\partial _j (\gamma \gamma ^{ij})) \end{aligned}$$

through substituting in the Eq. (2.10) by also using the Eq. (2.7) on-shell. Notice that the resulting Hamiltonian shows first derivatives of the metric coefficients only. The same holds with the Hamiltonian proposed by Schwinger, see Eq. (2.29) and the Eq. (2.27) on-shell. The Hamiltonians (2.20), (2.26), and (2.29) are identical as global objects because their integrands differ by total divergences which do vanish after integration.

Schwinger proposed still another set of canonical field variables $(q^{ij},\varPi _{ij})$, for which the Hamiltonian and momentum densities have the form

2.27 $$\begin{aligned} \mathcal {H}&\equiv \frac{c^4}{16\pi G}\gamma ^{-1/2} \Big (-\frac{1}{4}q^{mn}\partial _m q^{kl}\partial _n q^{kl} - \frac{1}{2}q_{ln}\partial _m q^{kl}\partial _k q^{mn} \nonumber \\&\quad - \frac{1}{2}q^{kl}\partial _k \text{ ln }(q^{1/2})\partial _l \text{ ln }(q^{1/2}) + \partial _i\partial _j q^{ij} + q^{ik}q^{jl}\varPi _{ij}\varPi _{kl} - (q^{ij}\varPi _{ij})^2 \Big ) + {\mathcal {H}}_{\text {m}}, \end{aligned}$$

2.28 $$\begin{aligned} {\mathcal {H}}_i&\equiv \frac{c^3}{16\pi G}\Big [-\varPi _{lm}\partial _iq^{lm} + \partial _i(2\varPi _{lm}q^{lm}) - \partial _l(2\varPi _{im}q^{lm})\Big ] + {\mathcal {H}}_{\text {m}i}, \end{aligned}$$

where $\varPi _{ij}\equiv -\gamma ^{-1}(\pi _{ij} - \frac{1}{2}\pi \gamma _{ij})$, $q^{ij}\equiv \gamma \gamma ^{ij}$, $q\equiv \gamma ^2$; Schwinger’s canonical field momentum $\displaystyle \frac{c^3}{16\pi G}\varPi _{ij}$ is just $\displaystyle \frac{c^3}{16\pi G}\gamma ^{-1/2} K_{ij}$. The Poisson bracket for the Schwinger variables does have the same structure as the one for the ADM variables. The Schwinger’s reduced Hamiltonian has the form

2.29 $$\begin{aligned} H_{\mathrm{S}} = -\frac{c^4}{16\pi G}\oint _{i^0} \mathrm {d}S_i\, \partial _j q^{ij} = -\frac{c^4}{16\pi G}\int \mathrm {d}^3x\, \partial _i\partial _j q^{ij}. \end{aligned}$$

If Schwinger would have chosen coordinate conditions corresponding to those introduced above in Eqs. (2.14) (ADM also introduced another set of coordinate conditions to which Schwinger adjusted), namely

2.30 $$\begin{aligned} \varPi _{ii} = 0, \quad q^{ij} = \varphi \delta _{ij} + f^{ij}_{\mathrm{TT}}, \end{aligned}$$

a similar simple technical formalism convenient for practical calculations would have resulted with the independent field variables $\varPi _{ij}^{\mathrm{TT}}$ and $f^{ij}_{\mathrm{TT}}$. To our best knowledge, only the paper by Kibble (1963) delivers an application of Schwinger’s formalism, apart from Schwinger himself, namely a Hamiltonian formulation of the Dirac spinor field in gravity. Much later, Nelson and Teitelboim (1978) completed the same task within the tetrad-generalized Dirac formalism (Dirac 1962).

### Derivation of the ADM Hamiltonian

The ADM Hamiltonian was derived via the generator of field and spacetime-coordinates variations. Let the generator of general field variations be defined as (it corresponds to the generator $G\equiv p_i\,\delta x^i$ of the point-particle dynamics in classical mechanics with the particle’s canonical momentum $p_i$ and position $x^i$)

2.31 $$\begin{aligned} G_{\mathrm{field}} \equiv \frac{c^3}{16\pi G} \int \mathrm {d}^3x\, \pi ^{ij} \delta \gamma _{ij}. \end{aligned}$$

Let the coefficients of three space-metric $\gamma _{ij}$ be fixed by the relations (2.14), then the only free variations left are

2.32 $$\begin{aligned} G_{\mathrm{field}} = \frac{c^3}{16\pi G} \int \mathrm {d}^3x\, \pi ^{ij}_{\mathrm{TT}} \delta h_{ij}^{\mathrm{TT}} + \frac{c^3}{16\pi G} \int \mathrm {d}^3x\, \pi ^{jj} \delta \psi \end{aligned}$$

or, modulo a total variation,

2.33 $$\begin{aligned} G_{\mathrm{field}} = \frac{c^3}{16\pi G} \int \mathrm {d}^3x\, \pi ^{ij}_{\mathrm{TT}} \delta h_{ij}^{\mathrm{TT}} - \frac{c^3}{16\pi G} \int \mathrm {d}^3x\, \psi \delta \pi ^{jj}. \end{aligned}$$

It is consistent with the Einstein field equations in space-asymptotically flat space–time with quasi-Cartesian coordinates to put [the mathematically precise meaning of this equation is detailed in the Appendix B of Arnowitt et al. (1960a)]

2.34 $$\begin{aligned} ct = - \frac{1}{2}\varDelta ^{-1} \pi ^{jj}, \end{aligned}$$

which results in, dropping total space derivatives,

2.35 $$\begin{aligned} G_{\mathrm{field}} = \frac{c^3}{16\pi G} \int \mathrm {d}^3x\, \pi ^{ij}_{\mathrm{TT}} \delta h_{ij}^{\mathrm{TT}} + \frac{c^4}{8\pi G} \int \mathrm {d}^3x\, \varDelta \psi \, \delta t. \end{aligned}$$

Hereof the Hamiltonian easily follows in the form

2.36 $$\begin{aligned} H = -\frac{c^4}{8\pi G} \int \mathrm {d}^3x\,\varDelta \psi , \end{aligned}$$

which can also be written, using the form of the three-metric from Eq. (2.14),

2.37 $$\begin{aligned} H = \frac{c^4}{16\pi G} \int \mathrm {d}^3x\, \partial _i\partial _j (\gamma _{ij} - \delta _{ij}\gamma _{kk}). \end{aligned}$$

This expression is valid also in case of other coordinate conditions (Arnowitt et al. 1962). For the derivation of the generator of space translations, the reader is referred to Arnowitt et al. (1962) or, equivalently, to Schwinger (1963a).

## The ADM formalism for point-mass systems

### Reduced Hamiltonian for point-mass systems

In this section we consider the ADM canonical formalism applied to a system of self-gravitating nonrotating point masses (particles). The energy-momentum tensor of such system reads

3.1 $$\begin{aligned} T^{\alpha \beta }(x^\gamma ) = \sum _a m_a c \int _{-\infty }^\infty \frac{u_a^\alpha u_a^\beta }{\sqrt{-g}} \delta ^{(4)}\big (x^\mu -x_a^\mu (\tau _a)\big )\mathrm {d}\tau _a, \end{aligned}$$

where $m_a$ is the mass parameter of *a*th point mass ($a=1,2,\ldots$ labels the point masses), $u_a^\alpha \equiv \mathrm {d}x_a^\alpha /\mathrm {d}\tau _a$ (with $c\,\mathrm {d}\tau _a=\sqrt{-g_{\mu \nu }\mathrm {d}x_a^\mu \mathrm {d}x_a^\nu }$) is the four-velocity along the worldline $x^\mu =x_a^\mu (\tau _a)$ of the *a*th particle. After performing the integration in (3.1) one gets

3.2 $$\begin{aligned} T^{\alpha \beta }(\mathbf {x},t) = \sum _a m_a c \frac{u_a^\alpha u_a^\beta }{u_a^0 \sqrt{-g}} \delta ^{(3)}\big (\mathbf {x}-\mathbf {x}_a(t)\big ), \end{aligned}$$

where $\mathbf {x}_a=(x_a^i)$ is the position three-vector of the *a*th particle. The linear four-momentum of the *a*th particle equals $p_a^\alpha \equiv m_a u_a^\alpha$, and the three-momentum canonically conjugate to the position $\mathbf {x}_a$ comes out to be $\mathbf {p}_a=(p_{ai})$, where $p_{ai}=m_a u_{ai}$.

The action functional describing particles-plus-field system reads

3.3 $$\begin{aligned} S = \int \mathrm {d}t \left( \frac{c^3}{16\pi G} \int \mathrm {d}^3x\, \pi ^{ij} \partial _t \gamma _{ij} + \sum _a p_{ai} \dot{x}_a^i - H_0\right) , \end{aligned}$$

where $\dot{x}_a^i\equiv \mathrm {d}x_a^i/\mathrm {d}t$. The asymptotic value 1 of the lapse function enters as prefactor of the surface integral in the Hamiltonian $H_0$, which takes the form

3.4 $$\begin{aligned} H_0 = \int \mathrm {d}^3x\, (N{\mathcal {H}} - cN^i {\mathcal {H}}_i) + \frac{c^4}{16\pi G} \oint _{i^0}\mathrm {d}S_i\,\partial _j (\gamma _{ij} - \delta _{ij} \gamma _{kk}), \end{aligned}$$

where the so-called super-Hamiltonian density $\mathcal {H}$ and super-momentum density ${\mathcal {H}}_i$ can be computed by means of Eqs. (2.7)–(2.8), (2.11)–(2.12), and (3.2). They read [here we use the abbreviation $\delta _a$ for $\delta ^{(3)}(\mathbf {x}-\mathbf {x}_a)$]

3.5 $$\begin{aligned} \mathcal {H}&= \frac{c^4}{16\pi G} \left[ \frac{1}{\gamma ^{1/2}}\left( \pi ^i_j\pi ^j_i-\frac{1}{2}\pi ^2\right) - \gamma ^{1/2} R\right] + \sum _a c \left( m_a^2c^2 + \gamma _a^{ij}p_{ai}p_{aj}\right) ^{1/2} \delta _a, \end{aligned}$$

3.6 $$\begin{aligned} \mathcal {H}_i&= \frac{c^3}{8\pi G} \nabla _j \pi ^j_i + \sum _a p_{ai}\delta _a, \end{aligned}$$

where $\gamma _a^{ij}\equiv \gamma _{\text {reg}}^{ij}({\mathbf {x}}_a)$ is the finite part of the inverse metric evaluated at the particle position, which can be perturbatively and, using dimensional regularization, unambiguously defined (see Sects. 4.2, 4.3 below and Appendix A4 of Jaranowski and Schäfer 2015).

The evolutionary part of the field equations is obtained by varying the action functional (3.3) with respect to the field variables $\gamma _{ij}$ and $\pi ^{ij}$. The resulting equations read

3.7 $$\begin{aligned} \gamma _{ij,0}&= 2N \gamma ^{-1/2} \left( \pi _{ij} - \frac{1}{2} \pi \gamma _{ij} \right) + \nabla _iN_j + \nabla _jN_i, \end{aligned}$$

3.8 $$\begin{aligned} \pi ^{ij}_{~,0}&= -N \gamma ^{1/2} \left( R^{ij} - \frac{1}{2}\gamma ^{ij}R\right) + \frac{1}{2}N\gamma ^{-1/2}\gamma ^{ij}\left( \pi ^{mn}\pi _{mn} - \frac{1}{2}\pi ^2\right) \nonumber \\&\quad - 2 N \gamma ^{-1/2}\left( \pi ^{im}\pi ^j_m - \frac{1}{2}\pi \pi ^{ij} \right) + \nabla _m(\pi ^{ij}N^m) - (\nabla _m N^i)\pi ^{mj} \nonumber \\&\quad - (\nabla _mN^j)\pi ^{mi} + \frac{1}{2} \sum _a N_a \gamma ^{ik}_a p_{ak} \gamma ^{jl}_a p_{al} \left( \gamma ^{mn}_ap_{am}p_{an} + m^2_ac^2\right) ^{-1/2}\delta _a. \end{aligned}$$

The constraint part of the field equations results from varying the action (3.3) with respect to *N* and $N^i$. It has the form

3.9 $$\begin{aligned} {\mathcal {H}} = 0, \qquad {\mathcal {H}}_i = 0. \end{aligned}$$

The variation of the action (3.3) with respect to $\mathbf {x}_a$ and $\mathbf {p}_a$ leads to equations of motion for the particles,

3.10 $$\begin{aligned} \dot{p}_{ai}&= -\frac{\partial }{\partial x_a^i} \int \mathrm {d}^3x\, (N{\mathcal {H}} - cN^k {\mathcal {H}}_k) \nonumber \\&= c p_{aj} \frac{\partial N^j_a}{\partial x_a^i} - c \left( m_a^2c^2 + \gamma ^{kl}_a p_{ak}p_{al} \right) ^{1/2} \frac{\partial N_a}{\partial x_a^i} \nonumber \\&\quad - \frac{c N_a}{2\left( m_a^2c^2 + \gamma ^{mn}_a p_{am}p_{an} \right) ^{1/2}}\frac{\partial \gamma ^{kl}_a}{\partial x_a^i}p_{ak}p_{al}, \end{aligned}$$

3.11 $$\begin{aligned} \dot{x}_a^i&= \frac{\partial }{\partial p_{ai}} \int \mathrm {d}^3x\, \left( N{\mathcal {H}} - cN^k {\mathcal {H}}_k\right) \nonumber \\&= \frac{c N_a \gamma _a^{ij}p_{aj}}{\left( m_a^2c^2 + \gamma ^{kl}_a p_{ak}p_{al}\right) ^{1/2}} - cN^i_a. \end{aligned}$$

Notice the involvement of lapse and shift functions in the equations of motion. Both the lapse and shift functions, four functions in total, get determined by the application of the four coordinate conditions (2.14) to the field equations (3.7) and (3.8).

The reduced action, which is fully sufficient for the derivation of the dynamics of the particles and the gravitational field, reads (only the asymptotic value 1 of the shift function survives)

3.12 $$\begin{aligned} S = \int \mathrm {d}t \left[ \frac{c^3}{16\pi G} \int \mathrm {d}^3x\, \pi _{\mathrm{TT}}^{ij} \partial _t h^{\mathrm{TT}}_{ij} + \sum _a p_{ai} \dot{x}_a^i - H_{\text {red}} \right] , \end{aligned}$$

where both the constraint equations (3.9) and the coordinate conditions (2.14) are taken to hold. The reduced Hamilton functional $H_{\text {red}}$ is given by

3.13 $$\begin{aligned} H_{\text {red}}\big [{\mathbf {x}}_a,{\mathbf {p}}_a,h^{\mathrm{TT}}_{ij},\pi _{\mathrm{TT}}^{ij}\big ] = -\frac{c^4}{16\pi G} \int \mathrm {d}^3x\,\varDelta \phi \big [{\mathbf {x}}_a,{\mathbf {p}}_a,h^{\mathrm{TT}}_{ij},\pi _{\mathrm{TT}}^{ij}\big ]. \end{aligned}$$

The remaining field equations read

3.14 $$\begin{aligned} \frac{c^3}{16\pi G}\partial _t\pi _{\text {TT}}^{ij} = -\delta ^{\text {TT} {ij}}_{kl} \frac{\delta H_{\text {red}}}{\delta h_{kl}^{\text {TT}}}, \quad \frac{c^3}{16\pi G}\partial _t h_{ij}^{\text {TT}} = \delta ^{\text {TT} {kl}}_{ij} \frac{\delta H_{\text {red}}}{\delta \pi _{\text {TT}}^{kl}}, \end{aligned}$$

and the equations of motion for the point masses take the form

3.15 $$\begin{aligned} \dot{p}_{ai} = -\frac{\partial H_{\text {red}}}{\partial x_a^i}, \quad \quad \dot{x}_a^i = \frac{\partial H_{\text {red}}}{\partial p_{ai}}. \end{aligned}$$

Evidently, there is no involvement of lapse and shift functions in the equations of motion and in the field equations for the independent degrees of freedom (Arnowitt et al. 1960b; Kimura 1961).

### Routh functional

The Routh functional (or Routhian) of the system is defined by

3.16 $$\begin{aligned} R\big [{\mathbf {x}}_a,{\mathbf {p}}_a,h^{\mathrm{TT}}_{ij},\partial _t h^{\mathrm{TT}}_{ij}\big ] \equiv H_{\text {red}} - \frac{c^3}{16\pi G} \int \mathrm {d}^3x\,\pi ^{ij}_{\mathrm{TT}}\,\partial _t h^{\mathrm{TT}}_{ij}. \end{aligned}$$

This functional is a Hamiltonian for the point-mass degrees of freedom, and a Lagrangian for the independent gravitational field degrees of freedom. Within the post-Newtonian framework it was first introduced by Jaranowski and Schäfer (1998, 2000c). The evolution equation for the gravitational field degrees of freedom reads

3.17 $$\begin{aligned} \frac{\displaystyle \delta }{\delta h^{\mathrm{TT}}_{ij}(\mathbf{x},t)}\int R(t')\,\mathrm {d}t' = 0. \end{aligned}$$

The Hamilton equations of motion for the two point masses take the form

3.18 $$\begin{aligned} \dot{p}_{ai} = - \frac{\partial R}{\partial x^i_a}, \quad \dot{x}^i_a = \frac{\partial R}{\partial p_{ai}}. \end{aligned}$$

For the following treatment of the conservative part of the dynamics only, we will make now a short model calculation revealing the structure and logic behind the treatment. Let’s take a Routhian of the form $R(q,p;\xi ,{{\dot{\xi }}})$. Then the action reads

3.19 $$\begin{aligned} S[q,p;\xi ] = \int \big (p\dot{q} - R(q,p;\xi ,{\dot{\xi }})\big )\mathrm {d}t. \end{aligned}$$

Its variation through the independent variables gives

3.20 $$\begin{aligned} \delta S&= \int \bigg [\frac{\mathrm {d}}{\mathrm {d}t}(p\delta q) + \left( \dot{q}-\frac{\partial R}{\partial p}\right) \delta p + \left( -\dot{p}-\frac{\partial R}{\partial q}\right) \delta q \nonumber \\&\quad \quad - \left( \frac{\partial R}{\partial \xi }-\frac{\mathrm {d}}{\mathrm {d}t}\frac{\partial R}{\partial {\dot{\xi }}}\right) \delta \xi - \frac{\mathrm {d}}{\mathrm {d}t}\left( \frac{\partial R}{\partial {\dot{\xi }}}\delta \xi \right) \bigg ]\mathrm {d}t. \end{aligned}$$

Going on-shell with the $\xi$-dynamics yields

3.21 $$\begin{aligned} \delta S = \int \left[ \frac{\mathrm {d}}{\mathrm {d}t}(p\delta q) + \left( \dot{q}-\frac{\partial R}{\partial p}\right) \delta p + \left( -\dot{p} -\frac{\partial R}{\partial q}\right) \delta q\right] \mathrm {d}t - \left( \frac{\partial R}{\partial {\dot{\xi }}}\delta \xi \right) ^{+\infty }_{-\infty }. \end{aligned}$$

The vanishing of the last term means—thinking in terms of $h^{\mathrm{TT}}_{ij}$ and $\dot{h}^{\mathrm{TT}}_{ij}$, i.e. considering the term $(\int \mathrm {d}^3x\,\pi ^{ij}_{\mathrm{TT}}\,\delta h^{\mathrm{TT}}_{ij})^{+\infty }_{-\infty }$ on the solution space of the field equations (“on-field-shell”)—that as much incoming as outgoing radiation has to be present, or time-symmetric boundary conditions have to be applied. Thus in the Fokker-type procedure no dissipation shows up. This, however, does not force the use of the symmetric Green function, which would exclude conservative tail contributions at 4PN and higher PN orders. Assuming a leading-order-type prolongation of the form $R=R(q,p,\dot{q},\dot{p})$, the autonomous dynamics can be deduced from the variation

3.22 $$\begin{aligned} \delta S = \int \left[ \frac{\mathrm {d}}{\mathrm {d}t}(p\delta q) + \left( \dot{q}-\frac{\delta R}{\delta p}\right) \delta p + \left( -\dot{p}-\frac{\delta R}{\delta q}\right) \delta q \right] \mathrm {d}t, \end{aligned}$$

where the Euler–Lagrange derivative $\delta A/\delta z\equiv \partial A /\partial z-\mathrm {d}(\partial A/\partial \dot{z})/\mathrm {d}t$ has been introduced.

Having explained that, the *conservative* part of the binary dynamics is given by the higher-order Hamiltonian equal to the on-field-shell Routhian,

3.23 $$\begin{aligned} H_{\text {con}}[&{\mathbf {x}}_a,{\mathbf {p}}_a,\dot{\mathbf{x}}_a,\dot{\mathbf{p}}_a,\ldots ] \nonumber \\&\equiv R\big [{\mathbf {x}}_a,{\mathbf {p}}_a,h^{\mathrm{TT}}_{ij}({\mathbf {x}}_a,{\mathbf {p}}_a,\dot{\mathbf{x}}_a,\dot{\mathbf{p}}_a,\ldots ),\dot{h}^{\mathrm{TT}}_{ij}({\mathbf {x}}_a,{\mathbf {p}}_a,\dot{\mathbf{x}}_a,\dot{\mathbf{p}}_a,\ldots )\big ], \end{aligned}$$

where the field variables $h^{\mathrm{TT}}_{ij}$, $\dot{h}^{\mathrm{TT}}_{ij}$ were “integrated out”, i.e., replaced by their solutions as functionals of particle variables. The conservative equations of motion defined by the higher-order Hamiltonian (3.23) read

3.24 $$\begin{aligned} \dot{p}_{ai}(t) = -\frac{\delta }{\delta x^i_a(t)} \int H_{\text {con}}(t')\,\mathrm {d}t', \quad \dot{x}^i_a(t) = \frac{\delta }{\delta p_{ai}(t)} \int H_{\text {con}}(t')\,\mathrm {d}t', \end{aligned}$$

where the functional derivative is given by

3.25 $$\begin{aligned} \frac{\delta }{\delta z(t)} \int H_{\text {con}}(t')\,\mathrm {d}t' = \frac{\partial H_{\text {con}}}{\partial z(t)} - \frac{\mathrm {d}}{\mathrm {d}t}\frac{\partial H_{\text {con}}}{\partial \dot{z}(t)} + \cdots , \end{aligned}$$

with $z=x^i_a$ or $z=p_{ai}$. Schäfer (1984) and Damour and Schäfer (1991) show that time derivatives of ${\mathbf {x}}_a$ and ${\mathbf {p}}_a$ in the higher-order Hamiltonian (3.23) can be eliminated by the use of lower-order equations of motion, leading to an ordinary Hamiltonian,

3.26 $$\begin{aligned} H_{\text {con}}^{\text {ord}}[{\mathbf {x}}_a,{\mathbf {p}}_a] = H_{\text {con}}[{\mathbf {x}}_a,{\mathbf {p}}_a,\dot{\mathbf{x}}_a({\mathbf {x}}_a,{\mathbf {p}}_a),\dot{\mathbf{p}}_a({\mathbf {x}}_a,{\mathbf {p}}_a),\ldots ]. \end{aligned}$$

Notice the important point that the two Hamiltonians $H_{\text {con}}$ and $H_{\text {con}}^{\text {ord}}$ do not belong to the same coordinate system. Therefore, the Hamiltonians $H_{\text {con}}$ and $H_{\text {con}}^{\text {ord}}$ and their variables should have, say, primed and unprimed notations which usually however does not happen in the literature due to a slight abuse of notation.

A formal PN expansion of the Routh functional in powers of $1/c^2$ is feasible to all PN orders. With the aid of the definition $\displaystyle h^{\mathrm{TT}}_{ij}\equiv \frac{16\pi G}{c^4}{\hat{h}}^{\mathrm{TT}}_{ij}$, we may write

3.27 $$\begin{aligned} R\big [{\mathbf {x}}_a,{\mathbf {p}}_a, h^{\mathrm{TT}}_{ij}, \partial _th^{\mathrm{TT}}_{ij}\big ] - \sum _a m_a c^2 = \sum _{n=0}^{\infty } \frac{1}{c^{2n}} R_n\big [{\mathbf {x}}_a,{\mathbf {p}}_a, {\hat{h}}^{\mathrm{TT}}_{ij}, \partial _t{{\hat{h}}}^{\mathrm{TT}}_{ij}\big ]. \end{aligned}$$

Hereof, the field equation for $h^{\mathrm{TT}}_{ij}$ results in a PN-series form,

3.28 $$\begin{aligned} \left( \varDelta -\frac{1}{c^2}\partial _t^2\right) {\hat{h}}^{\mathrm{TT}}_{ij} = \sum _{n=0}^{\infty } \frac{1}{c^{2n}} D^{\mathrm{TT}}_{(n)ij}\big [\mathbf{x},{\mathbf {x}}_a,{\mathbf {p}}_a, {\hat{h}}^{\mathrm{TT}}_{kl}, \partial _t{{\hat{h}}}^{\mathrm{TT}}_{kl}\big ]. \end{aligned}$$

This equation must now be solved step by step using either retarded integrals for getting the whole dynamics or time-symmetric ones for only the conservative dynamics defined by $H_{\text {con}}$, which themselves have to be expanded in powers of 1 / *c*. In higher orders, however, non-analytic in 1 / *c* log-terms do show up (see, e.g., Damour et al. 2014, 2016).

To calculate the reduced Hamiltonian of Eq. (2.21) for a many-particle system one has to perturbatively solve for $\phi$ and ${\tilde{\pi }}^ {ij}$ the constraint equations $\mathcal {H}=0$ and $\mathcal {H}_i=0$ with the densities $\mathcal {H}$, $\mathcal {H}_i$ defined in Eqs. (3.5)–(3.6). Then the transition to the Routhian of Eq. (3.16) is straightforward using the second equation in (3.14). The expansion of the Hamiltonian constraint equation up to $c^{-10}$ leads to the following equation [in this equation and in the next one we use units $c=1$, $G=1/(16\pi )$]^3^:

3.29 $$\begin{aligned} -\varDelta \phi&= \sum _a \bigg [ 1-\frac{1}{8}\phi +\frac{1}{64}\phi ^2-\frac{1}{512}\phi ^3+\frac{1}{4096}\phi ^4 \nonumber \\&\quad + \left( \frac{1}{2}-\frac{5}{16}\phi +\frac{15}{128}\phi ^2-\frac{35}{1024}\phi ^3\right) \frac{\mathbf{p}_a^2}{m_a^2} \nonumber \\&\quad + \left( -\frac{1}{8}+\frac{9}{64}\phi -\frac{45}{512}\phi ^2\right) \frac{(\mathbf{p}_a^2)^2}{m_a^4} +\left( \frac{1}{16}-\frac{13}{128}\phi \right) \frac{(\mathbf{p}_a^2)^3}{m_a^6} -\frac{5}{128}\frac{(\mathbf{p}_a^2)^4}{m_a^8} \nonumber \\&\quad +\left( -\frac{1}{2}+\frac{9}{16}\phi +\frac{1}{4}\frac{\mathbf{p}_a^2}{m_a^2}\right) \frac{p_{ai}p_{aj}}{m_a^2}{h^\mathrm{TT}_{ij}} -\frac{1}{16}\left( {h^\mathrm{TT}_{ij}}\right) ^2 \bigg ] m_a\delta _a \nonumber \\&\quad +\left( 1+\frac{1}{8}\phi \right) \left( {\widetilde{\pi }^{ij}}\right) ^2 +\left( 2+\frac{1}{4}\phi \right) {\widetilde{\pi }^{ij}}{\pi ^{ij}_{\mathrm{TT}}} +\left( {\pi ^{ij}_{\mathrm{TT}}}\right) ^2 \nonumber \\&\quad +\left[ \left( -\frac{1}{2}+\frac{1}{4}\phi -\frac{5}{64}\phi ^2\right) \phi _{,{ij}} +\left( \frac{3}{16}-\frac{15}{128}\phi \right) \phi _{,i}\phi _{,j} +2{\widetilde{\pi }^{ik}}{\widetilde{\pi }^{jk}}\right] {h^\mathrm{TT}_{ij}} \nonumber \\&\quad +\left( \frac{1}{4}-\frac{7}{32}\phi \right) \left( {h^{\mathrm{TT}}_{ij,k}}\right) ^2 +\left( \frac{1}{2}+\frac{1}{16}\phi \right) {h^{\mathrm{TT}}_{ij,k}}{h^{\mathrm{TT}}_{ik,j}} \nonumber \\&\quad + \varDelta \left[ \left( -\frac{1}{2}+\frac{7}{16}\phi \right) \left( {h^\mathrm{TT}_{ij}}\right) ^2\right] -\left[ \frac{1}{2}\phi {h^\mathrm{TT}_{ij}}{h^{\mathrm{TT}}_{ik,j}} +\frac{1}{4}\phi _{,k}\left( {h^\mathrm{TT}_{ij}}\right) ^2\right] _{,k} \nonumber \\&\quad + \mathcal {O}(c^{-12}). \end{aligned}$$

The expansion of the momentum constraint equation up to $c^{-7}$ reads

3.30 $$\begin{aligned} {\widetilde{\pi }^{ij}}_{,j}&= \left( -\frac{1}{2}+\frac{1}{4}\phi -\frac{5}{64}\phi ^2\right) \sum _a p_{ai}\delta _a + \left( -\frac{1}{2}+\frac{1}{16}\phi \right) \phi _{,j}{\widetilde{\pi }^{ij}} \nonumber \\&\quad - \frac{1}{2}\phi _{,j}{\pi ^{ij}_{\mathrm{TT}}} - {\widetilde{\pi }^{jk}}_{,k}{h^\mathrm{TT}_{ij}} + {\widetilde{\pi }^{jk}}\left( \frac{1}{2}{h^{\mathrm{TT}}_{jk,i}}-{h^{\mathrm{TT}}_{ij,k}}\right) + \mathcal {O}(c^{-8}). \end{aligned}$$

In the Eqs. (3.29) and (3.30) dynamical field variables $h^{\mathrm{TT}}_{ij}$ and $\pi ^{ij}_{\mathrm{TT}}$ are counted as being of the orders $1/c^4$ and $1/c^5$, respectively [cf. Eq. (3.28)].

### Poincaré invariance

In asymptotically flat spacetimes the Poincaré group is a global symmetry group. Its generators $P^{\mu }$ and $J^{\mu \nu }$ are realized as functions $P^\mu ({\mathbf {x}}_a,{\mathbf {p}}_a)$ and $J^{\mu \nu }({\mathbf {x}}_a,{\mathbf {p}}_a)$ on the many-body phase-space. They are conserved on shell and fulfill the Poincaré algebra relations for the Poisson bracket product (see, e.g., Regge and Teitelboim 1974),

3.31 $$\begin{aligned} \{ P^{\mu }, P^{\nu } \}&= 0, \end{aligned}$$

3.32 $$\begin{aligned} \{ P^{\mu }, J^{\rho \sigma } \}&= -\eta ^{\mu \rho } P^{\sigma } + \eta ^{\mu \sigma } P^{\rho }, \end{aligned}$$

3.33 $$\begin{aligned} \{ J^{\mu \nu }, J^{\rho \sigma } \}&= -\eta ^{\nu \rho } J^{\mu \sigma } + \eta ^{\mu \rho } J^{\nu \sigma } + \eta ^{\sigma \mu } J^{\rho \nu } - \eta ^{\sigma \nu } J^{\rho \mu }, \end{aligned}$$

where the Poisson brackets are defined in an usual way,

3.34 $$\begin{aligned} \{A,B\} \equiv \sum _a \left( \frac{\partial A}{\partial x_a^i}\frac{\partial B}{\partial p_{ai}} - \frac{\partial A}{\partial p_{ai}}\frac{\partial B}{\partial x_a^i} \right) . \end{aligned}$$

The meaning of the components of $P^{\mu }$ and $J^{\mu \nu }$ is as follows: the time component $P^0$ (i.e., the total energy) is realized as the Hamiltonian $H\equiv c P^0$, $P^i=P_i$ is linear momentum, $J^i\equiv \frac{1}{2}\varepsilon ^{ikl}J_{kl}$ [with $\varepsilon ^{ijk}\equiv \varepsilon _{ijk}\equiv \frac{1}{2}(i-j)(j-k)(k-i)$, $J_{kl}=J^{kl}$, and $J_{ij}=\varepsilon _{ijk}J^k$] is angular momentum, and Lorentz boost vector is $K^i\equiv J^{i0}/c$. The boost vector represents the constant of motion associated to the centre-of-mass theorem and can further be decomposed as $K^i=G^i-t\,P^i$ (with $G_i=G^i$). In terms of three-dimensional quantities the Poincaré algebra relations read (see, e.g., Damour et al. 2000c, d)

3.35 $$\begin{aligned} \{ P_i, H \}&= 0, \quad \{ J_i, H \} = 0, \end{aligned}$$

3.36 $$\begin{aligned} \{ J_i, P_j \}&= \varepsilon _{ijk} \, P_k, \quad \{ J_i, J_j \} = \varepsilon _{ijk} \, J_k, \end{aligned}$$

3.37 $$\begin{aligned} \{ J_i, G_j \}&= \varepsilon _{ijk} \, G_k, \end{aligned}$$

3.38 $$\begin{aligned} \{ G_i, H \}&= P_i, \end{aligned}$$

3.39 $$\begin{aligned} \{ G_i, P_j \}&= \frac{1}{c^2}\,H\,\delta _{ij}, \end{aligned}$$

3.40 $$\begin{aligned} \{ G_i, G_j \}&= -\frac{1}{c^2}\,\varepsilon _{ijk}\,J_k. \end{aligned}$$

The Hamiltonian *H* and the centre-of-mass vector $G^i$ have the integral representations

3.41 $$\begin{aligned} H&= -\frac{c^4}{16 \pi G} \int \mathrm {d}^3x\,\varDelta \phi = -\frac{c^4}{16 \pi G} \oint _{i^0} r^2 \mathrm {d}\varOmega \,{\mathbf {n}}\cdot \nabla \phi , \end{aligned}$$

3.42 $$\begin{aligned} G^i&= -\frac{c^2}{16 \pi G} \int \mathrm {d}^3x\, x^i \varDelta \phi = -\frac{c^2}{16 \pi G} \oint _{i^0} r^2\mathrm {d}\varOmega \, n^j (x^i \partial _j - \delta _{ij})\phi , \end{aligned}$$

where ${\mathbf {n}}\,r^2\mathrm {d}\varOmega$ (${\mathbf {n}}$ is the radial unit vector) is the two-dimensional surface-area element at $i^0$. The two quantities *H* and $G^i$ are the most involved ones of those entering the Poincaré algebra.

The Poincaré algebra has been extensively used in the calculations of PN Hamiltonians for spinning binaries (Hergt and Schäfer 2008a, b). Hereby the most useful equation was (3.38), which tells that the total linear momentum has to be a total time derivative. This equation was also used by Damour et al. (2000c, 2000d) to fix the so called “kinetic ambiguity” in the 3PN ADM two-point-mass Hamiltonian without using dimensional regularization. In harmonic coordinates, the kinetic ambiguity got fixed by a Lorentzian version of the Hadamard regularization based on the Fock–de Donder approach (Blanchet and Faye 2001b).

The explicit form of the generators $P^\mu ({\mathbf {x}}_a,{\mathbf {p}}_a)$ and $J^{\mu \nu }({\mathbf {x}}_a,{\mathbf {p}}_a)$ (i.e., $\mathbf {P}$, $\mathbf {J}$, $\mathbf {G}$, and *H*) for two-point-mass systems is given in Appendix C with 4PN accuracy.

The global Lorentz invariance results in the following useful expressions (see, e.g., Rothe and Schäfer 2010; Georg and Schäfer 2015). Let us define the quantity *M* through the relation

3.43 $$\begin{aligned} Mc^2 \equiv \sqrt{H^{2}-\mathbf{{P}}^{2}c^{2}} \quad \text{ or } \quad H = \sqrt{M^{2}c^{4}+\mathbf{{P}}^{2}c^{2}}, \end{aligned}$$

and let us introduce the canonical centre of the system vector $\mathbf{X}$ (with components $X^i = X_i$),

3.44 $$\begin{aligned} \mathbf{X} \equiv \frac{\mathbf{G}c^2}{H}+\frac{1}{M\left( H+Mc^{2}\right) }\left( \mathbf{J} - \left( \frac{\mathbf{G}c^2}{H}\times \mathbf{P}\right) \right) \times \mathbf{P}. \end{aligned}$$

Then the following commutation relations are fulfiled:

3.45 $$\begin{aligned} \left\{ X_i,\, P_j \right\}&= \delta _{ij}, \quad \left\{ X_i,\, X_j \right\} = 0, \quad \left\{ P_i,\, P_j \right\} = 0, \end{aligned}$$

3.46 $$\begin{aligned} \left\{ M,\, P_i \right\}&= 0, \quad \left\{ M,\, X_i \right\} = 0, \end{aligned}$$

3.47 $$\begin{aligned} \left\{ M,\, H \right\}&= 0, \quad \left\{ P_i,\, H \right\} = 0, \quad \frac{H}{c^2}\left\{ X_i,\, H \right\} = P_i. \end{aligned}$$

The commutation relations clearly show the complete decoupling of the internal dynamics from the external one by making use of the canonical variables. The equations (3.43) additionally indicate that $M^2$ is simpler (or, more primitive) than *M*, cf. Georg and Schäfer (2015). A centre-of-energy vector can be defined by $X_E^i = X_{Ei} = c^2 G^i / H = c^2 G_i / H$. This vector, however, is not a canonical position vector, see, e.g., Hanson and Regge (1974).

In view of our later treatment of particles with spin, let us decompose the total angular momentum $J^{\mu \nu }$ of a single object into orbital angular momentum $L^{\mu \nu }$ and spin $S^{\mu \nu }$, both of them are anti-symmetric tensors,

3.48 $$\begin{aligned} J^{\mu \nu } = L^{\mu \nu } + S^{\mu \nu }. \end{aligned}$$

The orbital angular momentum tensor is given by

3.49 $$\begin{aligned} L^{\mu \nu } = Z^{\mu }P^{\nu } - Z^{\nu }P^{\mu }, \end{aligned}$$

where $Z^{\mu }$ denotes 4-dimensional position vector (with $Z^0 = ct$). The splitting in space and time results in

3.50 $$\begin{aligned} J^{ij} = Z^iP^j - Z^jP^i + S^{ij}, \quad J^{i0} = Z^iH/c - P^ict + S^{i0}. \end{aligned}$$

Remarkably, relativity tells us that any object with mass *M*, spin length *S*, and positive energy density must have extension orthogonal to its spin vector of radius of at least *S* / (*Mc*) (see, e.g., Misner et al. 1973). Clearly then, the position vector of such an object is not given a priori but must be defined. As the total angular momentum should not depend on the fixation of the position vector, the notion of spin must depend on the fixation of the position vector and vice versa. Thus, imposing a *spin supplementary condition* (SSC) fixes the position vector. We enumerate here the most often used SSCs (see, e.g., Fleming 1965; Hanson and Regge 1974; Barker and O’Connell 1979).

(i) Covariant SSC (also called Tulczyjew-Dixon SSC):
  3.51 $$\begin{aligned} P_{\nu }S^{\mu \nu } = 0. \end{aligned}$$
  The variables corresponding to this SSC are denoted in Sect. 7 by $Z^i = z^i$, $S^{ij}$, and $P^i = p^i$.
(ii) Canonical SSC (also called Newton–Wigner SSC):
  3.52 $$\begin{aligned} (P_{\nu } + Mc~n_{\nu })S^{\mu \nu } = 0, \quad Mc = \sqrt{-P_{\mu }P^{\mu }}, \end{aligned}$$
  where $n_{\mu } = (-1,0,0,0)$, $n_{\mu }n^{\mu } = -1$. The variables corresponding to this SSC are denoted in Sect. 7 by ${\hat{z}}^i$, ${\hat{S}}^{ij}$, and $P^i$.
(iii) Centre-of-energy SSC (also called Corinaldesi–Papapetrou SSC):
  3.53 $$\begin{aligned} n_{\nu }S^{\mu \nu } = 0. \end{aligned}$$
  Here the boost vector takes the form of a spinless object, $K^i = Z^iH/c^2 - P^it = G^i - P^it$.

### Poynting theorem of GR

Let us start with the following local identity, having structure of a Poynting theorem for GR in local form,

3.54 $$\begin{aligned} -\dot{h}^{\text {TT}}_{ij} \Box h^{\text {TT}}_{ij} = -\partial _k\left( \dot{h}^{\text {TT}}_{ij}h^{\text {TT}}_{ij,k}\right) + \frac{1}{2}\partial _t \left[ (\dot{h}^{\text {TT}}_{ij}/c)^2 + (h^{\text {TT}}_{ij,k})^2\right] , \end{aligned}$$

where $\Box \equiv -\partial _t^2/c^2+\varDelta$ denotes the d’Alembertian. Integrating this equation over whole space gives, assuming past stationarity,

3.55 $$\begin{aligned} -\int _{V_\infty }\mathrm {d}^3x\, \dot{h}^{\text {TT}}_{ij} \Box h^{\text {TT}}_{ij} = \frac{1}{2} \int _{V_\infty }\mathrm {d}^3x\,\partial _t \left[ (\dot{h}^{\text {TT}}_{ij}/c)^2 + (h^{\text {TT}}_{ij,k})^2\right] , \end{aligned}$$

where $V_\infty$ is just another expression for ${{\mathbb {R}}}^3$. Notice that the far zone is understood as area of the *t* = const slice where gravitational waves are decoupled from their source and do freely propagate outwards, what means that the relation $h^{\text {TT}}_{ij,k}=-(n^k/c)\dot{h}^{\text {TT}}_{ij}+\mathcal {O}(r^{-2})$ is fulfilled in the far or wave zone. Using

3.56 $$\begin{aligned} -\int _{V_{\mathrm{fz}}}\mathrm {d}^3x\, \dot{h}^{\text {TT}}_{ij} \Box h^{\text {TT}}_{ij} = -\oint _{\mathrm{fz}} \mathrm {d}s_k\,\dot{h}^{\text {TT}}_{ij}h^{\text {TT}}_{ij,k} + \frac{1}{2} \int _{V_{\mathrm{fz}}}\mathrm {d}^3x\, \partial _t \left[ (\dot{h}^{\text {TT}}_{ij}/c)^2 + (h^{\text {TT}}_{ij,k})^2\right] , \end{aligned}$$

with $V_{\mathrm{fz}}$ as the volume of the space enclosed by the outer boundary of the far (or, wave) zone (fz) and $\mathrm {d}s_k = n^k r^2 \mathrm {d}\varOmega$ surface-area element of the two-surface of integration with $\mathrm {d}\varOmega$ as the solid-angle element and *r* the radial coordinate, it follows

3.57 $$\begin{aligned} -\int _{(V_\infty - V_{\mathrm{fz}})}\mathrm {d}^3x\, \dot{h}^{\text {TT}}_{ij} \Box h^{\text {TT}}_{ij} =&\oint _{\mathrm{fz}}\mathrm {d}s_k\,\dot{h}^{\text {TT}}_{ij}h^{\text {TT}}_{ij,k} \nonumber \\&\quad + \frac{1}{2} \int _{(V_\infty -V_{\mathrm{fz}})}\mathrm {d}^3x\, \partial _t \left[ (\dot{h}^{\text {TT}}_{ij}/c)^2 + (h^{\text {TT}}_{ij,k})^2\right] . \end{aligned}$$

Dropping the left side of this equation as negligibly small, assuming the source term for $\Box h^{\text {TT}}_{ij}$, which follows from the Routhian field equation (3.17), to decay at least as $1/r^3$ for $r\rightarrow \infty$ (for isolated systems, all source terms for $\Box h^{\text {TT}}_{ij}$ decay at least as $1/r^4$ if not TT-projected; the TT-projection may raise the decay to $1/r^3$, e.g. TT-projection of Dirac delta function), results in

3.58 $$\begin{aligned} \frac{c^3}{32\pi G}\oint _{\mathrm{fz}} \mathrm {d}\varOmega \, r^2 \Big (\dot{h}_{ij}^{\mathrm{TT}}\Big )^2 = \frac{c^2}{32\pi G}\frac{\mathrm {d}}{\mathrm {d}t} \int _{(V_\infty - V_{\mathrm{fz}})}\mathrm {d}^3x\, \Big (\dot{h}_{ij}^{\mathrm{TT}}\Big )^2, \end{aligned}$$

with meaning that the energy flux through a surface in the far zone equals the growth of gravitational energy beyond that surface.

### Near-zone energy loss and far-zone energy flux

The change in time of the matter Routhian reads, assuming ${{\mathcal {R}}}$ to be *local* in the gravitational field,

3.59 $$\begin{aligned} \frac{\mathrm {d}R}{\mathrm {d}t} = \frac{\partial R}{\partial t} = \int \mathrm {d}^3x\, \frac{\partial {{\mathcal {R}}}}{\partial h^{\text {TT}}_{ij}} \dot{h}^{\text {TT}}_{ij} + \int \mathrm {d}^3x\, \frac{\partial {{\mathcal {R}}}}{\partial h^{\text {TT}}_{ij,k}} \partial _k \dot{h}^{\text {TT}}_{ij} + \int \mathrm {d}^3x\, \frac{\partial {{\mathcal {R}}}}{\partial \dot{h}^{\text {TT}}_{ij}} \ddot{h}^{\text {TT}}_{ij}, \end{aligned}$$

where

3.60 $$\begin{aligned} R({\mathbf {x}}_a,{\mathbf {p}}_a,t) \equiv \int \mathrm {d}^3x\, \mathcal {R}({\mathbf {x}}_a,{\mathbf {p}}_a,h^{\text {TT}}_{ij}(t),h^{\text {TT}}_{ij,k}(t),\dot{h}^{\text {TT}}_{ij}(t)). \end{aligned}$$

The equation for $\mathrm {d}R/\mathrm {d}t$ is valid provided the equations of motion

3.61 $$\begin{aligned} {\dot{p}}_{ai} = -\frac{\partial R}{\partial x_a^i}, \quad {\dot{x}}_a^i = \frac{\partial R}{\partial p_{ai}} \end{aligned}$$

hold. Furthermore, we have

3.62 $$\begin{aligned}&\int \mathrm {d}^3x\, \frac{\partial {{\mathcal {R}}}}{\partial h^{\text {TT}}_{ij,k}} \partial _k \dot{h}^{\text {TT}}_{ij} + \int \mathrm {d}^3x\, \frac{\partial {{\mathcal {R}}}}{\partial \dot{h}^{\text {TT}}_{ij}} \ddot{h}^{\text {TT}}_{ij} = \int \mathrm {d}^3x\, \partial _k\left( \frac{\partial {{\mathcal {R}}}}{\partial h^{\text {TT}}_{ij,k}} \dot{h}^{\text {TT}}_{ij}\right) \nonumber \\&\quad + \frac{\mathrm {d}}{\mathrm {d}t} \int \mathrm {d}^3x\, \frac{\partial {{\mathcal {R}}}}{\partial \dot{h}^{\text {TT}}_{ij}} \dot{h}^{\text {TT}}_{ij} - \int \mathrm {d}^3x\, \partial _k\left( \frac{\partial {{\mathcal {R}}}}{\partial h^{\text {TT}}_{ij,k}}\right) \dot{h}^{\text {TT}}_{ij} - \int \mathrm {d}^3x\, \frac{\mathrm {d}}{\mathrm {d}t}\left( \frac{\partial {{\mathcal {R}}}}{\partial \dot{h}^{\text {TT}}_{ij}}\right) \dot{h}^{\text {TT}}_{ij}. \end{aligned}$$

The canonical field momentum is given by

3.63 $$\begin{aligned} \frac{c^3}{16\pi G} \pi ^{ij}_{\text {TT}} = -\delta ^{\text {TT}ij}_{kl}\frac{\partial {{\mathcal {R}}}}{\partial \dot{h}^{\text {TT}}_{kl}}. \end{aligned}$$

Performing the Legendre transformation

3.64 $$\begin{aligned} H = R + \frac{c^3}{16\pi G} \int \mathrm {d}^3x\,\pi ^{ij}_{\text {TT}}\dot{h}^{\text {TT}}_{ij}, \quad \text{ or } \quad R = H - \frac{c^3}{16\pi G} \int \mathrm {d}^3x\,\pi ^{ij}_{\text {TT}}\dot{h}^{\text {TT}}_{ij}, \end{aligned}$$

the energy loss equation takes the form [using Eq. (3.59) together with (3.62) and (3.63)]

3.65 $$\begin{aligned} \frac{\mathrm {d}H}{\mathrm {d}t}&= \int \mathrm {d}^3x\, \partial _k\left( \frac{\partial {{\mathcal {R}}}}{\partial h^{\text {TT}}_{ij,k}} \dot{h}^{\text {TT}}_{ij}\right) + \int \mathrm {d}^3x\, \frac{\partial {{\mathcal {R}}}}{\partial h^{\text {TT}}_{ij}} \dot{h}^{\text {TT}}_{ij} \nonumber \\&\quad - \int \mathrm {d}^3x\, \partial _k\left( \frac{\partial {{\mathcal {R}}}}{\partial h^{\text {TT}}_{ij,k}}\right) \dot{h}^{\text {TT}}_{ij} - \int \mathrm {d}^3x\, \frac{\mathrm {d}}{\mathrm {d}t}\left( \frac{\partial {{\mathcal {R}}}}{\partial \dot{h}^{\text {TT}}_{ij}}\right) \dot{h}^{\text {TT}}_{ij}. \end{aligned}$$

Application of the field equations

3.66 $$\begin{aligned} \frac{\partial {{\mathcal {R}}}}{\partial h^{\text {TT}}_{ij}} - \partial _k\left( \frac{\partial {{\mathcal {R}}}}{\partial h^{\text {TT}}_{ij,k}}\right) - \frac{\mathrm {d}}{\mathrm {d}t}\left( \frac{\partial \mathcal {R}}{\partial \dot{h}^{\text {TT}}_{ij}}\right) = 0 \end{aligned}$$

yields, assuming past stationarity [meaning that at any finite time *t* no radiation can have reached spacelike infinity, so the first (surface) term in the right-hand side of Eq. (3.65) vanishes],

3.67 $$\begin{aligned} \frac{\mathrm {d}H}{\mathrm {d}t} = 0. \end{aligned}$$

The Eq. (3.58) shows that the Eq. (3.64) infers, employing the leading-order quadratic field structure of ${{\mathcal {R}}}\,\,\big [\mathcal {R}=-(1/4)(c^2/(16\pi G))(\dot{h}^{\text {TT}}_{ij})^2+\cdots$; see Eq. (F.3)$\big ]$,

3.68 $$\begin{aligned} \frac{\mathrm {d}}{\mathrm {d}t}\left( R - \int _{V_\text {fz}}\mathrm {d}^3x\,\frac{\partial {{\mathcal {R}}}}{\partial \dot{h}^{\text {TT}}_{ij}}\dot{h}^{\text {TT}}_{ij}\right) = -{\mathcal {L}}, \end{aligned}$$

where

3.69 $$\begin{aligned} {{\mathcal {L}}} = -\frac{c^4}{32\pi G} \oint _{\mathrm{fz}} \mathrm {d}s_k h^{\text {TT}}_{ij,k} \dot{h}^{\text {TT}}_{ij} = \frac{c^3}{32\pi G} \oint _{\mathrm{fz}} \mathrm {d}\varOmega \, r^2 \Big (\dot{h}^{\text {TT}}_{ij}\Big )^2 \end{aligned}$$

is the well known total energy flux (or luminosity) of gravitational waves. The Eq. (3.68) can be put into the energy form, again employing the leading-order quadratic field structure of ${{\mathcal {R}}}$,

3.70 $$\begin{aligned} \frac{\mathrm {d}}{\mathrm {d}t}\left( H - \frac{c^2}{32\pi G}\int _{(V_\infty -V_\text {fz})}\mathrm {d}^3x\,\Big (\dot{h}^{\text {TT}}_{ij}\Big )^2\right) = -{\mathcal {L}}. \end{aligned}$$

Taking into account the Eqs. (3.29) and (3.41) we find that the second term in the parenthesis of the left side of Eq. (3.70) exactly subtracts the corresponding terms from pure $(h^{\mathrm{TT}}_{ij,k})^2$ and $(\pi _{\mathrm{TT}}^{ij})^2$ expressions therein. This improves, by one order in radial distance, the large distance decay of the integrand of the integral of the whole left side of Eq. (3.70), which runs over the whole hypersurface *t* = const. We may now perform near- and far-zone PN expansions of the left and right sides of the Eq. (3.70), respectively. Though the both series are differently defined—on the left side, expansion in powers of 1 / *c* around fixed time *t* of an energy expression which is time differentiated; on the right side, expansion in powers of 1 / *c* around fixed retarded time $t-r/c$—the expansions cannot contradict each other as long as they are not related term by term. For the latter relation we must keep in mind that PN expansions are instantaneous expansions so that the two times, *t* and $t-r/c$, are not allowed to be located too far apart from each other. This means that we have to read off the radiation right when it enters far zone. Time-averaging of the expressions on the both sides of Eq. (3.70) over several wave periods makes the difference between the two times negligible as it should be if one is interested in a one-to-one correspondence between the terms on the both sides. The Newtonian and 1PN wave generation processes were explicitly shown to fit into this scheme by Königsdörffer et al. (2003).

### Radiation field

In the far zone, the multipole expansion of the transverse-traceless (TT) part of the gravitational field, obtained by algebraic projection with

3.71 $$\begin{aligned} P_{ijkl}(\mathbf {n})&\equiv \frac{1}{2}\Big (P_{ik}(\mathbf {n})P_{jl}(\mathbf {n}) + P_{il}(\mathbf {n})P_{jk}(\mathbf {n}) - P_{ij}(\mathbf {n})P_{kl}(\mathbf {n}\Big ), \end{aligned}$$

3.72 $$\begin{aligned} P_{ij}(\mathbf {n})&\equiv \delta _{ij} - n_i n_j, \end{aligned}$$

where ${\mathbf {n}}\equiv \mathbf {x}/r$ ($r\equiv |\mathbf {x}|$) is the unit vector in the direction from the source to the far away observer, reads (see, e.g., Thorne 1980; Blanchet 2014)

3.73 $$\begin{aligned} h_{ij}^{\mathrm{TT\,fz}}(\mathbf {x},t)&= \frac{G}{c^4} \frac{P_{ijkm}(\mathbf {n})}{r} \sum _{l=2}^{\infty } \left\{ \left( \frac{1}{c^2}\right) ^{\frac{l-2}{2}} \frac{4}{l!}\, \text{ M }^{(l)}_{kmi_3 \ldots i_l}\left( t-\frac{r_*}{{c}}\right) ~N_{i_3 \ldots i_l} \right. \nonumber \\&\quad + \left. \left( \frac{1}{{c^2}}\right) ^{\frac{l-1}{2}} \frac{8l}{(l+1)!}\, \varepsilon _{pq(k} \text{ S }^{(l)}_{m)pi_3 \ldots i_l}\left( t-\frac{r_*}{{c}}\right) ~n_q~N_{i_3 \dots i_l}\right\} , \end{aligned}$$

where $N_{i_3 \dots i_l}\equiv n^{i_3}\ldots n^{i_l}$ and where $\text{ M }^{(l)}_{i_1i_2i_3\ldots i_l}$ and $\text{ S }^{(l)}_{i_1i_2i_3\ldots i_l}$ denote the *l*th time derivatives of the symmetric and tracefree (STF) radiative mass-type and current-type multipole moments, respectively. The term with the leading mass-quadrupole tensor takes the form (see, e.g., Schäfer 1990)

3.74 $$\begin{aligned} \text{ M }_{ij}^{(2)}&\left( t-\frac{r_*}{c}\right) = \widehat{\text{ M }}_{ij}^{(2)}\left( t - \frac{r_*}{c}\right) \nonumber \\&\quad + \frac{2Gm}{c^3} \int _0^{\infty } \mathrm {d}v \left[ \ln \left( \frac{v}{2b}\right) + \kappa \right] \widehat{\text{ M }}^{(4)}_{ij}\left( t-\frac{r_*}{c}-v\right) + {{\mathcal {O}}}\left( \frac{1}{c^4}\right) , \end{aligned}$$

with

3.75 $$\begin{aligned} r_* = r + \frac{2Gm}{c^2}\text{ ln }\left( \frac{r}{cb}\right) + {{\mathcal {O}}}\left( \frac{1}{c^3}\right) \end{aligned}$$

showing the leading-order tail term of the quadrupole radiation (the gauge dependent relative phase constant $\kappa$ between direct and tail term was not explored by Schäfer 1990; for more details see, e.g., Blanchet and Schäfer 1993; Blanchet 2014). Notice the modification of the standard PN expansion through tail terms. This expression nicely shows that also multipole expansions in the far zone do induce PN expansions. The mass-quadrupole tensor $\widehat{\text{ M }}_{ij}$ is just the standard Newtonian one. Higher-order tail terms up to “tails-of-tails-of-tails” can be found in Marchand et al. (2016). Leading-order tail terms result from the backscattering of the leading-order outgoing radiation, the “tails-of-tails” from their second backscattering, and so on.

Through 1.5PN order, the luminosity expression (3.69) takes the form

3.76 $$\begin{aligned} {\mathcal {L}}(t) = \frac{G}{5c^5}\left\{ \text{ M }^{(3)}_{ij}\text{ M }^{(3)}_{ij} + \frac{1}{c^2}\left[ \frac{5}{189}\text{ M }^{(4)}_{ijk}\text{ M }^{(4)}_{ijk} + \frac{16}{9}\text{ S }^{(3)}_{ij}\text{ S }^{(3)}_{ij}\right] \right\} . \end{aligned}$$

On reasons of energy balance in asymptotically flat space, for any coordinates or variables representation of the Einstein theory, the time-averaged energy loss has to fulfill a relation of the form

3.77 $$\begin{aligned} -\left\langle \frac{\displaystyle \mathrm {d}{\mathcal {E}}\left( t-{r_*}/{c}\right) }{\mathrm {d}t}\right\rangle = \left\langle {\mathcal {L}}(t)\right\rangle , \end{aligned}$$

where the time averaging procedure takes into account typical periods of the system. Generalizing our considerations after Eq. (3.70) we may take the observation time *t* much larger than the time, say $t_{\mathrm{bfz}}$, the radiation enters the far or wave zone, even larger than the damping time of the radiating system, by just freely transporting the radiation power along the null cone with tacitly assuming $\langle {\mathcal{L}}(t)\rangle =\langle {\mathcal{L}}(t_{\mathrm{bfz}})\rangle$. Coming back to Eq. (3.70), time averaging on the left side of Eq. (3.70) eliminates total time derivatives of higher PN order, so-called Schott terms, and transforms them into much higher PN orders. The both sides of the equation (3.77) are gauge (or, coordinate) invariant. We stress that the Eq. (3.77) is valid for bound systems. In case of scattering processes, a coordinate invariant quantity is the emitted total energy.

The energy flux to *n*PN order in the far zone implies energy loss to $(n+5/2)$PN order in the near zone. Hereof it follows that energy-loss calculations are quite efficient via energy-flux calculations (Blanchet 2014). In general, only after averaging over orbital periods the both expressions do coincide. In the case of circular orbits, however, this averaging procedure is not needed.

## Applied regularization techniques

The most efficient source model for analytical computations of many-body dynamics in general relativity are point masses (or particles) represented through Dirac delta functions. If internal degrees of freedom are come into play, derivatives of the delta functions must be incorporated into the source. Clearly, point-particle sources in field theories introduce field singularities, which must be regularized in computations. Two aspects are important: (i) the differentiation of singular functions, and (ii) the integration of singular functions, either to new (usually also singular) functions or to the final Routhian/Hamiltonian. The item (ii) relates to the integration of the field equations and the item (i) to the differentiation of their (approximate) solutions. On consistency reasons, differentiation and integration must commute.

The most efficient strategy developed for computation of higher-order PN point-particle Hamiltonians relies on performing a 3-dimensional full computation in the beginning (using Riesz-implemented Hadamard regularization defined later in this section) and then correcting it by a *d*-dimensional one around the singular points, as well the local ones (UV divergences) as the one at infinity (IR divergences). A *d*-dimensional full computation is not needed. At higher than the 2PN level 3-dimensional computations with analytical Hadamard and Riesz regularizations show up ambiguities which require a more powerful treatment. The latter is dimensional regularization. The first time this strategy was successfully applied was in the 3PN dynamics of binary point particles (Damour et al. 2001); IR divergences did not appear therein, those enter from the 4PN level on only, the same as the nonlocal-in-time tail terms to which they are connected. At 4PN order, using different regularization methods for the treatment of IR divergences (Jaranowski and Schäfer 2015), an ambiguity parameter was left which, however, got fixed by matching to self-force calculations in the Schwarzschild metric (Le Tiec et al. 2012; Bini and Damour 2013; Damour et al. 2014).

The regularization techniques needed to perform PN calculations up to (and including) 4PN order, are described in detail in Appendix A of Jaranowski and Schäfer (2015).

### Distributional differentiation of homogeneous functions

Besides appearance of UV divergences, another consequence of employing Dirac-delta sources is necessity to differentiate homogeneous functions using an enhanced (or distributional) derivative, which comes from standard distribution theory (see, e.g., Sect. 3.3 in Chapter III of Gel’fand and Shilov 1964).

Let *f* be a real-valued function defined in a neighbourhood of the origin of ${\mathbb {R}}^3$. *f* is said to be a *positively homogeneous function of degree* $\lambda$, if for any number $a>0$

4.1 $$\begin{aligned} f(a\,\mathbf{x}) = a^\lambda \,f(\mathbf{x}). \end{aligned}$$

Let $k:=-\lambda -2$. If $\lambda$ is an integer and if $\lambda \le -2$ (i.e., *k* is a nonnegative integer), then the partial derivative of *f* with respect to the coordinate $x^i$ has to be calculated by means of the formula

4.2 $$\begin{aligned} \partial _i f(\mathbf{x}) = \partial _{{\underline{i}}}f(\mathbf{x}) + \frac{(-1)^k}{k!} \frac{\partial ^k\delta (\mathbf{x})}{\partial x^{i_1}\cdots \partial x^{i_k}} \times \oint _\varSigma \mathrm {d}\sigma _i\,f(\mathbf{x}')\,x'^{i_1}\cdots x'^{i_k}, \end{aligned}$$

where $\partial _i f$ on the lhs denotes the derivative of *f* considered as a distribution, while $\partial _{{\underline{i}}}f$ on the rhs denotes the derivative of *f* considered as a function (which is computed using the standard rules of differentiation), $\varSigma$ is any smooth close surface surrounding the origin and $\mathrm {d}\sigma _i$ is the surface element on $\varSigma$.

The distributional derivative does not obey the Leibniz rule. It can easily be seen by considering the distributional partial derivative of the product $1/r_a$ and $1/r_a^2$. Let us suppose that the Leibniz rule is applicable here:

4.3 $$\begin{aligned} \partial _i{\frac{1}{r_a^3}} = \partial _i{\left( \frac{1}{r_a}\frac{1}{r_a^2}\right) } = \frac{1}{r_a^2}\, \partial _i{\frac{1}{r_a}} + \frac{1}{r_a}\, \partial _i{\frac{1}{r_a^2}}. \end{aligned}$$

The right-hand side of this equation can be computed using standard differential calculus (no terms with Dirac deltas), whereas computing the left-hand side one obtains some term proportional to $\partial _i\delta _a$. The distributional differentiation is necessary when one differentiates homogeneous functions under the integral sign. For more details, see Appendix A5 in Jaranowski and Schäfer (2015).

### Riesz-implemented Hadamard regularization

The usage of Dirac $\delta$-functions to model point-mass sources of gravitational field leads to occurrence of UV divergences, i.e., the divergences near the particle locations ${\mathbf {x}}_a$, as $r_a\equiv |\mathbf {x}-{\mathbf {x}}_a|\rightarrow 0$. To deal with them, Infeld (1954, 1957), and Infeld and Plebański (1960) introduced “good” $\delta$-functions, which, besides having the properties of ordinary Dirac $\delta$-functions, also satisfy the condition

4.4 $$\begin{aligned} \frac{1}{|\mathbf{x} - \mathbf{x}_0|^k}\delta (\mathbf{x}-\mathbf{x}_0) = 0, \quad k = 1,\ldots ,p, \end{aligned}$$

for some positive integer *p* (in practical calculations one takes *p* large enough to take all singularities appearing in the calculation into account). They also assumed that the “tweedling of products” property is always satisfied

4.5 $$\begin{aligned} \int \mathrm {d}^3x\, f_1(\mathbf{x})f_2(\mathbf{x})\delta (\mathbf{x} - \mathbf{x}_0) = f_{1 {\mathrm{reg}}}(\mathbf{x}_0) f_{2 {\mathrm{reg}}}(\mathbf{x}_0), \end{aligned}$$

where “reg” means regularized value of the function at its singular point (i.e., $\mathbf{x}_0$ in the equation above) evaluated by means of the rule (4.4).

A natural generalization of the rule (4.4) is the concept of “partie finie” value of function at its singular point, defined as

4.6 $$\begin{aligned} f_{\mathrm{reg}}(\mathbf{x}_0) \equiv \frac{1}{4\pi } \int \mathrm {d}\varOmega \, a_0({\mathbf {n}}), \end{aligned}$$

with (here *M* is some non-negative integer)

4.7 $$\begin{aligned} f(\mathbf{x} = \mathbf{x}_0 + \epsilon {\mathbf {n}}) = \sum _{m=-M}^{\infty } a_m({\mathbf {n}})\epsilon ^m, \quad {\mathbf {n}}\equiv \frac{\mathbf{x}-\mathbf{x}_0}{|\mathbf{x}-\mathbf{x}_0|}. \end{aligned}$$

Defining, for a function *f* singular at $\mathbf {x}=\mathbf{x}_0$,

4.8 $$\begin{aligned} \int \mathrm {d}^3x f(\mathbf{x})\delta (\mathbf{x}-\mathbf{x}_0) \equiv f_{\mathrm{reg}}(\mathbf{x}_0), \end{aligned}$$

the “tweedling of products” property (4.5) can be written as

4.9 $$\begin{aligned} (f_1 f_2)_{\mathrm{reg}}(\mathbf{x}_0) = f_{1 {\mathrm{reg}}}(\mathbf{x}_0) f_{2{\mathrm{reg}}}(\mathbf{x}_0). \end{aligned}$$

The above property is generally wrong for arbitrary singular functions $f_1$ and $f_2$. In the PN calculations problems with fulfilling this property begin at the 3PN order. This is one of the reasons why one should use dimensional regularization.

The Riesz-implemented Hadamard (RH) regularization was developed in the context of deriving PN equations of motion of binary systems by Jaranowski and Schäfer (1997, 1998, 2000c) to deal with locally divergent integrals computed in three dimensions. The method is based on the Hadamard “partie finie” and the Riesz analytic continuation procedures.

The RH regularization relies on multiplying the full integrand, say $i(\mathbf{x})$, of the divergent integral by a regularization factor,

4.10 $$\begin{aligned} i(\mathbf{x}) \longrightarrow i(\mathbf{x})\Big (\frac{r_1}{s_1}\Big )^{\epsilon _1} \Big (\frac{r_2}{s_2}\Big )^{\epsilon _2}, \end{aligned}$$

and studying the double limit $\epsilon _1\rightarrow 0$, $\epsilon _2\rightarrow 0$ within analytic continuation in the complex $\epsilon _1$ and $\epsilon _2$ planes (here $s_1$ and $s_2$ are arbitrary three-dimensional UV regularization scales). Let us thus consider such integral performed over the whole space ${{\mathbb {R}}}^3$ and let us assume than it develops *only local poles* (so it is convergent at spatial infinity). The value of the integral, after performing the RH regularization in three dimensions, has the structure

4.11 $$\begin{aligned} I^{\text {RH}}&(3;\epsilon _1,\epsilon _2) \equiv \int _{{{\mathbb {R}}}^3} i({{\mathbf {x}}}) \Big (\frac{r_1}{s_1}\Big )^{\epsilon _1} \Big (\frac{r_2}{s_2}\Big )^{\epsilon _2}\,\mathrm {d}^3x \nonumber \\&= A + c_1 \Big (\frac{1}{\epsilon _1} + \ln \frac{r_{12}}{s_1} \Big ) + c_2 \Big (\frac{1}{\epsilon _2} + \ln \frac{r_{12}}{s_2} \Big ) + \mathcal {O}(\epsilon _1,\epsilon _2). \end{aligned}$$

Let us mention that in the PN calculations regularized integrands $i({{\mathbf {x}}})(r_1/s_1)^{\epsilon _1}(r_2/s_2)^{\epsilon _2}$ depend on $\mathbf {x}$ only through $\mathbf {x}-\mathbf {x}_1$ and $\mathbf {x}-\mathbf {x}_2$, so they are translationally invariant. This explains why the regularization result (4.11) depends on $\mathbf {x}_1$ and $\mathbf {x}_2$ only through $\mathbf {x}_1-\mathbf {x}_2$.

In the case of an integral over ${{\mathbb {R}}}^3$ developing poles *only at spatial infinity* (so it is locally integrable) it would be enough to use a regularization factor of the form $(r/r_0)^\epsilon$ (where $r_0$ is an IR regularization scale), but it is more convenient to use the factor

4.12 $$\begin{aligned} \Big (\frac{r_1}{r_0}\Big )^{a\epsilon } \Big (\frac{r_2}{r_0}\Big )^{b\epsilon } \end{aligned}$$

and study the limit $\epsilon \rightarrow 0$. Let us denote the integrand again by $i(\mathbf {x})$. The integral, after performing the RH regularization in three dimensions, has the structure

4.13 $$\begin{aligned} I^{\text {RH}}(3;a,b,\epsilon ) \equiv \int _{{{\mathbb {R}}}^3} i({{\mathbf {x}}}) \Big (\frac{r_1}{r_0}\Big )^{a\epsilon } \Big (\frac{r_2}{r_0}\Big )^{b\epsilon }\,\mathrm {d}^3x = A - c_\infty \bigg (\frac{1}{(a+b)\epsilon } + \ln \frac{r_{12}}{r_0} \bigg ) + \mathcal {O}(\epsilon ). \end{aligned}$$

Let us remark here that the extended Hadamard regularization procedure developed by Blanchet and Faye (2000a, b, 2001a, b) is both incompatible with standard distribution theory and with the method of dimensional regularization; for more details see Jaranowski and Schäfer (2015) (see also the footnote 2 above).

Many integrals appearing in PN calculations were computed using a famous formula derived in Riesz (1949) in *d* dimensions. It reads

4.14 $$\begin{aligned} \int \mathrm {d}^dx\, r^{\alpha }_1r^{\beta }_2 = \pi ^{d/2} \frac{\varGamma (\frac{\alpha +d}{2}) \varGamma (\frac{\beta +d}{2}) \varGamma (-\frac{\alpha +\beta +d}{2}) }{\varGamma (-\frac{\alpha }{2})\varGamma (-\frac{\beta }{2})\varGamma (\frac{\alpha +\beta +2d}{2})}r^{\alpha + \beta + d}_{12}. \end{aligned}$$

To compute the 4PN-accurate two-point-mass Hamiltonian one needs to employ a generalization of the three-dimensional version of this formula for integrands of the form $r^{\alpha }_1r^{\beta }_2(r_1+r_2+r_{12})^\gamma$. Such formula was derived by Jaranowski and Schäfer (1998, 2000c) and also there an efficient way of implementing both formulae to regularize divergent integrals was proposed (it employs prolate spheroidal coordinates in three dimensions). See Appendix A1 of Jaranowski and Schäfer (2015) for details and Appendix A of Hartung et al. (2013) for generalization of this procedure to *d* space dimensions.

### Dimensional regularization

It was first shown by Damour et al. (2001), that the unambiguous treatment of UV divergences in the current context requires usage of dimensional regularization (see, e.g., Collins 1984). It was used both in the Hamiltonian approach and in the one using the Einstein field equations in harmonic coordinates (Damour et al. 2001, 2014; Blanchet et al. 2004; Jaranowski and Schäfer 2013, 2015; Bernard et al. 2016). The dimensional regularization preserves the law of “tweedling of products” (4.9) and gives all involved integrals, particularly the inverse Laplacians, a unique definition.

#### *D*-dimensional ADM formalism

Dimensional regularization (DR) needs the representation of the Einstein field equation for arbitrary space dimensions, say *d* for the dimension of space and $D=d+1$ for the spacetime dimension. In the following, $G_D = G_{\mathrm{N}} \ell _0^{d-3}$ will denote the gravitational constant in *D*-dimensional spacetime and $G_{\mathrm{N}}$ the standard Newtonian one, $\ell _0$ is the DR scale relating both constants.

The unconstraint Hamiltonian takes the form

4.15 $$\begin{aligned} H = \int \mathrm {d}^dx\,(N {{\mathcal {H}}} - c N^i {{\mathcal {H}}}_i) + \frac{c^4}{16\pi G_D}\oint _{i^0}\mathrm {d}^{d-1}S_i\,\partial _j(\gamma _{ij}-\delta _{ij}\gamma _{kk}), \end{aligned}$$

where $\mathrm {d}^{d-1}S_i$ denotes the $(d-1)$-dimensional surface element. The Hamiltonian and the momentum constraint equations written for many-point-particle systems are given by

4.16 $$\begin{aligned} \sqrt{\gamma }\,R&= \frac{1}{\sqrt{\gamma }} \left( \gamma _{ik}\gamma _{j\ell }\pi ^{ij}\pi ^{k\ell } - \frac{1}{d-1}(\gamma _{ij}\pi ^{ij})^2 \right) \nonumber \\&\quad + \frac{16\pi G_D}{c^3}\sum _a (m_a^2c^4 + \gamma _a^{ij}p_{ai}p_{aj})^{\frac{1}{2}}\delta _a, \end{aligned}$$

4.17 $$\begin{aligned} -\nabla _j\pi ^{ij}&= \frac{8\pi G_D}{c^3}\sum _a \gamma _a^{ij}p_{aj}\delta _a. \end{aligned}$$

The gauge (or coordinate) ADMTT conditions read

4.18 $$\begin{aligned} \gamma _{ij} = \left( 1 + \frac{d-2}{4(d-1)}\phi \right) ^{4/(d-2)} \delta _{ij} + h^{\mathrm{TT}}_{ij}, \quad \pi ^{ii}=0, \end{aligned}$$

where

4.19 $$\begin{aligned} h^{\mathrm{TT}}_{ii} = 0, \quad \partial _j h^{\mathrm{TT}}_{ij} = 0. \end{aligned}$$

The field momentum $\pi ^{ij}$ splits into its longitudinal and TT parts, respectively,

4.20 $$\begin{aligned} \pi ^{ij} = {\tilde{\pi }}^{ij} + \pi _{\mathrm{TT}}^{ij}, \end{aligned}$$

where the longitudinal part ${\tilde{\pi }}^{ij}$ can be expressed in terms of a vectorial function $V^i$,

4.21 $$\begin{aligned} {\tilde{\pi }}^{ij} = \partial _i V^j +\partial _j V^i - \frac{2}{d}\delta ^{ij}\partial _k V^k, \end{aligned}$$

and where the TT part satisfies the conditions,

4.22 $$\begin{aligned} \pi _{\mathrm{TT}}^{ii} = 0, \quad \partial _j \pi _{\mathrm{TT}}^{ij} = 0. \end{aligned}$$

The reduced Hamiltonian of the particles-plus-field system takes the form

4.23 $$\begin{aligned} H_\text {red}\big [{\mathbf {x}}_a,{\mathbf {p}}_a,h^{\mathrm{TT}}_{ij},\pi _{\mathrm{TT}}^{ij}\big ] = -\frac{c^4}{16\pi G_D}\int \mathrm {d}^dx\,\varDelta \phi \big [{\mathbf {x}}_a,{\mathbf {p}}_a,h^{\mathrm{TT}}_{ij},\pi _{\mathrm{TT}}^{ij}\big ]. \end{aligned}$$

The equations of motion for the particles read

4.24 $$\begin{aligned} \dot{\mathbf {x}}_a = \frac{\partial H_\text {red}}{\partial {{\mathbf {p}}_a}}, \quad \dot{\mathbf {p}}_a = -\frac{\partial H_\text {red}}{\partial {{\mathbf {x}}_a}}, \end{aligned}$$

and the field equations for the independent degrees of freedom are given by

4.25 $$\begin{aligned} \frac{\partial }{\partial t}h^{\mathrm{TT}}_{ij} = \frac{16\pi G_D}{c^3}\,\delta ^{\text {TT}kl}_{ij}\frac{\delta H_\text {red}}{\delta \pi _{\mathrm{TT}}^{kl}}, \qquad \frac{\partial }{\partial t}\pi _{\mathrm{TT}}^{ij} = -\frac{16\pi G_D}{c^3}\,\delta ^{\text {TT}ij}_{kl}\frac{\delta H_\text {red}}{\delta h^{\mathrm{TT}}_{kl}}, \end{aligned}$$

where the *d*-dimensional TT-projection operator is defined by

4.26 $$\begin{aligned} \delta ^{\text {TT}ij}_{kl}&\equiv \frac{1}{2}(\delta _{ik}\delta _{jl}+\delta _{il}\delta _{jk}) - \frac{1}{d-1}\delta _{ij}\delta _{kl} \nonumber \\&\quad - \frac{1}{2}(\delta _{ik}\partial _{jl}+\delta _{jl}\partial _{ik}+\delta _{il}\partial _{jk}+\delta _{jk}\partial _{il})\varDelta ^{-1} \nonumber \\&\quad + \frac{1}{d-1}(\delta _{ij}\partial _{kl}+\delta _{kl}\partial _{ij})\varDelta ^{-1} + \frac{d-2}{d-1}\partial _{ijkl}\varDelta ^{-2}. \end{aligned}$$

Finally, the Routh functional is defined as

4.27 $$\begin{aligned} R\big [{\mathbf {x}}_a,{\mathbf {p}}_a,h^{\mathrm{TT}}_{ij},\dot{h}^{\mathrm{TT}}_{ij}\big ] \equiv H_\text {red}\big [{\mathbf {x}}_a,{\mathbf {p}}_a, h^{\mathrm{TT}}_{ij}, \pi _{\mathrm{TT}}^{ij}\big ] -\frac{c^3}{16\pi G_D}\int \mathrm {d}^dx\,\pi _{\mathrm{TT}}^{ij}\dot{h}^{\mathrm{TT}}_{ij}, \end{aligned}$$

and the fully reduced matter Hamiltonian for the conservative dynamics reads

4.28 $$\begin{aligned} H[{\mathbf {x}}_a,{\mathbf {p}}_a] \equiv R\big [{\mathbf {x}}_a,{\mathbf {p}}_a,h^{\mathrm{TT}}_{ij}({\mathbf {x}}_a,{\mathbf {p}}_a),\dot{h}^{\mathrm{TT}}_{ij}({\mathbf {x}}_a,{\mathbf {p}}_a)\big ]. \end{aligned}$$

#### Local and asymptotic dimensional regularization

The technique developed by Damour et al. (2001) to control local (or UV) divergences boils down to the computation of the difference

4.29 $$\begin{aligned} \lim _{d\rightarrow 3} H^\text {loc}(d) - H^\text {RH loc}(3), \end{aligned}$$

where $H^\text {RH loc}(3)$ is the “local part” of the Hamiltonian obtained by means of the three-dimensional RH regularization [it is the sum of all integrals of the type $I^{\text {RH}}(3;\epsilon _1,\epsilon _2)$ introduced in Eq. (4.11)], $H^\text {loc}(d)$ is its *d*-dimensional counterpart.

Damour et al. (2001) showed that to find the DR correction to the integral $I^{\text {RH}}(3;\epsilon _1,\epsilon _2)$ of Eq. (4.11) related with the local pole at, say, $\mathbf {x}=\mathbf {x}_1$, it is enough to consider only this part of the integrand $i(\mathbf{x})$ which develops logarithmic singularities in three dimensions, i.e., which locally behaves like $1/r_1^3$,

4.30 $$\begin{aligned} i(\mathbf {x}) = \cdots + \tilde{c}_1(\mathbf {n}_1)\,r_1^{-3} + \cdots , \quad \text {when}\ \mathbf {x}\rightarrow \mathbf {x}_1. \end{aligned}$$

Then the pole part of the integral (4.11) related with the singularity at $\mathbf {x}=\mathbf {x}_1$ can be recovered by RH regularization of the integral of $\tilde{c}_1(\mathbf {n}_1)\,r_1^{-3}$ over the ball ${\mathbb {B}}(\mathbf {x}_1,{\ell _1})$ of radius $\ell _1$ surrounding the particle $\mathbf {x}_1$. The RH regularized value of this integral reads

4.31 $$\begin{aligned} I_1^{\text {RH}}(3;\epsilon _1) \equiv \int _{{\mathbb {B}}(\mathbf {x}_1,{\ell _1})} \tilde{c}_1(\mathbf {n}_1) \, r_1^{-3} \Big (\frac{r_1}{s_1}\Big )^{\epsilon _1} \, \mathrm {d}^3 {{\mathbf {r}}}_1 = c_1 \int _0^{\ell _1} r_1^{-1} \Big (\frac{r_1}{s_1}\Big )^{\epsilon _1}\,\mathrm {d}r_1, \end{aligned}$$

where $c_1/(4\pi )$ is the angle-averaged value of the coefficient $\tilde{c}_1(\mathbf {n}_1)$. The expansion of the integral $I_1^{\text {RH}}(3;\epsilon _1)$ around $\epsilon _1=0$ equals

4.32 $$\begin{aligned} I_1^{\text {RH}}(3;\epsilon _1) = c_1\Big (\frac{1}{\epsilon _1} + \ln \frac{\ell _1}{s_1}\Big ) + \mathcal {O}(\epsilon _1). \end{aligned}$$

The idea of the technique developed by Damour et al. (2001) relies on replacing the RH-regularized value of the three-dimensional integral $I_1^{\text {RH}}(3;\epsilon _1)$ by the value of its *d*-dimensional version $I_1(d)$. One thus considers the *d*-dimensional counterpart of the expansion (4.30). It reads

4.33 $$\begin{aligned} i(\mathbf {x}) = \cdots + \ell _0^{k(d-3)}\tilde{\mathfrak {c}}_1(d;{{\mathbf {n}}}_1)\,r_1^{6-3d} + \cdots , \quad \text {when}\ \mathbf {x}\rightarrow \mathbf {x}_1. \end{aligned}$$

Let us note that the specific exponent $6-3d$ of $r_1$ visible here follows from the $r_1\rightarrow 0$ behaviour of the (perturbative) solutions of the *d*-dimensional constraint equations (4.16)–(4.17). The number *k* in the exponent of $\ell _0^{k(d-3)}$ is related with the momentum-order of the considered term [e.g., at the 4PN level the term with *k* is of the order of $\mathcal {O}(p^{10-2k})$, for $k=1,\ldots ,5$; such term is proportional to $G_D^k$]. The integral $I_1(d)$ is defined as

4.34 $$\begin{aligned} I_1(d) \equiv \ell _0^{k(d-3)} \int _{{\mathbb {B}}(\mathbf {x}_1,{\ell _1})} \tilde{\mathfrak {c}}_1(d;{{\mathbf {n}}}_1) \, r_1^{6-3d}\, \mathrm {d}^d {{\mathbf {r}}}_1 = \mathfrak {c}_1(d) \int _0^{\ell _1} r_1^{5-2d}\,\mathrm {d}r_1, \end{aligned}$$

where ${\mathfrak {c}}_1(d)/\big (\varOmega _{d-1}\ell _0^{k(d-3)}\big )$ ($\varOmega _{d-1}$ stands for the area of the unit sphere in ${\mathbb {R}}^d$) is the angle-averaged value of the coefficient $\tilde{{\mathfrak {c}}}_1(d;{{\mathbf {n}}}_1)$,

4.35 $$\begin{aligned} {\mathfrak {c}}_1(d) \equiv \ell _0^{k(d-3)} \oint _{{\mathbb {S}}^{d-1}(\mathbf {0},1)} \tilde{{\mathfrak {c}}}_1(d;{{\mathbf {n}}}_1)\,\mathrm {d}\varOmega _{d-1}. \end{aligned}$$

One checks that always there is a smooth connection between ${\mathfrak {c}}_1(d)$ and its three-dimensional counterpart $c_1$,

4.36 $$\begin{aligned} \lim _{d\rightarrow 3} {\mathfrak {c}}_1(d) = {\mathfrak {c}}_1(3) = c_1. \end{aligned}$$

The radial integral in Eq. (4.34) is convergent if the real part $\mathfrak {R}(d)$ of *d* fulfills the condition $\mathfrak {R}(d)<3$. Making use of the expansion ${\mathfrak {c}}_1(d) = {\mathfrak {c}}_1(3+\varepsilon ) = c_1 + {\mathfrak {c}}'_1(3)\varepsilon + \mathcal {O}(\varepsilon ^2)$, where $\varepsilon \equiv d-3$, the expansion of the integral $I_1(d)$ around $\varepsilon =0$ reads

4.37 $$\begin{aligned} I_1(d) = -\frac{\ell _1^{-2\varepsilon }}{2\varepsilon }{\mathfrak {c}}_1(3+\varepsilon ) = -\frac{c_1}{2\varepsilon } -\frac{1}{2} {\mathfrak {c}}'_1(3) + c_1 \ln \ell _1 + \mathcal {O}(\varepsilon ). \end{aligned}$$

Let us note that the coefficient ${\mathfrak {c}}'_1(3)$ usually depends on $\ln r_{12}$ and it has the structure

4.38 $$\begin{aligned} {\mathfrak {c}}'_1(3) = {\mathfrak {c}}'_{11}(3) + {\mathfrak {c}}'_{12}(3) \ln \frac{r_{12}}{\ell _0} + 2c_1 \ln \ell _0, \end{aligned}$$

where ${\mathfrak {c}}'_{12}(3)=(2 - k)c_1$ [what can be inferred knowing the dependence of ${\mathfrak {c}}_1(d)$ on $\ell _0$ given in Eq. (4.35)]. Therefore the DR correction also changes the terms $\propto \ln {r_{12}}$.

The DR correction to the RH-regularized value of the integral $I^{\text {RH}}(3;\epsilon _1,\epsilon _2)$ relies on replacing this integral by

4.39 $$\begin{aligned} I^{\text {RH}}(3;\epsilon _1,\epsilon _2) + \varDelta I_1 + \varDelta I_2, \end{aligned}$$

where

4.40 $$\begin{aligned} \varDelta I_a \equiv I_a(d) - I_a^{\text {RH}}(3;\epsilon _a), \quad a=1,2. \end{aligned}$$

Then one computes the double limit

4.41 $$\begin{aligned} \lim _{\begin{array}{c} \epsilon _1\rightarrow 0 \\ \epsilon _2\rightarrow 0 \end{array}} \Big (I^{\text {RH}}&(3;\epsilon _1,\epsilon _2) + \varDelta I_1 + \varDelta I_2\Big ) \nonumber \\&= A - \frac{1}{2} \big ({\mathfrak {c}}'_{11}(3) + {\mathfrak {c}}'_{21}(3)\big ) - \frac{1}{2}\big ({\mathfrak {c}}'_{12}(3) + {\mathfrak {c}}'_{22}(3)\big ) \ln \frac{r_{12}}{\ell _0} \nonumber \\&\quad + \big (c_1 + c_2\big )\left( - \frac{1}{2\varepsilon } + \ln \frac{r_{12}}{\ell _0}\right) + \mathcal {O}(\varepsilon ). \end{aligned}$$

Note that all poles $\propto 1/\epsilon _1,1/\epsilon _2$ and all terms depending on radii $\ell _1$, $\ell _2$ or scales $s_1$, $s_2$ cancel each other. The result (4.41) is as if all computations were fully done in *d* dimensions.

In the DR correcting UV of divergences in the 3PN two-point-mass Hamiltonian performed by Damour et al. (2001), after collecting all terms of the type (4.41) together, all poles $\propto 1/(d-3)$ cancel each other. This is not the case for the UV divergences of the 4PN two-point-mass Hamiltonian derived by Jaranowski and Schäfer (2015). As explained in Sect. VIII D of Jaranowski and Schäfer (2015), after collecting all terms of the type (4.41), one has to add to the Hamiltonian a *unique* total time derivative to eliminate all poles $\propto 1/(d-3)$ (together with $\ell _0$-dependent logarithms).

The above described technique of the DR correcting of UV divergences can easily be transcribed to control IR divergences. This is done by the replacement of the integrals

4.42 $$\begin{aligned} \int _{{\mathbb {B}}(\mathbf {x}_a,{\ell _a})}\mathrm {d}^dx\,i(\mathbf{x}) \end{aligned}$$

by the integral

4.43 $$\begin{aligned} \int _{{\mathbb {R}}^d\setminus {\mathbb {B}}(\mathbf {0},R)}\mathrm {d}^dx\,i(\mathbf{x}), \end{aligned}$$

where ${\mathbb {B}}(\mathbf {0},R)$ means a large ball of radius *R* (with the centre at the origin $\mathbf {0}$ of the coordinate system), and by studying expansion of the integrand $i(\mathbf{x})$ for $r\rightarrow \infty$. This technique was not used to regularize IR divergences in the computation of the 4PN two-point-mass Hamiltonian by Damour et al. (2014) and Jaranowski and Schäfer (2015). This was so because this technique applied only to the instantaneous part of the 4PN Hamiltonian is not enough to get rid of the IR poles in the limit $d\rightarrow 3$. For resolving IR poles it was necessary to observe that the IR poles have to cancel with the UV poles from the tail part of the Hamiltonian (what can be achieved e.g. after implementing the so-called zero-bin subtraction in the EFT framework, see Porto and Rothstein 2017).

Another two different approaches were employed by Damour et al. (2014) and Jaranowski and Schäfer (2015) to regularize IR divergences in the instantaneous part of the 4PN Hamiltonian (see Appendix A3 in Jaranowski and Schäfer 2015): (i) modifying the behavior of the function $h^\mathrm {TT}_{(6)ij}$ at infinity,^4^ (ii) implementing a *d*-dimensional version of Riesz–Hadamard regularization. Both approaches were developed in *d* dimensions, but the final results of using any of them in the limit $d\rightarrow 3$ turned out to be identical with the results of computations performed in $d=3$ dimensions. Moreover, the results of the two approaches were different in the limit $d\rightarrow 3$, what indicated the ambiguity of IR regularization, discussed in detail by Jaranowski and Schäfer (2015) and fixed by Damour et al. (2014). This IR ambiguity can be expressed in terms of only one unknown parameter, because the results of two regularization approaches, albeit different, have exactly the same structure with only different numerical prefactors. This prefactor can be treated as the ambiguity parameter. The full 4PN Hamiltonian was thus computed up to a single ambiguity parameter and it was used to calculate, in a gauge invariant form, the energy of two-body system along circular orbits as a function of frequency. The ambiguity parameter was fixed by comparison of part of this formula [linear in the symmetric mass ratio $\nu$, see Eq. (6.3) below for the definition] with the analogous 4PN-accurate formula for the particle in the Schwarzschild metric which included self-force corrections.

Analogous ambiguity was discovered in 4PN-accurate calculations of two-body equations of motion done by Bernard et al. (2016) in harmonic coordinates, where also analytic regularization of the IR divergences of the instantaneous part of the dynamics was performed. However, the computations made by Bernard et al. (2016) faced also a second ambiguity (Damour et al. 2016; Bernard et al. 2017b), which must come from their different (harmonic instead of ADMTT) gauge condition and the potentiality of analytic regularization not to preserve gauge (in contrast to dimensional regularization). The first method of analytic regularization applied by Damour et al. (2014) and Jaranowski and Schäfer (2015) is manifest ADMTT gauge preserving. Finally, Marchand et al. (2018) and Bernard et al. (2017a) successfully applied in harmonic-coordinates approach *d*-dimensional regularization all-over.

#### Distributional differentiation in *d* dimensions

One can show that the formula (4.2) for distributional differentiation of homogeneous functions is also valid (without any change) in the *d*-dimensional case. It leads, e.g., to equality

4.44 $$\begin{aligned} \partial _i \partial _j r^{2-d} = (d-2)\frac{d\,n^i n^j-\delta _{ij}}{r^d} - \frac{4\pi ^{d/2}}{d\,\varGamma (d/2 -1)}\delta _{ij}\delta . \end{aligned}$$

To overcome the necessity of using distributional differentiations it is possible to replace Dirac $\delta$-function by the class of analytic functions introduced in Riesz (1949),

4.45 $$\begin{aligned} \delta _{\epsilon }(\mathbf{x}) \equiv \frac{\varGamma ((d-\epsilon )/2)}{\pi ^{d/2}2^{\epsilon }\varGamma (\epsilon /2)}r^{\epsilon - d}, \end{aligned}$$

resulting in the Dirac $\delta$-function in the limit

4.46 $$\begin{aligned} \delta = \lim _{\epsilon \rightarrow 0}\delta _{\epsilon }. \end{aligned}$$

On this class of functions, the inverse Laplacian operates as

4.47 $$\begin{aligned} \varDelta ^{-1} \delta _{\epsilon } = -\delta _{\epsilon + 2}, \end{aligned}$$

and instead of (4.44) one gets

4.48 $$\begin{aligned} \partial _i \partial _j r^{\epsilon + 2 - d} = (d-2-\epsilon )\frac{(d-\epsilon )n^i n^j-\delta _{ij}}{r^{d-\epsilon }}. \end{aligned}$$

There is no need to use distributional differentiation here, so no $\delta$-functions are involved.

Though the replacements in the stress–energy tensor density of $\delta _a$ through $\delta _{\epsilon _a}$ (with $a=1,2$) do destroy the divergence freeness of the stress–energy tensor and thus the integrability conditions of the Einstein theory, the relaxed Einstein field equations (the ones which result after imposing coordinate conditions) do not force the stress–energy tensor to be divergence free and can thus be solved without problems. The solutions one gets do not fulfill the complete Einstein field equations but in the final limits $\epsilon _a\rightarrow 0$ the general coordinate covariance of the theory is manifestly recovered. This property, however, only holds if these limits are taken *before* the limit $d=3$ is performed (Damour et al. 2008a).

## Point-mass representations of spinless black holes

This section is devoted to an insight of how black holes, the most compact objects in GR, can be represented by point masses. On the other side, the developments in the present section show that point masses, interpreted as fictitious point masses (analogously to image charges in the electrostatics), allow to represent black holes. Later on, in the section on approximate Hamiltonians for spinning binaries, neutron stars will also be considered, taking into account their different rotational deformation. Tidal deformation will not be considered in this review; for information about this topic the reader is referred to, e.g., Damour and Nagar (2010) and Steinhoff et al. (2016).

The simplest black hole is a Schwarzschildian one which is isolated and non-rotating. Its metric is a static solution of the *vacuum* Einstein field equations. In isotropic coordinates, the Schwarzschild metric reads (see, e.g., Misner et al. 1973)

5.1 $$\begin{aligned} \mathrm {d}s^2 = -\left( \frac{\displaystyle 1-\frac{GM}{2rc^2}}{\displaystyle 1+\frac{GM}{2rc^2}}\right) ^2 c^2 \mathrm {d}t^2 + \left( 1+\frac{GM}{2rc^2}\right) ^4 \mathrm {d}\mathbf{x}^2, \end{aligned}$$

where *M* is the gravitating mass of the black hole and $(x^1,x^2,x^3)$ are Cartesian coordinates in ${\mathbb {R}}^3$ with $r^2 = (x^1)^2 + (x^2)^2 + (x^3)^2$ and $\mathrm {d}\mathbf{x}^2 = (\mathrm {d}x^1)^2 + (\mathrm {d}x^2)^2 + (\mathrm {d}x^3)^2$. The origin of the coordinate system $r=0$ is not located where the Schwarzschild singularity $R=0$, with *R* the radial Schwarzschild coordinate, is located, rather it is located on the other side of the Einstein–Rosen bridge, at infinity, where space is flat. The point $r=0$ does not belong to the three-dimensional spacelike curved manifold, so we do have an open manifold at $r=0$, a so-called “puncture” manifold (see, e.g., Brandt and Brügmann 1997; Cook 2005). However, as we shall see below, the Schwarzschild metric can be contructed with the aid of a Dirac $\delta$ function with support at $r=0$, located in a conformally related flat space of dimension smaller than three. Distributional sources with support at the Schwarzschild singularity are summarized and treated by Pantoja and Rago (2002) and Heinzle and Steinbauer (2002).

A two black hole initial value solution of the vacuum Einstein field equations is the time-symmetric Brill–Lindquist one (Brill and Lindquist 1963; Lindquist 1963),

5.2 $$\begin{aligned} \mathrm {d}s^2 = -\left( \frac{\displaystyle 1-\frac{\beta _1G}{2r_1c^2}-\frac{\beta _2G}{2r_2c^2}}{\displaystyle 1+\frac{\alpha _1G}{2r_1c^2}+\frac{\alpha _2G}{2r_2c^2}}\right) ^2 c^2\mathrm {d}t^2 + \left( 1+\frac{\alpha _1G}{2r_1c^2}+\frac{\alpha _2G}{2r_2c^2}\right) ^4\mathrm {d}\mathbf{x}^2, \end{aligned}$$

where $\mathbf{r}_a\equiv \mathbf{x}-\mathbf{x}_a$ and $r_a\equiv |{\mathbf {r}}_a|$ ($a=1,2$), the coefficients $\alpha _a$ and $\beta _a$ can be found in Jaranowski and Schäfer (2002) (notice that $h^\mathrm {TT}_{ij}=0$, $\pi ^{ij}=0$, and, initially, $\partial _t r_a=0$). Its total energy results from the ADM surface integral [this is the reduced ADM Hamiltonian from Eq. (2.20) written for the metric (5.2)]

5.3 $$\begin{aligned} E_{\text {ADM}} = -\frac{c^4}{2\pi G} \oint _{i_0}\mathrm {d}S_i\, \partial _i\varPsi = (\alpha _1 + \alpha _2)c^2, \end{aligned}$$

where $\mathrm {d}S_i=n^ir^2\mathrm {d}\varOmega$ is a two-dimensional surface-area element (with unit radial vector $n^i\equiv {x^i/r}$ and solid angle element $\mathrm {d}\varOmega$) and

5.4 $$\begin{aligned} \varPsi \equiv 1 + \frac{\alpha _1G}{2r_1c^2} + \frac{\alpha _2G}{2r_2c^2}. \end{aligned}$$

Introducing the inversion map $\mathbf {x}\rightarrow \mathbf {x}'$ defined by Brill and Lindquist (1963)

5.5 $$\begin{aligned} \mathbf{r}'_1 \equiv \mathbf{r}_1 \frac{\alpha _1^2G^2}{4c^4r_1^2} \quad \Longrightarrow \quad \mathbf{r}_1 = \mathbf{r}'_1 \frac{\alpha _1^2G^2}{4c^4r_1'^{2}}, \end{aligned}$$

where $\mathbf{r}'_1\equiv \mathbf{x}'-\mathbf{x}_1$, $r'_1\equiv |\mathbf{x}'-\mathbf{x}_1|$, the three-metric $\mathrm {d}l^2 = \varPsi ^4 \mathrm {d}\mathbf{x}^2$ transforms into

5.6 $$\begin{aligned} \mathrm {d}l^2 = \varPsi '^4 \mathrm {d}\mathbf{x}'^2, \quad \text {with}\quad \varPsi ' \equiv 1+\frac{\alpha _1G}{2r'_1c^2}+\frac{\alpha _1\alpha _2G^2}{4r_2 r'_1 c^4}, \end{aligned}$$

where $\mathbf{r}_2 = \mathbf{r}'_1 \alpha _1^2G^2/(4c^4r_1'^{2}) + \mathbf{r}_{12}$ with $\mathbf{r}_{12}\equiv \mathbf{x}_{1} - \mathbf{x}_{2}$. From the new metric function $\varPsi '$ the proper mass of the throat 1 results in,

5.7 $$\begin{aligned} m_1 \equiv -\frac{c^2}{2\pi G}\oint _{i_0^1} \mathrm {d}S'_i\, \partial '_i \varPsi ' = \alpha _1 + \frac{\alpha _1\alpha _2G}{2r_{12}c^2}, \end{aligned}$$

where $i_0^1$ denotes the black hole’s 1 own spacelike infinity. Hereof the ADM energy comes out in the form,

5.8 $$\begin{aligned} E_{\text {ADM}} = (m_1 + m_2)c^2 - G \frac{\alpha _1\alpha _2}{r_{12}}, \end{aligned}$$

where

5.9 $$\begin{aligned} \alpha _a = \frac{m_a-m_b}{2} + \frac{c^2r_{ab}}{G}\left( \sqrt{1+\frac{m_a+m_b}{c^2r_{ab}/G} + \left( \frac{m_a-m_b}{2c^2r_{ab}/G}\right) ^2} - 1 \right) . \end{aligned}$$

This construction, as performed by Brill and Lindquist (1963), is a purely geometrical (or vacuum) one without touching singularities. Recall that this energy belongs to an initial value solution of the Einstein constraint equations with vanishing of both $h^{\mathrm{TT}}_{ij}$ and particle together with field momenta. In this initial conditions spurious gravitational waves are included.

In the following we will show how the vacuum Brill–Lindquist solution can be obtained with Dirac $\delta$-function source terms located at $r_1=0$ and $r_2=0$ in a conformally related three-dimensional flat space. To do this we will formulate the problem in *d* space dimensions and make analytical continuation in *d* of the results down to $d=3$. The insertion of the stress–energy density for point masses into the Hamiltonian constraint equation yields, for $p_{ai}=0$, $h^{\mathrm{TT}}_{ij}=0$, and $\pi ^{ij}=0$,

5.10 $$\begin{aligned} -\varPsi \varDelta \phi = \frac{16\pi G}{c^2} \sum _a m_a \delta _a, \end{aligned}$$

where $\varPsi$ and $\phi$ parametrize the space metric,

5.11 $$\begin{aligned} \gamma _{ij} = \varPsi ^{4/(d-2)}\delta _{ij}, \quad \varPsi \equiv 1 + \frac{d-2}{4(d-1)}\phi . \end{aligned}$$

If the lapse function *N* is represented by

5.12 $$\begin{aligned} N \equiv \frac{\chi }{\varPsi }, \end{aligned}$$

an equation for $\chi$ results of the form (using the initial-data conditions $p_{ai}=0$, $h^{\mathrm{TT}}_{ij}=0$, $\pi ^{ij}=0$),

5.13 $$\begin{aligned} \varPsi ^2 \varDelta \chi =\frac{4\pi G}{c^2} \frac{d-2}{d-1}\chi \sum _a m_a\delta _a. \end{aligned}$$

With the aid of the relation

5.14 $$\begin{aligned} \varDelta \frac{1}{r_a^{d-2}} = -\frac{4\pi ^{d/2}}{\varGamma (d/2-1)}\delta _a \end{aligned}$$

it is easy to show that for $1<d<2$ the equations for $\varPsi$ and $\chi$ do have well-defined solutions. To obtain these solutions we employ the ansatz

5.15 $$\begin{aligned} \phi = \frac{4G}{c^2}\frac{\varGamma (d/2-1)}{\pi ^{d/2-1}}\left( \frac{\alpha _1}{r^{d-2}_1}+\frac{\alpha _2}{r^{d-2}_2}\right) , \end{aligned}$$

where $\alpha _1$ and $\alpha _2$ are some constants. After plugging the ansatz (5.15) into Eq. (5.10) we compare the coefficients of the Dirac $\delta$-functions on both sides of the equation. For point mass 1 we get

5.16 $$\begin{aligned} \bigg (1 + \frac{G(d-2)\varGamma (d/2-1)}{c^2(d-1)\pi ^{d/2-1}} \Big (\frac{\alpha _1}{r^{d-2}_1}+\frac{\alpha _2}{r^{d-2}_2}\Big )\bigg ) \alpha _1 \delta _1 = m_1 \delta _1. \end{aligned}$$

After taking $1<d<2$, one can perform the limit $r_1 \rightarrow 0$ for the coefficient of $\delta _1$ in the left-hand-side of the above equation,

5.17 $$\begin{aligned} \bigg (1 + \frac{G(d-2)\varGamma (d/2-1)}{c^2(d-1)\pi ^{d/2-1}} \frac{\alpha _2}{r^{d-2}_{12}}\bigg ) \alpha _1 \delta _1 = m_1 \delta _1. \end{aligned}$$

Going over to $d=3$ by arguing that the solution is analytic in *d* results in the relation

5.18 $$\begin{aligned} \alpha _a = \frac{m_a}{\displaystyle 1+ \frac{G}{2c^2}\frac{\alpha _b}{r_{ab}}}, \end{aligned}$$

where $b \ne a$ and $a,b = 1,2$. The ADM energy is again given by, in the limit $d=3$,

5.19 $$\begin{aligned} E_{\text {ADM}} = (\alpha _1 + \alpha _2) c^2. \end{aligned}$$

Here we recognize the important aspect that although the metric may describe close binary black holes with strongly deformed apparent horizons, the both black holes can still be generated by point masses in conformally related flat space. This is the justification for our particle model to be taken as model for orbiting black holes. Obviously black holes generated by point masses are orbiting black holes without spin, i.e., Schwarzschild-type black holes. The representation of a Schwarzschild-type black hole in binary-black-hole systems with one Dirac $\delta$-function seems not to be the only possibility. As shown by Jaranowski and Schäfer (2000a), binary-black-hole configurations defined through isometry-conditions at the apparent horizons (Misner 1963) need infinitely many Dirac $\delta$-functions per each one of the black holes. Whether or not those black holes are more physical is not known. It has been found by Jaranowski and Schäfer (1999) that the expressions for ADM energy of the two kinds of binary black holes do agree through 2PN order, and that at the 3PN level the energy of the Brill–Lindquist binary black holes is additively higher by $G^4m_1^2m_2^2(m_1+m_2)/(8c^6r^4_{12})$, i.e. the Misner configuration seems stronger bound. The same paper has shown that the spatial metrics of both binary-black-hole configurations coincide through 3PN order, and that at least through 5PN order they can be made to coincide by shifts of black-hole position variables.

## Post-Newtonian Hamilton dynamics of nonspinning compact binaries

In this section we collect explicit results on Hamilton dynamics of binaries made of compact and nonspinning bodies. Up to the 4PN order the Hamiltonian of binary point-mass systems is explicitly known and it can be written as the sum

6.1 $$\begin{aligned} H[{\mathbf {x}}_a,{\mathbf {p}}_a,t]&= \sum _a m_a c^2 + H_{\text {N}}({\mathbf {x}}_a,{\mathbf {p}}_a) + \frac{1}{c^2} H_{\text {1PN}}({\mathbf {x}}_a,{\mathbf {p}}_a) + \frac{1}{c^4} H_{\text {2PN}}({\mathbf {x}}_a,{\mathbf {p}}_a) \nonumber \\&\quad + \frac{1}{c^5} H_{\text {2.5N}}({\mathbf {x}}_a,{\mathbf {p}}_a,t) + \frac{1}{c^6} H_{\text {3PN}}({\mathbf {x}}_a,{\mathbf {p}}_a) + \frac{1}{c^7} H_{\text {3.5PN}}({\mathbf {x}}_a,{\mathbf {p}}_a,t) \nonumber \\&\quad + \frac{1}{c^8} H_{\text {4PN}}[{\mathbf {x}}_a,{\mathbf {p}}_a] + \mathcal {O}(c^{-9}). \end{aligned}$$

This Hamiltonian is the PN-expanded reduced ADM Hamiltonian of point-masses plus field system; the nontrivial procedure of reduction is described in Sects. 3.1 and 3.2 of this review. The non-autonomous dissipative Hamiltonians $H_{\text {2.5PN}}({\mathbf {x}}_a,{\mathbf {p}}_a,t)$ and $H_{\text {3.5PN}}({\mathbf {x}}_a,{\mathbf {p}}_a,t)$ are written as explicitly depending on time because they depend on the gravitational field variables (see Sect. 6.5 for more details). The dependence of the 4PN Hamiltonian $H_{\text {4PN}}$ on ${\mathbf {x}}_a$ and ${\mathbf {p}}_a$ is both pointwise and functional (and this is why we have used square brackets for arguments of $H_{\text {4PN}}$).

We will display here the conservative Hamiltonians $H_{\text {N}}$ to $H_{\text {4PN}}$ in the centre-of-mass reference frame, relegating their generic, noncentre-of-mass forms, to Appendix C. In the ADM formalism the centre-of-mass reference frame is defined by the simple requirement

6.2 $$\begin{aligned} \mathbf {p}_1 + \mathbf {p}_2 = \mathbf {0}. \end{aligned}$$

Here we should point out that at the 3.5PN order for the first time recoil arises, hence the conservation of linear momentum is violated [see, e.g., Fitchett 1983 (derivation based on wave solutions of linearized field equations) and Junker and Schäfer 1992 (derivation based on wave solutions of non-linear field equations)]. This however has no influence on the energy through 6.5PN order, if $\mathbf {P}\equiv \mathbf {p}_1+\mathbf {p}_2=\mathbf {0}$ holds initially, because up to 3PN order the Eq. (3.43) is valid and the change of the Hamiltonian *H* caused by nonconservation of $\mathbf {P}$ equals $\mathrm {d}H/\mathrm {d}t=[(c^2/H)\mathbf {P}]_{\mathrm{3PN}}\cdot (\mathrm {d}\mathbf {P}/\mathrm {d}t)_{\mathrm{3.5PN}}=0$ through 6.5PN order.

Let us define

6.3 $$\begin{aligned} M \equiv m_1 + m_2, \quad \mu \equiv \frac{m_1m_2}{M}, \quad \nu \equiv \frac{\mu }{M}, \end{aligned}$$

where the symmetric mass ratio $0 \le \nu \le 1/4$, with $\nu = 0$ being the test-body case and $\nu = 1/4$ for equal-mass binaries. It is convenient to introduce reduced (or rescaled) variables ${\mathbf {r}}$ and ${\mathbf {p}}$ (together with the rescaled time variable ${\hat{t}}$),

6.4 $$\begin{aligned} {\mathbf {r}}\equiv \frac{{\mathbf{x}}_1 - \mathbf{x}_2}{GM}, \quad {\mathbf {n}}\equiv \frac{\mathbf{r}}{|{\mathbf{r}}|}, \quad \mathbf{p} \equiv \frac{\mathbf{p}_1}{\mu } = -\frac{\mathbf{p}_2}{\mu }, \quad p_r \equiv {\mathbf {n}}\cdot {\mathbf{p}}, \quad {\hat{t}} \equiv \frac{t}{GM}, \end{aligned}$$

as well as the reduced Hamiltonian

6.5 $$\begin{aligned} {\hat{H}} \equiv \frac{H-Mc^2}{\mu }. \end{aligned}$$

### Conservative Hamiltonians through 4PN order

The conservative reduced 4PN-accurate two-point-mass Hamiltonian in the centre-of-mass frame reads

6.6 $$\begin{aligned} {\hat{H}}[{\mathbf {r}},{\mathbf {p}}]&= {\hat{H}}_{\text {N}}({\mathbf {r}},{\mathbf {p}}) + \frac{1}{c^2} {\hat{H}}_{\text {1PN}}({\mathbf {r}},{\mathbf {p}}) + \frac{1}{c^4} {\hat{H}}_{\text {2PN}}({\mathbf {r}},{\mathbf {p}}) \nonumber \\&\quad + \frac{1}{c^6} {\hat{H}}_{\text {3PN}}({\mathbf {r}},{\mathbf {p}}) + \frac{1}{c^8} {\hat{H}}_{\text {4PN}}[{\mathbf {r}},{\mathbf {p}}]. \end{aligned}$$

The Hamiltonians ${\hat{H}}_{\text {N}}$ through ${\hat{H}}_{\text {3PN}}$ are local in time. They explicitly read

6.7 $$\begin{aligned} {\hat{H}}_{\text {N}}({\mathbf {r}},{\mathbf {p}})= & {} \frac{p^2}{2} - \frac{1}{r}, \end{aligned}$$

6.8 $$\begin{aligned} {\hat{H}}_{\text {1PN}}({\mathbf {r}},{\mathbf {p}})= & {} \frac{1}{8} (3{\nu } -1) p^4 - \frac{1}{2}\left[ (3+{\nu }) p^2 + {\nu } p^2_r\right] \frac{1}{r} + \frac{1}{2r^2}, \end{aligned}$$

6.9 $$\begin{aligned} {\hat{H}}_{\text {2PN}}({\mathbf {r}},{\mathbf {p}})= & {} \frac{1}{16}(1-5{\nu }+5\nu ^2)p^6 \nonumber \\&+\, \frac{1}{8}\big [(5-20{\nu } - 3\nu ^2)p^4 - 2\nu ^2p^2_rp^2 - 3\nu ^2 p^4_r\big ]\frac{1}{r} \nonumber \\&+\, \frac{1}{2}[(5+8{\nu } )p^2 + 3{\nu } p_r^2]\frac{1}{r^2} - \frac{1}{4}(1+3{\nu })\frac{1}{r^3}, \end{aligned}$$

6.10 $$\begin{aligned} {\hat{H}}_{\text {3PN}}({\mathbf {r}},{\mathbf {p}})= & {} \frac{1}{128}(-5+35{\nu } - 70\nu ^2 + 35 \nu ^3) p^8 \nonumber \\&+\, \frac{1}{16}\Big [(-7+42{\nu } - 53\nu ^2 - 5\nu ^3)p^6 +(2-3\nu )\nu ^2p_r^2p^4 \nonumber \\&+\, 3(1-\nu )\nu ^2p_r^4p^2 - 5\nu ^3p_r^6\Big ]\frac{1}{r} + \Big [\frac{1}{16}(-27 + 136{\nu } + 109\nu ^2)p^4 \nonumber \\&+\, \frac{1}{16}(17+30\nu ){\nu } p_r^2p^2 + \frac{1}{12}(5+43\nu ){\nu } p_r^4\Big ]\frac{1}{r^2} \nonumber \\&+\, \Bigg [\left( -\frac{25}{8} + \left( \frac{1}{64} \pi ^2 - \frac{335}{48}\right) {\nu } - \frac{23}{8} \nu ^2\right) p^2 \nonumber \\&+\, \left( -\frac{85}{16} - \frac{3}{64}\pi ^2 - \frac{7}{4} \nu \right) {\nu } p^2_r\Bigg ] \frac{1}{r^3} \nonumber \\&+\, \left[ \frac{1}{8} + \left( \frac{109}{12} - \frac{21}{32} \pi ^2\right) {\nu }\right] \frac{1}{r^4}. \end{aligned}$$

The total 4PN Hamiltonian ${\hat{H}}_{\text {4PN}}[{\mathbf {r}},{\mathbf {p}}]$ is the sum of the local-in-time piece ${\hat{H}}_{\text {4PN}}^{\text {local}}({\mathbf {r}},{\mathbf {p}})$ and the piece ${\hat{H}}_{\text {4PN}}^{\text {nonlocal}}[{\mathbf {r}},{\mathbf {p}}]$ which is nonlocal in time:

6.11 $$\begin{aligned} {\hat{H}}_{\text {4PN}}[{\mathbf {r}},{\mathbf {p}}] = {\hat{H}}_{\text {4PN}}^{\text {local}}({\mathbf {r}},{\mathbf {p}}) + {\hat{H}}_{\text {4PN}}^{\text {nonlocal}}[{\mathbf {r}},{\mathbf {p}}]. \end{aligned}$$

The local-in-time 4PN Hamiltonian ${\hat{H}}_{\text {4PN}}^{\text {local}}({\mathbf {r}},{\mathbf {p}})$ reads

6.12 $$\begin{aligned} {\hat{H}}_{\text {4PN}}^{\text {local}}({\mathbf {r}},{\mathbf {p}}) =&\left( \frac{7}{256} -\frac{63}{256}\nu +\frac{189}{256}\nu ^2 -\frac{105}{128}\nu ^3 +\frac{63}{256}\nu ^4 \right) p^{10} \nonumber \\&+ \Bigg \{ \frac{45}{128} p^8 -\frac{45}{16} p^8\nu +\left( \frac{423}{64} p^8 -\frac{3}{32} p_r^2 p^6 -\frac{9}{64} p_r^4 p^4 \right) \nu ^2 \nonumber \\&+ \left( -\frac{1013}{256} p^8 +\frac{23}{64} p_r^2 p^6 +\frac{69}{128} p_r^4 p^4 -\frac{5}{64} p_r^6 +\frac{35}{256} p_r^8 \right) \nu ^3 \nonumber \\&+ \left( -\frac{35}{128} p^8 -\frac{5}{32} p_r^2 p^6 -\frac{9}{64} p_r^4 p^4 -\frac{5}{32} p_r^6 -\frac{35}{128} p_r^8 \right) \nu ^4 \Bigg \}\frac{1}{r} \nonumber \\&+ \Bigg \{ \frac{13}{8} p^6 + \left( -\frac{791}{64}p^6 +\frac{49}{16} p_r^2 p^4 -\frac{889}{192} p_r^4 +\frac{369}{160} p_r^6 \right) \nu \nonumber \\&+ \left( \frac{4857}{256} p^6 -\frac{545}{64} p_r^2 p^4 +\frac{9475}{768} p_r^4 -\frac{1151}{128} p_r^6 \right) \nu ^2 \nonumber \\&+ \left( \frac{2335}{256} p^6 +\frac{1135}{256} p_r^2 p^4 -\frac{1649}{768} p_r^4 +\frac{10353}{1280} p_r^6 \right) \nu ^3 \Bigg \}\frac{1}{r^2} \nonumber \\&+ \Bigg \{ \frac{105}{32} p^4 + \left[ \left( \frac{2749}{8192}\pi ^2-\frac{589189}{19200}\right) p^4 + \left( \frac{63347}{1600} - \frac{1059}{1024}\pi ^2\right) p_r^2 p^2 \right. \nonumber \\&\left. + \left( \frac{375}{8192}\pi ^2-\frac{23533}{1280}\right) p_r^4 \right] \nu + \bigg [ \left( \frac{18491}{16384}\pi ^2 - \frac{1189789}{28800}\right) p^4 \nonumber \\&- \left( \frac{127}{3} + \frac{4035}{2048}\pi ^2\right) p_r^2 p^2 + \left( \frac{57563}{1920} - \frac{38655}{16384}\pi ^2 \right) p_r^4 \bigg ]\nu ^2 \nonumber \\&+ \bigg ( -\frac{553}{128} p^4 -\frac{225}{64} p_r^2 -\frac{381}{128} p_r^4 \bigg )\nu ^3 \Bigg \}\frac{1}{r^3} \nonumber \\&+ \Bigg \{ \frac{105}{32}p^2 + \left[ \left( \frac{185761}{19200} - \frac{21837}{8192}\pi ^2\right) p^2 + \left( \frac{3401779}{57600} - \frac{28691}{24576}\pi ^2\right) p_r^2 \right] \nu \nonumber \\&+ \left[ \left( \frac{672811}{19200} - \frac{158177}{49152}\pi ^2\right) p^2 + \left( -\frac{21827}{3840} + \frac{110099}{49152}\pi ^2\right) p_r^2 \right] \nu ^2 \Bigg \}\frac{1}{r^4} \nonumber \\&+ \Bigg \{ -\frac{1}{16} + \left( {-\frac{169199}{2400} + \frac{6237}{1024}\pi ^2}\right) \, \nu + \left( -\frac{1256}{45} + \frac{7403}{3072}\pi ^2\right) \,\nu ^2 \Bigg \}\frac{1}{r^5}. \end{aligned}$$

The time-symmetric but nonlocal-in-time Hamiltonian ${\hat{H}}_{\text {4PN}}^{\text {nonlocal}}[{\mathbf {r}},{\mathbf {p}}]$ is related with the leading-order tail effects (Damour et al. 2014). It equals

6.13 $$\begin{aligned} {\hat{H}}_{\text {4PN}}^{\text {nonlocal}}[{\mathbf {r}},{\mathbf {p}}] = -\frac{1}{5}\frac{G^2}{\nu c^8} \dddot{I}_{\!ij}(t) \times {\mathrm {Pf}}_{2r_{12}/c} \int _{-\infty }^{+\infty } \frac{\mathrm {d}\tau }{\vert \tau \vert } \dddot{I}_{\!ij}(t+\tau ), \end{aligned}$$

where ${\mathrm {Pf}}_T$ is a Hadamard partie finie with time scale $T\equiv 2r_{12}/c$ and where $\dddot{I}_{\!ij}$ denotes a third time derivative of the Newtonian quadrupole moment $I_{ij}$ of the binary system,

6.14 $$\begin{aligned} I_{ij} \equiv \sum _a m_a \left( x_a^i x_a^j - \frac{1}{3}\delta ^{ij}{\mathbf {x}}_a^2\right) . \end{aligned}$$

The Hadamard partie finie operation is defined as (Damour et al. 2014)

6.15 $$\begin{aligned} {\mathrm {Pf}}_T \int _0^{+\infty } \frac{\mathrm {d}v}{v}g(v) \equiv \int _0^T \frac{\mathrm {d}v}{v}[g(v)-g(0)] + \int _T^{+\infty } \frac{\mathrm {d}v}{v}g(v). \end{aligned}$$

Let us also note that in reduced variables the quadrupole moment $I_{ij}$ and its third time derivative $\dddot{I}_{\!ij}$ read

6.16 $$\begin{aligned} I_{ij} = (GM)^2 \mu \left( r^ir^j-\frac{1}{3}\mathbf{r}^2\delta ^{ij}\right) , \quad \dddot{I}_{\!ij} = -\frac{\nu }{Gr^2} \left( 4 n^{\langle i} p_{j\rangle } - 3(\mathbf {n}\cdot \mathbf {p})n^{\langle i}n^{j\rangle } \right) , \end{aligned}$$

where $\langle \cdots \rangle$ denotes a symmetric tracefree projection and where in $\dddot{I}_{\!ij}$ the time derivatives $\dot{{\mathbf {r}}}$, $\ddot{{\mathbf {r}}}$, and $\dddot{{\mathbf {r}}}$ were eliminated by means of Newtonian equations of motion.

From the reduced conservative Hamiltonians displayed above, where a factor of $1/\nu$ is factorized out [through the definition (6.5) of the reduced Hamiltonian], the standard test-body dynamics is very easily obtained, simply by putting $\nu =0$. The conservative Hamiltonians ${\hat{H}}_{\text {N}}$ through ${\hat{H}}_{\text {4PN}}$ serve as basis of the EOB approach, where with the aid of a canonical transformation the two-body dynamics is put into test-body form of an effective particle moving in deformed Schwarzschild metric, with $\nu$ being the deformation parameter (Buonanno and Damour 1999, 2000; Damour et al. 2000a, 2015). These Hamiltonians, both directly and through the EOB approach, constitute an important element in the construction of templates needed to detect gravitational waves emitted by coalescing compact binaries. Let us stress again that the complete 4PN Hamiltonian has been obtained only in 2014 (Damour et al. 2014), based on earlier calculations (Blanchet and Damour 1988; Bini and Damour 2013; Jaranowski and Schäfer 2013) and a work published later (Jaranowski and Schäfer 2015).

### Nonlocal-in-time tail Hamiltonian at 4PN order

The nonlocal-in-time tail Hamiltonian at the 4PN level (derived and applied by Damour et al. 2014, 2015, respectively) is the most subtle part of the 4PN Hamiltonian. It certainly deserves some discussion. Let us remark that though the tail Hamiltonian derived in 2016 by Bernard et al. (2016) was identical with the one given in Damour et al. (2014), the derivation there of the equations of motion and the conserved energy was incorrectly done, as detailed by Damour et al. (2016), which was later confirmed by Bernard et al. (2017b).

The 4PN-level tail-related contribution to the action reads

6.17 $$\begin{aligned} S^\text {tail}_\text {4PN} = -\int {H}^{\mathrm{tail}}_\text {4PN}(t)\, \mathrm {d}t, \end{aligned}$$

where the 4PN tail Hamiltonian equals

6.18 $$\begin{aligned} {H}^\text {tail}_\text {4PN}(t) = -\frac{G^2M}{5c^8} \dddot{I}_{\!ij}(t)\, \mathrm{Pf}_{2r(t)/c}\int _{-\infty }^{\infty }\frac{\mathrm {d}v}{|v|}\dddot{I}_{\!ij}(t+v). \end{aligned}$$

Because formally

6.19 $$\begin{aligned} \dddot{I}_{\!ij}(t+v) = \exp \left( v\frac{\mathrm {d}}{\mathrm {d}t}\right) \dddot{I}_{\!ij}(t), \end{aligned}$$

the tail Hamiltonian can also be written as

6.20 $$\begin{aligned} {H}^\text {tail}_\text {4PN}(t)&= - \frac{G^2M}{5c^8} \dddot{I}_{\!ij}(t)\, \mathrm{Pf}_{2r(t)/c}\int _0^{\infty }\frac{\mathrm {d}v}{v}\left[ \dddot{I}_{\!ij}(t+v)+\dddot{I}_{\!ij}(t-v)\right] \nonumber \\&= - \frac{2G^2M}{5c^8} \dddot{I}_{\!ij}(t)\, \mathrm{Pf}_{2r(t)/c}\int _0^{\infty }\frac{\mathrm {d}v}{v} \text{ cosh }\left( v\frac{\mathrm {d}}{\mathrm {d}t}\right) \dddot{I}_{\!ij}(t). \end{aligned}$$

Another writing of the tail Hamiltonian is

6.21 $$\begin{aligned} H^\text {tail}_\text {4PN}(t) = -\frac{2G^2M}{5c^8} \dddot{I}_{\!ij}(t)\, \mathrm{Pf}_{2r(t)/c}\int _0^{\infty }\frac{\mathrm {d}v}{v} \cosh \left( vX(H_0)\right) \dddot{I}_{\!ij}(t) \end{aligned}$$

with

6.22 $$\begin{aligned} X(H_0) \equiv \sum _i \left( \frac{\partial H_0}{\partial p_i(t)}\frac{\partial }{\partial x^i(t)} - \frac{\partial H_0}{\partial x^i(t)}\frac{\partial }{\partial p_i(t)}\right) , \quad H_0 = \frac{(\mathbf{p}(t))^2}{2\mu } - \frac{GM\mu }{r(t)}.\nonumber \\ \end{aligned}$$

This presentation shows that $H^\text {tail}_\text {4PN}$ can be constructed from positions and momenta at time *t*.

For circular orbits, $\dddot{I}_{\!ij}(t)$ is an eigenfunction of $\text{ cosh }\left( v\frac{\mathrm {d}}{\mathrm {d}t}\right)$, reading

6.23 $$\begin{aligned} \cosh \left( v\frac{\mathrm {d}}{\mathrm {d}t}\right) \dddot{I}_{\!ij}(t) = \cos \left( 2v\varOmega (t)\right) \dddot{I}_{\!ij}(t), \end{aligned}$$

where $\varOmega$ is the angular frequency along circular orbit ($p_r=0$),

6.24 $$\begin{aligned} \varOmega (t) \equiv \dot{\varphi } = \frac{\partial H_0(p_{\varphi },r)}{\partial p_{\varphi }} = \frac{p_{\varphi }(t)}{\mu r^2(t)}, \quad H_0(p_{\varphi },r) = \frac{p_\varphi ^2}{2\mu r^2} - \frac{GM\mu }{r}. \end{aligned}$$

Notice that the representation of $\varOmega (t)$ as function of the still independent (dynamical equation $\dot{p}_r=-\partial H_0/\partial r$ has not yet been used) canonical variables $p_\varphi (t)$ and *r*(*t*) (in Damour et al. 2014, 2016, a more concise representation for circular orbits has been applied, based on the orbital angular momentum as only variable). The somewhat complicated structure of Eq. (6.23) can be made plausible by writing $\displaystyle v\frac{\mathrm {d}}{\mathrm {d}t}=v\,\varOmega (p_\varphi ,r)\frac{\mathrm {d}}{\mathrm {d}\varphi }$, see Eq. (6.24), and parametrizing the Eq. (6.16) for circular orbits ($p_r=0$) with orbital angle $\varphi$. The 4PN tail Hamiltonian for circular orbits can thus be written as

6.25 $$\begin{aligned} H^\text {tail circ}_\text {4PN}(t)&= -\frac{2G^2M}{5c^8} \left( \dddot{I}_{\!ij}(t)\right) ^2\, \mathrm{Pf}_{2r(t)/c}\int _0^{\infty }\frac{\mathrm {d}v}{v}\cos \left( \frac{2p_{\phi }(t)}{\mu r^2(t)}v\right) \nonumber \\&= \frac{2G^2M}{5c^8} \left( \dddot{I}_{\!ij}(t)\right) ^2\, \left[ \ln \left( \frac{4p_{\phi }(t)}{\mu c r(t)}\right) + \gamma _\mathrm {E} \right] , \end{aligned}$$

where $\gamma _\mathrm {E}=0.577\ldots$ denotes Euler’s constant. This representation has been used by Bernard et al. (2016), see Eq. (5.32) therein, for a straightforward comparison with the tail results presented by Damour et al. (2014).

### Dynamical invariants of two-body conservative dynamics

The observables of two-body systems that can be measured from infinity by, say, gravitational-wave observations, are describable in terms of dynamical invariants, i.e., functions which do not depend on the choice of phase-space coordinates. Dynamical invariants are easily obtained within a Hamiltonian framework of integrable systems.

We start from the reduced conservative Hamiltonian ${\hat{H}}({\mathbf {r}},{\mathbf {p}})$ in the centre-of-mass frame (we are thus considering here a local-in-time Hamiltonian; for the local reduction of a nonlocal-in-time 4PN-level Hamiltonian see Sect. 6.3.2 below) and we employ reduced variables $({\mathbf {r}},{\mathbf {p}})$. The invariance of ${\hat{H}}({\mathbf {r}},{\mathbf {p}})$ under time translations and spatial rotations leads to the conserved quantities

6.26 $$\begin{aligned} E \equiv {\hat{H}}({\mathbf {r}},{\mathbf {p}}), \quad \mathbf {j} \equiv \frac{\mathbf {J}}{\mu GM} = {\mathbf {r}}\times {\mathbf {p}}, \end{aligned}$$

where *E* is the total energy and $\mathbf {J}$ is the total orbital angular momentum of the binary system in the centre-of-mass frame. We further restrict considerations to the plane of the relative trajectory endowed with polar coordinates $(r,\phi )$ and we use Hamilton–Jacobi approach to obtain the motion. To do this we separate the variables ${\hat{t}}\equiv t/(GM)$ and $\phi$ in the reduced planar action ${\hat{S}}\equiv S/(G\mu M)$, which takes the form

6.27 $$\begin{aligned} {\hat{S}} = -E {\hat{t}} + j \phi + \int \sqrt{R(r,E,j)}\,\mathrm {d}r. \end{aligned}$$

Here $j\equiv |\mathbf {j}|$ and the effective radial potential *R*(*r*, *E*, *j*) is obtained by solving the equation $E={\hat{H}}({\mathbf {r}},{\mathbf {p}})$ with respect to $p_r\equiv {\mathbf {n}}\cdot {\mathbf {p}}$, after making use of the relation

6.28 $$\begin{aligned} {\mathbf {p}}^2 = ({\mathbf {n}}\cdot {\mathbf {p}})^2 + ({\mathbf {n}}\times {\mathbf {p}})^2 = p_r^2 + \frac{j^2}{r^2}. \end{aligned}$$

The Hamilton–Jacobi theory shows that the observables of the two-body dynamics can be deduced from the (reduced) radial action integral

6.29 $$\begin{aligned} i_r(E,j) \equiv \frac{2}{2\pi } \int _{r_\text {min}}^{r_\text {max}} \sqrt{R(r,E,j)}\, \mathrm {d}r, \end{aligned}$$

where the integration is defined from minimal to maximal radial distance. The dimensionless parameter $k\equiv \varDelta \varPhi /(2\pi )$ (with $\varDelta \varPhi \equiv \varPhi -2\pi$) measuring the fractional periastron advance per orbit and the periastron-to-periastron period *P* are obtained by differentiating the radial action integral:

6.30 $$\begin{aligned} k&= -\frac{\partial i_r(E,j)}{\partial j} - 1, \end{aligned}$$

6.31 $$\begin{aligned} P&= 2\pi GM\,\frac{\partial i_r(E,j)}{\partial E}. \end{aligned}$$

It is useful to express the Hamiltonian as a function of the Delaunay (reduced) action variables (see, e.g., Goldstein 1981) defined by

6.32 $$\begin{aligned} n \equiv i_r + j = \frac{\mathcal {N}}{\mu GM}, \quad j = \frac{J}{\mu GM}, \quad m \equiv j_z = \frac{J_z}{\mu GM}. \end{aligned}$$

The angle variables conjugate to *n*, *j*, and *m* are, respectively: the mean anomaly, the argument of the periastron, and the longitude of the ascending node. In the quantum language, ${\mathcal{N}}/\hbar$ is the principal quantum number, $J/\hbar$ the total angular-momentum quantum number, and $J_z/\hbar$ the magnetic quantum number. They are adiabatic invariants of the dynamics and they are, according to the Bohr–Sommerfeld rules of the old quantum theory, (approximately) quantized in integers. Knowing the Delaunay Hamiltonian ${\hat{H}}(n,j,m)$ one computes the angular frequencies of the (generic) rosette motion of the binary system by differentiating ${\hat{H}}$ with respect to the action variables. Namely,

6.33 $$\begin{aligned} \omega _{\text {radial}}&= \frac{2\pi }{P} = \frac{1}{GM} \frac{\partial {\hat{H}}(n,j,m)}{\partial n}, \end{aligned}$$

6.34 $$\begin{aligned} \omega _{\text {periastron}}&= \frac{\varDelta \varPhi }{P} = \frac{2\pi k}{P} = \frac{1}{GM} \frac{\partial {\hat{H}}(n,j,m)}{\partial j}. \end{aligned}$$

Here, $\omega _{\text {radial}}$ is the angular frequency of the radial motion, i.e., the angular frequency of the return to the periastron, while $\omega _{\text {periastron}}$ is the average angular frequency with which the major axis advances in space.

#### 3PN-accurate results

The dynamical invariants of two-body dynamics were computed by Damour and Schäfer (1988) at the 2PN level and then generalized to the 3PN level of accuracy by Damour et al. (2000b). We are displaying here 3PN-accurate formulae. The periastron advance parameter *k* reads

6.35 $$\begin{aligned} k&= \frac{3}{c^2j^2} \Bigg \{ 1 + \frac{1}{c^2} \left[ \frac{5}{4}(7-2\nu )\frac{1}{j^2} + \frac{1}{2}(5-2\nu )\,E \right] \nonumber \\&\quad + \frac{1}{c^4} \Bigg [ \frac{5}{2} \Bigg (\frac{77}{2} + \left( \frac{41}{64}\pi ^2-\frac{125}{3}\right) \nu + \frac{7}{4}\nu ^2\Bigg )\frac{1}{j^4} \nonumber \\&\quad + \Bigg (\frac{105}{2} + \left( \frac{41}{64}\pi ^2-\frac{218}{3}\right) \nu + \frac{45}{6}\nu ^2\Bigg )\frac{E}{j^2} \nonumber \\&\quad + \frac{1}{4}(5-5\nu +4\nu ^2)\,E^2 \Bigg ] + \mathcal {O}(c^{-6}) \Bigg \}. \end{aligned}$$

The 3PN-accurate formula for the orbital period reads

6.36 $$\begin{aligned} P&= \frac{2\pi GM}{(-2E)^{3/2}} \Bigg \{1 - \frac{1}{c^2} \frac{1}{4}(15-\nu ) E \nonumber \\&\quad + \frac{1}{c^4} \left[ \frac{3}{2}(5-2\nu )\frac{(-2E)^{3/2}}{j} -\frac{3}{32}(35+30\nu +3\nu ^2)\,E^2 \right] \nonumber \\&\quad + \frac{1}{c^6} \Bigg [ \Bigg (\frac{105}{2} + \left( \frac{41}{64}\pi ^2-\frac{218}{3}\right) \nu + \frac{45}{6}\nu ^2\Bigg )\frac{(-2E)^{3/2}}{j^3} \nonumber \\&\quad - \frac{3}{4}(5-5\nu +4\nu ^2) \frac{(-2E)^{5/2}}{j} \nonumber \\&\quad + \frac{5}{128}(21-105\nu +15\nu ^2+5\nu ^3)\,E^3 \Bigg ] + \mathcal {O}(c^{-8}) \Bigg \}. \end{aligned}$$

These expressions have direct applications to binary pulsars (Damour and Schäfer 1988). Explicit analytic orbit solutions of the conservative dynamics through 3PN order are given by Memmesheimer et al. (2005). The 4PN periastron advance was first derived by Damour et al. (2015, 2016), with confirmation provided in a later rederivation (Bernard et al. 2017b; also see Le Tiec and Blanchet 2017).

All conservative two-body Hamiltonians respect rotational symmetry, therefore the Delaunay variable *m* does not enter these Hamiltonians. The 3PN-accurate Delaunay Hamiltonian reads (Damour et al. 2000b)

6.37 $$\begin{aligned} {\widehat{H}}(n,j,m)&= -\frac{1}{2n^2} \Bigg \{ 1 + \frac{1}{c^2} \bigg [ \frac{6}{j n}-\frac{1}{4}(15-\nu )\frac{1}{n^2} \bigg ] \nonumber \\&\quad + \frac{1}{c^4} \bigg [ \frac{5}{2}(7-2\nu )\frac{1}{j^3 n} + \frac{27}{j^2 n^2} - \frac{3}{2}(35-4\nu )\frac{1}{j n^3} + \frac{1}{8}(145-15\nu +\nu ^2)\frac{1}{n^4} \bigg ] \nonumber \\&\quad + \frac{1}{c^6} \Bigg [ \Bigg (\frac{231}{2}+\bigg (\frac{123}{64}\pi ^2-125\bigg )\nu +\frac{21}{4}\nu ^2\Bigg )\frac{1}{j^5 n} + \frac{45}{2}(7-2\nu )\frac{1}{j^4 n^2} \nonumber \\&\quad +\Bigg (-\frac{303}{4}+\bigg (\frac{1427}{12}-\frac{41}{64}\pi ^2\bigg )\nu -10\nu ^2\Bigg )\frac{1}{j^3 n^3} - \frac{45}{2}(20-3\nu )\frac{1}{j^2 n^4} \nonumber \\&\quad + \frac{3}{2}(275-50\nu +4\nu ^2)\frac{1}{j n^5} - \frac{1}{64}(6363-805\nu +90\nu ^2-5\nu ^3)\frac{1}{n^6} \Bigg ] + \mathcal {O}(c^{-8}) \Bigg \}. \end{aligned}$$

#### Results at 4PN order

The reduced 4PN Hamiltonian ${\hat{H}}_{\text {4PN}}[{\mathbf {r}},{\mathbf {p}}]$ can be decomposed in two parts in a way slightly different from the splitting shown in Eq. (6.11). Namely,

6.38 $$\begin{aligned} {\hat{H}}_{\text {4PN}}[{\mathbf {r}},{\mathbf {p}}] = {\hat{H}}^{\text {I}}_{\text {4PN}}({\mathbf {r}},{\mathbf {p}};s) + {\hat{H}}^{\text {II}}_{\text {4PN}}[{\mathbf {r}},{\mathbf {p}};s], \end{aligned}$$

where the first part is local in time while the second part is nonlocal in time; $s\equiv {s_\text {phys}}/(GM)$ is a reduced scale with dimension of 1/$\hbox {velocity}^2$, where $s_\text {phys}$ is a scale with dimension of a length. The Hamiltonian ${\hat{H}}^{\text {I}}_{\text {4PN}}$ is a function of phase-space variables $({\mathbf {r}},{\mathbf {p}})$ of the form

6.39 $$\begin{aligned} {\hat{H}}^{\text {I}}_{\text {4PN}}({\mathbf {r}},{\mathbf {p}};s) = {\hat{H}}^{\text {loc}}_{\text {4PN}}({\mathbf {r}},{\mathbf {p}}) + F({\mathbf {r}},{\mathbf {p}})\ln \frac{r}{s}, \quad F({\mathbf {r}},{\mathbf {p}}) \equiv \frac{2}{5}\frac{G^2M}{c^8}(\dddot{I}_{\!ij})^2, \end{aligned}$$

where the Hamiltonian ${\hat{H}}^{\text {loc}}_{\text {4PN}}$ is given in Eq. (6.12) above. The Hamiltonian ${\hat{H}}^{\text {II}}_{\text {4PN}}$ is a functional of phase-space trajectories $({\mathbf {r}}(t),{\mathbf {p}}(t))$,

6.40 $$\begin{aligned} {\hat{H}}^{\text {II}}_{\text {4PN}}[{\mathbf {r}},{\mathbf {p}};s] = -\frac{1}{5}\frac{G^2}{\nu c^8} \dddot{I}_{\!ij}(t) \times {\mathrm {Pf}}_{2s_\text {phys}/c} \int _{-\infty }^{+\infty } \frac{\mathrm {d}\tau }{\vert \tau \vert } \dddot{I}_{\!ij}(t+\tau ). \end{aligned}$$

The nonlocal Hamiltonian ${\hat{H}}^{\text {II}}_{\text {4PN}}[{\mathbf {r}},{\mathbf {p}};s]$ differs from what is displayed in Eq. (6.13) as the nonlocal part of the 4PN Hamiltonian. There the nonlocal piece of ${\hat{H}}_{\text {4PN}}$ is defined by taking as regularization scale in the partie finie operation entering Eq. (6.13) the time $2r_{12}/c$ instead of $2s_{\mathrm{phys}}/c$ appearing in (6.40). Thus the arbitrary scale $s_{\mathrm{phys}}$ enters both parts ${\hat{H}}^{\text {I}}_{\text {4PN}}$ and ${\hat{H}}^{\text {II}}_{\text {4PN}}$ of ${\hat{H}}_{\text {4PN}}$, though it cancels out in the total Hamiltonian. Damour et al. (2015) has shown that modulo some nonlocal-in-time shift of the phase-space coordinates, one can reduce a nonlocal dynamics defined by the Hamiltonian ${\hat{H}}[{\mathbf {r}},{\mathbf {p}};s]\equiv {\hat{H}}_{\text {N}}({\mathbf {r}},{\mathbf {p}})+{\hat{H}}^{\text {II}}_{\text {4PN}}[{\mathbf {r}},{\mathbf {p}};s]$ to an ordinary (i.e., local in time) one. We will sketch here this reduction procedure, which employs the Delaunay form of the Newtonian equations of motion.

It is enough to consider the planar case. In that case the action-angle variables are $(\mathcal {L},\ell ;\mathcal {G},g)$, using the standard notation of Brouwer and Clemence (1961) (with $\mathcal {L}\equiv n$ and $\mathcal {G}\equiv j$). The variable $\mathcal {L}$ is conjugate to the “mean anomaly” $\ell$, while $\mathcal {G}$ is conjugate to the argument of the periastron $g=\omega$. The variables $\mathcal {L}$ and $\mathcal {G}$ are related to the usual Keplerian variables *a* (semimajor axis) and *e* (eccentricity) via

6.41 $$\begin{aligned} \mathcal {L}\equiv \sqrt{a}, \quad \mathcal {G}\equiv \sqrt{a(1-e^2)}. \end{aligned}$$

By inverting (6.41) one can express *a* and *e* as functions of $\mathcal {L}$ and $\mathcal {G}$:

6.42 $$\begin{aligned} a = \mathcal {L}^2, \quad e = \sqrt{1 - \left( \frac{\mathcal {G}}{\mathcal {L}}\right) ^2}. \end{aligned}$$

We use here rescaled variables: in particular, *a* denotes the rescaled semimajor axis $a\equiv a_{\mathrm{phys}}/(GM)$. We also use the rescaled time variable ${\hat{t}}\equiv t_{\mathrm{phys}}/(GM)$ appropriate for the rescaled Newtonian Hamiltonian

6.43 $$\begin{aligned} {\hat{H}}_{\text {N}}(\mathcal {L}) = \frac{1}{2} \, {\mathbf {p}}^2 - \frac{1}{r} = -\frac{1}{2 \mathcal {L}^2}. \end{aligned}$$

The explicit expressions of the Cartesian coordinates (*x*, *y*) of a Newtonian motion in terms of action-angle variables are given by

6.44 $$\begin{aligned} x (\mathcal {L}, \ell ; \mathcal {G}, g)&= \cos g \, x_0 - \sin g \, y_0, \quad y (\mathcal {L}, \ell ; \mathcal {G}, g) = \sin g \, x_0 + \cos g \, y_0, \end{aligned}$$

6.45 $$\begin{aligned} x_0&= a (\cos u - e), \quad y_0 = a \sqrt{1-e^2} \sin u, \end{aligned}$$

where the “eccentric anomaly” *u* is the function of $\ell$ and *e* defined by solving Kepler’s equation

6.46 $$\begin{aligned} u - e \sin u = \ell . \end{aligned}$$

The solution of Kepler’s equation can be written in terms of Bessel functions:

6.47 $$\begin{aligned} u = \ell + \sum _{n=1}^\infty \frac{2}{n} J_n(ne) \sin (n \, \ell ). \end{aligned}$$

Note also the following Bessel–Fourier expansions of $\cos u$ and $\sin u$ [which directly enter $(x_0, y_0)$ and thereby (*x*, *y*)]

6.48 $$\begin{aligned} \cos u&= -\frac{e}{2} + \sum _{n=1}^{\infty } \frac{1}{n} [J_{n-1} (ne) - J_{n+1} (ne)] \cos n \, \ell , \end{aligned}$$

6.49 $$\begin{aligned} \sin u&= \sum _{n=1}^{\infty } \frac{1}{n} [J_{n-1} (ne) + J_{n+1} (ne)] \sin n \, \ell . \end{aligned}$$

For completeness, we also recall the expressions involving the “true anomaly” *f* (polar angle from the periastron) and the radius vector *r*:

6.50 $$\begin{aligned} r&= a (1-e \cos u) = \frac{a (1-e^2)}{1+e \cos f}, \end{aligned}$$

6.51 $$\begin{aligned} \frac{x_0}{r}&= \cos f = \frac{\cos u -e}{1-e \cos u}, \quad \frac{y_0}{r} = \sin f = \frac{\sqrt{1-e^2} \sin u}{1-e \cos u}. \end{aligned}$$

The above expressions allows one to evaluate the expansions of *x*, *y*, and therefrom the components of the quadrupole tensor $I_{ij}$, as power series in *e* and Fourier series in $\ell$.

Let us then consider the expression

6.52 $$\begin{aligned} \mathcal {F}(t,\tau ) \equiv \dddot{I}_{\!ij}(t)\dddot{I}_{\!ij}(t+\tau ), \end{aligned}$$

which enters the nonlocal-in-time piece (6.40) of the Hamiltonian. In order to evaluate the order-reduced value of $\mathcal {F}(t,\tau )$ one needs to use the equations of motion, both for computing the third time derivatives of $I_{ij}$, and for expressing the phase-space variables at time $t+\tau$ in terms of the phase-space variables at time *t*. One employs the zeroth-order equations of motion following from the Newtonian Hamiltonian (6.43),

6.53 $$\begin{aligned} \frac{\mathrm{d}\ell }{\mathrm{d} {{\hat{t}}}}&= \frac{\partial {\hat{H}}_{\text {N}}}{\partial \mathcal {L}} = \frac{1}{\mathcal {L}^3} \equiv \varOmega (\mathcal {L}), \quad \frac{\mathrm{d} g}{\mathrm{d} {{\hat{t}}}} = \frac{\partial {\hat{H}}_{\text {N}}}{\partial \mathcal {G}} = 0, \end{aligned}$$

6.54 $$\begin{aligned} \frac{\mathrm{d}\mathcal {L}}{\mathrm{d} {{\hat{t}}}}&= - \frac{\partial {\hat{H}}_{\text {N}}}{\partial \ell } = 0, \quad \frac{\mathrm{d} \mathcal {G}}{\mathrm{d}{{\hat{t}}}} = - \frac{\partial {\hat{H}}_{\text {N}}}{\partial g} = 0, \end{aligned}$$

where $\varOmega (\mathcal {L}) \equiv \mathcal {L}^{-3}$ is the (${\hat{t}}$-time) rescaled Newtonian (anomalistic) orbital frequency $\varOmega = G M \varOmega _{\mathrm{phys}}$ (it satisfies the rescaled third Kepler’s law: $\varOmega = a^{-3/2}$). The fact that *g*, $\mathcal {L}$, and $\mathcal {G}$ are constant and that $\ell$ varies linearly with time, makes it easy to compute $\dddot{I}_{\!ij} (t+\tau )$ in terms of the values of $(\ell ,g,\mathcal {L},\mathcal {G})$ at time *t*. It suffices to use (denoting by a prime the values at time $t' \equiv t+\tau$)

6.55 $$\begin{aligned} \ell ' \equiv \ell (t+\tau ) = \ell (t) + \varOmega (\mathcal {L}) {\hat{\tau }}, \end{aligned}$$

where ${\hat{\tau }} \equiv \tau /(GM)$, together with $g' = g$, $\mathcal {L}' = \mathcal {L}$, and $\mathcal {G}' = \mathcal {G}$. The order-reduced value of $\mathcal {F}(t,\tau )$ is given by (using $\mathrm {d}/\mathrm {d}{{\hat{t}}} = \varOmega \,\mathrm {d}/\mathrm {d}\ell$)

6.56 $$\begin{aligned} \mathcal {F}(\ell ,{\hat{\tau }}) = \bigg (\frac{\varOmega (\mathcal {L})}{GM}\bigg )^6 \, \frac{\mathrm {d}^3 I_{ij}}{\mathrm {d}\ell ^3}(\ell ) \frac{\mathrm {d}^3 I_{ij}}{\mathrm {d}\ell ^3}(\ell +\varOmega (\mathcal {L}) {\hat{\tau }}). \end{aligned}$$

Inserting the expansion of $I_{ij} (\ell )$ in powers of *e* and in trigonometric functions of $\ell$ and *g*, yields $\mathcal {F}$ in the form of a series of monomials of the type

6.57 $$\begin{aligned} \mathcal {F}(\ell ,{\hat{\tau }}) = \sum _{n_1, n_2, \pm n_3} C_{n_1 n_2 n_3}^{\pm } \, e^{n_1} \cos (n_2 \, \ell \pm n_3 \, \varOmega \, {\hat{\tau }}), \end{aligned}$$

where $n_1$, $n_2$, $n_3$ are natural integers. (Because of rotational invariance, and of the result $g' = g$, there is no dependence of $\mathcal {F}$ on *g*.)

All the terms in the expansion (6.57) containing a nonzero value of $n_2$ will, after integrating over ${\hat{\tau }}$ with the measure $\mathrm {d}{\hat{\tau }}/\vert {\hat{\tau }\vert }$ as indicated in Eq. (6.40), generate a corresponding contribution to ${\hat{H}}^{\text {II}}_{\text {4PN}}$ which varies with $\ell$ proportionally to $\cos (n_2\,\ell )$. One employs now the standard Delaunay technique: any term of the type $A(\mathcal {L})\cos (n\ell )$ in a first-order perturbation $\varepsilon H_1(\mathcal {L},\ell )\equiv {\hat{H}}^{\text {II}}_{\text {4PN}}(\mathcal {L},\ell )$ of the leading-order Hamiltonian $H_0(\mathcal {L})\equiv H_{\text {N}}(\mathcal {L})$ can be eliminated by a canonical transformation with generating function of the type $\varepsilon {\mathfrak {g}}(\mathcal {L},\ell )\equiv \varepsilon B(\mathcal {L}) \sin (n\ell )$. Indeed,

6.58 $$\begin{aligned} \delta _{\mathfrak {g}} H_1 = \{ H_0 (\mathcal {L}), {\mathfrak {g}} \} = - \frac{\partial H_0 (\mathcal {L})}{\partial \mathcal {L}} \, \frac{\partial {\mathfrak {g}}}{\partial \ell } = - n \, \varOmega (\mathcal {L}) \, B(\mathcal {L}) \cos (n\ell ), \end{aligned}$$

so that the choice $B = A/(n \, \varOmega )$ eliminates the term $A \cos (n\ell )$ in $H_1$. This shows that all the periodically varying terms (with $n_2 \ne 0$) in the expansion (6.57) of $\mathcal {F}$ can be eliminated by a canonical transformation. Consequently one can simplify the nonlocal part ${\hat{H}}^{\text {II}}_{\text {4PN}}$ of the 4PN Hamiltonian by replacing it by its $\ell$-averaged value,

6.59 $$\begin{aligned} \hat{{\bar{H}}}^{\text {II}}_{\text {4PN}}(\mathcal {L},\mathcal {G};s) \equiv \frac{1}{2\pi }\int _0^{2\pi }\mathrm {d}\ell \,{\hat{H}}^{\text {II}}_{\text {4PN}}[{\mathbf {r}},{\mathbf {p}};s] = -\frac{1}{5} \, \frac{G^2}{\nu c^8} \, \mathrm{Pf}_{2s/c} \int _{-\infty }^{+\infty } \frac{\mathrm{d} {\hat{\tau }}}{\vert {\hat{\tau }} \vert } \, {\bar{\mathcal {F}}}, \end{aligned}$$

where ${\bar{\mathcal {F}}}$ denotes the $\ell$-average of $\mathcal {F}(\ell , {\hat{\tau }})$ [which is simply obtained by dropping all the terms with $n_2 \ne 0$ in the expansion (6.57)]. This procedure yields an averaged Hamiltonian $\hat{{\bar{H}}}^{\text {II}}_{\text {4PN}}$ which depends only on $\mathcal {L}$, $\mathcal {G}$ (and *s*) and which is given as an expansion in powers of *e* (because of the averaging this expansion contains only even powers of *e*). Damour et al. (2015) derived the $\ell$-averaged Hamiltonian as a power series of the form^5^

6.60 $$\begin{aligned} \hat{{\bar{H}}}^{\text {II}}_{\text {4PN}}(\mathcal {L},\mathcal {G};s) = \frac{4}{5}\frac{\nu }{c^8\mathcal {L}^{10}} \sum _{p=1}^\infty p^6 |{\hat{I}}^p_{ij}(e)|^2 \ln \left( 2p\frac{\mathrm {e}^{\gamma _\text {E}}s}{c\mathcal {L}^3}\right) , \end{aligned}$$

where ${\hat{I}}^p_{ij}(e)$ are coefficients in the Bessel–Fourier expansion of the dimensionless reduced quadrupole moment ${\hat{I}}_{ij}\equiv I_{ij}/[(GM)^2\mu a^2]$,

6.61 $$\begin{aligned} {\hat{I}}_{ij}(\ell ,e) = \sum _{p=-\infty }^{+\infty } {\hat{I}}_{ij}^p(e) \mathrm {e}^{\mathrm {i}p \ell }. \end{aligned}$$

Equation (6.60) is the basic expression for the transition of the tail-related part of the 4PN dynamics to the EOB approach (Damour et al. 2015).

For another approach to the occurrence and treatment of the $(\ell ,\ell ')$-structure in nonlocal-in-time Hamiltonians the reader is referred to Damour et al. (2016) (therein, $\ell$ is called $\lambda$).

### The innermost stable circular orbit

The innermost stable circular orbit (ISCO) of a test-body orbiting in the Schwarzschild metric is located at $R=6MG/c^2$, in Schwarzschild coordinates. Within a Hamiltonian formalism the calculation of the ISCO for systems made of bodies of comparable masses is rather straightforward. It is relevant to start from discussion of dynamics of a two-body system along circular orbits.

The centre-of-mass conservative Hamiltonian ${\hat{H}}({\mathbf {r}},{\mathbf {p}})$ can be reduced to circular orbits by setting $p_r = {\mathbf {n}}\cdot {\mathbf {p}}= 0$ and ${\mathbf {p}}^2 = j^2/r^2$, then ${\hat{H}}={\hat{H}}(r,j)$. Moreover, $\partial {\hat{H}}(r,j)/\partial r = 0$ along circular orbits, what gives the link between *r* and *j*, $r=r(j)$. Finally the energy ${\hat{E}}_\text {circ}$ along circular orbits can be expressed as a function of *j* only, ${\hat{E}}_\text {circ}(j)\equiv {\hat{H}}(r(j),j)$. The link between the (reduced) centre-of-mass energy ${\hat{E}}_\text {circ}$ and the (reduced) angular momentum *j* is explicitly known up to the 4PN order. It reads (Bini and Damour 2013; Damour et al. 2014)

6.62 $$\begin{aligned} {\hat{E}}^\text {circ}(j;\nu ) =&-\frac{1}{2j^2} \Bigg \{ 1 + \bigg (\frac{9}{4}+\frac{1}{4}\nu \bigg )\frac{1}{j^2} + \bigg (\frac{81}{8} - \frac{7}{8}\nu + \frac{1}{8}\nu ^2\bigg )\frac{1}{j^4} \nonumber \\&+ \bigg [ \frac{3861}{64} + \bigg (\frac{41\pi ^2}{32}-\frac{8833}{192}\bigg ) \nu - \frac{5}{32}\nu ^2 + \frac{5}{64}\nu ^3 \bigg ] \frac{1}{j^6} \nonumber \\&+ \bigg [ \frac{53703}{128} + \bigg (\frac{6581\pi ^2}{512}-\frac{989911}{1920}-\frac{64}{5}\bigg (2\gamma _\mathrm {E}+\ln \frac{16}{j^2}\bigg )\bigg )\nu \nonumber \\&+ \bigg (\frac{8875}{384}-\frac{41\pi ^2}{64}\bigg )\nu ^2-\frac{3}{64}\nu ^3+\frac{7}{128}\nu ^4\bigg ] \frac{1}{j^8} + \mathcal {O}(j^{-10}) \Bigg \}. \end{aligned}$$

An important observational quantity is the angular frequency of circular orbits, $\omega _\text {circ}$. It can be computed as

6.63 $$\begin{aligned} \omega _\text {circ} = \frac{1}{GM} \frac{\mathrm {d}{\hat{E}}_\text {circ}}{\mathrm {d}j}. \end{aligned}$$

It is convenient to introduce the coordinate-invariant dimensionless variable (which can also serve as small PN expansion parameter)

6.64 $$\begin{aligned} x \equiv \left( \frac{GM\omega _\text {circ}}{c^3}\right) ^{2/3}. \end{aligned}$$

Making use of Eqs. (6.63) and (6.64) it is not difficult to translate the link of Eq. (6.62) into the dependence of the energy ${\hat{E}}_\text {circ}$ on the parameter *x*. The 4PN-accurate formula reads (Bini and Damour 2013; Damour et al. 2014)

6.65 $$\begin{aligned} {\hat{E}}^\text {circ}(x;\nu )&= -\frac{x}{2} \Bigg \{ 1 - \bigg (\frac{3}{4} + \frac{\nu }{12} \bigg ) x + \bigg (-\frac{27}{8} + \frac{19\nu }{8} - \frac{\nu ^2}{24}\bigg ) x^2 \nonumber \\&\quad + \bigg [ -\frac{675}{64} + \left( \frac{34445}{576}-\frac{205\pi ^2}{96}\right) \nu -\frac{155\nu ^2}{96} - \frac{35\nu ^3}{5184} \bigg ] x^3 \nonumber \\&\quad + \bigg [ -\frac{3969}{128} + \bigg (\frac{9037 \pi ^2}{1536}-\frac{123671}{5760}+\frac{448}{15}\big (2\gamma _\mathrm {E}+\ln (16 x)\big )\bigg )\nu \nonumber \\&\quad + \left( \frac{3157\pi ^2}{576} -\frac{498449}{3456}\right) \nu ^2 + \frac{301\nu ^3}{1728} + \frac{77\nu ^4}{31104} \bigg ] x^4 + \mathcal {O}(x^5) \Bigg \}. \end{aligned}$$

In the test-mass limit $\nu \rightarrow 0$ (describing motion of a test particle on a circular orbit in the Schwarzschild spacetime) the link ${\hat{E}}^\text {circ}(x;\nu )$ is exactly known,

6.66 $$\begin{aligned} {\hat{E}}^\text {circ}(x;0) = \frac{1-2x}{\sqrt{1-3x}} - 1. \end{aligned}$$

The location $x_{\text {ISCO}}=1/6$ of the ISCO in the test-mass limit corresponds to the minimum of the function ${\hat{E}}^\text {circ}(x;0)$, i.e.

6.67 $$\begin{aligned} \frac{\mathrm {d}{\hat{E}}^\text {circ}(x;0)}{\mathrm {d}x}\bigg |_{x=x_{\text {ISCO}}} = 0. \end{aligned}$$

Therefore the most straightforward way of locating the ISCO for $\nu >0$ relies on looking for the minimum of the function ${\hat{E}}^\text {circ}(x;\nu )$, i.e., for a given value of $\nu$, the location of the ISCO is obtained by (usually numerically) solving the equation $\mathrm {d}{\hat{E}}^\text {circ}(x;\nu )/(\mathrm {d}x)=0$ (Blanchet 2002). Equivalently the location of the ISCO can be defined as a solution of the set of simultaneous equations $\partial {\hat{H}}(r,j)/\partial r = 0$ and $\partial ^2{\hat{H}}(r,j)/\partial r^2 = 0$. Both approaches are equivalent only for the *exact* Hamiltonian ${\hat{H}}(r,j)$, see however Sect. IV A 2 in Buonanno et al. (2003, 2006) for subtleties related to equivalence of both approaches when using post-Newtonian-accurate Hamiltonians. With the aid of the latter method, Schäfer and Wex (1993a) computed the *n*PN-accurate ISCO of the test mass in the Schwarzschild metric through 9PN order in three different coordinate systems, obtaining three different results. Clearly, the application of the first method only results in a *n*PN-accurate ISCO described by parameters which are coordinate invariant.

Let us consider the 4PN-accurate expansion of the exact test-mass-limit formula (6.66),

6.68 $$\begin{aligned} {\hat{E}}^\text {circ}(x;0) = -\frac{x}{2} \bigg ( 1 - \frac{3}{4} x - \frac{27}{8} x^2 -\frac{675}{64} x^3 -\frac{3969}{128}x^4 +\mathcal {O}(x^5) \bigg ). \end{aligned}$$

Let us compute the successive PN estimations of the exact ISCO frequency parameter $x_{\text {ISCO}}=1/6\cong 0.166667$ in the test-mass limit, by solving the equations $\mathrm {d}{\hat{E}}_{n\text {PN}}^\text {circ}(x;0)/(\mathrm {d}x)=0$ for $n=1,\ldots ,4$, where the function ${\hat{E}}_{n\text {PN}}^\text {circ}(x;0)$ is defined as the $\mathcal {O}(x^{n+1})$-accurate truncation of the right-hand-side of Eq. (6.68). They read: 0.666667 (1PN), 0.248807 (2PN), 0.195941 (3PN), 0.179467 (4PN). One sees that the 4PN prediction for the ISCO frequency parameter is still $\sim$8% larger than the exact result. This suggests that the straightforward Taylor approximants of the energy function ${\hat{E}}^\text {circ}(x;\nu )$ do not converge fast enough to determine satisfactorily the frequency parameter of the ISCO also in $\nu >0$ case, at least for sufficiently small values of $\nu$. The extrapolation of this statement for larger $\nu$ is supported by the values of the ISCO locations in the equal-mass case ($\nu =1/4$), obtained by solving the equations $\mathrm {d}{\hat{E}}_\text {nPN}^\text {circ}(x;1/4)/(\mathrm {d}x)=0$ for $n=1,\ldots ,4$, where the function ${\hat{E}}_{n\text {PN}}^\text {circ}(x;\nu )$ is now defined as the $\mathcal {O}(x^{n+1})$-accurate truncation of the right-hand-side of Eq. (6.65). For the approximations from 1PN up to 4PN the ISCO locations read (Damour et al. 2000a; Blanchet 2002; Jaranowski and Schäfer 2013): 0.648649 (1PN), 0.265832 (2PN), 0.254954 (3PN), and 0.236599 (4PN).^6^

To overcome the problem of the slow convergence of PN expansions several new methods of determination of the ISCO for comparable-mass binaries were devised by Damour et al. (2000a). They use different “resummation” techniques and are based on the consideration of gauge-invariant functions. One of the methods, called the “*j*-method” by Damour et al. (2000a), employs the invariant function linking the angular momentum and the angular frequency along circular orbits and uses Padé approximants. The ISCO is defined in this method as the minimum, for the fixed value of $\nu$, of the function $j^2(x;\nu )$, where *j* is the reduced angular momentum [introduced in Eq. (6.26)]. The function $j^2(x;\nu )$ is known in the test-mass limit,

6.69 $$\begin{aligned} j^2(x;0) = \frac{1}{x(1-3x)}, \end{aligned}$$

and its minimum coincides with the exact “location” $x_{\text {ISCO}}=1/6$ of the test-mass ISCO. The form of this function suggests to use Padé approximants instead of direct Taylor expansions. It also suggests to require that all used approximants have a pole for some $x_\text {pole}$, which is related with the test-mass “light-ring” orbit occurring for $x_\text {lr}=1/3$ in the sense that $x_\text {pole}(\nu )\rightarrow 1/3$ when $\nu \rightarrow 0$. The 4PN-accurate function $j^2(x;\nu )$ has the symbolic structure $(1/x)(1+x+\cdots +x^4+x^4\ln x)$. In the *j*-method the Taylor expansion at the 1PN level with symbolic form $1+x$ is replaced by Padé approximant of type (0,1), at the 2PN level $1+x+x^2$ is replaced by (1,1) approximant, at the 3PN level $1+x+x^2+x^3$ is replaced by (2,1) approximant, and finally at the 4PN level $1+x+x^2+x^3+x^4$ is replaced by (3,1) Padé approximant [the explicit form of the (0,1), (1,1), and (2,1) approximants can be found in Eqs. (4.16) of Damour et al. 2000a]. At all PN levels the test-mass result is recovered exactly and Jaranowski and Schäfer (2013) showed that the ISCO locations resulting from 3PN-accurate and 4PN-accurate calculations almost coincide for all values of $\nu$, $0\le \nu \le \frac{1}{4}$. The ISCO locations in the equal-mass case $\nu =1/4$ for the approximations from 1PN up to 4PN are as follows (Jaranowski and Schäfer 2013): 0.162162 (1PN), 0.185351 (2PN), 0.244276 (3PN), 0.242967 (4PN).

### Dissipative Hamiltonians

To discuss dissipative Hamiltonians it is convenient to use the toy model from Sect. 3.2 with the Routhian $R(q,p;\xi ,{\dot{\xi }})$ and its corresponding Hamiltonian $H(q,p;\xi ,\pi )=\pi {\dot{\xi }}-R$. The Hamilton equations of motion for the (*q*, *p*) variables read

6.70 $$\begin{aligned} \dot{p} = -\frac{\partial H}{\partial q} = -\frac{\partial R}{\partial q}, \quad \dot{q} = \frac{\partial H}{\partial p} = \frac{\partial R}{\partial p}, \end{aligned}$$

and the Euler–Lagrange equation for the $\xi$ variable is

6.71 $$\begin{aligned} \frac{\partial R}{\partial \xi } - \frac{\mathrm {d}}{\mathrm {d}t}\frac{\partial R}{\partial {\dot{\xi }}} = 0. \end{aligned}$$

Alternatively, the Hamilton equations of motion for the $(\xi ,\pi )$ variables can be used. Solutions of the Euler–Lagrange equation are functions $\xi =\xi (q,p)$. Under those solutions, the Hamilton equations of motion for the (*q*, *p*) variables become

6.72 $$\begin{aligned} \dot{p} = -\frac{\partial R}{\partial q}\bigg |_{\xi =\xi (q,p)}, \quad \dot{q} = \frac{\partial R}{\partial p}\bigg |_{\xi =\xi (q,p)}. \end{aligned}$$

These autonomous equations in the (*q*, *p*) variables contain the full conservative and dissipative content of the (*q*, *p*) dynamics. The time-symmetric part of *R* yields the conservative equations of motion and the time-antisymmetric part of the dissipative ones. The conservative equations of motion agree with the Fokker-type ones showing the same boundary conditions for the $(\xi ,{\dot{\xi }})$ variables. When going from the $(\xi ,{\dot{\xi }})$ variables to the field variables $h^{\mathrm{TT}}$ and $\dot{h}^{\mathrm{TT}}$, those time-symmetric boundary conditions mean as much incoming as outgoing radiation.

To describe astrophysical systems one should use the physical boundary conditions of no incoming radiation and past stationarity. Clearly, radiative dissipation happens now and the time-symmetric part of the whole dynamics makes the conservative part. In linear theories the conservative part just results from the symmetric Green function $G_\text {s}$, whereas the dissipative one from the antisymmetric Green function $G_\text {a}$, which is a homogeneous solution of the wave equation. The both together combine to the retarded Green function $G_{\mathrm{ret}}=G_{\mathrm{s}}+G_{\mathrm{a}}$, with $G_{\mathrm{s}} = (1/2)(G_{\mathrm{ret}} + G_{\mathrm{adv}})$ and $G_{\mathrm{a}} = (1/2)(G_{\mathrm{ret}} - G_{\mathrm{adv}})$, where $G_{\mathrm{adv}}$ denotes the advanced Green function. In non-linear theories time-symmetric effects can also result from homogeneous solutions, e.g., the tail contributions.

For a binary system, the leading-order direct and tail radiation reaction enter the Routhian in the form

6.73 $$\begin{aligned} R^{\text {rr}}({\mathbf {x}}_a,{\mathbf {p}}_a,t) = -\frac{1}{2}\,h^{\text {TT rr}}_{ij}(t)\, \left( \frac{p_{1i}p_{1j}}{m_1} + \frac{p_{2i}p_{2j}}{m_2} - \frac{Gm_1m_2}{r_{12}} n_{12}^i n_{12}^j\right) , \end{aligned}$$

where $h^{\text {TT rr}}_{ij}(t)$ decomposes into a direct radiation-reaction term and a tail one (Damour et al. 2016),

6.74 $$\begin{aligned} h^{\text {TT rr}}_{ij}(t) = -\frac{4G}{5c^5} \left( {I}^{(3)}_{\!ij}(t) + \frac{4GM}{c^3} \int ^\infty _0\mathrm {d}\tau \,\mathrm{ln}\left( \frac{c\tau }{2s_{\mathrm{phys}}}\right) {I}^{(5)}_{\!ij}(t-\tau )\right) . \end{aligned}$$

The last term on the right side results in a Routhian, which reproduces the corresponding tail effects in Blanchet (1993) and Galley et al. (2016).

The conservative (time-symmetric) part in $h^{\text {TT rr}}_{ij}$ reads

6.75 $$\begin{aligned} h^{\text {TT rr con}}_{ij}(t) = -\frac{8G^2M}{5c^8} \mathrm{Pf}_{2s_{\mathrm{phys}}/c} \int ^\infty _{-\infty } \frac{\mathrm {d}t'}{|t-t'|}\,{I}^{(4)}_{\!ij}(t'), \end{aligned}$$

and the dissipative (time-antisymmetric) one equals

6.76 $$\begin{aligned} h^{\text {TT rr dis}}_{ij}(t) = -\frac{4G}{5c^5} {I}^{(3)}_{\!ij}(t) - \frac{8G^2M}{5c^8} \mathrm{Pf}_{2s_{\mathrm{phys}}/c} \int ^\infty _{-\infty }\frac{\mathrm {d}t'}{t-t'}\,{I}^{(4)}_{\!ij}(t'), \end{aligned}$$

where use has been made of the relations

6.77 $$\begin{aligned} \mathrm{Pf}_{\tau _0} \int ^\infty _{-\infty } \frac{\mathrm {d}t' f(t')}{|t-t'|}&= \int ^{\infty }_0 \mathrm {d}\tau ~ \mathrm{ln}\left( \frac{\tau }{\tau _0}\right) [f^{(1)}(t-\tau ) - f^{(1)}(t+\tau )], \end{aligned}$$

6.78 $$\begin{aligned} \mathrm{Pf}_{\tau _0} \int ^\infty _{-\infty } \frac{\mathrm {d}t' f(t')}{t-t'}&= \int ^{\infty }_0 \mathrm {d}\tau ~\mathrm{ln}\left( \frac{\tau }{\tau _0}\right) [f^{(1)}(t-\tau ) + f^{(1)}(t+\tau )]. \end{aligned}$$

The leading-order 2.5PN dissipative binary orbital dynamics is described by the non-autonomous Hamiltonian (Schäfer 1995),

6.79 $$\begin{aligned} H_{\text {2.5PN}}({\mathbf {x}}_a,{\mathbf {p}}_a,t) = \frac{2G}{5c^5}\,\dddot{I}_{\!ij}\big (x'^k_a(t)\big )\, \left( \frac{p_{1i}p_{1j}}{m_1} + \frac{p_{2i}p_{2j}}{m_2} - \frac{Gm_1m_2}{r_{12}} n_{12}^i n_{12}^j\right) , \end{aligned}$$

where $I_{ij}$ is the Newtonian mass-quadrupole tensor,

6.80 $$\begin{aligned} I_{ij}\big (x'^k_a(t)\big ) \equiv \sum _a m_a \big (x'^i_a(t)x'^j_a(t) - \frac{1}{3}{} \mathbf{x}'^2_a(t)\delta _{ij}\big ). \end{aligned}$$

Only after the Hamilton equations of motion have been obtained the primed position and momentum variables coming from $\dddot{I}_{\!ij}$ are allowed to be identified with the unprimed position and momentum variables, also see Galley (2013). Generally, the treatment of dissipation with Hamiltonians or Lagrangians necessarily needs doubling of variables (Bateman 1931). In quantum mechanics, that treatment was introduced by Schwinger (1961) and Keldysh (1965). In the EFT approach as well a doubling of variables is needed if one wants to treat dissipative systems in a full-fledged manner on the action level (see, e.g., Galley and Leibovich 2012; Galley et al. 2016). However, one should keep in mind that in quantum mechanics damping can also be treated without doubling of variables by making use of the fact that the Feynman Green function $G_{\mathrm{F}}$, the analogue of the retarded Green function of classical physics, decomposes into real and imaginary parts, $G_{\mathrm{F}} = G_{\mathrm{s}} + (i/2)G^{(1)}$, where both $G_{\mathrm{s}}$ from above and $G^{(1)}$, Hadamard’s elementary function, are symmetric Green functions, $G^{(1)}$ solving homogeneous wave equation as $G_a$ does. The imaginary part in e.g. the Eq. (8.7.57) in the book by Brown (1992) yields nothing but the dipole radiation loss formula and this without any doubling of variables (also see Sect. 9-4 in Feynman and Hibbs 1965).

Applications of the 2.5PN Hamiltonian can be found in, e.g., Kokkotas and Schäfer (1995), Ruffert et al. (1996), Buonanno and Damour (1999), and Gopakumar and Schäfer (2008), where in Gopakumar and Schäfer (2008) a transformation to the Burke–Thorne gauge (coordinate conditions) is performed. More information on the 2.5PN dissipation can be found in Damour (1987a). The 3.5PN Hamiltonian for many point-mass systems is known too, it is displayed in Appendix E (Jaranowski and Schäfer 1997; Königsdörffer et al. 2003). Regarding gravitational spin interaction, see the next section, also for this case radiation reaction Hamiltonians have been derived through leading order spin–orbit and spin–spin couplings (Steinhoff and Wang 2010; Wang et al. 2011). Recent related developments within the EFT formalism are found in Maia et al. (2017a, b).

Let us mention that the already cited article Galley et al. (2016) contains two interesting results improving upon and correcting an earlier article by Foffa and Sturani (2013b): on the one hand it confirms the conservative part of the tail action, particularly the additional rational constant 41/30 which corresponds to the famous 5/6 in the Lamb shift (see, e.g., Brown 2000), and on the other side it correctly delivers the dissipative part of the tail interaction. It is worth noting that in the both articles the involved calculations were performed in harmonic coordinates.

## Generalized ADM formalism for spinning objects

In this section we review the recent generalization of ADM formalism describing dynamics of systems made of spinning point masses or, more precisely, pole–dipole particles. We start from reviewing the generalization which is of fully reduced form (i.e., without unresolved constraints, spin supplementary and coordinate conditions) and which is valid to linear order in spin variables [our presentation of linear-in-spins dynamics closely follows that of Steinhoff and Schäfer (2009a)].

### Dynamics linear in spins

In this section Latin indices from the middle of the alphabet *i*, *j*, *k*, $\ldots$ are running through $\{1,2,3\}$. We utilize three different reference frames here, denoted by different indices. Greek indices refer to the *coordinate frame* $(x^\mu )$ and have the values $\mu =0,1,2,3$. Lower case Latin indices from the beginning of the alphabet refer to the *local Lorentz frame* with its associated tetrad fields $\big (e_a^\mu (x^\nu )\big )$ ($e_a^\mu$ denotes thus the $\mu$ coordinate-frame component of the tetrad vector of label *a*), while upper case ones denote the so-called *body-fixed Lorentz frame* with its associated “tetrad” $\big (\varLambda ^{~a}_A(z^\mu )\big )$, where $(z^\mu )$ denotes coordinate-frame components of the body’s position (so $\varLambda ^{~a}_A$ is the *a* local-Lorentz-frame component of the tetrad vector of label *A*). The values of these Lorentz indices are marked by round and square brackets as $a=(0),(i)$ and $A=[0],[i]$, respectively, e.g., $A=[0],[1],[2],[3]$. The basics of the tetrad formalism in GR can be found in, e.g., Sect. 12.5 of Weinberg (1972).

In GR, the coupling of a spinning object to a gravitational field, in terms of a Lagrangian density, reads

7.1 $$\begin{aligned} \mathcal {L}_M = \int \mathrm {d}\tau \left[ \left( p_{\mu } - \frac{1}{2} S_{ab} \,\omega _{\mu }^{~ab}\right) \frac{\mathrm {d}z^{\mu }}{\mathrm {d}\tau } + \frac{1}{2} S_{ab} \frac{\delta \theta ^{ab}}{\mathrm {d}\tau } \right] \delta ^{(4)}(x^{\nu }-z^{\nu }(\tau )). \end{aligned}$$

The linear momentum variable is $p_{\mu }$ and the spin tensor is denoted by $S_{ab}$. The object’s affine time variable is $\tau$ and $\delta ^{(4)}(x^{\nu }-z^{\nu }(\tau ))$ is the 4-dimensional Dirac delta function (from now on we will abbreviate it to $\delta ^{(4)}$). The angle variables are represented by some Lorentz matrix satisfying $\varLambda ^{Aa} \varLambda ^{Bb} \eta _{AB} = \eta ^{ab}$ or $\varLambda _{Aa} \varLambda _{Bb} \eta ^{ab} = \eta _{AB}$, where $\eta _{AB} = \text{ diag }(-1,1,1,1) = \eta ^{ab}$, which must be respected upon infinitesimal Lorentz transformations (see Hanson and Regge 1974), so $\delta \theta ^{ab}\equiv \varLambda _{C}^{~a}\mathrm {d}\varLambda ^{Cb}=-\delta \theta ^{ba}$. The Ricci rotation coefficients $\omega _{\mu }^{~ab}$ are given by $\omega _{\mu \alpha \beta }=e_{a\alpha }e_{b\beta }\omega _{\mu }^{~ a b}=-\varGamma _{\beta \alpha \mu }^{(4)} + e_{\alpha ,\mu }^c e_{c\beta }$, with $\varGamma _{\beta \alpha \mu }^{(4)} = \frac{1}{2} (g_{\beta \alpha ,\mu } + g_{\beta \mu ,\alpha } - g_{\alpha \mu ,\beta })$ as the 4-dimensional Christoffel symbols of the first kind with $g_{\mu \nu } = e_{a\mu } e_{b\nu } \eta ^{ab}$ the 4-dimensional metric. As in Hanson and Regge (1974), the matrix $\varLambda ^{Ca}$ can be subjected to right (or left) Lorentz transformations, which correspond to transformations of the local Lorentz reference frame (or the body-fixed frame, respectively). In the action (7.1) only a minimal coupling between spin variables and gravitational field is employed; for more general (than minimal) couplings, the reader is referred to Bailey and Israel (1975).

The matter constraints are given by, also in terms of a Lagrangian density,

7.2 $$\begin{aligned} \mathcal {L}_C = \int \mathrm {d}\tau \left[ \lambda _1^a p^b S_{ab} + \lambda _{2[i]} \varLambda ^{[i] a} p_a - \frac{\lambda _3}{2} (p^2 + m^2c^2) \right] \delta ^{(4)}, \end{aligned}$$

where *m* is the constant mass of the object, $p^2\equiv p_{\mu }p^{\mu }$, and $\lambda _1^a$, $\lambda _{2[i]}$, $\lambda _3$ are the Lagrange multipliers. The constraint

7.3 $$\begin{aligned} p^b S_{ab} = 0 \end{aligned}$$

is called the *spin supplementary condition* (SSC), it states that in the rest frame the spin tensor contains the 3-dimensional spin $S_{(i)(j)}$ only (i.e., the mass-dipole part $S_{(0)(i)}$ vanishes).^7^ The conjugate constraint $\varLambda ^{[i] a} p_a = 0$ ensures that $\varLambda ^{C a}$ is a pure 3-dimensional rotation matrix in the rest frame (no Lorentz boosts), see Hanson and Regge (1974). Finally, the gravitational part is given by the usual Einstein–Hilbert Lagrangian density

7.4 $$\begin{aligned} \mathcal {L}_G = \frac{c^4}{16\pi G} \sqrt{-g} R^{(4)}, \end{aligned}$$

where *g* is the determinant of the 4-dimensional metric and $R^{(4)}$ is the 4-dimensional Ricci scalar. Using a second-order form of the gravitational action, i.e., not varying the connection independently, ensures that the torsion tensor vanishes, see, e.g., Nelson and Teitelboim (1978). The complete Lagrangian density is the sum

7.5 $$\begin{aligned} \mathcal {L} = \mathcal {L}_G + \mathcal {L}_M + \mathcal {L}_C. \end{aligned}$$

We assume space-asymptotic flatness as a boundary condition of the spacetime. The total action is given in a second-order form, where the Ricci rotation coefficients are not independent field degrees of freedom and where no torsion of spacetime shows up. It reads

7.6 $$\begin{aligned} W[e_{a\mu },z^{\mu },p_{\mu },\varLambda ^{Ca},S_{ab},\lambda _1^a,\lambda _{2[i]},\lambda _3] = \int \mathrm {d}t\,\mathrm {d}^3x\, \mathcal {L}, \end{aligned}$$

and must be varied with respect to the tetrad field $e_{a\mu }$, the Lagrange multipliers $\lambda _1^a$, $\lambda _{2[i]}$, $\lambda _3$, position $z^{\mu }$ and linear momentum $p_{\mu }$ of the object, as well as with respect to angle-type variables $\varLambda ^{Ca}$ and spin tensor $S_{ab}$ associated with the object.

Variation of the action $\delta W = 0$ leads to the equations of motion for the matter variables (here $\mathrm {d}$ and $\mathrm {D}$ denote ordinary and covariant total derivatives, respectively)

7.7 $$\begin{aligned} \frac{\mathrm {D}S_{ab}}{\mathrm {D}\tau }&= 0, \quad \frac{\mathrm {D}\varLambda ^{Ca}}{\mathrm {D}\tau } = 0, \quad u^{\mu } \equiv \frac{\mathrm {d}z^\mu }{\mathrm {d}\tau } = \lambda _3 p^{\mu }, \end{aligned}$$

7.8 $$\begin{aligned} \frac{\mathrm {D}p_{\mu }}{\mathrm {D}\tau }&= -\frac{1}{2} R_{\mu \rho ab}^{(4)} u^{\rho } S^{ab}, \end{aligned}$$

as well as to the usual Einstein equations with the stress–energy tensor (cf. Tulczyjew 1957 and Sect. 12.5 in Weinberg 1972,8)

7.9 $$\begin{aligned} T^{\mu \nu }&= \frac{e^{\mu }_a}{\sqrt{-g}} \frac{\delta ( \mathcal {L}_M + \mathcal {L}_C )}{\delta e_{a \nu }} \nonumber \\&= \int \mathrm {d}\tau \left[ \lambda _3 p^{\mu } p^{\nu } \frac{\delta ^{(4)}}{\sqrt{-g}} + \bigg ( u^{(\mu } S^{\nu )\alpha } \frac{\delta ^{(4)}}{\sqrt{-g}} \bigg )_{||\alpha } \right] , \end{aligned}$$

where $R_{\mu \rho ab}^{(4)}$ is the 4-dimensional Riemann tensor in mixed indices, $_{||\alpha }$ denotes the 4-dimensional covariant derivative. Here it was already used that preservation of the constraints in time requires $\lambda _1^a$ to be proportional to $p^a$ and $\lambda _{2[i]}$ to be zero, so that $\lambda _1^a$ and $\lambda _{2[i]}$ drop out of the matter equations of motion and the stress–energy tensor. The Lagrange multiplier $\lambda _3 = \lambda _3(\tau )$ represents the reparametrization invariance of the action (notice $\lambda _3=\sqrt{-u^2}/m$). Further, an antisymmetric part of the stress–energy tensor vanishes,

7.10 $$\begin{aligned} \frac{1}{2} \int \mathrm {d}\tau \left( S^{\mu \nu } u^{\rho } \frac{\delta ^{(4)}}{\sqrt{-g}} \right) _{||\rho } = \frac{1}{2} \int \mathrm {d}\tau \frac{\mathrm {D}S^{\mu \nu }}{\mathrm {D}\tau } \frac{\delta ^{(4)}}{\sqrt{-g}} = 0, \end{aligned}$$

and ${T^{\mu \nu }}_{||\nu }=0$ holds by virtue of the matter equations of motion. Obviously, the spin length *s* as defined by $2s^2\equiv S_{ab}S^{ab}$ is conserved.

A fully reduced action is obtained by the elimination of all constraints and gauge degrees of freedom. However, after that the action has still to be transformed into canonical form by certain variable transformations. To perform this reduction we employ $3+1$ splitting of spacetime by spacelike hypersurfaces $t=\text {const}$. The timelike unit covector orthogonal to these hypersurfaces reads $n_{\mu }=(-N,0,0,0)$ or $n^{\mu }=(1,-N^i)/N$. The three matter constraints can then be solved in terms of $p_i$, $S_{ij}$, and $\varLambda ^{[i](k)}$ as

7.11 $$\begin{aligned} np&\equiv n^{\mu } p_{\mu } = -\sqrt{m^2c^2 + \gamma ^{ij} p_{i} p_{j}}, \end{aligned}$$

7.12 $$\begin{aligned} nS_{i}&\equiv n^{\mu } S_{\mu i} = \frac{p_{k} \gamma ^{kj} S_{ji}}{np} = \gamma _{ij} nS^j,\end{aligned}$$

7.13 $$\begin{aligned} \varLambda ^{[j](0)}&= \varLambda ^{[j](i)} \frac{p_{(i)}}{p^{(0)}}, \quad \varLambda ^{[0]a} = -\frac{p^a}{mc}. \end{aligned}$$

We take $\mathcal {L}_C=0$ from now on. A split of the Ricci rotation coefficients results in

7.14 $$\begin{aligned} \omega _{kij}&= -\varGamma _{jik} + e_{i,k}^a e_{aj}, \end{aligned}$$

7.15 $$\begin{aligned} n^{\mu } \omega _{k \mu i}&= K_{ki} - g_{ij} \frac{N^j_{,k}}{N} + \frac{e_{ai}}{N} (e^a_{0,k} - e^a_{l,k} N^l), \end{aligned}$$

7.16 $$\begin{aligned} \omega _{0ij}&= N K_{ij} - N_{j;i} + e_{i,0}^a e_{aj}, \end{aligned}$$

7.17 $$\begin{aligned} n^{\mu } \omega _{0 \mu i}&= K_{ij} N^j - N_{;i} - \gamma _{ij} \frac{N^j_{,0}}{N} + \frac{e_{ai}}{N} (e^a_{0,0} - e^a_{l,0} N^l), \end{aligned}$$

where $_{;i}$ denotes the 3-dimensional covariant derivative, $\varGamma _{jik}$ the 3-dimensional Christoffel symbols, and the extrinsic curvature $K_{ij}$ is given by $2NK_{ij}=-\gamma _{ij,0}+2N_{(i;j)}$, where $_{(\cdots )}$ denotes symmetrization.

It is convenient to employ here the time gauge (see Schwinger 1963a and also Dirac 1962; Kibble 1963; Nelson and Teitelboim 1978),

7.18 $$\begin{aligned} e^{\mu }_{(0)} = n^{\mu }. \end{aligned}$$

Then lapse and shift turn into Lagrange multipliers in the matter action, like in the ADM formalism for nonspinning matter points. The condition (7.18) leads to the following relations:

7.19 $$\begin{aligned} e_{i}^{(0)}&= 0 = e_{(i)}^0, \quad e^{(0)}_0 = N = 1/e_{(0)}^0, \end{aligned}$$

7.20 $$\begin{aligned} N^i&= -N e^{i}_{(0)}, \quad e^{(i)}_0 = N^j e^{(i)}_j, \end{aligned}$$

7.21 $$\begin{aligned} \gamma _{ij}&= e_i^{(m)} e_{(m)j}, \quad \gamma ^{ij} = e^i_{(m)} e^{(m)j}, \end{aligned}$$

which effectively reduce the tetrad $e^{a\mu }$ to a triad $e^{(i)j}$.

The matter part of the Lagrangian density, after making use of the covariant SSC (7.3), turns into

7.22 $$\begin{aligned} \mathcal {L}_M = \mathcal {L}_{MK} + \mathcal {L}_{MC} + \mathcal {L}_{GK} + (\text{ td }), \end{aligned}$$

where $(\text{ td })$ denotes an irrelevant total divergence. After fixing the yet arbitrary parameter $\tau$ by choosing $\tau =z^0=ct$, where *t* is the time coordinate, the terms attributed to the kinetic matter part are given by

7.23 $$\begin{aligned} \mathcal {L}_{MK}&= \bigg [ p_{i} + K_{ij} nS^j + A^{kl} e_{(j)k} e_{l,i}^{(j)} -\bigg ( \frac{1}{2} S_{kj} + \frac{p_{(k} nS_{j)}}{np} \bigg ) \varGamma ^{kj}_{~~i} \bigg ] \dot{z}^{i} \delta + \frac{nS^i}{2 np} \dot{p}_i \delta \nonumber \\&\quad +\bigg [ S_{(i)(j)} + \frac{nS_{(i)} p_{(j)} - nS_{(j)} p_{(i)}}{np} \bigg ] \frac{\varLambda _{[k]}^{(i)} \dot{\varLambda }^{[k](j)}}{2} \delta , \end{aligned}$$

where $\delta \equiv \delta (x^i - z^i(t))$ and $A^{ij}$ is defined by

7.24 $$\begin{aligned} \gamma _{ik} \gamma _{jl} A^{kl} = \frac{1}{2} S_{ij} + \frac{nS_i p_j}{2 np}. \end{aligned}$$

The matter parts of the gravitational constraints result from

7.25 $$\begin{aligned} \mathcal {L}_{MC} = -N\mathcal {H}^{\mathrm{matter}} + N^i \mathcal {H}^{\mathrm{matter}}_i, \end{aligned}$$

where the densities $\mathcal {H}^{\mathrm{matter}}$ and $\mathcal {H}^{\mathrm{matter}}_i$ are computed from Eqs. (2.11)–(2.12) and (7.9). After employing the covariant SSC one gets (Steinhoff et al. 2008c)

7.26 $$\begin{aligned} \mathcal {H}^{\mathrm{matter}}&= \sqrt{\gamma }T_{\mu \nu } n^{\mu }n^{\nu } = -np \delta - K^{ij} \frac{p_i nS_j}{np} \delta - ( nS^k \delta )_{;k}, \end{aligned}$$

7.27 $$\begin{aligned} \mathcal {H}^{\mathrm{matter}}_i&= -\sqrt{\gamma }T_{i \nu }n^{\nu } = (p_i + K_{ij} nS^j ) \delta + \bigg ( \frac{1}{2} \gamma ^{mk} S_{ik} \delta + \delta _i^{(k} \gamma ^{l)m} \frac{p_k nS_l}{np} \delta \bigg )_{;m}. \end{aligned}$$

Further, some terms attributed to the kinetic part of the gravitational field appear as

7.28 $$\begin{aligned} \mathcal {L}_{GK} = A^{ij} e_{(k)i} \dot{e}_{j}^{(k)} \delta . \end{aligned}$$

Now we proceed to Newton–Wigner (NW) variables ${\hat{z}}^i$, $P_i$, ${\hat{S}}_{(i)(j)}$, and $\hat{\varLambda }^{[i](j)}$, which turn the kinetic matter part $\mathcal {L}_{MK}$ into canonical form. The variable transformations read

7.29 $$\begin{aligned} z^i&= {\hat{z}}^i - \frac{nS^i}{mc - np}, \quad nS_i = -\frac{p_k\gamma ^{kj}{\hat{S}}_{ji}}{mc}, \end{aligned}$$

7.30 $$\begin{aligned} S_{ij}&= {\hat{S}}_{ij} - \frac{p_i nS_{j}}{mc-np} + \frac{p_j nS_i}{mc - np}, \end{aligned}$$

7.31 $$\begin{aligned} \varLambda ^{[i](j)}&= \hat{\varLambda }^{[i](k)} \bigg ( \delta _{kj} + \frac{p_{(k)}p^{(j)}}{mc(mc-np)} \bigg ), \end{aligned}$$

7.32 $$\begin{aligned} P_i&= p_i + K_{ij} nS^j + {\hat{A}}^{kl} e_{(j)k} e_{l,i}^{(j)} - \bigg ( \frac{1}{2} S_{kj} + \frac{p_{(k} nS_{j)}}{np} \bigg ) \varGamma ^{kj}_{~~i}, \end{aligned}$$

where ${\hat{A}}^{ij}$ is given by

7.33 $$\begin{aligned} \gamma _{ik} \gamma _{jl} {\hat{A}}^{kl} = \frac{1}{2} {\hat{S}}_{ij} + \frac{mc p_{(i} nS_{j)}}{np (mc-np)}. \end{aligned}$$

The NW variables have the important properties ${\hat{S}}_{(i)(j)} {\hat{S}}_{(i)(j)}=2s^2=\text{ const }$ and $\hat{\varLambda }_{[k]}^{(i)}\hat{\varLambda }^{[k](j)} = \delta _{ij}$, which implies that $\delta \hat{\theta }^{(i)(j)}\equiv \hat{\varLambda }_{[k]}^{(i)}\mathrm {d}\hat{\varLambda }^{[k](j)}$ is antisymmetric. The redefinitions of position, spin tensor, and angle-type variables are actually quite natural generalizations of their Minkowski space versions to curved spacetime, cf. Hanson and Regge (1974) and Fleming (1965). However, there is no difference between linear momentum $p_i$ and canonical momentum $P_i$ in the Minkowski case. In these NW variables, one has

7.34 $$\begin{aligned} \mathcal {L}_{GK} + \mathcal {L}_{MK} = \hat{\mathcal {L}}_{GK} + \hat{\mathcal {L}}_{MK} + (\text{ td }), \end{aligned}$$

with [from now on $\delta = \delta (x^i - {\hat{z}}^i(t))$]

7.35 $$\begin{aligned} \hat{\mathcal {L}}_{MK}&= P_i \dot{{\hat{z}}}^i \delta + \frac{1}{2} {\hat{S}}_{(i)(j)} \dot{\hat{\theta }}^{(i)(j)} \delta , \end{aligned}$$

7.36 $$\begin{aligned} \hat{\mathcal {L}}_{GK}&= {\hat{A}}^{ij} e_{(k)i} e_{j,0}^{(k)} \delta . \end{aligned}$$

Notice that all $\dot{p}_i$ terms in the action have been canceled by the redefinition of the position and also all $K_{ij}$ terms were eliminated from $\mathcal {L}_{MC}$ and $\mathcal {L}_{MK}$ by the redefinition of the linear momentum. If the terms explicitly depending on the triad $e^{(i)}_j$ are neglected, the known source terms of Hamilton and momentum constraints in canonical variables are obtained [cf. Eqs. (4.23) and (4.25) in Steinhoff et al. (2008c)].

The final step goes with the ADM action functional of the gravitational field (Arnowitt et al. 1962; De Witt 1967; Regge and Teitelboim 1974), but in tetrad form as derived by Deser and Isham (1976). The canonical momentum conjugate to $e_{(k)j}$ is given by

7.37 $$\begin{aligned} \bar{\pi }^{(k)j} = \frac{8\pi G}{c^3}\frac{\partial \mathcal {L}}{\partial e_{(k)j,0}} = e_i^{(k)} \pi ^{ij} + e_i^{(k)} \frac{8\pi G}{c^3}{\hat{A}}^{ij} \delta , \end{aligned}$$

where the momentum $\pi ^{ij}$ is given by

7.38 $$\begin{aligned} \pi ^{ij} = \sqrt{\gamma } (\gamma ^{ij}\gamma ^{kl} - \gamma ^{ik}\gamma ^{jl})K_{kl}. \end{aligned}$$

Legendre transformation leads to

7.39 $$\begin{aligned} \hat{\mathcal {L}}_{GK} + \mathcal {L}_G = \frac{c^3}{8\pi G} \bar{\pi }^{(k)j} e_{(k)j,0} - \frac{c^4}{16\pi G} \mathcal {E}_{i,i} + \mathcal {L}_{GC} + (\text{ td }). \end{aligned}$$

In asymptotically flat spacetimes the quantity $\mathcal {E}_i$ is given by [cf. Eq. (2.6)]

7.40 $$\begin{aligned} \mathcal {E}_i = \gamma _{ij,j} - \gamma _{jj,i}. \end{aligned}$$

The total energy then reads

7.41 $$\begin{aligned} E = \frac{c^4}{16\pi G}\oint \mathrm {d}^2s_i\,\mathcal {E}_i. \end{aligned}$$

The constraint part of the gravitational Lagrangian density takes the form

7.42 $$\begin{aligned} \mathcal {L}_{GC} = - N \mathcal {H}^{\mathrm{field}} + N^i \mathcal {H}^{\mathrm{field}}_i, \end{aligned}$$

with

7.43 $$\begin{aligned} \mathcal {H}^{\mathrm{field}}&= - \frac{c^4}{16\pi G\sqrt{\gamma }} \left[ \gamma R + \frac{1}{2} \left( \gamma _{ij} \pi ^{ij} \right) ^2 - \gamma _{ij} \gamma _{k l} \pi ^{ik} \pi ^{jl}\right] , \end{aligned}$$

7.44 $$\begin{aligned} \mathcal {H}^{\mathrm{field}}_i&= \frac{c^3}{8\pi G} \gamma _{ij} \pi ^{jk}_{~~ ; k}, \end{aligned}$$

where *R* is the 3-dimensional Ricci scalar. Due to the symmetry of $\pi ^{ij}$, not all components of $\bar{\pi }^{(k)j}$ are independent variables (i.e., the Legendre map is not invertible), leading to the additional constraint ([...] denotes anti-symmetrization)

7.45 $$\begin{aligned} \bar{\pi }^{[ij]} = \frac{8\pi G}{c^3} {\hat{A}}^{[ij]} \delta . \end{aligned}$$

This constraint will be eliminated by going to the spatial symmetric gauge (for the frame $e_{(i)j}$)

7.46 $$\begin{aligned} e_{(i)j} = e_{ij} = e_{ji}, \quad e^{(i)j} = e^{ij} = e^{ji}. \end{aligned}$$

Then the triad is fixed as the matrix square-root of the 3-dimensional metric, $e_{ij} e_{jk} = \gamma _{ik}$, or, in matrix notation,

7.47 $$\begin{aligned} (e_{ij}) = \sqrt{(\gamma _{ij})}. \end{aligned}$$

Therefore, we can define a quantity $B^{kl}_{ij}$ as

7.48 $$\begin{aligned} e_{k[i} e_{j]k,\mu } = B^{kl}_{ij} \gamma _{kl,\mu }, \end{aligned}$$

or, in explicit form,

7.49 $$\begin{aligned} 2 B^{kl}_{ij} = e_{mi} \frac{\partial e_{mj}}{\partial g_{kl}} - e_{mj} \frac{\partial e_{mi}}{\partial g_{kl}}. \end{aligned}$$

This expression may be evaluated perturbatively, cf. Steinhoff et al. (2008c). One also has $B^{kl}_{ij} \delta _{kl} = 0$. Furthermore,

7.50 $$\begin{aligned} e_{(k)i} e_{j,\mu }^{(k)} = B^{kl}_{ij} \gamma _{kl,\mu } + \frac{1}{2} \gamma _{ij,\mu }, \end{aligned}$$

which yields

7.51 $$\begin{aligned} \bar{\pi }^{(k)j} e_{(k)j,0} = \frac{1}{2} \pi _{\mathrm{can}}^{ij} \gamma _{ij,0}, \end{aligned}$$

with the new canonical field momentum

7.52 $$\begin{aligned} \pi _{\mathrm{can}}^{ij} = \pi ^{ij} + \frac{8\pi G}{c^3} {\hat{A}}^{(ij)} \delta + \frac{16\pi G}{c^3} B^{ij}_{kl} {\hat{A}}^{[kl]} \delta . \end{aligned}$$

The gravitational constraints arising from the variations $\delta N$ and $\delta N^i$ read,

7.53 $$\begin{aligned} \mathcal {H}^{\mathrm{field}} + \mathcal {H}^{\mathrm{matter}} = 0, \quad \mathcal {H}^{\mathrm{field}}_i + \mathcal {H}^{\mathrm{matter}}_i = 0. \end{aligned}$$

They are eliminated by imposing the gauge conditions

7.54 $$\begin{aligned} 3 \gamma _{ij,j} - \gamma _{jj,i} = 0, \quad \pi ^{ii}_{\mathrm{can}} = 0, \end{aligned}$$

which allow for the decompositions

7.55 $$\begin{aligned} \gamma _{ij} = \varPsi ^4 \delta _{ij} + h^{\mathrm{TT}}_{ij}, \quad \pi ^{ij}_{\mathrm{can}} = {\tilde{\pi }}^{ij}_{\mathrm{can}} + \pi ^{ij\mathrm TT}_{\mathrm{can}}, \end{aligned}$$

where $h^{\mathrm{TT}}_{ij}$ and $\pi ^{ij\mathrm TT}_{\mathrm{can}}$ are transverse and traceless quantities, and longitudinal part ${\tilde{\pi }}^{ij}_{\mathrm{can}}$ is related to a vector potential $V^i_{\mathrm{can}}$ by

7.56 $$\begin{aligned} {\tilde{\pi }}^{ij}_{\mathrm{can}} = V^i_{\mathrm{can},j} + V^j_{\mathrm{can},i} - \frac{2}{3} \delta _{ij} V^k_{\mathrm{can},k}. \end{aligned}$$

Let us note that in the construction of $V^i_{\mathrm{can}}$ the operator $\varDelta ^{-1}$ is employed [see the text below Eq. (2.15)].

The gravitational constraints can now be solved for $\varPsi$ and ${\tilde{\pi }}^{ij}_{\mathrm{can}}$, leaving $h^{\mathrm{TT}}_{ij}$ and $\pi ^{ij\mathrm TT}_{\mathrm{can}}$ as the final degrees of freedom of the gravitational field. Notice that our gauge condition $\pi ^{ii}_{\mathrm{can}} = 0$ deviates from the original ADM one $\pi ^{ii} = 0$ by spin corrections (which enter at 5PN order). The final fully reduced action reads,

7.57 $$\begin{aligned} W = \frac{c^4}{16\pi G}\int \mathrm {d}^4 x\, \pi ^{ij\mathrm TT}_{\mathrm{can}} h^{\mathrm{TT}}_{ij,0} + \int \mathrm {d}t \bigg [ P_i \dot{{\hat{z}}}^i + \frac{1}{2} {\hat{S}}_{(i)(j)} \dot{\hat{\theta }}^{(i)(j)} - E \bigg ]. \end{aligned}$$

The dynamics is completely described by the ADM energy *E*, which is the total Hamiltonian ($E=H$) once it is expressed in terms of the canonical variables. This Hamiltonian can be written as the volume integral

7.58 $$\begin{aligned} H[{\hat{z}}^i, P_i, {\hat{S}}_{(i)(j)}, h^{\mathrm{TT}}_{ij}, \pi ^{ij\mathrm TT}_{\mathrm{can}}] = -\frac{c^4}{2\pi G} \int \mathrm {d}^3 x\, \varDelta \varPsi \left[ {\hat{z}}^i, P_i, {\hat{S}}_{(i)(j)}, h^{\mathrm{TT}}_{ij}, \pi ^{ij\mathrm TT}_{\mathrm{can}}\right] . \end{aligned}$$

The equal-time Poisson bracket relations take the standard form,

7.59 $$\begin{aligned}&\{ {\hat{z}}^i, P_j\} = \delta _{ij}, \quad \{{{{\hat{S}}}}_{(i)}, {{{\hat{S}}}}_{(j)}\} = \epsilon _{ijk} {{{\hat{S}}}}_{(k)}, \end{aligned}$$

7.60 $$\begin{aligned}&\{h^{\mathrm{TT}}_{ij}(\mathbf{x},t), \pi ^{kl\mathrm TT}_{\mathrm{can}}(\mathbf{x}',t)\} = \frac{16\pi G}{c^3} \delta ^{\mathrm{TT}kl}_{ij}\delta (\mathbf{x} - \mathbf{x}'), \end{aligned}$$

zero otherwise, where ${{{\hat{S}}}}_{(i)}=\frac{1}{2}\epsilon _{(i)(j)(k)}{{{\hat{S}}}}_{(j)(k)}$, $\epsilon _{(i)(j)(k)}=\epsilon _{ijk}=(i-j)(j-k)(k-i)/2$, and $\delta ^{\mathrm{TT}ij}_{mn}$ is the TT-projection operator, see, e.g., Steinhoff et al. (2008c). Though the commutation relations (7.59) and (7.60) are sufficient for the variables on which the Hamiltonian (7.58) depends on, for completeness we add the non-trivial ones needed when a Hamiltonian, besides ${\hat{S}}_{(i)(j)}$, also depends on the 3-dimensional rotation matrix $\hat{\varLambda }_{[i](j)}$ (“angle” variables). They read

7.61 $$\begin{aligned} \{\hat{\varLambda }_{[i](j)}, {\hat{S}}_{(k)(l)}\} = \hat{\varLambda }_{[i](k)} \delta _{lj} - \hat{\varLambda }_{[i](l)}\delta _{kj}. \end{aligned}$$

The angular velocity tensor $\hat{\varOmega }^{(i)(j)}$, the Legendre dual to ${\hat{S}}_{(i)(j)}$, i.e. $\hat{\varOmega }^{(i)(j)} = 2 \partial H/\partial {\hat{S}}_{(i)(j)}$, is defined by $\hat{\varOmega }^{(i)(j)} = \delta \hat{\theta }^{(i)(j)}/\mathrm {d}t = \hat{\varLambda }_{[k]}^{~(i)}\dot{\hat{\varLambda }}^{[k](j)}$, and the time derivative of the spin tensor thus reads

7.62 $$\begin{aligned} \dot{{\hat{S}}}_{(i)(j)} = 2{\hat{S}}_{(k)[(i)}\varOmega _{(j)](k)} + \hat{\varLambda }^{[k](j)}\frac{\partial H}{\partial \hat{\varLambda }^{[k](i)}} - \hat{\varLambda }^{[k](i)}\frac{\partial H}{\partial \hat{\varLambda }^{[k](j)}}. \end{aligned}$$

The Hamiltonian *H* of Eq. (7.58) generates the time evolution in the reduced matter+field phase space. Generalization and application to many-body systems is quite straightforward, see Steinhoff et al. (2008c). The total linear ($P_i^{\mathrm{tot}}$) and angular ($J_{ij}^{\mathrm{tot}}$) momenta take the forms (particle labels are denoted by *a*),

7.63 $$\begin{aligned} P_i^{\mathrm{tot}}&= \sum _a P_{ai} - \frac{c^3}{16\pi G} \int \mathrm {d}^3x \, \pi _{\mathrm{can}}^{kl\mathrm TT} h^{\mathrm{TT}}_{kl,i}, \end{aligned}$$

7.64 $$\begin{aligned} J_{ij}^{\mathrm{tot}}&= \sum _a \left( {\hat{z}}_a^i P_{aj} - {\hat{z}}_a^j P_{ai} + {\hat{S}}_{a(i)(j)}\right) - \frac{c^3}{8\pi G} \int \mathrm {d}^3x\, \left( \pi _{\mathrm{can}}^{ik\mathrm TT} h^{\mathrm{TT}}_{kj}- \pi _{\mathrm{can}}^{jk\mathrm TT} h^{\mathrm{TT}}_{ki} \right) \nonumber \\&\quad - \frac{c^3}{16\pi G} \int \mathrm {d}^3x \left( x^i \pi _{\mathrm{can}}^{kl\mathrm TT} h^{\mathrm{TT}}_{kl,j} - x^j \pi _{\mathrm{can}}^{kl\mathrm TT} h^{\mathrm{TT}}_{kl,i} \right) , \end{aligned}$$

and are obtained from the reduced action in the standard Noether manner.

### Spin-squared dynamics

For the construction of the spin-squared terms we resort to the well-known stress–energy tensor for pole–dipole particles but augmented for quadrupolar terms. The stress–energy tensor density then reads (Steinhoff et al. 2008b)

7.65 $$\begin{aligned} \sqrt{-g}\,T^{\mu \nu } = \int \mathrm {d}\tau \bigg [ t^{\mu \nu } \delta _{(4)} + ( t^{\mu \nu \alpha } \delta _{(4)} )_{||\alpha } + ( t^{\mu \nu \alpha \beta } \delta _{(4)} )_{||\alpha \beta } \bigg ]. \end{aligned}$$

The quantities $t^{\mu \nu \dots }=t^{\nu \mu \dots }$ only depend on the four-velocity $u^{\mu }\equiv \mathrm {d}z^{\mu }/{\mathrm {d}\tau }$, where $z^{\mu }(\tau )$ is the parametrization of the worldline in terms of its proper time $\tau$, and on the spin and quadrupole tensors. Notice that, in general, the quadrupole expressions include not only the mass-quadrupole moment, but also the current-quadrupole moment and the stress-quadrupole moment (see, e.g., Steinhoff and Puetzfeld 2010). For the pole–dipole particle $t^{\mu \nu \alpha \beta }$ is zero. In contrast to the stress–energy tensor of pole–dipole particles, the Riemann tensor shows up at the quadrupolar level. However, the source terms of the constraints,

7.66 $$\begin{aligned} {\gamma }^\frac{1}{2} T^{\mu \nu }n_\mu n_\nu = {\mathcal {H}}^{\mathrm{matter}}, \quad -{\gamma }^\frac{1}{2}T^{\;\mu }_i n_\mu = {\mathcal {H}}^{\mathrm{matter}}_i, \end{aligned}$$

at the approximation considered here, do not include the Riemann tensor.

Regarding rotating black holes, the mass-quadrupole tensor $Q^{ij}_1$ of object 1 is given by Steinhoff et al. (2008b) (also see, e.g., Thorne 1980; Damour 2001)

7.67 $$\begin{aligned} m_1 c^2 Q^{ij}_1 \equiv \gamma ^{ik}\gamma ^{jl}\gamma ^{mn}{\hat{S}}_{1km}{\hat{S}}_{1nl} + \frac{2}{3}{} \mathbf{S}^2_1\gamma ^{ij}, \end{aligned}$$

where $\mathbf {S}_1=(S_{1(i)})$ is the three-dimensional Euclidean spin vector related to a spin tensor ${\hat{S}}_{1ij}$ with the help of a dreibein $e_{i(j)}$ by ${\hat{S}}_{1ij}=e_{i(k)}e_{j(l)}\epsilon _{klm}S_{1(m)}$. The quantity $\mathbf{S}_1^2$ is conserved in time,

7.68 $$\begin{aligned} 2\mathbf{S}_1^2 = \gamma ^{ik} \gamma ^{jl} {\hat{S}}_{1ij} {\hat{S}}_{1kl} = \text{ const }. \end{aligned}$$

The source terms of the constraints in the static case (independent from the linear momenta $P_i$ of the objects, what means taking $P_i=0$, but $p_i\ne 0$) read

7.69 $$\begin{aligned} \mathcal {H}^{\mathrm{matter}}_{S_1^2,\,\mathrm{static}}&= c_1 \left( c^2 Q^{ij}_1 \delta _1 \right) _{; ij} + \frac{1}{8 m_1} \gamma _{mn} \gamma ^{pj} \gamma ^{ql} {\gamma ^{mi}}_{,p} {\gamma ^{nk}}_{,q} {\hat{S}}_{1 ij} {\hat{S}}_{1 kl} \delta _1 \nonumber \\&\quad + \frac{1}{4m_1} \left( \gamma ^{ij} \gamma ^{mn} \gamma ^{kl}_{~~,m} {\hat{S}}_{1 ln} {\hat{S}}_{1 jk} \delta _1 \right) _{,i}, \end{aligned}$$

7.70 $$\begin{aligned} \mathcal {H}_{i \,\mathrm static}^{\mathrm{matter}}&= \frac{1}{2} \left( \gamma ^{mk}{{{\hat{S}}}}_{ik} \delta \right) _{,m} + \mathcal {O}{({\hat{S}}^3)}. \end{aligned}$$

The $c_1$ is some constant that must be fixed by additional considerations, like matching to the Kerr metric. The noncovariant terms are due to the transition from three-dimensional covariant linear momentum $p_i$ to canonical linear momentum $P_i$ given by [cf. Eq. (4.24) in Steinhoff et al. 2008c or Eq. (7.32) above]

7.71 $$\begin{aligned} p_i = P_i - \frac{1}{2} \gamma _{ij} \gamma ^{lm} \gamma ^{jk}_{~~,m} {\hat{S}}_{kl} + \mathcal {O}(P^2) + \mathcal {O}({\hat{S}}^2). \end{aligned}$$

Thus the source terms are indeed covariant when the point-mass and linear-in-spin terms depending on the (noncovariant) canonical linear momentum are added, cf. Eqs. (7.26) and (7.27).

The simple structure of the $Q_1^{ij}$ term in Eq. (7.69) is just the structure of minimal coupling of the Minkowski space mass-quadrupole term to gravity. As shown by Steinhoff et al. (2008b), the most general ansatz for the spin-squared coupling including the three-dimensional Ricci tensor reduces to the shown term. Here we may argue that the correct limit to flat space on the one side and the occurrence of a multiplicative second delta-function through the Ricci tensor from the spinning “point” particle on the other side makes the ansatz unique. A deeper analysis of the structure of nonlinear-in-spin couplings can be found in, e.g., Levi and Steinhoff (2015).

### Approximate Hamiltonians for spinning binaries

All the approximate Hamiltonians presented in this subsection have been derived or rederived in recent papers by one of the authors and his collaborators employing canonical formalisms presented in Sects. 7.1 and 7.2 (Damour et al. 2008c; Steinhoff et al. 2008c, b). They are two-point-particle Hamiltonians, which can be used to approximately model binaries made of spinning black holes. For the rest of this section, canonical variables (which are arguments of displayed Hamiltonians) are not hatted any further. We use $a,b=1,2$ as the bodies labels, and for $a \ne b$ we define $r_{ab}\mathbf {n}_{ab}\equiv \mathbf {x}_{a}-\mathbf {x}_{b}$ with $\mathbf {n}_{ab}^2=1$.

The Hamiltonian of leading-order (LO) spin–orbit coupling reads (let us note that in the following $\mathbf {p}_a$ will denote the canonical linear momenta)

7.72 $$\begin{aligned} H_{\text {SO}}^{\text {LO}} = \sum _a \sum _{b \ne a} \frac{G}{c^2r_{a b}^2} (\mathbf {S}_a \times \mathbf {n}_{ab}) \cdot \left( \frac{3 m_b}{2 m_a} \mathbf {p}_a - 2 \mathbf {p}_b \right) , \end{aligned}$$

and the one of leading-order spin(1)–spin(2) coupling is given by

7.73 $$\begin{aligned} H_{{S_1S_2}}^{\text {LO}} = \sum _a \sum _{b \ne a} \frac{G}{2 c^2r_{a b}^3} \left[ 3(\mathbf {S}_a \cdot \mathbf {n}_{ab})(\mathbf {S}_b \cdot \mathbf {n}_{ab}) - (\mathbf {S}_a \cdot \mathbf {S}_b) \right] . \end{aligned}$$

The more complicated Hamiltonian is the one with spin-squared terms because it relates to the rotational deformation of spinning black holes. To leading order, say for spin(1), it reads

7.74 $$\begin{aligned} H_{{S_1^2}}^{\text {LO}} = \frac{Gm_2}{2 c^2m_1r_{12}^3} \left[ 3 (\mathbf {S}_1 \cdot \mathbf {n}_{12}) (\mathbf {S}_1 \cdot \mathbf {n}_{12}) - (\mathbf {S}_1\cdot \mathbf {S}_1) \right] . \end{aligned}$$

The LO spin–orbit and spin(*a*)–spin(*b*) centre-of-mass vectors take the form

7.75 $$\begin{aligned} \mathbf {G}_{\text {SO}}^{\text {LO}} = \sum _a \frac{1}{2c^2 m_a} (\mathbf {p}_a \times \mathbf {S}_a), \quad \mathbf {G}_{{S_1S_2}}^{\text {LO}} =0, \quad \mathbf {G}_{{S^2_1}}^{\text {LO}} = 0. \end{aligned}$$

The LO spin Hamiltonians have been applied to studies of binary pulsar and solar system dynamics, including satellites on orbits around the Earth (see, e.g., Barker and O’Connell 1979; Schäfer 2004). Another application to the coalescence of spinning binary black holes via the effective-one-body approach is given in Damour (2001). The LO spin dynamics was analysed for black holes and other extended objects in external fields by D’Eath (1975a) and Thorne and Hartle (1985), and for binary black holes in the slow-motion limit by D’Eath (1975b). In Barausse et al. (2009, 2012) the spinning test-particle dynamics in the Kerr metric has been explored at LO within Hamiltonian formalism based on Dirac brackets. In the article Kidder (1995) the LO spin–orbit and spin1–spin2 dynamics for compact binaries is treated in full detail, even including their influence on the gravitational waves and the related gravitational damping, particularly the quasi-circular inspiraling and the recoil of the linear momentum from the LO spin coupling was obtained.

The Hamiltonian of the next-to-leading-order (NLO) spin–orbit coupling reads

7.76 $$\begin{aligned} H_{\text {SO}}^{\text {NLO}}&= -G\frac{((\mathbf {p}_1 \times \mathbf {S}_1) \cdot \mathbf {n}_{1 2})}{c^4r_{12}^2} \Bigg ( \frac{5 m_2 \mathbf {p}_1^2}{8 m_1^3} + \frac{3 ((\mathbf {p}_1\cdot \mathbf {p}_2)+(\mathbf {n}_{12}\cdot \mathbf {p}_1)(\mathbf {n}_{12}\cdot \mathbf {p}_2))}{4 m_1^2} \nonumber \\&\quad - \frac{3(\mathbf {p}_2^2- 2(\mathbf {n}_{12}\cdot \mathbf {p}_2)^2)}{4 m_1 m_2} \Bigg ) + G \frac{((\mathbf {p}_1 \times \mathbf {S}_1) \cdot \mathbf {p}_2)}{c^4r_{12}^2} \left( \frac{2 (\mathbf {n}_{12}\cdot \mathbf {p}_2)}{m_1 m_2} - \frac{3 (\mathbf {n}_{12}\cdot \mathbf {p}_1)}{4 m_1^2} \right) \nonumber \\&\quad + G\frac{((\mathbf {p}_2 \times \mathbf {S}_1) \cdot \mathbf {n}_{1 2})}{c^4r_{12}^2} \frac{(\mathbf {p}_1\cdot \mathbf {p}_2)+ 3 (\mathbf {n}_{12}\cdot \mathbf {p}_1)(\mathbf {n}_{12}\cdot \mathbf {p}_2)}{m_1 m_2} \nonumber \\&\quad - G^2\frac{((\mathbf {p}_1 \times \mathbf {S}_1) \cdot \mathbf {n}_{1 2})}{c^4r_{12}^3} \left( \frac{11 m_2}{2} + \frac{5 m_2^2}{m_1}\right) \nonumber \\&\quad + G^2 \frac{((\mathbf {p}_2 \times \mathbf {S}_1) \cdot \mathbf {n}_{1 2})}{c^4r_{12}^3} \left( 6 m_1 + \frac{15 m_2}{2}\right) + (1 \leftrightarrow 2). \end{aligned}$$

This Hamiltonian was derived by Damour et al. (2008c). The equivalent derivation of the NLO spin–orbit effects in two-body equations of motion was done in harmonic coordinates by Blanchet et al. (2006, 2007, 2010).

The NLO spin(1)–spin(2) Hamiltonian is given by

7.77 $$\begin{aligned} H_{{S_1S_2}}^{\text {NLO}}&= \frac{G}{2 m_1 m_2 c^4r_{12}^3} \Big [6 ((\mathbf {p}_2 \times \mathbf {S}_1) \cdot \mathbf {n}_{1 2})((\mathbf {p}_1 \times \mathbf {S}_2) \cdot \mathbf {n}_{1 2})\nonumber \\&\quad + \frac{3}{2} ((\mathbf {p}_1 \times \mathbf {S}_1) \cdot \mathbf {n}_{1 2})((\mathbf {p}_2 \times \mathbf {S}_2) \cdot \mathbf {n}_{1 2})\nonumber \\&\quad - 15 (\mathbf {S}_1 \cdot \mathbf {n}_{1 2})(\mathbf {S}_2 \cdot \mathbf {n}_{1 2})(\mathbf {n}_{12}\cdot \mathbf {p}_1)(\mathbf {n}_{12}\cdot \mathbf {p}_2)\nonumber \\&\quad - 3 (\mathbf {S}_1 \cdot \mathbf {n}_{1 2})(\mathbf {S}_2 \cdot \mathbf {n}_{1 2})(\mathbf {p}_1\cdot \mathbf {p}_2)+ 3 (\mathbf {S}_1 \cdot \mathbf {p}_2)(\mathbf {S}_2 \cdot \mathbf {n}_{1 2})(\mathbf {n}_{12}\cdot \mathbf {p}_1)\nonumber \\&\quad + 3 (\mathbf {S}_2 \cdot \mathbf {p}_1)(\mathbf {S}_1 \cdot \mathbf {n}_{1 2})(\mathbf {n}_{12}\cdot \mathbf {p}_2)+ 3 (\mathbf {S}_1 \cdot \mathbf {p}_1)(\mathbf {S}_2 \cdot \mathbf {n}_{1 2})(\mathbf {n}_{12}\cdot \mathbf {p}_2)\nonumber \\&\quad + 3 (\mathbf {S}_2 \cdot \mathbf {p}_2)(\mathbf {S}_1 \cdot \mathbf {n}_{1 2})(\mathbf {n}_{12}\cdot \mathbf {p}_1)- 3 (\mathbf {S}_1 \cdot \mathbf {S}_2)(\mathbf {n}_{12}\cdot \mathbf {p}_1)(\mathbf {n}_{12}\cdot \mathbf {p}_2)\nonumber \\&\quad + (\mathbf {S}_1 \cdot \mathbf {p}_1)(\mathbf {S}_2 \cdot \mathbf {p}_2)- \frac{1}{2} (\mathbf {S}_1 \cdot \mathbf {p}_2)(\mathbf {S}_2 \cdot \mathbf {p}_1)+ \frac{1}{2} (\mathbf {S}_1 \cdot \mathbf {S}_2)(\mathbf {p}_1\cdot \mathbf {p}_2)\Big ] \nonumber \\&\quad + \frac{3}{2 m_1^2 r_{12}^3} \Big [-((\mathbf {p}_1 \times \mathbf {S}_1) \cdot \mathbf {n}_{1 2})((\mathbf {p}_1 \times \mathbf {S}_2) \cdot \mathbf {n}_{1 2})\nonumber \\&\quad + (\mathbf {S}_1 \cdot \mathbf {S}_2)(\mathbf {n}_{12}\cdot \mathbf {p}_1)^2 - (\mathbf {S}_1 \cdot \mathbf {n}_{1 2})(\mathbf {S}_2 \cdot \mathbf {p}_1)(\mathbf {n}_{12}\cdot \mathbf {p}_1)\Big ] \nonumber \\&\quad + \frac{3}{2 m_2^2 r_{12}^3} \Big [-((\mathbf {p}_2 \times \mathbf {S}_2) \cdot \mathbf {n}_{1 2})((\mathbf {p}_2 \times \mathbf {S}_1) \cdot \mathbf {n}_{1 2})\nonumber \\&\quad + (\mathbf {S}_1 \cdot \mathbf {S}_2)(\mathbf {n}_{12}\cdot \mathbf {p}_2)^2 - (\mathbf {S}_2 \cdot \mathbf {n}_{1 2})(\mathbf {S}_1 \cdot \mathbf {p}_2)(\mathbf {n}_{12}\cdot \mathbf {p}_2)\Big ] \nonumber \\&\quad + \frac{6(m_1 + m_2 )G^2}{c^4r_{12}^4} [(\mathbf {S}_1 \cdot \mathbf {S}_2)- 2(\mathbf {S}_1 \cdot \mathbf {n}_{1 2})(\mathbf {S}_2 \cdot \mathbf {n}_{1 2})]. \end{aligned}$$

The calculation of the LO and NLO $S_1^2$-Hamiltonians needs employing the source terms (7.69)–(7.70). In the case of polar–dipolar–quadrupolar particles which are to model spinning black holes, $Q^{ij}_1$ is the quadrupole tensor of the black hole 1 resulting from its rotational deformation and the value of the constant $c_1$ is fixed by matching to the test-body Hamiltonian in a Kerr background: $c_1=-1/2$. Additionally one has to use the Poincaré algebra for unique fixation of all coefficients in momentum-dependent part of the Hamiltonian. The NLO $S_1^2$-Hamiltonian was presented for the first time by Steinhoff et al. (2008b).^9^ It reads

7.78 $$\begin{aligned} H_{S_1^2}^{\text {NLO}}&= \frac{G}{c^4r_{12}^3}\bigg \{ \frac{m_{2}}{m_{1}^3} \bigg [ \frac{1}{4}\left( {{\mathbf {p}}}_{1}\cdot {{\mathbf {S}}}_{1}\right) ^2 + \frac{3}{8}\left( {{\mathbf {p}}}_{1}\cdot {{\mathbf {n}}}_{12}\right) ^{2}{{\mathbf {S}}}_{1}^{2} - \frac{3}{8} {{\mathbf {p}}}_{1}^{2}\left( {{\mathbf {S}}}_{1}\cdot {{\mathbf {n}}}_{12}\right) ^2 \nonumber \\&\quad - \frac{3}{4} \left( {{\mathbf {p}}}_{1}\cdot {{\mathbf {n}}}_{12}\right) \left( {{\mathbf {S}}}_{1}\cdot {{\mathbf {n}}}_{12}\right) \left( {{\mathbf {p}}}_{1}\cdot {{\mathbf {S}}}_{1}\right) \bigg ] + \frac{3}{4m_{1}m_{2}}\Big [3{{\mathbf {p}}}_{2}^{2}\left( {{\mathbf {S}}}_{1}\cdot {{\mathbf {n}}}_{12}\right) ^2 \nonumber \\&\quad - {{\mathbf {p}}}_{2}^{2}{{\mathbf {S}}}_{1}^{2}\Big ] + \frac{1}{m_1^2} \bigg [ \frac{3}{4}\left( {{\mathbf {p}}}_{1}\cdot {{\mathbf {p}}}_{2}\right) {{\mathbf {S}}}_{1}^2 -\frac{9}{4}\left( {{\mathbf {p}}}_{1}\cdot {{\mathbf {p}}}_{2}\right) \left( {{\mathbf {S}}}_{1} \cdot {{\mathbf {n}}}_{12}\right) ^2 \nonumber \\&\quad - \frac{3}{2}\left( {{\mathbf {p}}}_{1}\cdot {{\mathbf {n}}}_{12}\right) \left( {{\mathbf {p}}}_{2}\cdot {{\mathbf {S}}}_{1}\right) \left( {{\mathbf {S}}}_{1}\cdot {{\mathbf {n}}}_{12}\right) +3\left( {{\mathbf {p}}}_{2}\cdot {{\mathbf {n}}}_{12}\right) \left( {{\mathbf {p}}}_{1} \cdot {{\mathbf {S}}}_{1}\right) \left( {{\mathbf {S}}}_{1}\cdot {{\mathbf {n}}}_{12}\right) \nonumber \\&\quad + \frac{3}{4}\left( {{\mathbf {p}}}_{1}\cdot {{\mathbf {n}}}_{12}\right) \left( {{\mathbf {p}}}_{2} \cdot {{\mathbf {n}}}_{12}\right) {{\mathbf {S}}}_{1}^2 -\frac{15}{4}\left( {{\mathbf {p}}}_{1}\cdot {{\mathbf {n}}}_{12}\right) \left( {{\mathbf {p}}}_{2}\cdot {{\mathbf {n}}}_{12}\right) \left( {{\mathbf {S}}}_{1}\cdot {{\mathbf {n}}}_{12}\right) ^2 \bigg ] \bigg \} \nonumber \\&\quad - \frac{G^2 m_2}{2 c^4r_{12}^4} \bigg [9({{\mathbf {S}}}_1 \cdot {{\mathbf {n}}}_{12})^2 - 5 {{\mathbf {S}}}_1^2 + \frac{14 m_2}{m_1} ({{\mathbf {S}}}_1 \cdot {{\mathbf {n}}}_{12})^2 - \frac{6 m_2}{m_1}{{\mathbf {S}}}_1^2 \bigg ]. \end{aligned}$$

The spin precession equations corresponding to the Hamiltonians $H^{\text {NLO}}_{S_1S_2}$ and $H_{S_1^2}^{\text {NLO}}$ have been calculated also by Porto and Rothstein (2008a, b),^10^ respectively, where the first paper [Porto and Rothstein 2008b has benefited from Steinhoff et al. (2008a)] when forgotten terms from spin-induced velocity corrections in the LO spin–orbit coupling could be identified (so-called subleading corrections), see Eq. (57) in Porto and Rothstein (2008b).

The NLO spin–orbit and spin(*a*)–spin(*b*) centre-of-mass vectors take the form

7.79 $$\begin{aligned} \mathbf {G}_{\text {SO}}^{\text {NLO}}&= -\sum _a \frac{\mathbf {p}_a^2}{8 c^4m_a^3} (\mathbf {p}_a \times \mathbf {S}_a) \nonumber \\&\quad + \sum _a \sum _{b \ne a} \frac{G m_b}{4 c^4m_a r_{ab}} \bigg \{ [(\mathbf {p}_a\times \mathbf {S}_a)\cdot \mathbf {n}_{ab}] \frac{5\mathbf {x}_a+\mathbf {x}_b}{r_{ab}} - 5 (\mathbf {p}_a \times \mathbf {S}_a) \bigg \} \nonumber \\&\quad + \sum _a \sum _{b \ne a} \frac{G}{c^4 r_{ab}} \bigg \{ \frac{3}{2} (\mathbf {p}_b \times \mathbf {S}_a) - \frac{1}{2} (\mathbf {n}_{ab} \times \mathbf {S}_a) (\mathbf {p}_b \cdot \mathbf {n}_{ab}) \nonumber \\&\quad - [(\mathbf {p}_a \times \mathbf {S}_a) \cdot \mathbf {n}_{ab}] \frac{\mathbf {x}_a+\mathbf {x}_b}{r_{ab}} \bigg \}, \end{aligned}$$

7.80 $$\begin{aligned} \mathbf {G}_{{S_1S_2}}^{\text {NLO}}&= \frac{G}{2c^4} \sum _a \sum _{b \ne a} \bigg \{ \left[ 3(\mathbf {S}_{a}\cdot \mathbf {n}_{ab})(\mathbf {S}_{b}\cdot \mathbf {n}_{ab}) -(\mathbf {S}_{a}\cdot \mathbf {S}_{b})\right] \frac{\mathbf {x}_{a}}{r_{ab}^3} + (\mathbf {S}_{b}\cdot \mathbf {n}_{ab}) \frac{\mathbf {S}_{a}}{r_{ab}^2} \bigg \}, \end{aligned}$$

7.81 $$\begin{aligned} \mathbf {G}_{S_1^2}^{\text {NLO}}&= \frac{2Gm_2}{c^4m_{1}} \bigg \{ \frac{3\left( \mathbf {S}_{1}\cdot \mathbf {n}_{12}\right) ^2}{8r_{12}^3}\left( \mathbf {x}_{1}+\mathbf {x}_{2}\right) +\frac{\mathbf {S}_{1}^2}{8r_{12}^3} \left( 3\mathbf {x}_{1}-5\mathbf {x}_{2}\right) -\frac{\left( \mathbf {S}_{1}\cdot \mathbf {n}_{12}\right) \mathbf {S}_1}{r_{12}^2}\bigg \}. \end{aligned}$$

We can sum up all centre-of-mass vectors displayed in this subsection in the following equation:

7.82 $$\begin{aligned} \mathbf {G} = \mathbf {G}_{\text {N}} + \mathbf {G}_{\text {1PN}} + \mathbf {G}_{\text {2PN}} + \mathbf {G}_{\text {3PN}} + \mathbf {G}_{\text {4PN}} + \mathbf {G}_{\text {SO}}^{\text {LO}} + \mathbf {G}_{\text {SO}}^{\text {NLO}} + \mathbf {G}_{S_{1}S_{2}}^{\text {NLO}} + \mathbf {G}_{S_{1}^2}^{\text {NLO}} + \mathbf {G}_{S_{2}^2}^{\text {NLO}}, \end{aligned}$$

where $\mathbf {G}_{\text {N}}$ up to $\mathbf {G}_{\text {4PN}}$ represent the pure orbital contributions, which do not depend on spin variables [the explicit formulae for them one can find in Jaranowski and Schäfer (2015)]. The last term in Eq. (7.82) can be obtained from the second last one by means of the exchange $(1 \leftrightarrow 2)$ of the bodies’ labels.

The currently known conservative two-point-particle Hamiltonians, modeling binaries made of spinning black holes, can be summarized as follows:

7.83 $$\begin{aligned} H&= H_{\text {N}} + H_{\text {1PN}} + H_{\text {2PN}} + H_{\text {3PN}} + H_{\text {4PN}} \nonumber \\&\quad + H_{\text {SO}}^{\text {LO}} + H^{\text {LO}}_{S_{1}S_{2}} + H^{\text {LO}}_{S_{1}^2} + H^{\text {LO}}_{S_{2}^2} \nonumber \\&\quad + H_{\text {SO}}^{\text {NLO}} + H^{\text {NLO}}_{S_{1}S_{2}} + H^{\text {NLO}}_{S_{1}^2} + H^{\text {NLO}}_{S_{2}^2} \nonumber \\&\quad + H_{\text {SO}}^{\text {NNLO}} + H^{\text {NNLO}}_{S_{1}S_{2}} + H^{\text {NNLO}}_{S_{1}^2} + H^{\text {NNLO}}_{S_{2}^2} \nonumber \\&\quad + H_{p_{1}S_{2}^3}+H_{p_{2}S_{1}^3} + H_{p_{1}S_{1}^3} + H_{p_{2}S_{2}^3} \nonumber \\&\quad + H_{p_{1}S_{1}S_{2}^2} + H_{p_{2}S_{2}S_{1}^2} + H_{p_{1}S_{2}S_{1}^2} + H_{p_{2}S_{1}S_{2}^2} \nonumber \\&\quad + H_{S_{1}^2S_{2}^2} + H_{S_{1} S_{2}^3} + H_{S_{2}S_{1}^3} + H_{S_{1}^4} + H_{S_{2}^4}, \end{aligned}$$

where the first line comprises pure orbital, i.e., spin-independent, Hamiltonians. The Hamiltonians from the second and the third line are explicitly given above. The NNLO spin–orbit $H_{\text {SO}}^{\text {NNLO}}$ and spin1–spin2 $H^{\text {NNLO}}_{S_{1}S_{2}}$ Hamiltonians were obtained by Hartung et al. (2013), their explicit forms can be found in Appendix D. Levi and Steinhoff (2016a) derived, applying the EFT method to extended bodies, the NNLO spin-squared Hamiltonians $H^{\text {NNLO}}_{S_{1}^2}$ and $H^{\text {NNLO}}_{S_{2}^2}$; we do not display them explicitly, as their derivation is not yet fully confirmed. All the Hamiltonians with labels containing linear momenta $p_1$ or $p_2$ and those quartic in the spins were derived by Hergt and Schäfer (2008a, b) with the aid of approximate ADMTT coordinates of the Kerr metric and application of the Poincaré algebra.^11^ Their generalizations to general extended objects were achieved by Levi and Steinhoff (2015), where also for the first time the Hamiltonians $H_{S_1^4}$ and $H_{S_2^4}$ were obtained (correcting Hergt and Schäfer 2008a). All the Hamiltonians cubic and quartic in the spins and displayed in Eq. (7.83) are explicitly given in Appendix D. Notice that not all Hamiltonians from Eq. (7.83) are necessarily given in the ADM gauge, because any use of the equations of motion in their derivation has changed gauge. E.g., for spinless particles the highest conservative Hamiltonian in ADM gauge is $H_{\text {2PN}}$.

For completeness we also give the spin-squared Hamiltonians for neutron stars through next-to-leading order (Porto and Rothstein 2008a, 2010a; Hergt et al. 2010). They depend on the quantity $C_Q$, which parametrizes quadrupolar deformation effects induced by spins. The LO Hamiltonian reads (cf., e.g., Barker and O’Connell 1979)

7.84 $$\begin{aligned} H_{S_{1}^2\text {(NS)}}^{\text {LO}} = \frac{Gm_{1}m_{2}}{2r_{12}^3} C_{Q_1} \left( 3\frac{(\mathbf {S}_1 \cdot \mathbf {n}_{1 2})^2}{m_{1}^2}-\frac{\mathbf {S}_1^2}{m_{1}^2}\right) . \end{aligned}$$

The NLO Hamiltonian equals

7.85 $$\begin{aligned} H_{S_{1}^2\text {(NS)}}^{\text {NLO}}&= \frac{G}{r_{12}^3} \Bigg [\frac{m_{2}}{m_{1}^3}\Bigg ( \left( -\frac{21}{8}+\frac{9}{4}C_{Q_1}\right) \mathbf {p}_1^2({\mathbf {S}}_1 \cdot {\mathbf {n}}_{1 2})^2 +\left( \frac{3}{2}C_{Q_1}-\frac{5}{4}\right) ({\mathbf {S}}_1 \cdot \mathbf {p}_1)^2 \nonumber \\&\quad +\left( \frac{15}{4}-\frac{9}{2}C_{Q_1}\right) (\mathbf {p}_1 \cdot {\mathbf {n}}_{1 2})({\mathbf {S}}_1 \cdot {\mathbf {n}}_{1 2})({\mathbf {S}}_1 \cdot \mathbf {p}_1)\nonumber \\&\quad +\left( -\frac{9}{8}+\frac{3}{2}C_{Q_1}\right) (\mathbf {p}_1 \cdot {\mathbf {n}}_{1 2})^2{\mathbf {S}}_1^2+\left( \frac{5}{4}-\frac{5}{4}C_{Q_1}\right) \mathbf {p}_1^2{\mathbf {S}}_1^2\Bigg ) \nonumber \\&\quad +\frac{1}{m_{1}^2} \Bigg (-\frac{15}{4}C_{Q_1}(\mathbf {p}_1 \cdot {\mathbf {n}}_{1 2})(\mathbf {p}_2 \cdot {\mathbf {n}}_{1 2})({\mathbf {S}}_1 \cdot {\mathbf {n}}_{1 2})^2 \nonumber \\&\quad +\left( 3-\frac{21}{4}C_{Q_1}\right) (\mathbf {p}_1\cdot \mathbf {p}_2)({\mathbf {S}}_1 \cdot {\mathbf {n}}_{1 2})^2 \nonumber \\&\quad +\left( -\frac{3}{2}+\frac{9}{2}C_{Q_1}\right) (\mathbf {p}_2 \cdot {\mathbf {n}}_{1 2})({\mathbf {S}}_1 \cdot {\mathbf {n}}_{1 2})({\mathbf {S}}_1 \cdot \mathbf {p}_1)\nonumber \\&\quad +\left( -3+\frac{3}{2}C_{Q_1}\right) (\mathbf {p}_1 \cdot {\mathbf {n}}_{1 2})({\mathbf {S}}_1 \cdot {\mathbf {n}}_{1 2})({\mathbf {S}}_1 \cdot \mathbf {p}_2)\nonumber \\&\quad +\left( \frac{3}{2}-\frac{3}{2}C_{Q_1}\right) ({\mathbf {S}}_1 \cdot \mathbf {p}_1)({\mathbf {S}}_1 \cdot \mathbf {p}_2)\nonumber \\&\quad +\left( \frac{3}{2}-\frac{3}{4}C_{Q_1}\right) (\mathbf {p}_1 \cdot {\mathbf {n}}_{1 2})(\mathbf {p}_2 \cdot {\mathbf {n}}_{1 2}){\mathbf {S}}_1^2\nonumber \\&\quad +\left( -\frac{3}{2}+\frac{9}{4}C_{Q_1}\right) (\mathbf {p}_1\cdot \mathbf {p}_2){\mathbf {S}}_1^2\Bigg ) +\frac{C_{Q_1}}{m_{1}m_{2}}\Big (\frac{9}{4}\mathbf {p}_2^2({\mathbf {S}}_1 \cdot {\mathbf {n}}_{1 2})^2-\frac{3}{4}\mathbf {p}_2^2{\mathbf {S}}_1^2\Big )\Bigg ] \nonumber \\&\quad +\frac{G^2m_{2}}{r_{12}^4}\Bigg [\left( 2+\frac{1}{2}C_{Q_1}+\frac{m_{2}}{m_{1}}\big (1+2C_{Q_1}\big )\right) {\mathbf {S}}_1^2\nonumber \\&\quad +\left( -3-\frac{3}{2}C_{Q_1}-\frac{m_{2}}{m_{1}}\big (1+6C_{Q_1}\big )\right) ({\mathbf {S}}_1 \cdot {\mathbf {n}}_{1 2})^2\Bigg ]. \end{aligned}$$

This Hamiltonian for $C_{Q_1}=1$ agrees with that given in Eq. (7.78) describing black-hole binaries. It has been derived fully correctly for the first time by Porto and Rothstein (2010a) using the EFT method. Shortly afterwards, an independent calculation by Hergt et al. (2010), in part based on the Eqs. (7.69) and (7.70) including (7.67), has confirmed the result.

The radiation-reaction (or dissipative) Hamiltonians for leading-order spin–orbit and spin1–spin2 couplings are derived by Steinhoff and Wang (2010) and Wang et al. (2011). All the known dissipative Hamiltonians can thus be summarized as

7.86 $$\begin{aligned} H^{\mathrm{diss}} = H_{\mathrm{2.5PN}} + H_{\mathrm{3.5PN}} + H^{\text {LO diss}}_{\mathrm{SO}} + H^{\text {LO diss}}_{S_1S_2}, \end{aligned}$$

where $H_{\mathrm{2.5PN}}$ and $H_{\mathrm{2.5PN}}$ are spin-independent (purely orbital) dissipative Hamiltonians. The leading-order Hamiltonian $H_{\mathrm{2.5PN}}$ is given in Eq. (6.79) for two-point-mass and in Appendix E for many-point-mass systems, and the next-to-leading-order Hamiltonian $H_{\mathrm{3.5PN}}$ is explicitly given in the Appendix E (also for many-point-mass systems). The spin-dependent dissipative Hamiltonians $H^{\text {LO diss}}_{\mathrm{SO}}$ and $H^{\text {LO diss}}_{S_1S_2}$ can be read off from the Hamiltonian $H^{\text {spin}}_{\text {3.5PN}}$ given in the Appendix E (we keep here the notation of the Hamiltonian used by Wang et al. 2011, which indicates spin corrections to the spinless 3.5PN dynamics).

## A Hamiltonian dynamics of ideal fluids in Newtonian gravity

In the Newtonian theory the equations for gravitating ideal fluids are usually given in the following form:

(i) The equation for the conservation of mass,^12^
  A.1 $$\begin{aligned} \partial _t \varrho _* + \text{ div }( \varrho _* \mathbf{v}) = 0, \end{aligned}$$
  where $\varrho _*$ is the mass density and $\mathbf{v}= (v^i)$ is the velocity field of the fluid.
(ii) The equations of motion,
  A.2 $$\begin{aligned} \varrho _* \partial _t \mathbf{v} + \frac{\varrho _*}{2}~ \text{ grad }~\mathbf{v}^2 - \varrho _*~ \mathbf{v} \times \text{ curl }~\mathbf{v} = - \mathrm {grad}\, p + \varrho _*~\text{ grad }~U, \end{aligned}$$
  where *p* is the pressure in the fluid and *U* the gravitational potential.
(iii) The equation of state,
  A.3 $$\begin{aligned} \epsilon = \epsilon (\varrho _*, s) \quad \text{ with } \quad \mathrm {d}\epsilon = h \mathrm {d}\varrho _* + \varrho _*T \mathrm {d}s, \quad \text{ or } \quad \mathrm {d}p = \varrho _* \mathrm {d}h - \varrho _*T \mathrm {d}s, \end{aligned}$$
  with the temperature *T*, the internal energy density $\epsilon$ and the specific enthalpy *h*.
(iv) The conservation law for the specific entropy *s* along the flow lines,
  A.4 $$\begin{aligned} \partial _t s + \mathbf{v}\cdot \text{ grad }~s = 0. \end{aligned}$$
(v) The Newtonian gravitational field equation,
  A.5 $$\begin{aligned} \varDelta U = - 4\pi G \varrho _*, \end{aligned}$$
  where $\varDelta$ is the Laplacian. The gravitational potential hereof reads
  A.6 $$\begin{aligned} U(\mathbf{x},t) = G \int \mathrm {d}^3\mathbf{x}' \frac{ \varrho _*(\mathbf{x}',t)}{|\mathbf{x}-\mathbf{x}'|}. \end{aligned}$$

Within the Hamilton framework the equations of motion are obtained from the relation $\partial _t A(\mathbf{x},t)=\{A(\mathbf{x},t),H\}$, valid for any function $A(\mathbf{x},t)$ living in phase space, i.e. built out of the fundamental variables $\varrho _*$, $\pi _i$, and *s*, with the Hamiltonian given by $H=H[\varrho _*,\pi _i,s]$, where $\pi _i$ is the linear momentum density of the fluid (Holm 1985). The brackets $\{\cdot ,\cdot \}$ are called Lie–Poisson brackets. They may be defined by

A.7 $$\begin{aligned} \left\{ \int \mathrm {d}^3x\, \xi ^i \pi _i,\, F[\varrho _*,s,\pi _i] \right\} = \int \mathrm {d}^3x \left( \frac{\delta F}{\delta \varrho _*}{{\mathcal {L}}}_\xi \varrho _* + \frac{\delta F}{\delta s}{{\mathcal {L}}}_\xi s + \frac{\delta F}{\delta \pi _i}{{\mathcal {L}}}_\xi \pi _i \right) , \end{aligned}$$

where *F* is a functional of $\varrho _*$, *s*, and $\pi _i$, ${{\mathcal {L}}}_\xi$ denotes the Lie derivative along the vector field $\xi ^i$, and $\delta F/\delta (\cdots )$ are the Fréchet derivatives of the functional *F* [see, e.g., Appendix C of Blanchet et al. (1990) and references therein].

Explicitly, the equations in (i), (ii), and (iv) take the following Hamiltonian form [the equations in (iii) and (v) remain unchanged]:

(i) The mass conservation equation
  A.8 $$\begin{aligned} \frac{\partial \varrho _*}{\partial t} = -\partial _i \left( \frac{\delta H}{\delta \pi _i}\varrho _*\right) , \end{aligned}$$
  notice that $$\displaystyle v^i = \frac{\delta H}{\delta \pi _i}$$.
(ii) The equations of motion
  A.9 $$\begin{aligned} \frac{\partial \pi _i}{\partial t} = -\partial _j \left( \frac{\delta H}{\delta \pi _j}\pi _i\right) - \partial _i \left( \frac{\delta H}{\delta \pi _j}\right) \pi _j - \partial _i \left( \frac{\delta H}{\delta \varrho _*}\right) \varrho _* + \frac{\delta H}{\delta s} \partial _i s. \end{aligned}$$
(iv) The entropy conservation law
  A.10 $$\begin{aligned} \frac{\partial s}{\partial t} = -\frac{\delta H}{\delta \pi _i} \partial _i s. \end{aligned}$$

The following kinematical Lie–Poisson bracket relations between the fundamental variables are fulfilled:

A.11 $$\begin{aligned} \{\pi _i(\mathbf{x},t),\varrho _*(\mathbf{x}',t)\}&= \frac{\partial }{\partial x'^i}[\varrho _*(\mathbf{x}',t) \delta (\mathbf{x}-\mathbf{x}')], \end{aligned}$$

A.12 $$\begin{aligned} \{\pi _i(\mathbf{x},t),s(\mathbf{x}',t)\}&= \frac{\partial s(\mathbf{x}',t)}{\partial {x}'^i} \delta (\mathbf{x}-\mathbf{x}'), \end{aligned}$$

A.13 $$\begin{aligned} \{\pi _i(\mathbf{x},t),\pi _j(\mathbf{x}',t)\}&= \pi _i(\mathbf{x}',t)\frac{\partial }{\partial {x}'^j} \delta (\mathbf{x}-\mathbf{x}') - \pi _j(\mathbf{x},t)\frac{\partial }{\partial {x}^i} \delta (\mathbf{x}-\mathbf{x}'), \end{aligned}$$

and zero otherwise. More explicitly the Hamiltonian of the fluid takes the form,

A.14 $$\begin{aligned} H = \frac{1}{2}\int \mathrm {d}^3\mathbf{x} \frac{\pi _i\pi _i}{\varrho _*} - \frac{G}{2}\int \mathrm {d}^3\mathbf{x}\,\mathrm {d}^3\mathbf{x}'\,\frac{\varrho _*(\mathbf{x},t)\varrho _*(\mathbf{x}',t)}{|\mathbf{x}-\mathbf{x}'|} + \int \mathrm {d}^3\mathbf{x}\,\epsilon . \end{aligned}$$

For point masses, the momentum and mass densities are given by

A.15 $$\begin{aligned} \pi _i = \sum _a p_{ai} \delta (\mathbf{x}-\mathbf{x}_a), \qquad \varrho _* = \sum _a m_a \delta (\mathbf{x}-\mathbf{x}_{a}), \end{aligned}$$

and we have also $h=p=s=0$. The position and momentum variables fulfill the standard Poisson bracket relations,

A.16 $$\begin{aligned} \{x^i_a,p_{aj}\} = \delta _{ij}, \quad \quad \text{ zero } \text{ otherwise }, \end{aligned}$$

and the Hamiltonian results in

A.17 $$\begin{aligned} H = \frac{1}{2} \sum _a \frac{{\mathbf {p}}_a^2}{m_a} - \frac{G}{2} \sum _{a \ne b} \frac{m_am_b}{|\mathbf{x}_a-\mathbf{x}_b|}, \end{aligned}$$

where the internal and self-energy terms have been dropped (after performing a proper regularization, see Sect. 4.2 in our review).

Let us remark that for fluids a canonical formalism with standard Poisson brackets can be obtained with the transition to Lagrangian coordinates $b^A(x^i,t)$, such that $\partial _t b^A + \mathbf{v}\cdot \text{ grad }~b^A = 0$. Then,

A.18 $$\begin{aligned} p_A = b^i_A \pi _i \quad \text{ with } \quad b^i_A = \frac{\partial x^i}{\partial b^A}. \end{aligned}$$

The variables $b^A$ and $p_B$ are canonically conjugate to each other, i.e.

A.19 $$\begin{aligned} \{b^A(x^i,t),\, p_B(y^j,t)\} = \delta ^A_B(x^i-y^i). \end{aligned}$$

The mass density in Lagrangian coordinates, say $\mu (b^A,t)$, is defined by $\varrho _*\,\mathrm {d}^3x=\mu \,\mathrm {d}^3b$ and relates to the usual mass density as $\varrho _*=\mu (b^A,t)\det (b^B_j)$.

## B Hamiltonian dynamics of ideal fluids in GR

The general-relativistic equations governing the dynamics of gravitating ideal fluids are as follows (see, e.g., Holm 1985; Blanchet et al. 1990).

(i) The equation for the conservation of mass,
  B.1 $$\begin{aligned} \partial _{\mu }(\sqrt{-g}\varrho u^{\mu })=0 \quad \text{ or } \quad \partial _t \varrho _* + \text{ div }( \varrho _* \mathbf{v}) = 0, \end{aligned}$$
  where $\varrho$ denotes the proper rest-mass density and $u^{\mu }$ the four-velocity field of the fluid ($g_{\mu \nu }u^{\mu }u^{\nu } = -1$), $\varrho _* = \sqrt{-g} u^0 \varrho$ is the coordinate mass density and $\mathbf{v}$ the velocity field of the fluid, $v^i = c u^i/u^0$.
(ii) The equations of motion,
  B.2 $$\begin{aligned} \partial _{\mu }\left( \sqrt{-g}\,T^{\mu }_i\right) - \frac{1}{2}\, \sqrt{-g}\,T^{\mu \nu }\, \partial _i g_{\mu \nu } = 0, \end{aligned}$$
  where
  B.3 $$\begin{aligned} T^{\mu \nu } = \varrho (c^2 + h) u^{\mu }u^{\nu } + p g^{\mu \nu } \end{aligned}$$
  is the stress–energy tensor of the fluid with pressure *p* and specific enthalpy *h*.
(iii) The equation of state, using the energy density $e= \varrho (c^2 + h) - p$,
  B.4 $$\begin{aligned} e = e(\varrho ,s) \quad \text{ with } \quad \mathrm {d}e=(c^2+h)\mathrm {d}\varrho + \varrho T\mathrm {d}s \quad \text{ or } \quad \mathrm {d}p = \varrho \mathrm {d}h - \varrho T \mathrm {d}s. \end{aligned}$$
(iv) The conservation law for the specific entropy *s* along the flow lines,
  B.5 $$\begin{aligned} u^{\mu }\partial _{\mu }s=0 \quad \text{ or } \quad \partial _t s + \mathbf{v}\cdot \mathrm {grad}\,s = 0. \end{aligned}$$
(v) The Einsteinian field equations for gravitational potential (or metric) functions $g_{\mu \nu }$,
  B.6 $$\begin{aligned} R^{\mu \nu } = \frac{8\pi G}{c^4} \left( T^{\mu \nu } - \frac{1}{2}g^{\mu \nu }g_{\alpha \beta }T^{\alpha \beta }\right) . \end{aligned}$$

The variables of the canonical formalism get chosen to be

B.7 $$\begin{aligned} \varrho _* = \sqrt{-g} u^0 \varrho , \quad s, \quad \pi _i = \frac{1}{c} \sqrt{-g} T^0_i. \end{aligned}$$

They do fulfill the same (universal, free of spacetime metric) kinematical Lie–Poisson bracket relations as in the Newtonian theory (see Holm 1985 or also Blanchet et al. 1990),

B.8 $$\begin{aligned} \{\pi _i(\mathbf{x},t),\varrho _*(\mathbf{x}',t)\}&= \frac{\partial }{\partial x'^i}[\varrho _*(\mathbf{x}',t) \delta (\mathbf{x}-\mathbf{x}')], \end{aligned}$$

B.9 $$\begin{aligned} \{\pi _i(\mathbf{x},t),s(\mathbf{x}',t)\}&= \frac{\partial s(\mathbf{x}',t)}{\partial x'^i} \delta (\mathbf{x}-\mathbf{x}'), \end{aligned}$$

B.10 $$\begin{aligned} \{\pi _i(\mathbf{x},t),\pi _j(\mathbf{x}',t)\}&= \pi _i(\mathbf{x}',t)\frac{\partial }{\partial x'^j} \delta (\mathbf{x}-\mathbf{x}') - \pi _j(\mathbf{x},t)\frac{\partial }{\partial x^i} \delta (\mathbf{x}-\mathbf{x}'). \end{aligned}$$

Written as Hamiltonian equations of motion, i.e. $\partial _t A(\mathbf{x},t) = \{A(\mathbf{x},t), H\}$, the equations in (i), (ii), and (iv) take the following form [the equations in (iii) and (v) remain unchanged]:

(i) The mass conservation equation
  B.11 $$\begin{aligned} \frac{\partial \varrho _*}{\partial t} = -\partial _i \left( \frac{\delta H}{\delta \pi _i}\varrho _*\right) , \end{aligned}$$
  notice $$\displaystyle v^i = \frac{\delta H}{\delta \pi _i}$$.
(ii) The equations of motion
  B.12 $$\begin{aligned} \frac{\partial \pi _i}{\partial t} = -\partial _j \left( \frac{\delta H}{\delta \pi _j}\pi _i\right) - \partial _i \left( \frac{\delta H}{\delta \pi _j}\right) \pi _j - \partial _i \left( \frac{\delta H}{\delta \varrho _*}\right) \varrho _* + \frac{\delta H}{\delta s} \partial _i s. \end{aligned}$$
(iv) The entropy conservation law
  B.13 $$\begin{aligned} \frac{\partial s}{\partial t} = -\frac{\delta H}{\delta \pi _i} \partial _i s, \end{aligned}$$
  where the Hamiltonian functional is given by $H=H[\varrho _*,\pi _i,s]$, see Holm (1985).

Point-mass systems fulfill

B.14 $$\begin{aligned} h = p = s = 0, \end{aligned}$$

(just as for dust) and the momentum and mass densities read

B.15 $$\begin{aligned} \pi _i = \sum _a p_{ai} \delta (\mathbf{x}-\mathbf{x}_a), \quad \quad \varrho _* = \sum _a m_a \delta (\mathbf{x}-\mathbf{x}_{a}), \quad \quad v^i_a = \frac{\mathrm {d}x^i_a}{\mathrm {d}t}. \end{aligned}$$

The position and momentum variables again fulfill the standard Poisson bracket relations,

B.16 $$\begin{aligned} \{x^i_a,p_{aj}\} = \delta _{ij}, \qquad \text{ zero } \text{ otherwise }. \end{aligned}$$

Hereof the standard Hamilton equations are recovered,

B.17 $$\begin{aligned} \frac{\mathrm {d}p_{ai}}{\mathrm {d}t} = -\frac{\partial H}{\partial x^i_a}, \quad \frac{\mathrm {d}x^i_a}{\mathrm {d}t} = \frac{\partial H}{\partial p_{ai}}. \end{aligned}$$

Remarkably, the difference to the Newtonian theory solely results from the Hamiltonian, so the difference between GR and the Newtonian theory is essentially a dynamical and not a kinematical one. This statement refers to the matter only and not to the gravitational field. The latter is much more complicated in GR, dynamically and kinematically as well.

## C 4PN-accurate generators of Poincaré symmetry for two-point-mass systems

Generators of Poincaré symmetry for two-point-mass systems are realized as functions on the two-body phase-space $(\mathbf {x}_1,\mathbf {x}_2,{\mathbf {p}}_1,{\mathbf {p}}_2)$. In the $3+1$ splitting the 10 generators are: Hamiltonian *H*, linear momentum $P^i$, angular momentum $J^i$, and centre-of-energy vector $G^i$ (related to boost vector $K^i$ through $K^i=G^i-tP^i$). They all fulfill the Poincaré algebra relations (3.35)–(3.40). In this appendix we show 4PN-accurate formulae for these generators derived within the ADM formalism (see Bernard et al. 2018 for recent derivation of corresponding and equivalent formulae for integrals of motion in harmonic coordinates).

The gauge fixing used in the ADM formalism manifestly respects the Euclidean group (which means that the Hamiltonian *H* is translationally and rotationally invariant), therefore the generators $P^i$ and $J^i$ are simply realized as

C.1 $$\begin{aligned} P^i ({\mathbf {x}}_a,{\mathbf {p}}_a) = \sum _a p_{ai}, \quad J^i ({\mathbf {x}}_a,{\mathbf {p}}_a) = \sum _a \varepsilon _{ik\ell } \, x_a^k \, p_{a\ell }. \end{aligned}$$

These formula are exact (i.e., valid at all PN orders).

The 4PN-accurate conservative Hamiltonian $H_{\le \text {4PN}}$ is the sum of local and nonlocal-in-time parts,

C.2 $$\begin{aligned} H_{\le \text {4PN}}[{\mathbf {x}}_a,{\mathbf {p}}_a] = H_{\le \text {4PN}}^\text {local}({\mathbf {x}}_a,{\mathbf {p}}_a) + H_\text {4PN}^\text {nonlocal}[{\mathbf {x}}_a,{\mathbf {p}}_a], \end{aligned}$$

where the nonlocal-in-time piece equals

C.3 $$\begin{aligned} H_{\text {4PN}}^{\text {nonlocal}}[{\mathbf {x}}_a,{\mathbf {p}}_a] = -\frac{1}{5}\frac{G^2M}{c^8} \dddot{I}_{\!ij}(t) \times {\mathrm {Pf}}_{2r_{12}/c} \int _{-\infty }^{+\infty } \frac{\mathrm {d}\tau }{\vert \tau \vert } \dddot{I}_{\!ij}(t+\tau ). \end{aligned}$$

The third time derivative of $I_{ij}$, after replacing all time derivatives of ${\mathbf {x}}_a$ by using the Newtonian equations of motion, can be written as

C.4 $$\begin{aligned} \dddot{I}_{\!ij}&= -\frac{2Gm_1m_2}{r_{12}^2} \left\{ 4 n^{\langle i}_{12} \left( \frac{p_{1j\rangle }}{m_1}-\frac{p_{2j\rangle }}{m_2}\right) - 3 \left( \frac{(\mathbf {n}_{12}\cdot \mathbf {p}_1)}{m_1}-\frac{(\mathbf {n}_{12}\cdot \mathbf {p}_2)}{m_2}\right) n^{\langle i}_{12}n^{j\rangle }_{12} \right\} \nonumber \\&= -\frac{2Gm_1m_2}{r_{12}^3} \left\{ 4 x^{\langle i}_{12} v_{12}^{j \rangle } - \frac{3}{r_{12}} (\mathbf {n}_{12}\cdot \mathbf {v}_{12})x^{\langle i}_{12} x_{12}^{j \rangle }\right\} , \end{aligned}$$

where the relative velocity $\mathbf {v}_{12}\equiv {\mathbf {p}}_1/m_1-{\mathbf {p}}_2/m_2$ ($\langle \cdots \rangle$ denotes a symmetric tracefree projection). This formula is valid in an arbitrary reference frame and it is obviously Galileo-invariant. Consequently the nonlocal-in-time Hamiltonian (C.3) is Galileo-invariant as well. The local part of the 4PN-accurate Hamiltonian reads

C.5 $$\begin{aligned} H_{\le \text {4PN}}^\text {local}({\mathbf {x}}_a,{\mathbf {p}}_a)&= Mc^2 + H_\text {N}({\mathbf {x}}_a,{\mathbf {p}}_a) + H_\text {1PN}({\mathbf {x}}_a,{\mathbf {p}}_a) + H_\text {2PN}({\mathbf {x}}_a,{\mathbf {p}}_a) \nonumber \\&\quad + H_\text {3PN}({\mathbf {x}}_a,{\mathbf {p}}_a) + H_\text {4PN}^\text {local}({\mathbf {x}}_a,{\mathbf {p}}_a). \end{aligned}$$

The Hamiltonians $H_\text {N}$ to $H_\text {3PN}$ in generic, i.e. noncentre-of-mass, reference frame, are equal to [the operation “$+\big (1\leftrightarrow 2\big )$” used below denotes the addition for each term, including the ones which are symmetric under the exchange of body labels, of another term obtained by the label permutation $1\leftrightarrow 2$]

C.6 $$\begin{aligned} H_{\text {N}}({\mathbf {x}}_a,{\mathbf {p}}_a)= & {} \frac{{\mathbf {p}}_1^2}{2m_1} - \frac{G m_1 m_2}{2r_{12}} + \big (1\leftrightarrow 2\big ), \end{aligned}$$

C.7 $$\begin{aligned} c^2\,H_{\text {1PN}}({\mathbf {x}}_a,{\mathbf {p}}_a)= & {} - \frac{(\mathbf {p}_1^2)^2}{8m_1^3} + \frac{Gm_1m_2}{4r_{12}} \Bigg ( -\frac{6\mathbf {p}_1^2}{m_1^2} + \frac{7(\mathbf{p}_1\cdot \mathbf{p}_2)}{m_1m_2} \nonumber \\&\quad +\, \frac{(\mathbf {n}_{12}\cdot \mathbf {p}_1)(\mathbf{n}_{12}\cdot \mathbf{p}_2)}{m_1m_2} \Bigg ) + \frac{G^2m_1^2m_2}{2r_{12}^2} + \big (1\leftrightarrow 2\big ), \end{aligned}$$

C.8 $$\begin{aligned} c^4\,H_{\text {2PN}}({\mathbf {x}}_a,{\mathbf {p}}_a)= & {} \frac{(\mathbf {p}_1^2)^3}{16m_1^5} + \frac{Gm_1m_2}{8r_{12}} \Bigg ( 5\,\frac{(\mathbf {p}_1^2)^2}{m_1^4} - \frac{11}{2}\frac{\mathbf {p}_1^2\,\mathbf{p}_2^2}{m_1^2m_2^2} - \frac{(\mathbf{p}_1\cdot \mathbf{p}_2)^2}{m_1^2m_2^2} \nonumber \\&\quad +\, 5\,\frac{\mathbf {p}_1^2\,(\mathbf{n}_{12}\cdot \mathbf{p}_2)^2}{m_1^2m_2^2} - 6\,\frac{(\mathbf{p}_1\cdot \mathbf{p}_2)\,(\mathbf {n}_{12}\cdot \mathbf {p}_1)(\mathbf{n}_{12}\cdot \mathbf{p}_2)}{m_1^2m_2^2} \nonumber \\&\quad -\, \frac{3}{2}\frac{(\mathbf {n}_{12}\cdot \mathbf {p}_1)^2(\mathbf{n}_{12}\cdot \mathbf{p}_2)^2}{m_1^2m_2^2} \Bigg ) + \frac{G^2m_1m_2}{4r_{12}^2} \Bigg ( m_2\Bigg (10\frac{\mathbf {p}_1^2}{m_1^2}+19\frac{\mathbf{p}_2^2}{m_2^2}\Bigg ) \nonumber \\&\quad -\, \frac{1}{2}(m_1+m_2)\frac{27\,(\mathbf{p}_1\cdot \mathbf{p}_2)+6\,(\mathbf {n}_{12}\cdot \mathbf {p}_1)(\mathbf{n}_{12}\cdot \mathbf{p}_2)}{m_1m_2} \Bigg ) \nonumber \\&\quad -\, \frac{G^3m_1m_2 (m_1^2+5m_1m_2+m_2^2)}{8r_{12}^3} + \big (1\leftrightarrow 2\big ), \end{aligned}$$

$$\begin{aligned} c^6\,H_{\text {3PN}}({\mathbf {x}}_a,{\mathbf {p}}_a)= & {} - \frac{5(\mathbf {p}_1^2)^4}{128m_1^7} + \frac{Gm_1m_2}{32r_{12}} \Bigg ( - \frac{14(\mathbf {p}_1^2)^3}{m_1^6} + \frac{6\mathbf {p}_1^2(\mathbf {n}_{12}\cdot \mathbf {p}_1)^2(\mathbf{n}_{12}\cdot \mathbf{p}_2)^2}{m_1^4m_2^2} \\&+\, 4\frac{\big ((\mathbf{p}_1\cdot \mathbf{p}_2)^2+4\,\mathbf {p}_1^2\,\mathbf{p}_2^2\big )\mathbf {p}_1^2}{m_1^4m_2^2} - 10\frac{\big (\mathbf {p}_1^2\,(\mathbf{n}_{12}\cdot \mathbf{p}_2)^2+\mathbf{p}_2^2\,(\mathbf {n}_{12}\cdot \mathbf {p}_1)^2\big )\mathbf {p}_1^2}{m_1^4m_2^2} \\&+\, 24\frac{\mathbf {p}_1^2\,(\mathbf{p}_1\cdot \mathbf{p}_2)(\mathbf {n}_{12}\cdot \mathbf {p}_1)(\mathbf{n}_{12}\cdot \mathbf{p}_2)}{m_1^4m_2^2} + 2\frac{\mathbf {p}_1^2\,(\mathbf{p}_1\cdot \mathbf{p}_2)(\mathbf{n}_{12}\cdot \mathbf{p}_2)^2}{m_1^3m_2^3} \\&+\, \frac{\big (7\,\mathbf {p}_1^2\,\mathbf{p}_2^2-10\,(\mathbf{p}_1\cdot \mathbf{p}_2)^2\big )(\mathbf {n}_{12}\cdot \mathbf {p}_1)(\mathbf{n}_{12}\cdot \mathbf{p}_2)}{m_1^3m_2^3} \\&+\, \frac{\big (\mathbf {p}_1^2\,\mathbf{p}_2^2-2\,(\mathbf{p}_1\cdot \mathbf{p}_2)^2\big )(\mathbf{p}_1\cdot \mathbf{p}_2)}{m_1^3m_2^3} + 15\frac{(\mathbf{p}_1\cdot \mathbf{p}_2)(\mathbf {n}_{12}\cdot \mathbf {p}_1)^2(\mathbf{n}_{12}\cdot \mathbf{p}_2)^2}{m_1^3m_2^3} \\&-\, 18\frac{\mathbf {p}_1^2\,(\mathbf {n}_{12}\cdot \mathbf {p}_1)(\mathbf{n}_{12}\cdot \mathbf{p}_2)^3}{m_1^3m_2^3} + 5\frac{(\mathbf {n}_{12}\cdot \mathbf {p}_1)^3(\mathbf{n}_{12}\cdot \mathbf{p}_2)^3}{m_1^3m_2^3} \Bigg ) \\&+\, \frac{G^2m_1m_2}{r_{12}^2} \Bigg ( \frac{1}{16}(m_1-27m_2)\frac{(\mathbf {p}_1^2)^2}{m_1^4} - \frac{115}{16}m_1\frac{\mathbf {p}_1^2\,(\mathbf{p}_1\cdot \mathbf{p}_2)}{m_1^3m_2} \end{aligned}$$

C.9 $$\begin{aligned}&+\, \frac{1}{48}m_2\frac{25\,(\mathbf{p}_1\cdot \mathbf{p}_2)^2+371\,\mathbf {p}_1^2\,\mathbf{p}_2^2}{m_1^2 m_2^2} + \frac{17}{16}\frac{\mathbf {p}_1^2(\mathbf {n}_{12}\cdot \mathbf {p}_1)^2}{m_1^3} + \frac{5}{12}\frac{(\mathbf {n}_{12}\cdot \mathbf {p}_1)^4}{m_1^3} \nonumber \\&-\, \frac{1}{8}m_1 \frac{\big (15\,\mathbf {p}_1^2\,(\mathbf{n}_{12}\cdot \mathbf{p}_2)+11\,(\mathbf{p}_1\cdot \mathbf{p}_2)\,(\mathbf {n}_{12}\cdot \mathbf {p}_1)\big )(\mathbf {n}_{12}\cdot \mathbf {p}_1)}{m_1^3 m_2}\nonumber \\&-\, \frac{3}{2}m_1\frac{(\mathbf {n}_{12}\cdot \mathbf {p}_1)^3(\mathbf{n}_{12}\cdot \mathbf{p}_2)}{m_1^3m_2} + \frac{125}{12}m_2\frac{(\mathbf{p}_1\cdot \mathbf{p}_2)\,(\mathbf {n}_{12}\cdot \mathbf {p}_1)(\mathbf{n}_{12}\cdot \mathbf{p}_2)}{m_1^2m_2^2}\nonumber \\&+\, \frac{10}{3}m_2\frac{(\mathbf {n}_{12}\cdot \mathbf {p}_1)^2(\mathbf{n}_{12}\cdot \mathbf{p}_2)^2}{m_1^2m_2^2} - \frac{1}{48} (220 m_1 + 193 m_2) \frac{\mathbf {p}_1^2(\mathbf{n}_{12}\cdot \mathbf{p}_2)^2}{m_1^2 m_2^2} \Bigg ) \nonumber \\&+\, \frac{G^3m_1m_2}{r_{12}^3} \Bigg ( -\frac{1}{48} \bigg (425\,m_1^2+\Big (473-\frac{3}{4}\pi ^2\Big )m_1m_2+150\,m_2^2\bigg ) \frac{\mathbf {p}_1^2}{m_1^2} \nonumber \\&+\, \frac{1}{16} \bigg (77(m_1^2+m_2^2)+\Big (143-\frac{1}{4}\pi ^2\Big )m_1m_2\bigg ) \frac{(\mathbf{p}_1\cdot \mathbf{p}_2)}{m_1m_2} \nonumber \\&+\, \frac{1}{16} \bigg (20\,m_1^2-\Big (43+\frac{3}{4}\pi ^2\Big )m_1m_2\bigg ) \frac{(\mathbf {n}_{12}\cdot \mathbf {p}_1)^2}{m_1^2} \nonumber \\&+\, \frac{1}{16} \bigg (21(m_1^2+m_2^2)+\Big (119+\frac{3}{4}\pi ^2\Big )m_1m_2\bigg ) \frac{(\mathbf {n}_{12}\cdot \mathbf {p}_1)(\mathbf{n}_{12}\cdot \mathbf{p}_2)}{m_1m_2} \Bigg ) \nonumber \\&+\, \frac{G^4m_1m_2^3}{8r_{12}^4} \Bigg ( \bigg (\frac{227}{3}-\frac{21}{4}\pi ^2\bigg )m_1+m_2 \Bigg ) + \big (1\leftrightarrow 2\big ). \end{aligned}$$

The formula for the Hamiltonian $H_\text {4PN}^\text {local}$ is large, therefore we display it in smaller pieces:

C.10 $$\begin{aligned} c^8\,H_\text {4PN}^\text {local}({\mathbf {x}}_a,{\mathbf {p}}_a)&= \frac{7 (\mathbf {p}_1^2)^5}{256 m_1^9} + \frac{G m_1 m_2}{r_{12}} H_{48}({\mathbf {x}}_a,{\mathbf {p}}_a) + \frac{G^2 m_1 m_2}{r_{12}^2} m_1\,H_{46}({\mathbf {x}}_a,{\mathbf {p}}_a) \nonumber \\&\quad + \frac{G^3 m_1 m_2}{r_{12}^3} \Big (m_1^2\,H_{441}({\mathbf {x}}_a,{\mathbf {p}}_a) + m_1 m_2\,H_{442}({\mathbf {x}}_a,{\mathbf {p}}_a) \Big ) \nonumber \\&\quad + \frac{G^4 m_1 m_2}{r_{12}^4} \Big (m_1^3\,H_{421}({\mathbf {x}}_a,{\mathbf {p}}_a) + m_1^2 m_2\,H_{422}({\mathbf {x}}_a,{\mathbf {p}}_a)\Big ) \nonumber \\&\quad + \frac{G^5 m_1 m_2}{r_{12}^5} H_{40}({\mathbf {x}}_a,{\mathbf {p}}_a) + \big (1\leftrightarrow 2\big ), \end{aligned}$$

where

C.11 $$\begin{aligned}&H_{48}({\mathbf {x}}_a,{\mathbf {p}}_a) = \frac{45 (\mathbf {p}_1^2)^4}{128 m_1^8} -\frac{9 (\mathbf {n}_{12}\cdot \mathbf {p}_1)^2 (\mathbf {n}_{12}\cdot \mathbf {p}_2)^2 (\mathbf {p}_1^2)^2}{64 m_1^6 m_2^2} +\frac{15 (\mathbf {n}_{12}\cdot \mathbf {p}_2)^2 (\mathbf {p}_1^2)^3}{64m_1^6 m_2^2} \nonumber \\&\quad -\frac{9 (\mathbf {n}_{12}\cdot \mathbf {p}_1)(\mathbf {n}_{12}\cdot \mathbf {p}_2)(\mathbf {p}_1^2)^2 (\mathbf {p}_1\cdot \mathbf {p}_2)}{16 m_1^6 m_2^2} -\frac{3 (\mathbf {p}_1^2)^2 (\mathbf {p}_1\cdot \mathbf {p}_2)^2}{32m_1^6 m_2^2} \nonumber \\&\quad +\frac{15 (\mathbf {n}_{12}\cdot \mathbf {p}_1)^2 (\mathbf {p}_1^2)^2 \mathbf {p}_2^2}{64 m_1^6 m_2^2} -\frac{21 (\mathbf {p}_1^2)^3 \mathbf {p}_2^2}{64 m_1^6m_2^2} -\frac{35 (\mathbf {n}_{12}\cdot \mathbf {p}_1)^5 (\mathbf {n}_{12}\cdot \mathbf {p}_2)^3}{256 m_1^5 m_2^3} \nonumber \\&\quad +\frac{25 (\mathbf {n}_{12}\cdot \mathbf {p}_1)^3 (\mathbf {n}_{12}\cdot \mathbf {p}_2)^3 \mathbf {p}_1^2}{128 m_1^5m_2^3} +\frac{33 (\mathbf {n}_{12}\cdot \mathbf {p}_1)(\mathbf {n}_{12}\cdot \mathbf {p}_2)^3 (\mathbf {p}_1^2)^2}{256 m_1^5 m_2^3} \nonumber \\&\quad -\frac{85 (\mathbf {n}_{12}\cdot \mathbf {p}_1)^4 (\mathbf {n}_{12}\cdot \mathbf {p}_2)^2 (\mathbf {p}_1\cdot \mathbf {p}_2)}{256 m_1^5m_2^3} -\frac{45 (\mathbf {n}_{12}\cdot \mathbf {p}_1)^2 (\mathbf {n}_{12}\cdot \mathbf {p}_2)^2 \mathbf {p}_1^2(\mathbf {p}_1\cdot \mathbf {p}_2)}{128 m_1^5 m_2^3} \nonumber \\&\quad -\frac{(\mathbf {n}_{12}\cdot \mathbf {p}_2)^2 (\mathbf {p}_1^2)^2 (\mathbf {p}_1\cdot \mathbf {p}_2)}{256m_1^5 m_2^3} +\frac{25 (\mathbf {n}_{12}\cdot \mathbf {p}_1)^3 (\mathbf {n}_{12}\cdot \mathbf {p}_2)(\mathbf {p}_1\cdot \mathbf {p}_2)^2}{64 m_1^5 m_2^3} \nonumber \\&\quad +\frac{7 (\mathbf {n}_{12}\cdot \mathbf {p}_1)(\mathbf {n}_{12}\cdot \mathbf {p}_2)\mathbf {p}_1^2(\mathbf {p}_1\cdot \mathbf {p}_2)^2}{64 m_1^5 m_2^3} -\frac{3 (\mathbf {n}_{12}\cdot \mathbf {p}_1)^2 (\mathbf {p}_1\cdot \mathbf {p}_2)^3}{64 m_1^5 m_2^3} +\frac{3 \mathbf {p}_1^2(\mathbf {p}_1\cdot \mathbf {p}_2)^3}{64 m_1^5m_2^3} \nonumber \\&\quad +\frac{55 (\mathbf {n}_{12}\cdot \mathbf {p}_1)^5 (\mathbf {n}_{12}\cdot \mathbf {p}_2)\mathbf {p}_2^2}{256 m_1^5 m_2^3} -\frac{7 (\mathbf {n}_{12}\cdot \mathbf {p}_1)^3 (\mathbf {n}_{12}\cdot \mathbf {p}_2)\mathbf {p}_1^2\mathbf {p}_2^2}{128m_1^5 m_2^3} \nonumber \\&\quad -\frac{25 (\mathbf {n}_{12}\cdot \mathbf {p}_1)(\mathbf {n}_{12}\cdot \mathbf {p}_2)(\mathbf {p}_1^2)^2 \mathbf {p}_2^2}{256 m_1^5 m_2^3} -\frac{23 (\mathbf {n}_{12}\cdot \mathbf {p}_1)^4 (\mathbf {p}_1\cdot \mathbf {p}_2)\mathbf {p}_2^2}{256 m_1^5 m_2^3} \nonumber \\&\quad +\frac{7 (\mathbf {n}_{12}\cdot \mathbf {p}_1)^2 \mathbf {p}_1^2(\mathbf {p}_1\cdot \mathbf {p}_2)\mathbf {p}_2^2}{128 m_1^5 m_2^3} -\frac{7 (\mathbf {p}_1^2)^2(\mathbf {p}_1\cdot \mathbf {p}_2)\mathbf {p}_2^2}{256 m_1^5 m_2^3} -\frac{5 (\mathbf {n}_{12}\cdot \mathbf {p}_1)^2 (\mathbf {n}_{12}\cdot \mathbf {p}_2)^4 \mathbf {p}_1^2}{64 m_1^4 m_2^4} \nonumber \\&\quad +\frac{7 (\mathbf {n}_{12}\cdot \mathbf {p}_2)^4(\mathbf {p}_1^2)^2}{64 m_1^4 m_2^4} -\frac{(\mathbf {n}_{12}\cdot \mathbf {p}_1)(\mathbf {n}_{12}\cdot \mathbf {p}_2)^3 \mathbf {p}_1^2(\mathbf {p}_1\cdot \mathbf {p}_2)}{4 m_1^4 m_2^4} \nonumber \\&\quad +\frac{(\mathbf {n}_{12}\cdot \mathbf {p}_2)^2 \mathbf {p}_1^2(\mathbf {p}_1\cdot \mathbf {p}_2)^2}{16 m_1^4 m_2^4} -\frac{5 (\mathbf {n}_{12}\cdot \mathbf {p}_1)^4 (\mathbf {n}_{12}\cdot \mathbf {p}_2)^2 \mathbf {p}_2^2}{64 m_1^4 m_2^4} \nonumber \\&\quad +\frac{21 (\mathbf {n}_{12}\cdot \mathbf {p}_1)^2 (\mathbf {n}_{12}\cdot \mathbf {p}_2)^2\mathbf {p}_1^2\mathbf {p}_2^2}{64 m_1^4 m_2^4} -\frac{3 (\mathbf {n}_{12}\cdot \mathbf {p}_2)^2 (\mathbf {p}_1^2)^2 \mathbf {p}_2^2}{32 m_1^4 m_2^4} \nonumber \\&\quad -\frac{(\mathbf {n}_{12}\cdot \mathbf {p}_1)^3(\mathbf {n}_{12}\cdot \mathbf {p}_2)(\mathbf {p}_1\cdot \mathbf {p}_2)\mathbf {p}_2^2}{4 m_1^4 m_2^4} +\frac{(\mathbf {n}_{12}\cdot \mathbf {p}_1)(\mathbf {n}_{12}\cdot \mathbf {p}_2)\mathbf {p}_1^2(\mathbf {p}_1\cdot \mathbf {p}_2)\mathbf {p}_2^2}{16 m_1^4m_2^4} \nonumber \\&\quad +\frac{(\mathbf {n}_{12}\cdot \mathbf {p}_1)^2 (\mathbf {p}_1\cdot \mathbf {p}_2)^2 \mathbf {p}_2^2}{16 m_1^4 m_2^4} -\frac{\mathbf {p}_1^2(\mathbf {p}_1\cdot \mathbf {p}_2)^2 \mathbf {p}_2^2}{32 m_1^4m_2^4} +\frac{7 (\mathbf {n}_{12}\cdot \mathbf {p}_1)^4 (\mathbf {p}_2^2)^2}{64 m_1^4 m_2^4} \nonumber \\&\quad -\frac{3 (\mathbf {n}_{12}\cdot \mathbf {p}_1)^2 \mathbf {p}_1^2(\mathbf {p}_2^2)^2}{32 m_1^4m_2^4} -\frac{7 (\mathbf {p}_1^2)^2 (\mathbf {p}_2^2)^2}{128 m_1^4 m_2^4}, \end{aligned}$$

C.12 $$\begin{aligned}&H_{46}({\mathbf {x}}_a,{\mathbf {p}}_a) = \frac{369 (\mathbf {n}_{12}\cdot \mathbf {p}_1)^6}{160 m_1^6} -\frac{889 (\mathbf {n}_{12}\cdot \mathbf {p}_1)^4 \mathbf {p}_1^2}{192 m_1^6} +\frac{49 (\mathbf {n}_{12}\cdot \mathbf {p}_1)^2 (\mathbf {p}_1^2)^2}{16 m_1^6} \nonumber \\&\quad -\frac{63(\mathbf {p}_1^2)^3}{64 m_1^6} -\frac{549 (\mathbf {n}_{12}\cdot \mathbf {p}_1)^5 (\mathbf {n}_{12}\cdot \mathbf {p}_2)}{128 m_1^5 m_2} +\frac{67 (\mathbf {n}_{12}\cdot \mathbf {p}_1)^3 (\mathbf {n}_{12}\cdot \mathbf {p}_2)\mathbf {p}_1^2}{16 m_1^5m_2} \nonumber \\&\quad -\frac{167 (\mathbf {n}_{12}\cdot \mathbf {p}_1)(\mathbf {n}_{12}\cdot \mathbf {p}_2)(\mathbf {p}_1^2)^2}{128 m_1^5 m_2} +\frac{1547 (\mathbf {n}_{12}\cdot \mathbf {p}_1)^4 (\mathbf {p}_1\cdot \mathbf {p}_2)}{256 m_1^5m_2} \nonumber \\&\quad -\frac{851 (\mathbf {n}_{12}\cdot \mathbf {p}_1)^2 \mathbf {p}_1^2(\mathbf {p}_1\cdot \mathbf {p}_2)}{128 m_1^5 m_2} +\frac{1099 (\mathbf {p}_1^2)^2 (\mathbf {p}_1\cdot \mathbf {p}_2)}{256 m_1^5 m_2} \nonumber \\&\quad +\frac{3263(\mathbf {n}_{12}\cdot \mathbf {p}_1)^4 (\mathbf {n}_{12}\cdot \mathbf {p}_2)^2}{1280 m_1^4 m_2^2} +\frac{1067 (\mathbf {n}_{12}\cdot \mathbf {p}_1)^2 (\mathbf {n}_{12}\cdot \mathbf {p}_2)^2 \mathbf {p}_1^2}{480 m_1^4 m_2^2} \nonumber \\&\quad -\frac{4567(\mathbf {n}_{12}\cdot \mathbf {p}_2)^2 (\mathbf {p}_1^2)^2}{3840 m_1^4 m_2^2} -\frac{3571 (\mathbf {n}_{12}\cdot \mathbf {p}_1)^3 (\mathbf {n}_{12}\cdot \mathbf {p}_2)(\mathbf {p}_1\cdot \mathbf {p}_2)}{320 m_1^4 m_2^2} \nonumber \\&\quad +\frac{3073(\mathbf {n}_{12}\cdot \mathbf {p}_1)(\mathbf {n}_{12}\cdot \mathbf {p}_2)\mathbf {p}_1^2(\mathbf {p}_1\cdot \mathbf {p}_2)}{480 m_1^4 m_2^2} +\frac{4349 (\mathbf {n}_{12}\cdot \mathbf {p}_1)^2 (\mathbf {p}_1\cdot \mathbf {p}_2)^2}{1280 m_1^4 m_2^2} \nonumber \\&\quad -\frac{3461\mathbf {p}_1^2(\mathbf {p}_1\cdot \mathbf {p}_2)^2}{3840 m_1^4 m_2^2} +\frac{1673 (\mathbf {n}_{12}\cdot \mathbf {p}_1)^4 \mathbf {p}_2^2}{1920 m_1^4 m_2^2} -\frac{1999 (\mathbf {n}_{12}\cdot \mathbf {p}_1)^2 \mathbf {p}_1^2\mathbf {p}_2^2}{3840 m_1^4 m_2^2} \nonumber \\&\quad +\frac{2081 (\mathbf {p}_1^2)^2 \mathbf {p}_2^2}{3840 m_1^4 m_2^2} -\frac{13 (\mathbf {n}_{12}\cdot \mathbf {p}_1)^3 (\mathbf {n}_{12}\cdot \mathbf {p}_2)^3}{8m_1^3 m_2^3} +\frac{191 (\mathbf {n}_{12}\cdot \mathbf {p}_1)(\mathbf {n}_{12}\cdot \mathbf {p}_2)^3 \mathbf {p}_1^2}{192 m_1^3 m_2^3} \nonumber \\&\quad -\frac{19 (\mathbf {n}_{12}\cdot \mathbf {p}_1)^2 (\mathbf {n}_{12}\cdot \mathbf {p}_2)^2 (\mathbf {p}_1\cdot \mathbf {p}_2)}{384m_1^3 m_2^3} -\frac{5 (\mathbf {n}_{12}\cdot \mathbf {p}_2)^2 \mathbf {p}_1^2(\mathbf {p}_1\cdot \mathbf {p}_2)}{384 m_1^3 m_2^3} \nonumber \\&\quad +\frac{11 (\mathbf {n}_{12}\cdot \mathbf {p}_1)(\mathbf {n}_{12}\cdot \mathbf {p}_2)(\mathbf {p}_1\cdot \mathbf {p}_2)^2}{192m_1^3 m_2^3} +\frac{77 (\mathbf {p}_1\cdot \mathbf {p}_2)^3}{96 m_1^3 m_2^3} \nonumber \\&\quad +\frac{233 (\mathbf {n}_{12}\cdot \mathbf {p}_1)^3 (\mathbf {n}_{12}\cdot \mathbf {p}_2)\mathbf {p}_2^2}{96 m_1^3m_2^3} -\frac{47 (\mathbf {n}_{12}\cdot \mathbf {p}_1)(\mathbf {n}_{12}\cdot \mathbf {p}_2)\mathbf {p}_1^2\mathbf {p}_2^2}{32 m_1^3 m_2^3} \nonumber \\&\quad +\frac{(\mathbf {n}_{12}\cdot \mathbf {p}_1)^2 (\mathbf {p}_1\cdot \mathbf {p}_2)\mathbf {p}_2^2}{384 m_1^3m_2^3} -\frac{185 \mathbf {p}_1^2(\mathbf {p}_1\cdot \mathbf {p}_2)\mathbf {p}_2^2}{384 m_1^3 m_2^3} -\frac{7 (\mathbf {n}_{12}\cdot \mathbf {p}_1)^2 (\mathbf {n}_{12}\cdot \mathbf {p}_2)^4}{4 m_1^2 m_2^4} \nonumber \\&\quad +\frac{7(\mathbf {n}_{12}\cdot \mathbf {p}_2)^4 \mathbf {p}_1^2}{4 m_1^2 m_2^4} -\frac{7 (\mathbf {n}_{12}\cdot \mathbf {p}_1)(\mathbf {n}_{12}\cdot \mathbf {p}_2)^3 (\mathbf {p}_1\cdot \mathbf {p}_2)}{2 m_1^2 m_2^4} \nonumber \\&\quad +\frac{21 (\mathbf {n}_{12}\cdot \mathbf {p}_2)^2(\mathbf {p}_1\cdot \mathbf {p}_2)^2}{16 m_1^2 m_2^4} +\frac{7 (\mathbf {n}_{12}\cdot \mathbf {p}_1)^2 (\mathbf {n}_{12}\cdot \mathbf {p}_2)^2 \mathbf {p}_2^2}{6 m_1^2 m_2^4} \nonumber \\&\quad +\frac{49 (\mathbf {n}_{12}\cdot \mathbf {p}_2)^2 \mathbf {p}_1^2\mathbf {p}_2^2}{48 m_1^2 m_2^4} -\frac{133 (\mathbf {n}_{12}\cdot \mathbf {p}_1)(\mathbf {n}_{12}\cdot \mathbf {p}_2)(\mathbf {p}_1\cdot \mathbf {p}_2)\mathbf {p}_2^2}{24 m_1^2 m_2^4} \nonumber \\&\quad -\frac{77 (\mathbf {p}_1\cdot \mathbf {p}_2)^2\mathbf {p}_2^2}{96 m_1^2 m_2^4} +\frac{197 (\mathbf {n}_{12}\cdot \mathbf {p}_1)^2 (\mathbf {p}_2^2)^2}{96 m_1^2 m_2^4} -\frac{173 \mathbf {p}_1^2(\mathbf {p}_2^2)^2}{48 m_1^2m_2^4} +\frac{13 (\mathbf {p}_2^2)^3}{8 m_2^6}, \end{aligned}$$

C.13 $$\begin{aligned}&H_{441}({\mathbf {x}}_a,{\mathbf {p}}_a) = \frac{5027 (\mathbf {n}_{12}\cdot \mathbf {p}_1)^4}{384 m_1^4} -\frac{22993 (\mathbf {n}_{12}\cdot \mathbf {p}_1)^2 \mathbf {p}_1^2}{960 m_1^4} -\frac{6695 (\mathbf {p}_1^2)^2}{1152 m_1^4} \nonumber \\&\quad -\frac{3191(\mathbf {n}_{12}\cdot \mathbf {p}_1)^3 (\mathbf {n}_{12}\cdot \mathbf {p}_2)}{640 m_1^3 m_2} +\frac{28561 (\mathbf {n}_{12}\cdot \mathbf {p}_1)(\mathbf {n}_{12}\cdot \mathbf {p}_2)\mathbf {p}_1^2}{1920 m_1^3 m_2} \nonumber \\&\quad +\frac{8777 (\mathbf {n}_{12}\cdot \mathbf {p}_1)^2(\mathbf {p}_1\cdot \mathbf {p}_2)}{384 m_1^3 m_2} +\frac{752969 \mathbf {p}_1^2(\mathbf {p}_1\cdot \mathbf {p}_2)}{28800 m_1^3 m_2} \nonumber \\&\quad -\frac{16481 (\mathbf {n}_{12}\cdot \mathbf {p}_1)^2 (\mathbf {n}_{12}\cdot \mathbf {p}_2)^2}{960m_1^2 m_2^2} +\frac{94433 (\mathbf {n}_{12}\cdot \mathbf {p}_2)^2 \mathbf {p}_1^2}{4800 m_1^2 m_2^2} \nonumber \\&\quad -\frac{103957 (\mathbf {n}_{12}\cdot \mathbf {p}_1)(\mathbf {n}_{12}\cdot \mathbf {p}_2)(\mathbf {p}_1\cdot \mathbf {p}_2)}{2400 m_1^2m_2^2} +\frac{791 (\mathbf {p}_1\cdot \mathbf {p}_2)^2}{400 m_1^2 m_2^2} \nonumber \\&\quad +\frac{26627 (\mathbf {n}_{12}\cdot \mathbf {p}_1)^2 \mathbf {p}_2^2}{1600 m_1^2 m_2^2} -\frac{118261 \mathbf {p}_1^2\mathbf {p}_2^2}{4800 m_1^2 m_2^2} +\frac{105 (\mathbf {p}_2^2)^2}{32 m_2^4}, \end{aligned}$$

C.14 $$\begin{aligned}&H_{442}({\mathbf {x}}_a,{\mathbf {p}}_a) = \left( \frac{2749\pi ^2}{8192}-\frac{211189}{19200}\right) \frac{(\mathbf {p}_1^2)^2}{m_1^4} +\left( \frac{375\pi ^2}{8192}-\frac{23533}{1280}\right) \frac{(\mathbf {n}_{12}\cdot \mathbf {p}_1)^4}{m_1^4} \nonumber \\&\quad +\left( \frac{63347}{1600}-\frac{1059\pi ^2}{1024}\right) \frac{(\mathbf {n}_{12}\cdot \mathbf {p}_1)^2 \mathbf {p}_1^2}{m_1^4} +\left( \frac{10631\pi ^2}{8192}-\frac{1918349}{57600}\right) \frac{(\mathbf {p}_1\cdot \mathbf {p}_2)^2}{m_1^2 m_2^2} \nonumber \\&\quad +\left( \frac{13723\pi ^2}{16384}-\frac{2492417}{57600}\right) \frac{\mathbf {p}_1^2\mathbf {p}_2^2}{m_1^2 m_2^2} +\left( \frac{1411429}{19200}-\frac{1059\pi ^2}{512}\right) \frac{(\mathbf {n}_{12}\cdot \mathbf {p}_2)^2\mathbf {p}_1^2}{m_1^2 m_2^2} \nonumber \\&\quad +\left( \frac{248991}{6400}-\frac{6153\pi ^2}{2048}\right) \frac{(\mathbf {n}_{12}\cdot \mathbf {p}_1)(\mathbf {n}_{12}\cdot \mathbf {p}_2)(\mathbf {p}_1\cdot \mathbf {p}_2)}{m_1^2 m_2^2} \nonumber \\&\quad -\left( \frac{30383}{960}+\frac{36405\pi ^2}{16384}\right) \frac{(\mathbf {n}_{12}\cdot \mathbf {p}_1)^2 (\mathbf {n}_{12}\cdot \mathbf {p}_2)^2}{m_1^2m_2^2} \nonumber \\&\quad +\left( \frac{2369}{60}+\frac{35655\pi ^2}{16384}\right) \frac{(\mathbf {n}_{12}\cdot \mathbf {p}_1)^3 (\mathbf {n}_{12}\cdot \mathbf {p}_2)}{m_1^3 m_2} \nonumber \\&\quad +\left( \frac{1243717}{14400}-\frac{40483\pi ^2}{16384}\right) \frac{\mathbf {p}_1^2(\mathbf {p}_1\cdot \mathbf {p}_2)}{m_1^3m_2} \nonumber \\&\quad +\left( \frac{43101\pi ^2}{16384}-\frac{391711}{6400}\right) \frac{(\mathbf {n}_{12}\cdot \mathbf {p}_1)(\mathbf {n}_{12}\cdot \mathbf {p}_2)\mathbf {p}_1^2}{m_1^3 m_2} \nonumber \\&\quad +\left( \frac{56955\pi ^2}{16384}-\frac{1646983}{19200}\right) \frac{(\mathbf {n}_{12}\cdot \mathbf {p}_1)^2 (\mathbf {p}_1\cdot \mathbf {p}_2)}{m_1^3 m_2}, \end{aligned}$$

C.15 $$\begin{aligned}&H_{421}({\mathbf {x}}_a,{\mathbf {p}}_a) = \frac{64861 \mathbf {p}_1^2}{4800 m_1^2} - \frac{91(\mathbf {p}_1\cdot \mathbf {p}_2)}{8 m_1 m_2} + \frac{105\mathbf {p}_2^2}{32 m_2^2} \nonumber \\&\quad - \frac{9841(\mathbf {n}_{12}\cdot \mathbf {p}_1)^2}{1600m_1^2} - \frac{7 (\mathbf {n}_{12}\cdot \mathbf {p}_1)(\mathbf {n}_{12}\cdot \mathbf {p}_2)}{2 m_1 m_2}, \end{aligned}$$

C.16 $$\begin{aligned}&H_{422}({\mathbf {x}}_a,{\mathbf {p}}_a) = \left( \frac{1937033}{57600}-\frac{199177 \pi ^2}{49152}\right) \frac{\mathbf {p}_1^2}{m_1^2} +\left( \frac{282361}{19200}-\frac{21837 \pi ^2}{8192}\right) \frac{\mathbf {p}_2^2}{m_2^2} \nonumber \\&\quad +\left( \frac{176033 \pi ^2}{24576}-\frac{2864917}{57600}\right) \frac{(\mathbf {p}_1\cdot \mathbf {p}_2)}{m_1 m_2} \nonumber \\&\quad +\left( \frac{698723}{19200}+\frac{21745 \pi ^2}{16384}\right) \frac{(\mathbf {n}_{12}\cdot \mathbf {p}_1)^2}{m_1^2} \nonumber \\&\quad +\left( \frac{63641 \pi ^2}{24576}-\frac{2712013}{19200}\right) \frac{(\mathbf {n}_{12}\cdot \mathbf {p}_1)(\mathbf {n}_{12}\cdot \mathbf {p}_2)}{m_1 m_2} \nonumber \\&\quad +\left( \frac{3200179}{57600}-\frac{28691 \pi ^2}{24576}\right) \frac{(\mathbf {n}_{12}\cdot \mathbf {p}_2)^2}{m_2^2}, \end{aligned}$$

C.17 $$\begin{aligned}&H_{40}({\mathbf {x}}_a,{\mathbf {p}}_a) = -\frac{m_1^4}{16} +\left( \frac{6237 \pi ^2}{1024}-\frac{169799}{2400}\right) m_1^3 m_2 \nonumber \\&\quad +\left( \frac{44825 \pi ^2}{6144}-\frac{609427}{7200}\right) m_1^2 m_2^2. \end{aligned}$$

The centre-of-energy vector $G^i({\mathbf {x}}_a,{\mathbf {p}}_a)$ was constructed with 3PN-accuracy (using the method of undetermined coefficients) by Damour et al. (2000c, 2000d), and at the 4PN level by Jaranowski and Schäfer (2015). It can be written as^13^

C.18 $$\begin{aligned} G^i({\mathbf {x}}_a,{\mathbf {p}}_a) = \sum _a \Big ( M_a(\mathbf{x}_b,\mathbf{p}_b)\,x_a^i + N_a(\mathbf{x}_b,\mathbf{p}_b)\,p_{ai} \Big ), \end{aligned}$$

where the functions $M_a$ and $N_a$ possess the following 4PN-accurate expansions

C.19 $$\begin{aligned} M_a({\mathbf {x}}_a,{\mathbf {p}}_a)&= m_a + \frac{1}{c^2}\,M_a^{\mathrm{1PN}}({\mathbf {x}}_a,{\mathbf {p}}_a) + \frac{1}{c^4} \, M_a^{\mathrm{2PN}}({\mathbf {x}}_a,{\mathbf {p}}_a) \nonumber \\&\quad + \frac{1}{c^6} \, M_a^{\mathrm{3PN}}({\mathbf {x}}_a,{\mathbf {p}}_a) + \frac{1}{c^8} \, M_a^{\mathrm{4PN}}({\mathbf {x}}_a,{\mathbf {p}}_a), \end{aligned}$$

C.20 $$\begin{aligned} N_a({\mathbf {x}}_a,{\mathbf {p}}_a)&= \frac{1}{c^4} \, N_a^{\mathrm{2PN}}({\mathbf {x}}_a,{\mathbf {p}}_a) + \frac{1}{c^6} \, N_a^{\mathrm{3PN}}({\mathbf {x}}_a,{\mathbf {p}}_a) + \frac{1}{c^8} \, N_a^{\mathrm{4PN}}({\mathbf {x}}_a,{\mathbf {p}}_a). \end{aligned}$$

The functions $M_1^{\mathrm{1PN}}$ to $M_1^{\mathrm{3PN}}$ read

C.21 $$\begin{aligned} M_1^{\mathrm{1PN}}({\mathbf {x}}_a,{\mathbf {p}}_a)= & {} \frac{\mathbf {p}_1^2}{2m_1} - \frac{Gm_1m_2}{2r_{12}}, \end{aligned}$$

C.22 $$\begin{aligned} M^{\text {2PN}}_1({\mathbf {x}}_a,{\mathbf {p}}_a)= & {} -\frac{(\mathbf {p}_1^2)^2}{8m_1^3} + \frac{Gm_1m_2}{4r_{12}} \Bigg ( -\frac{5\mathbf {p}_1^2}{m_1^2} - \frac{\mathbf{p}_2^2}{m_2^2} + \frac{7(\mathbf{p}_1\cdot \mathbf{p}_2)}{m_1m_2} \nonumber \\&+\, \frac{(\mathbf {n}_{12}\cdot \mathbf {p}_1)(\mathbf{n}_{12}\cdot \mathbf{p}_2)}{m_1m_2} \Bigg ) + \frac{G^2m_1m_2(m_1+m_2)}{4r_{12}^2}, \end{aligned}$$

C.23 $$\begin{aligned} M^{\text {3PN}}_1({\mathbf {x}}_a,{\mathbf {p}}_a)= & {} \frac{(\mathbf {p}_1^2)^3}{16m_1^5} + \frac{Gm_1m_2}{16r_{12}} \Bigg ( 9\,\frac{(\mathbf {p}_1^2)^2}{m_1^4} + \frac{(\mathbf{p}_2^2)^2}{m_2^4} - 11\,\frac{\mathbf {p}_1^2\,\mathbf{p}_2^2}{m_1^2m_2^2} - 2\,\frac{(\mathbf{p}_1\cdot \mathbf{p}_2)^2}{m_1^2m_2^2} \nonumber \\&+\, 3\,\frac{\mathbf {p}_1^2\,(\mathbf{n}_{12}\cdot \mathbf{p}_2)^2}{m_1^2m_2^2} + 7\,\frac{\mathbf{p}_2^2\,(\mathbf {n}_{12}\cdot \mathbf {p}_1)^2}{m_1^2m_2^2}\nonumber \\&-\, 12\,\frac{(\mathbf{p}_1\cdot \mathbf{p}_2)\,(\mathbf {n}_{12}\cdot \mathbf {p}_1)(\mathbf{n}_{12}\cdot \mathbf{p}_2)}{m_1^2m_2^2} - 3\,\frac{(\mathbf {n}_{12}\cdot \mathbf {p}_1)^2(\mathbf{n}_{12}\cdot \mathbf{p}_2)^2}{m_1^2m_2^2} \Bigg ) \nonumber \\&+\, \frac{G^2m_1m_2}{24r_{12}^2} \Bigg ( (112m_1+45m_2)\frac{\mathbf {p}_1^2}{m_1^2} + (15m_1+2m_2)\frac{\mathbf{p}_2^2}{m_2^2} \nonumber \\&-\, \frac{1}{2}(209m_1+115m_2)\frac{(\mathbf{p}_1\cdot \mathbf{p}_2)}{m_1m_2} + \frac{(\mathbf {n}_{12}\cdot \mathbf {p}_1)^2}{m_1} - \frac{(\mathbf{n}_{12}\cdot \mathbf{p}_2)^2}{m_2} \nonumber \\&-\, (31m_1+5m_2)\frac{(\mathbf {n}_{12}\cdot \mathbf {p}_1)(\mathbf{n}_{12}\cdot \mathbf{p}_2)}{m_1m_2} \Bigg ) \nonumber \\&-\, \frac{G^3m_1m_2 (m_1^2+5m_1m_2+m_2^2)}{8r_{12}^3}. \end{aligned}$$

The function $M_1^{\text {4PN}}$ has the following structure:

C.24 $$\begin{aligned} M_1^{\text {4PN}}({\mathbf {x}}_a,{\mathbf {p}}_a)&= -\frac{5(\mathbf {p}_1^2)^4}{128 m_1^7} + \frac{G m_1 m_2}{r_{12}} M_{46}({\mathbf {x}}_a,{\mathbf {p}}_a) \nonumber \\&\quad + \frac{G^2 m_1 m_2}{r_{12}^2} \Big (m_1\,M_{441}({\mathbf {x}}_a,{\mathbf {p}}_a) + m_2\,M_{442}({\mathbf {x}}_a,{\mathbf {p}}_a)\Big ) \nonumber \\&\quad + \frac{G^3 m_1 m_2}{r_{12}^3} \Big ( m_1^2\,M_{421}({\mathbf {x}}_a,{\mathbf {p}}_a) + m_1 m_2\,M_{422}({\mathbf {x}}_a,{\mathbf {p}}_a) \nonumber \\&\quad + m_2^2\,M_{423}({\mathbf {x}}_a,{\mathbf {p}}_a) \Big ) + \frac{G^4 m_1 m_2}{r_{12}^4}M_{40}({\mathbf {x}}_a,{\mathbf {p}}_a), \end{aligned}$$

where

$$\begin{aligned} M_{46}({\mathbf {x}}_a,{\mathbf {p}}_a)&= -\frac{13 (\mathbf {p}_1^2)^3}{32 m_1^6} -\frac{15 (\mathbf {n}_{12}\cdot \mathbf {p}_1)^4 (\mathbf {n}_{12}\cdot \mathbf {p}_2)^2}{256 m_1^4m_2^2} -\frac{91 (\mathbf {n}_{12}\cdot \mathbf {p}_2)^2 (\mathbf {p}_1^2)^2}{256 m_1^4 m_2^2} \nonumber \\&\quad +\frac{45 (\mathbf {n}_{12}\cdot \mathbf {p}_1)^2 (\mathbf {n}_{12}\cdot \mathbf {p}_2)^2 \mathbf {p}_1^2}{128 m_1^4m_2^2} -\frac{5(\mathbf {n}_{12}\cdot \mathbf {p}_1)^3 (\mathbf {n}_{12}\cdot \mathbf {p}_2)(\mathbf {p}_1\cdot \mathbf {p}_2)}{32 m_1^4 m_2^2} \nonumber \\&\quad +\frac{25 (\mathbf {n}_{12}\cdot \mathbf {p}_1)(\mathbf {n}_{12}\cdot \mathbf {p}_2)\mathbf {p}_1^2(\mathbf {p}_1\cdot \mathbf {p}_2)}{32 m_1^4 m_2^2} +\frac{5 (\mathbf {n}_{12}\cdot \mathbf {p}_1)^2(\mathbf {p}_1\cdot \mathbf {p}_2)^2}{64 m_1^4 m_2^2} \nonumber \\&\quad +\frac{7 \mathbf {p}_1^2(\mathbf {p}_1\cdot \mathbf {p}_2)^2}{64 m_1^4m_2^2} +\frac{11 (\mathbf {n}_{12}\cdot \mathbf {p}_1)^4 \mathbf {p}_2^2}{256 m_1^4 m_2^2} -\frac{47(\mathbf {n}_{12}\cdot \mathbf {p}_1)^2 \mathbf {p}_1^2\mathbf {p}_2^2}{128 m_1^4 m_2^2} \nonumber \\&\quad +\frac{91 (\mathbf {p}_1^2)^2\mathbf {p}_2^2}{256 m_1^4 m_2^2} +\frac{5 (\mathbf {n}_{12}\cdot \mathbf {p}_1)^3 (\mathbf {n}_{12}\cdot \mathbf {p}_2)^3}{32 m_1^3m_2^3} -\frac{7 (\mathbf {n}_{12}\cdot \mathbf {p}_1)(\mathbf {n}_{12}\cdot \mathbf {p}_2)^3 \mathbf {p}_1^2}{32 m_1^3 m_2^3} \nonumber \\&\quad +\frac{15 (\mathbf {n}_{12}\cdot \mathbf {p}_1)^2 (\mathbf {n}_{12}\cdot \mathbf {p}_2)^2 (\mathbf {p}_1\cdot \mathbf {p}_2)}{32 m_1^3 m_2^3} +\frac{7 (\mathbf {n}_{12}\cdot \mathbf {p}_2)^2\mathbf {p}_1^2(\mathbf {p}_1\cdot \mathbf {p}_2)}{32 m_1^3 m_2^3} \nonumber \\&\quad -\frac{5 (\mathbf {n}_{12}\cdot \mathbf {p}_1)(\mathbf {n}_{12}\cdot \mathbf {p}_2)(\mathbf {p}_1\cdot \mathbf {p}_2)^2}{16 m_1^3 m_2^3} -\frac{11(\mathbf {n}_{12}\cdot \mathbf {p}_1)^3 (\mathbf {n}_{12}\cdot \mathbf {p}_2)\mathbf {p}_2^2}{32 m_1^3 m_2^3} \nonumber \\&\quad -\frac{(\mathbf {p}_1\cdot \mathbf {p}_2)^3}{16 m_1^3 m_2^3} +\frac{7 (\mathbf {n}_{12}\cdot \mathbf {p}_1)(\mathbf {n}_{12}\cdot \mathbf {p}_2)\mathbf {p}_1^2\mathbf {p}_2^2}{32 m_1^3 m_2^3} -\frac{5 (\mathbf {n}_{12}\cdot \mathbf {p}_1)^2 (\mathbf {p}_1\cdot \mathbf {p}_2)\mathbf {p}_2^2}{32 m_1^3 m_2^3} \end{aligned}$$

C.25 $$\begin{aligned}&\quad +\frac{\mathbf {p}_1^2(\mathbf {p}_1\cdot \mathbf {p}_2)\mathbf {p}_2^2}{32 m_1^3m_2^3} +\frac{15 (\mathbf {n}_{12}\cdot \mathbf {p}_1)^2 (\mathbf {n}_{12}\cdot \mathbf {p}_2)^4}{256 m_1^2 m_2^4} -\frac{11(\mathbf {n}_{12}\cdot \mathbf {p}_2)^4 \mathbf {p}_1^2}{256 m_1^2 m_2^4} \nonumber \\&\quad +\frac{5 (\mathbf {n}_{12}\cdot \mathbf {p}_1)(\mathbf {n}_{12}\cdot \mathbf {p}_2)^3(\mathbf {p}_1\cdot \mathbf {p}_2)}{32 m_1^2 m_2^4} -\frac{5 (\mathbf {n}_{12}\cdot \mathbf {p}_2)^2 (\mathbf {p}_1\cdot \mathbf {p}_2)^2}{64 m_1^2m_2^4} \nonumber \\&\quad -\frac{21 (\mathbf {n}_{12}\cdot \mathbf {p}_1)^2 (\mathbf {n}_{12}\cdot \mathbf {p}_2)^2 \mathbf {p}_2^2}{128 m_1^2 m_2^4} +\frac{7(\mathbf {n}_{12}\cdot \mathbf {p}_2)^2 \mathbf {p}_1^2\mathbf {p}_2^2}{128 m_1^2 m_2^4} +\frac{(\mathbf {p}_1\cdot \mathbf {p}_2)^2 \mathbf {p}_2^2}{64 m_1^2m_2^4} \nonumber \\&\quad -\frac{(\mathbf {n}_{12}\cdot \mathbf {p}_1)(\mathbf {n}_{12}\cdot \mathbf {p}_2)(\mathbf {p}_1\cdot \mathbf {p}_2)\mathbf {p}_2^2}{32 m_1^2 m_2^4} +\frac{11 (\mathbf {n}_{12}\cdot \mathbf {p}_1)^2 (\mathbf {p}_2^2)^2}{256 m_1^2 m_2^4} \nonumber \\&\quad +\frac{37 \mathbf {p}_1^2(\mathbf {p}_2^2)^2}{256 m_1^2 m_2^4} -\frac{(\mathbf {p}_2^2)^3}{32 m_2^6}, \end{aligned}$$

C.26 $$\begin{aligned} M_{441}({\mathbf {x}}_a,{\mathbf {p}}_a)&= \frac{7711 (\mathbf {n}_{12}\cdot \mathbf {p}_1)^4}{3840 m_1^4} -\frac{2689 (\mathbf {n}_{12}\cdot \mathbf {p}_1)^2 \mathbf {p}_1^2}{3840m_1^4} +\frac{2683 (\mathbf {p}_1^2)^2}{1920 m_1^4} \nonumber \\&\quad -\frac{67 (\mathbf {n}_{12}\cdot \mathbf {p}_1)^3 (\mathbf {n}_{12}\cdot \mathbf {p}_2)}{30m_1^3 m_2} +\frac{1621 (\mathbf {n}_{12}\cdot \mathbf {p}_1)(\mathbf {n}_{12}\cdot \mathbf {p}_2)\mathbf {p}_1^2}{1920 m_1^3m_2} \nonumber \\&\quad -\frac{411 (\mathbf {n}_{12}\cdot \mathbf {p}_1)^2 (\mathbf {p}_1\cdot \mathbf {p}_2)}{1280 m_1^3 m_2} -\frac{25021 \mathbf {p}_1^2(\mathbf {p}_1\cdot \mathbf {p}_2)}{3840 m_1^3 m_2} \nonumber \\&\quad +\frac{289 (\mathbf {n}_{12}\cdot \mathbf {p}_1)^2 (\mathbf {n}_{12}\cdot \mathbf {p}_2)^2}{128 m_1^2m_2^2}-\frac{259 (\mathbf {n}_{12}\cdot \mathbf {p}_2)^2 \mathbf {p}_1^2}{128 m_1^2 m_2^2} \nonumber \\&\quad +\frac{689(\mathbf {n}_{12}\cdot \mathbf {p}_1)(\mathbf {n}_{12}\cdot \mathbf {p}_2)(\mathbf {p}_1\cdot \mathbf {p}_2)}{192 m_1^2 m_2^2} +\frac{11 (\mathbf {p}_1\cdot \mathbf {p}_2)^2}{48m_1^2 m_2^2} \nonumber \\&\quad -\frac{147 (\mathbf {n}_{12}\cdot \mathbf {p}_1)^2 \mathbf {p}_2^2}{64 m_1^2 m_2^2} +\frac{283\mathbf {p}_1^2\mathbf {p}_2^2}{64 m_1^2 m_2^2} +\frac{7 (\mathbf {n}_{12}\cdot \mathbf {p}_1)(\mathbf {n}_{12}\cdot \mathbf {p}_2)^3}{12m_1 m_2^3} \nonumber \\&\quad +\frac{49(\mathbf {n}_{12}\cdot \mathbf {p}_2)^2(\mathbf {p}_1\cdot \mathbf {p}_2)}{48 m_1 m_2^3} -\frac{7(\mathbf {n}_{12}\cdot \mathbf {p}_1)(\mathbf {n}_{12}\cdot \mathbf {p}_2)\mathbf {p}_2^2}{6 m_1 m_2^3} \nonumber \\&\quad -\frac{7(\mathbf {p}_1\cdot \mathbf {p}_2)\mathbf {p}_2^2}{48m_1 m_2^3} -\frac{9(\mathbf {p}_2^2)^2}{32 m_2^4}, \end{aligned}$$

C.27 $$\begin{aligned} M_{442}({\mathbf {x}}_a,{\mathbf {p}}_a)&= -\frac{45 (\mathbf {p}_1^2)^2}{32 m_1^4} +\frac{7 \mathbf {p}_1^2(\mathbf {p}_1\cdot \mathbf {p}_2)}{48 m_1^3m_2} +\frac{7 (\mathbf {n}_{12}\cdot \mathbf {p}_1)(\mathbf {n}_{12}\cdot \mathbf {p}_2)\mathbf {p}_1^2}{6 m_1^3 m_2} \nonumber \\&\quad -\frac{49(\mathbf {n}_{12}\cdot \mathbf {p}_1)^2 (\mathbf {p}_1\cdot \mathbf {p}_2)}{48 m_1^3 m_2} -\frac{7 (\mathbf {n}_{12}\cdot \mathbf {p}_1)^3 (\mathbf {n}_{12}\cdot \mathbf {p}_2)}{12 m_1^3 m_2} +\frac{7 (\mathbf {p}_1\cdot \mathbf {p}_2)^2}{24 m_1^2 m_2^2} \nonumber \\&\quad +\frac{635 \mathbf {p}_1^2\mathbf {p}_2^2}{192 m_1^2 m_2^2} -\frac{983 (\mathbf {n}_{12}\cdot \mathbf {p}_1)^2 \mathbf {p}_2^2}{384 m_1^2m_2^2} +\frac{413 (\mathbf {n}_{12}\cdot \mathbf {p}_1)^2 (\mathbf {n}_{12}\cdot \mathbf {p}_2)^2}{384 m_1^2 m_2^2} \nonumber \\&\quad -\frac{331(\mathbf {n}_{12}\cdot \mathbf {p}_2)^2 \mathbf {p}_1^2}{192 m_1^2 m_2^2} +\frac{437 (\mathbf {n}_{12}\cdot \mathbf {p}_1)(\mathbf {n}_{12}\cdot \mathbf {p}_2)(\mathbf {p}_1\cdot \mathbf {p}_2)}{64 m_1^2 m_2^2} \nonumber \\&\quad +\frac{11 (\mathbf {n}_{12}\cdot \mathbf {p}_1)(\mathbf {n}_{12}\cdot \mathbf {p}_2)^3}{15 m_1m_2^3} -\frac{1349 (\mathbf {n}_{12}\cdot \mathbf {p}_2)^2 (\mathbf {p}_1\cdot \mathbf {p}_2)}{1280 m_1 m_2^3} \nonumber \\&\quad -\frac{5221(\mathbf {n}_{12}\cdot \mathbf {p}_1)(\mathbf {n}_{12}\cdot \mathbf {p}_2)\mathbf {p}_2^2}{1920 m_1 m_2^3} -\frac{2579 (\mathbf {p}_1\cdot \mathbf {p}_2)\mathbf {p}_2^2}{3840 m_1 m_2^3}\nonumber \\&\quad +\frac{6769 (\mathbf {n}_{12}\cdot \mathbf {p}_2)^2 \mathbf {p}_2^2}{3840m_2^4} -\frac{2563 (\mathbf {p}_2^2)^2}{1920 m_2^4} -\frac{2037 (\mathbf {n}_{12}\cdot \mathbf {p}_2)^4}{1280 m_2^4}, \end{aligned}$$

C.28 $$\begin{aligned} M_{421}({\mathbf {x}}_a,{\mathbf {p}}_a)&= -\frac{179843 \mathbf {p}_1^2}{14400 m_1^2} +\frac{10223 (\mathbf {p}_1\cdot \mathbf {p}_2)}{1200 m_1m_2} -\frac{15 \mathbf {p}_2^2}{16 m_2^2} \nonumber \\&\quad +\frac{8881 (\mathbf {n}_{12}\cdot \mathbf {p}_1)(\mathbf {n}_{12}\cdot \mathbf {p}_2)}{2400 m_1m_2} +\frac{17737 (\mathbf {n}_{12}\cdot \mathbf {p}_1)^2}{1600 m_1^2}, \end{aligned}$$

C.29 $$\begin{aligned} M_{422}({\mathbf {x}}_a,{\mathbf {p}}_a)&= \left( \frac{8225 \pi ^2}{16384}-\frac{12007}{1152}\right) \frac{\mathbf {p}_1^2}{m_1^2} +\left( \frac{143}{16}-\frac{\pi ^2}{64}\right) \frac{(\mathbf {p}_1\cdot \mathbf {p}_2)}{m_1m_2} \nonumber \\&\quad +\left( \frac{655}{1152}-\frac{7969 \pi ^2}{16384}\right) \frac{\mathbf {p}_2^2}{m_2^2} +\left( \frac{6963 \pi ^2}{16384}-\frac{40697}{3840}\right) \frac{(\mathbf {n}_{12}\cdot \mathbf {p}_1)^2}{m_1^2} \nonumber \\&\quad +\left( \frac{119}{16}+\frac{3 \pi ^2}{64}\right) \frac{(\mathbf {n}_{12}\cdot \mathbf {p}_1)(\mathbf {n}_{12}\cdot \mathbf {p}_2)}{m_1 m_2} \nonumber \\&\quad +\left( \frac{30377}{3840}-\frac{7731 \pi ^2}{16384}\right) \frac{(\mathbf {n}_{12}\cdot \mathbf {p}_2)^2}{m_2^2}, \end{aligned}$$

C.30 $$\begin{aligned} M_{423}({\mathbf {x}}_a,{\mathbf {p}}_a)&= - \frac{35\mathbf {p}_1^2}{16 m_1^2} + \frac{1327(\mathbf {p}_1\cdot \mathbf {p}_2)}{1200 m_1 m_2} + \frac{52343\mathbf {p}_2^2}{14400 m_2^2} \nonumber \\&\quad - \frac{2581(\mathbf {n}_{12}\cdot \mathbf {p}_1)(\mathbf {n}_{12}\cdot \mathbf {p}_2)}{2400 m_1 m_2} - \frac{15737(\mathbf {n}_{12}\cdot \mathbf {p}_2)^2}{1600 m_2^2}, \end{aligned}$$

C.31 $$\begin{aligned} M_{40}({\mathbf {x}}_a,{\mathbf {p}}_a)&= \frac{m_1^3}{16} + \left( \frac{3371\pi ^2}{6144}-\frac{6701}{1440}\right) m_1^2 m_2 \nonumber \\&\quad + \left( \frac{20321}{1440}-\frac{7403\pi ^2}{6144}\right) m_1 m_2^2 + \frac{m_2^3}{16}. \end{aligned}$$

The functions $N_1^{\mathrm{2PN}}$ and $N_1^{\mathrm{3PN}}$ equal

C.32 $$\begin{aligned} N^{\text {2PN}}_1({\mathbf {x}}_a,{\mathbf {p}}_a)= & {} -\frac{5}{4}\,G\,(\mathbf{n}_{12}\cdot \mathbf{p}_2), \end{aligned}$$

C.33 $$\begin{aligned} N^{\text {3PN}}_1({\mathbf {x}}_a,{\mathbf {p}}_a)= & {} \frac{G}{8m_1m_2} \Big ( 2\,(\mathbf{p}_1\cdot \mathbf{p}_2)(\mathbf{n}_{12}\cdot \mathbf{p}_2)- \mathbf{p}_2^2\,(\mathbf {n}_{12}\cdot \mathbf {p}_1)\nonumber \\&+\, 3\,(\mathbf {n}_{12}\cdot \mathbf {p}_1)(\mathbf{n}_{12}\cdot \mathbf{p}_2)^2 \Big ) + \frac{G^2}{48r_{12}} \Big ( 19\,m_2\,(\mathbf {n}_{12}\cdot \mathbf {p}_1)\nonumber \\&+\, \left( 130\,m_1+137\,m_2\right) (\mathbf{n}_{12}\cdot \mathbf{p}_2)\Big ). \end{aligned}$$

The more complicated function $N_1^{\text {4PN}}$ has the structure:

C.34 $$\begin{aligned} N_1^{\mathrm{4PN}}({\mathbf {x}}_a,{\mathbf {p}}_a)&= G m_2 N_{45}({\mathbf {x}}_a,{\mathbf {p}}_a) + \frac{G^2 m_2}{r_{12}} \Big (m_1\,N_{431}({\mathbf {x}}_a,{\mathbf {p}}_a) \nonumber \\&\quad + m_2\,N_{432}({\mathbf {x}}_a,{\mathbf {p}}_a)\Big ) + \frac{G^3 m_2}{r_{12}^2} \Big (m_1^2\,N_{411}({\mathbf {x}}_a,{\mathbf {p}}_a) \nonumber \\&\quad + m_1 m_2\,N_{412}({\mathbf {x}}_a,{\mathbf {p}}_a) + m_2^2\,N_{413}({\mathbf {x}}_a,{\mathbf {p}}_a)\Big ), \end{aligned}$$

where

C.35 $$\begin{aligned}&N_{45}({\mathbf {x}}_a,{\mathbf {p}}_a) = -\frac{5 (\mathbf {n}_{12}\cdot \mathbf {p}_1)^3 (\mathbf {n}_{12}\cdot \mathbf {p}_2)^2}{64 m_1^3 m_2^2} +\frac{(\mathbf {n}_{12}\cdot \mathbf {p}_1)(\mathbf {n}_{12}\cdot \mathbf {p}_2)^2\mathbf {p}_1^2}{64 m_1^3 m_2^2} \nonumber \\&\quad +\frac{5 (\mathbf {n}_{12}\cdot \mathbf {p}_1)^2 (\mathbf {n}_{12}\cdot \mathbf {p}_2)(\mathbf {p}_1\cdot \mathbf {p}_2)}{32m_1^3 m_2^2} -\frac{(\mathbf {n}_{12}\cdot \mathbf {p}_2)\mathbf {p}_1^2(\mathbf {p}_1\cdot \mathbf {p}_2)}{32 m_1^3m_2^2} \nonumber \\&\quad +\frac{3 (\mathbf {n}_{12}\cdot \mathbf {p}_1)(\mathbf {p}_1\cdot \mathbf {p}_2)^2}{32 m_1^3 m_2^2} -\frac{(\mathbf {n}_{12}\cdot \mathbf {p}_1)^3\mathbf {p}_2^2}{64 m_1^3 m_2^2} -\frac{(\mathbf {n}_{12}\cdot \mathbf {p}_1)\mathbf {p}_1^2\mathbf {p}_2^2}{64 m_1^3m_2^2} \nonumber \\&\quad +\frac{(\mathbf {n}_{12}\cdot \mathbf {p}_1)^2 (\mathbf {n}_{12}\cdot \mathbf {p}_2)^3}{32 m_1^2 m_2^3} -\frac{7 (\mathbf {n}_{12}\cdot \mathbf {p}_2)^3\mathbf {p}_1^2}{32 m_1^2 m_2^3} +\frac{3 (\mathbf {n}_{12}\cdot \mathbf {p}_1)(\mathbf {n}_{12}\cdot \mathbf {p}_2)^2 (\mathbf {p}_1\cdot \mathbf {p}_2)}{16m_1^2 m_2^3} \nonumber \\&\quad +\frac{(\mathbf {n}_{12}\cdot \mathbf {p}_2)(\mathbf {p}_1\cdot \mathbf {p}_2)^2}{16 m_1^2 m_2^3} -\frac{9(\mathbf {n}_{12}\cdot \mathbf {p}_1)^2 (\mathbf {n}_{12}\cdot \mathbf {p}_2)\mathbf {p}_2^2}{32 m_1^2 m_2^3} +\frac{5 (\mathbf {n}_{12}\cdot \mathbf {p}_2)\mathbf {p}_1^2\mathbf {p}_2^2}{32 m_1^2 m_2^3} \nonumber \\&\quad -\frac{3 (\mathbf {n}_{12}\cdot \mathbf {p}_1)(\mathbf {p}_1\cdot \mathbf {p}_2)\mathbf {p}_2^2}{16 m_1^2m_2^3} -\frac{11 (\mathbf {n}_{12}\cdot \mathbf {p}_1)(\mathbf {n}_{12}\cdot \mathbf {p}_2)^4}{128 m_1 m_2^4} +\frac{(\mathbf {n}_{12}\cdot \mathbf {p}_2)^3(\mathbf {p}_1\cdot \mathbf {p}_2)}{32 m_1 m_2^4} \nonumber \\&\quad +\frac{7 (\mathbf {n}_{12}\cdot \mathbf {p}_1)(\mathbf {n}_{12}\cdot \mathbf {p}_2)^2 \mathbf {p}_2^2}{64 m_1m_2^4} +\frac{(\mathbf {n}_{12}\cdot \mathbf {p}_2)(\mathbf {p}_1\cdot \mathbf {p}_2)\mathbf {p}_2^2}{32 m_1 m_2^4} -\frac{3(\mathbf {n}_{12}\cdot \mathbf {p}_1)(\mathbf {p}_2^2)^2}{128 m_1 m_2^4}, \end{aligned}$$

C.36 $$\begin{aligned} N_{431}({\mathbf {x}}_a,{\mathbf {p}}_a)&= -\frac{387 (\mathbf {n}_{12}\cdot \mathbf {p}_1)^3}{1280 m_1^3} +\frac{10429 (\mathbf {n}_{12}\cdot \mathbf {p}_1)\mathbf {p}_1^2}{3840m_1^3} \nonumber \\&\quad -\frac{751 (\mathbf {n}_{12}\cdot \mathbf {p}_1)^2 (\mathbf {n}_{12}\cdot \mathbf {p}_2)}{480 m_1^2 m_2} +\frac{2209(\mathbf {n}_{12}\cdot \mathbf {p}_2)\mathbf {p}_1^2}{640 m_1^2 m_2} \nonumber \\&\quad -\frac{6851 (\mathbf {n}_{12}\cdot \mathbf {p}_1)(\mathbf {p}_1\cdot \mathbf {p}_2)}{1920m_1^2 m_2} +\frac{43 (\mathbf {n}_{12}\cdot \mathbf {p}_1)(\mathbf {n}_{12}\cdot \mathbf {p}_2)^2}{192 m_1 m_2^2} \nonumber \\&\quad -\frac{125(\mathbf {n}_{12}\cdot \mathbf {p}_2)(\mathbf {p}_1\cdot \mathbf {p}_2)}{192 m_1 m_2^2} +\frac{25 (\mathbf {n}_{12}\cdot \mathbf {p}_1)\mathbf {p}_2^2}{48 m_1m_2^2} \nonumber \\&\quad -\frac{7 (\mathbf {n}_{12}\cdot \mathbf {p}_2)^3}{8 m_2^3} +\frac{7 (\mathbf {n}_{12}\cdot \mathbf {p}_2)\mathbf {p}_2^2}{12 m_2^3}, \end{aligned}$$

C.37 $$\begin{aligned}&N_{432}({\mathbf {x}}_a,{\mathbf {p}}_a) = \frac{7 (\mathbf {n}_{12}\cdot \mathbf {p}_2)\mathbf {p}_1^2}{48 m_1^2 m_2} +\frac{7 (\mathbf {n}_{12}\cdot \mathbf {p}_1)(\mathbf {p}_1\cdot \mathbf {p}_2)}{24m_1^2 m_2} -\frac{49 (\mathbf {n}_{12}\cdot \mathbf {p}_1)^2 (\mathbf {n}_{12}\cdot \mathbf {p}_2)}{48 m_1^2 m_2} \nonumber \\&\quad +\frac{295(\mathbf {n}_{12}\cdot \mathbf {p}_1)(\mathbf {n}_{12}\cdot \mathbf {p}_2)^2}{384 m_1 m_2^2} -\frac{5 (\mathbf {n}_{12}\cdot \mathbf {p}_2)(\mathbf {p}_1\cdot \mathbf {p}_2)}{24m_1 m_2^2} -\frac{155 (\mathbf {n}_{12}\cdot \mathbf {p}_1)\mathbf {p}_2^2}{384 m_1 m_2^2} \nonumber \\&\quad -\frac{5999(\mathbf {n}_{12}\cdot \mathbf {p}_2)^3}{3840 m_2^3} +\frac{11251 (\mathbf {n}_{12}\cdot \mathbf {p}_2)\mathbf {p}_2^2}{3840 m_2^3}, \end{aligned}$$

C.38 $$\begin{aligned}&N_{411}({\mathbf {x}}_a,{\mathbf {p}}_a) = -\frac{37397(\mathbf {n}_{12}\cdot \mathbf {p}_1)}{7200m_1} - \frac{12311(\mathbf {n}_{12}\cdot \mathbf {p}_2)}{2400m_2}, \end{aligned}$$

C.39 $$\begin{aligned}&N_{412}({\mathbf {x}}_a,{\mathbf {p}}_a) = \left( \frac{5005\pi ^2}{8192}-\frac{81643}{11520}\right) \frac{(\mathbf {n}_{12}\cdot \mathbf {p}_1)}{m_1} \nonumber \\&\qquad \qquad \qquad \qquad + \left( \frac{773\pi ^2}{2048}-\frac{61177}{11520}\right) \frac{(\mathbf {n}_{12}\cdot \mathbf {p}_2)}{m_2}, \end{aligned}$$

C.40 $$\begin{aligned}&N_{413}({\mathbf {x}}_a,{\mathbf {p}}_a) = -\frac{7073(\mathbf {n}_{12}\cdot \mathbf {p}_2)}{1200 m_2}. \end{aligned}$$

## D Higher-order spin-dependent conservative Hamiltonians

In this appendix we present explicit formulae for higher-order spin-dependent conservative Hamiltonians not displayed in the main body of the review. We start with the next-to-next-to-leading-order spin–orbit Hamiltonian, which was calculated by Hartung et al. (2013) (see also Hartung and Steinhoff 2011a). It reads

$$\begin{aligned}&H^{\text {NNLO}}_{\text {SO}}({\mathbf {x}}_a,{\mathbf {p}}_a,\mathbf {S}_a) = \frac{G}{r_{12}^2} \left\{ \biggl ( \frac{7 m_2 (\mathbf {p}_{1}^2)^2}{16 m_1^5} + \frac{9 (\mathbf {n}_{12}\cdot \mathbf {p}_{1})(\mathbf {n}_{12}\cdot \mathbf {p}_{2})\mathbf {p}_{1}^2}{16 m_1^4}\right. \nonumber \\&\quad + \frac{3 \mathbf {p}_{1}^2 (\mathbf {n}_{12}\cdot \mathbf {p}_{2})^2}{4 m_1^3 m_2} + \frac{45 (\mathbf {n}_{12}\cdot \mathbf {p}_{1})(\mathbf {n}_{12}\cdot \mathbf {p}_{2})^3}{16 m_1^2 m_2^2} + \frac{9 \mathbf {p}_{1}^2 (\mathbf {p}_{1}\cdot \mathbf {p}_{2})}{16 m_1^4} \nonumber \\&\quad - \frac{3 (\mathbf {n}_{12}\cdot \mathbf {p}_{2})^2 (\mathbf {p}_{1}\cdot \mathbf {p}_{2})}{16 m_1^2 m_2^2} - \frac{3 (\mathbf {p}_{1}^2) (\mathbf {p}_{2}^2)}{16 m_1^3 m_2} - \frac{15 (\mathbf {n}_{12}\cdot \mathbf {p}_{1})(\mathbf {n}_{12}\cdot \mathbf {p}_{2}) \mathbf {p}_{2}^2}{16 m_1^2 m_2^2} \nonumber \\&\quad + \frac{3 (\mathbf {n}_{12}\cdot \mathbf {p}_{2})^2 \mathbf {p}_{2}^2}{4 m_1 m_2^3} - \frac{3 (\mathbf {p}_{1}\cdot \mathbf {p}_{2}) \mathbf {p}_{2}^2}{16 m_1^2 m_2^2} - \frac{3 (\mathbf {p}_{2}^2)^2}{16 m_1 m_2^3} \biggr )((\mathbf {n}_{12}\times \mathbf {p}_{1})\cdot \mathbf {S}_{1}) \nonumber \\&\quad + \biggl ( - \frac{3 (\mathbf {n}_{12}\cdot \mathbf {p}_{1})(\mathbf {n}_{12}\cdot \mathbf {p}_{2})\mathbf {p}_{1}^2}{2 m_1^3 m_2} - \frac{15 (\mathbf {n}_{12}\cdot \mathbf {p}_{1})^2(\mathbf {n}_{12}\cdot \mathbf {p}_{2})^2}{4 m_1^2 m_2^2} \nonumber \\&\quad + \frac{3 \mathbf {p}_{1}^2 (\mathbf {n}_{12}\cdot \mathbf {p}_{2})^2}{4 m_1^2 m_2^2} - \frac{\mathbf {p}_{1}^2 (\mathbf {p}_{1}\cdot \mathbf {p}_{2})}{2 m_1^3 m_2} + \frac{(\mathbf {p}_{1}\cdot \mathbf {p}_{2})^2}{2 m_1^2 m_2^2} \nonumber \\&\quad + \frac{3 (\mathbf {n}_{12}\cdot \mathbf {p}_{1})^2 \mathbf {p}_{2}^2}{4 m_1^2 m_2^2} - \frac{(\mathbf {p}_{1}^2) (\mathbf {p}_{2}^2)}{4 m_1^2 m_2^2} - \frac{3 (\mathbf {n}_{12}\cdot \mathbf {p}_{1})(\mathbf {n}_{12}\cdot \mathbf {p}_{2})\mathbf {p}_{2}^2}{2 m_1 m_2^3} \nonumber \\&\quad - \frac{(\mathbf {p}_{1}\cdot \mathbf {p}_{2}) \mathbf {p}_{2}^2}{2 m_1 m_2^3} \biggr )((\mathbf {n}_{12}\times \mathbf {p}_{2})\cdot \mathbf {S}_{1}) + \biggl ( - \frac{9 (\mathbf {n}_{12}\cdot \mathbf {p}_{1}) \mathbf {p}_{1}^2}{16 m_1^4} + \frac{\mathbf {p}_{1}^2 (\mathbf {n}_{12}\cdot \mathbf {p}_{2})}{m_1^3 m_2} \end{aligned}$$

D.1 $$\begin{aligned}&\qquad \qquad \quad + \frac{27 (\mathbf {n}_{12}\cdot \mathbf {p}_{1})(\mathbf {n}_{12}\cdot \mathbf {p}_{2})^2}{16 m_1^2 m_2^2} - \frac{(\mathbf {n}_{12}\cdot \mathbf {p}_{2})(\mathbf {p}_{1}\cdot \mathbf {p}_{2})}{8 m_1^2 m_2^2} - \frac{5 (\mathbf {n}_{12}\cdot \mathbf {p}_{1}) \mathbf {p}_{2}^2}{16 m_1^2 m_2^2} \nonumber \\&\left. \quad \qquad \qquad + \frac{(\mathbf {n}_{12}\cdot \mathbf {p}_{2})\mathbf {p}_{2}^2}{m_1 m_2^3} \biggr )((\mathbf {p}_{1} \times \mathbf {p}_{2})\cdot \mathbf {S}_{1}) \right\} \nonumber \\&\quad + \frac{G^2}{r_{12}^3} \left\{ \biggl ( -\frac{3 m_2 (\mathbf {n}_{12}\cdot \mathbf {p}_{1})^2}{2 m_1^2} {+}\left( -\frac{3 m_2}{2 m_1^2} +\frac{27 m_2^2}{8 m_1^3} \right) \mathbf {p}_{1}^2 +\left( \frac{177}{16 m_1} {+}\frac{11}{m_2} \right) (\mathbf {n}_{12}\cdot \mathbf {p}_{2})^2\right. \nonumber \\&\quad +\left( \frac{11}{2 m_1} +\frac{9 m_2}{2 m_1^2} \right) (\mathbf {n}_{12}\cdot \mathbf {p}_{1}) (\mathbf {n}_{12}\cdot \mathbf {p}_{2}) +\left( \frac{23}{4 m_1} +\frac{9 m_2}{2 m_1^2} \right) (\mathbf {p}_{1}\cdot \mathbf {p}_{2}) \nonumber \\&\quad -\left( \frac{159}{16 m_1} +\frac{37}{8 m_2} \right) \mathbf {p}_{2}^2 \biggr )((\mathbf {n}_{12}\times \mathbf {p}_{1})\cdot \mathbf {S}_{1}) +\biggl ( \frac{4 (\mathbf {n}_{12}\cdot \mathbf {p}_{1})^2}{m_1} +\frac{13 \mathbf {p}_{1}^2}{2 m_1} \nonumber \\&\quad +\frac{5 (\mathbf {n}_{12}\cdot \mathbf {p}_{2})^2}{m_2} +\frac{53 \mathbf {p}_{2}^2}{8 m_2} - \left( \frac{211}{8 m_1} +\frac{22}{m_2} \right) (\mathbf {n}_{12}\cdot \mathbf {p}_{1}) (\mathbf {n}_{12}\cdot \mathbf {p}_{2}) \nonumber \\&\quad -\left( \frac{47}{8 m_1} + \frac{5}{m_2} \right) (\mathbf {p}_{1}\cdot \mathbf {p}_{2}) \biggr )((\mathbf {n}_{12}\times \mathbf {p}_{2})\cdot \mathbf {S}_{1}) + \biggl ( -\left( \frac{8}{m_1} +\frac{9 m_2}{2 m_1^2} \right) (\mathbf {n}_{12}\cdot \mathbf {p}_{1}) \nonumber \\&\left. \quad + \left( \frac{59}{4 m_1} + \frac{27}{2 m_2}\right) (\mathbf {n}_{12}\cdot \mathbf {p}_{2}) \biggr ) ((\mathbf {p}_{1}\times \mathbf {p}_{2})\cdot \mathbf {S}_{1}) \right\} \nonumber \\&\quad + \frac{G^3}{r_{12}^4} \left\{ \left( \frac{181 m_1 m_2}{16} + \frac{95 m_2^2}{4} + \frac{75 m_2^3}{8 m_1} \right) ((\mathbf {n}_{12}\times \mathbf {p}_{1})\cdot \mathbf {S}_{1})\right. \nonumber \\&\left. \quad - \left( \frac{21 m_1^2}{2} + \frac{473 m_1 m_2}{16} + \frac{63 m_2^2}{4} \right) ((\mathbf {n}_{12}\times \mathbf {p}_{2})\cdot \mathbf {S}_{1}) \right\} + (1\leftrightarrow 2). \end{aligned}$$

The next-to-next-to-leading-order spin1–spin2 Hamiltonian was calculated for the first time by Hartung et al. (2013). Its explicit form reads

$$\begin{aligned}&H^{\text {NNLO}}_{S_1S_2}({\mathbf {x}}_a,{\mathbf {p}}_a,\mathbf {S}_a) = \frac{G}{r_{12}^3} \bigg \{ \frac{((\mathbf {p}_{1} \times \mathbf {p}_{2})\cdot \mathbf {S}_{1})((\mathbf {p}_{1} \times \mathbf {p}_{2})\cdot \mathbf {S}_{2})}{16 m_1^2 m_2^2} \nonumber \\&\quad - \frac{9((\mathbf {p}_{1} \times \mathbf {p}_{2})\cdot \mathbf {S}_{1})((\mathbf {n}_{12}\times \mathbf {p}_{2})\cdot \mathbf {S}_{2})(\mathbf {n}_{12}\cdot \mathbf {p}_{1})}{8 m_1^2 m_2^2} \nonumber \\&\quad - \frac{3((\mathbf {n}_{12}\times \mathbf {p}_{2})\cdot \mathbf {S}_{1})((\mathbf {p}_{1} \times \mathbf {p}_{2})\cdot \mathbf {S}_{2})(\mathbf {n}_{12}\cdot \mathbf {p}_{1})}{2 m_1^2 m_2^2} \nonumber \\&\quad + ((\mathbf {n}_{12}\times \mathbf {p}_{1})\cdot \mathbf {S}_{1})((\mathbf {n}_{12}\times \mathbf {p}_{1})\cdot \mathbf {S}_{2}) \biggl (\frac{9 \mathbf {p}_{1}^2}{8 m_1^4} + \frac{15 (\mathbf {n}_{12}\cdot \mathbf {p}_{2})^2}{4 m_1^2 m_2^2} - \frac{3 \mathbf {p}_{2}^2}{4 m_1^2 m_2^2} \biggr ) \nonumber \\&\quad + ((\mathbf {n}_{12}\times \mathbf {p}_{2})\cdot \mathbf {S}_{1})((\mathbf {n}_{12}\times \mathbf {p}_{1})\cdot \mathbf {S}_{2}) \biggl ( - \frac{3 \mathbf {p}_{1}^2}{2 m_1^3 m_2} + \frac{3 (\mathbf {p}_{1}\cdot \mathbf {p}_{2})}{4 m_1^2 m_2^2} \nonumber \\&\quad - \frac{15(\mathbf {n}_{12}\cdot \mathbf {p}_{1})(\mathbf {n}_{12}\cdot \mathbf {p}_{2})}{4 m_1^2 m_2^2} \biggr ) +((\mathbf {n}_{12}\times \mathbf {p}_{1})\cdot \mathbf {S}_{1})((\mathbf {n}_{12}\times \mathbf {p}_{2})\cdot \mathbf {S}_{2}) \nonumber \\&\quad \times \biggl ( \frac{3\mathbf {p}_{1}^2}{16 m_1^3 m_2} -\frac{3 (\mathbf {p}_{1}\cdot \mathbf {p}_{2})}{16 m_1^2 m_2^2} -\frac{15 (\mathbf {n}_{12}\cdot \mathbf {p}_{1})(\mathbf {n}_{12}\cdot \mathbf {p}_{2})}{16 m_1^2 m_2^2} \biggr ) \end{aligned}$$

$$\begin{aligned}&\quad \quad \quad + (\mathbf {p}_{1}\cdot \mathbf {S}_{1}) (\mathbf {p}_{1}\cdot \mathbf {S}_{2}) \bigg (\frac{3(\mathbf {n}_{12}\cdot \mathbf {p}_{2})^2}{4 m_1^2 m_2^2} - \frac{\mathbf {p}_{2}^2}{4 m_1^2 m_2^2}\bigg ) \nonumber \\&\quad + (\mathbf {p}_{1}\cdot \mathbf {S}_{1}) (\mathbf {p}_{2}\cdot \mathbf {S}_{2}) \bigg (-\frac{\mathbf {p}_{1}^2}{4 m_1^3 m_2} + \frac{(\mathbf {p}_{1}\cdot \mathbf {p}_{2})}{4 m_1^2 m_2^2}\bigg ) \nonumber \\&\quad + (\mathbf {p}_{2}\cdot \mathbf {S}_{1})(\mathbf {p}_{1}\cdot \mathbf {S}_{2}) \bigg (\frac{5\mathbf {p}_{1}^2}{16 m_1^3 m_2} - \frac{3(\mathbf {p}_{1}\cdot \mathbf {p}_{2})}{16 m_1^2 m_2^2} - \frac{9(\mathbf {n}_{12}\cdot \mathbf {p}_{1})(\mathbf {n}_{12}\cdot \mathbf {p}_{2})}{16 m_1^2 m_2^2}\bigg ) \nonumber \\&\quad + (\mathbf {n}_{12}\cdot \mathbf {S}_{1})(\mathbf {p}_{1}\cdot \mathbf {S}_{2}) \bigg (\frac{9(\mathbf {n}_{12}\cdot \mathbf {p}_{1})\mathbf {p}_{1}^2}{8 m_1^4} - \frac{3(\mathbf {n}_{12}\cdot \mathbf {p}_{2})\mathbf {p}_{1}^2}{4 m_1^3 m_2} - \frac{3(\mathbf {n}_{12}\cdot \mathbf {p}_{2})\mathbf {p}_{2}^2}{4 m_1 m_2^3}\bigg ) \nonumber \\&\quad + (\mathbf {p}_{1}\cdot \mathbf {S}_{1})(\mathbf {n}_{12}\cdot \mathbf {S}_{2}) \bigg (-\frac{3 (\mathbf {n}_{12}\cdot \mathbf {p}_{2}) \mathbf {p}_{1}^2}{4 m_1^3 m_2} - \frac{15 (\mathbf {n}_{12}\cdot \mathbf {p}_{1})(\mathbf {n}_{12}\cdot \mathbf {p}_{2})^2}{4 m_1^2 m_2^2} \nonumber \\&\quad + \frac{3(\mathbf {n}_{12}\cdot \mathbf {p}_{1})\mathbf {p}_{2}^2}{4 m_1^2 m_2^2} - \frac{3(\mathbf {n}_{12}\cdot \mathbf {p}_{2})\mathbf {p}_{2}^2}{4 m_1 m_2^3}\bigg ) + (\mathbf {n}_{12}\cdot \mathbf {S}_{1})(\mathbf {n}_{12}\cdot \mathbf {S}_{2}) \bigg (-\frac{3(\mathbf {p}_{1}\cdot \mathbf {p}_{2})^2{}}{8 m_1^2 m_2^2} \nonumber \\&\quad + \frac{105(\mathbf {n}_{12}\cdot \mathbf {p}_{1})^2 (\mathbf {n}_{12}\cdot \mathbf {p}_{2})^2}{16 m_1^2 m_2^2} - \frac{15 (\mathbf {n}_{12}\cdot \mathbf {p}_{2})^2 \mathbf {p}_{1}^2}{8 m_1^2 m_2^2} + \frac{3 \mathbf {p}_{1}^2(\mathbf {p}_{1}\cdot \mathbf {p}_{2})}{4 m_1^3 m_2} + \frac{3 \mathbf {p}_{1}^2 \mathbf {p}_{2}^2}{16 m_1^2 m_2^2} \nonumber \\&\quad + \frac{15 \mathbf {p}_{1}^2 (\mathbf {n}_{12}\cdot \mathbf {p}_{1})(\mathbf {n}_{12}\cdot \mathbf {p}_{2})}{4 m_1^3 m_2}\bigg ) + (\mathbf {S}_{1}\cdot \mathbf {S}_{2}) \biggl (\frac{(\mathbf {p}_{1}\cdot \mathbf {p}_{2})^2}{16 m_1^2 m_2^2} - \frac{9 (\mathbf {n}_{12}\cdot \mathbf {p}_{1})^2 \mathbf {p}_{1}^2}{8 m_1^4} \nonumber \\&\quad - \frac{5 (\mathbf {p}_{1}\cdot \mathbf {p}_{2}) \mathbf {p}_{1}^2}{16 m_1^3 m_2} - \frac{3 (\mathbf {n}_{12}\cdot \mathbf {p}_{2})^2\mathbf {p}_{1}^2}{8 m_1^2 m_2^2} - \frac{15 (\mathbf {n}_{12}\cdot \mathbf {p}_{1})^2 (\mathbf {n}_{12}\cdot \mathbf {p}_{2})^2}{16 m_1^2 m_2^2} + \frac{3 \mathbf {p}_{1}^2 \mathbf {p}_{2}^2}{16 m_1^2 m_2^2} \nonumber \\&\quad + \frac{3\mathbf {p}_{1}^2(\mathbf {n}_{12}\cdot \mathbf {p}_{1})(\mathbf {n}_{12}\cdot \mathbf {p}_{2})}{4 m_1^3 m_2} + \frac{9(\mathbf {p}_{1}\cdot \mathbf {p}_{2})(\mathbf {n}_{12}\cdot \mathbf {p}_{1})(\mathbf {n}_{12}\cdot \mathbf {p}_{2})}{16 m_1^2 m_2^2}\bigg ) \bigg \} \nonumber \\&\quad + \frac{G^2}{r_{12}^4} \bigg \{ ((\mathbf {n}_{12}\times \mathbf {p}_{1})\cdot \mathbf {S}_{1})((\mathbf {n}_{12}\times \mathbf {p}_{1})\cdot \mathbf {S}_{2}) \bigg (\frac{12}{m_1} + \frac{9 m_2}{m_1^2}\bigg ) \nonumber \\&\quad - \frac{81}{4m_1}((\mathbf {n}_{12}\times \mathbf {p}_{2})\cdot \mathbf {S}_{1})((\mathbf {n}_{12}\times \mathbf {p}_{1})\cdot \mathbf {S}_{2}) \nonumber \\&\quad - \frac{27}{4m_1}((\mathbf {n}_{12}\times \mathbf {p}_{1})\cdot \mathbf {S}_{1})((\mathbf {n}_{12}\times \mathbf {p}_{2})\cdot \mathbf {S}_{2}) \nonumber \\&\quad - \frac{5}{2 m_1}(\mathbf {p}_{1}\cdot \mathbf {S}_{1})(\mathbf {p}_{2}\cdot \mathbf {S}_{2}) + \frac{29}{8 m_1}(\mathbf {p}_{2}\cdot \mathbf {S}_{1})(\mathbf {p}_{1}\cdot \mathbf {S}_{2}) - \frac{21}{8 m_1}(\mathbf {p}_{1}\cdot \mathbf {S}_{1})(\mathbf {p}_{1}\cdot \mathbf {S}_{2}) \end{aligned}$$

D.2 $$\begin{aligned}&\quad + (\mathbf {n}_{12}\cdot \mathbf {S}_{1})(\mathbf {p}_{1}\cdot \mathbf {S}_{2})\bigg [ \left( \frac{33}{2 m_1} + \frac{9 m_2}{m_1^2}\right) (\mathbf {n}_{12}\cdot \mathbf {p}_{1}) - \left( \frac{14}{m_1} + \frac{29}{2 m_2}\right) (\mathbf {n}_{12}\cdot \mathbf {p}_{2})\bigg ] \nonumber \\&\quad + (\mathbf {p}_{1}\cdot \mathbf {S}_{1}) (\mathbf {n}_{12}\cdot \mathbf {S}_{2}) \bigg [ \frac{4}{m_1}(\mathbf {n}_{12}\cdot \mathbf {p}_{1}) - \left( \frac{11}{m_1} + \frac{11}{m_2}\right) (\mathbf {n}_{12}\cdot \mathbf {p}_{2})\bigg ] \nonumber \\&\quad + (\mathbf {n}_{12}\cdot \mathbf {S}_{1}) (\mathbf {n}_{12}\cdot \mathbf {S}_{2}) \bigg [ - \frac{12}{m_1} (\mathbf {n}_{12}\cdot \mathbf {p}_{1})^2 - \frac{10}{m_1} \mathbf {p}_{1}^2 + \frac{37}{4 m_1}(\mathbf {p}_{1}\cdot \mathbf {p}_{2}) \nonumber \\&\quad + \frac{255}{4 m_1}(\mathbf {n}_{12}\cdot \mathbf {p}_{1})(\mathbf {n}_{12}\cdot \mathbf {p}_{2})\bigg ] + (\mathbf {S}_{1}\cdot \mathbf {S}_{2}) \bigg [ - \left( \frac{25}{2 m_1} + \frac{9 m_2}{m_1^2}\right) (\mathbf {n}_{12}\cdot \mathbf {p}_{1})^2 \nonumber \\&\quad + \frac{49}{8 m_1} \mathbf {p}_{1}^2 + \frac{35}{4 m_1}(\mathbf {n}_{12}\cdot \mathbf {p}_{1})(\mathbf {n}_{12}\cdot \mathbf {p}_{2}) - \frac{43}{8 m_1}(\mathbf {p}_{1}\cdot \mathbf {p}_{2})\bigg ] \bigg \} \nonumber \\&\quad + \frac{G^3}{r_{12}^5} \bigg \{ - (\mathbf {S}_{1}\cdot \mathbf {S}_{2}) \left( \frac{63}{4} m_1^2 + \frac{145}{8} m_1 m_2\right) \nonumber \\&\quad + (\mathbf {n}_{12}\cdot \mathbf {S}_{1}) (\mathbf {n}_{12}\cdot \mathbf {S}_{2}) \left( \frac{105}{4} m_1^2 + \frac{289}{8} m_1 m_2\right) \bigg \} + (1\leftrightarrow 2). \end{aligned}$$

Leading-order cubic in spin Hamiltonians (which are also proportional to the linear momenta of the bodies) were derived by Hergt and Schäfer (2008a, b) and Levi and Steinhoff (2015). They are collected here into the single Hamiltonian $H_{pS^3}^{\text {LO}}$, which equals

D.3 $$\begin{aligned} H_{pS^3}^{\text {LO}}({\mathbf {x}}_a,{\mathbf {p}}_a,\mathbf {S}_a)&\equiv H_{p_{1}S_{2}^3} + H_{p_{2}S_{1}^3} + H_{p_{1}S_{1}^3} + H_{p_{2}S_{2}^3} \nonumber \\&\quad + H_{p_{1}S_{1}S_{2}^2} + H_{p_{2}S_{2}S_{1}^2} + H_{p_{1}S_{2}S_{1}^2} + H_{p_2 S_1 S_2^2} \nonumber \\&= \frac{G}{m_1^2r_{12}^4} \left\{ \frac{3}{2}\left[ \mathbf {S}_{1}^2\,(\mathbf {S}_{2}\cdot (\mathbf {n}_{12}\times \mathbf {p}_{1})) + (\mathbf {S}_{1}\cdot \mathbf {n}_{12})\,(\mathbf {S}_{2}\cdot (\mathbf {S}_{1}\times \mathbf {p}_{1}))\right. \right. \nonumber \\&\quad + (\mathbf {n}_{12}\cdot (\mathbf {S}_{1}\times \mathbf {S}_{2}))\big ((\mathbf {S}_{1}\cdot \mathbf {p}_{1})-5(\mathbf {S}_{1}\cdot \mathbf {n}_{12})(\mathbf {p}_{1}\cdot \mathbf {n}_{12})\big ) \nonumber \\&\quad - 5(\mathbf {S}_{1}\cdot \mathbf {n}_{12})^2\,(\mathbf {S}_{2}\cdot (\mathbf {n}_{12}\times \mathbf {p}_{1})) -\frac{3m_1}{2m_2} \Big ( \mathbf {S}_{1}^2\,(\mathbf {S}_{2}\cdot (\mathbf {n}_{12}\times \mathbf {p}_2)) \nonumber \\&\left. \quad + 2(\mathbf {S}_{1}\cdot \mathbf {n}_{12})(\mathbf {S}_{2}\cdot (\mathbf {S}_{1}\times \mathbf {p}_{2})) - 5(\mathbf {S}_{1}\cdot \mathbf {n}_{12})^2(\mathbf {S}_{2}\cdot (\mathbf {n}_{12}\times \mathbf {p}_{2})) \Big )\right] \nonumber \\&\left. \quad - (\mathbf {S}_{1}\times \mathbf {n}_{12})\cdot \Big (\mathbf {p}_{2} - \frac{m_2}{4m_1}\mathbf {p}_{1}\Big ) \left( \mathbf {S}_{1}^2 - 5\left( \mathbf {S}_{1}\cdot \mathbf {n}_{12}\right) ^2 \right) \right\} + (1\leftrightarrow 2). \end{aligned}$$

Leading-order quartic in spin Hamiltonians were derived by Levi and Steinhoff (2015). They are collected here into the single Hamiltonian $H_{S^4}^{\text {LO}}$, which reads

D.4 $$\begin{aligned} H_{S^4}^{\text {LO}}({\mathbf {x}}_a,\mathbf {S}_a)&\equiv H_{S_{1}^2S_{2}^2} + H_{S_{1} S_{2}^3} + H_{S_{2}S_{1}^3} + H_{S_{1}^4} + H_{S_{2}^4} \nonumber \\&= -\frac{3G}{2m_{1}m_{2}r_{12}^5} \bigg \{ \frac{1}{2}\mathbf {S}_{1}^2\mathbf {S}_{2}^2+\left( \mathbf {S}_{1}\cdot \mathbf {S}_{2}\right) ^2 - \frac{5}{2}\left( \mathbf {S}_{1}^2\left( \mathbf {S}_{2}\cdot \mathbf {n}_{12}\right) ^2 + \mathbf {S}_{2}^2\left( \mathbf {S}_{1}\cdot \mathbf {n}_{12}\right) ^2\right) \nonumber \\&\quad - 10(\mathbf {S}_{1}\cdot \mathbf {n}_{12})\,(\mathbf {S}_{2}\cdot \mathbf {n}_{12}) \left( (\mathbf {S}_{1}\cdot \mathbf {S}_{2}) -\frac{7}{4}(\mathbf {S}_{1}\cdot \mathbf {n}_{12})\,(\mathbf {S}_{2}\cdot \mathbf {n}_{12})\right) \bigg \} \nonumber \\&\quad - \frac{3G}{2m_{1}^2r_{12}^5} \bigg \{ \mathbf {S}_{1}^2\,(\mathbf {S}_{1}\cdot \mathbf {S}_{2}) - 5(\mathbf {S}_{1}\cdot \mathbf {S}_{2})(\mathbf {S}_{1}\cdot \mathbf {n}_{12})^2 \nonumber \\&\quad - 5\mathbf {S}_{1}^2\,(\mathbf {S}_{1}\cdot \mathbf {n}_{12})\,(\mathbf {S}_{2}\cdot \mathbf {n}_{12}) + \frac{35}{3}(\mathbf {S}_{2}\cdot \mathbf {n}_{12})(\mathbf {S}_{1}\cdot \mathbf {n}_{12})^3 \bigg \} \nonumber \\&\quad -\frac{3Gm_2}{8m_1^3r_{12}^5} \bigg \{ S_1^4(\mathbf {S}_{1}^2)^2 - 10\mathbf {S}_{1}^2\left( \mathbf {S}_1\cdot \mathbf {n}_{12}\right) ^2 +\frac{35}{3}\left( \mathbf {S}_1\cdot \mathbf {n}_{12}\right) ^4 \bigg \} + (1\leftrightarrow 2). \end{aligned}$$

Let us note that it is possible to compute the leading-order Hamiltonians to all orders in spin (Vines and Steinhoff 2018).

## E Dissipative many-point-mass Hamiltonians

In this appendix we display all known dissipative Hamiltonians for many-body systems (i.e. for systems comprising any number of components), made of both spinless or spinning bodies. We start by displaying the dissipative leading-order 2.5PN and next-to-leading-order 3.5PN ADM Hamiltonians valid for spinless bodies. The 2.5PN Hamiltonian is given in Eq. (6.79) for two-body systems, but in this appendix we display formula for it valid for many-body systems. The 3.5PN Hamiltonian was computed for the first time by Jaranowski and Schäfer (1997). The Hamiltonians read [in this appendix we use units in which $c=1$ and $G=1/(16\pi )$]^14^

E.1 $$\begin{aligned} H_{\mathrm{2.5PN}}({\mathbf {x}}_a,{\mathbf {p}}_a,t)&= 5\pi \,\dot{\chi }_{(4)ij}(t)\,\chi _{(4)ij}({\mathbf {x}}_a,{\mathbf {p}}_a), \end{aligned}$$

E.2 $$\begin{aligned} H_{\mathrm{3.5PN}}({\mathbf {x}}_a,{\mathbf {p}}_a,t)&= 5\pi \,\chi _{(4)ij}({\mathbf {x}}_a,{\mathbf {p}}_a) \big (\dot{\varPi }_{1ij}(t) + \dot{\varPi }_{2ij}(t) + \ddot{\varPi }_{3ij}(t)\big ) \nonumber \\&\quad + 5\pi \,\dot{\chi }_{(4)ij}(t) \big ({\varPi }_{1ij}({\mathbf {x}}_a,{\mathbf {p}}_a) + \widetilde{\varPi }_{2ij}({\mathbf {x}}_a,t)\big ) \nonumber \\&\quad - 5\pi \,\ddot{\chi }_{(4)ij}(t) {\varPi }_{3ij}({\mathbf {x}}_a,{\mathbf {p}}_a) \nonumber \\&\quad + \dot{\chi }_{(4)ij}(t) \big (Q'_{ij}({\mathbf {x}}_a,{\mathbf {p}}_a,t) + Q''_{ij}({\mathbf {x}}_a,t)\big ) \nonumber \\&\quad + \frac{\partial ^3}{\partial t^3} \big (R'({\mathbf {x}}_a,{\mathbf {p}}_a,t) + R''({\mathbf {x}}_a,t)\big ). \end{aligned}$$

To display the building blocks of these Hamiltonians we adopt the notation that the explicit dependence on time *t* is through canonical variables with *primed* indices only, e.g., $\chi _{(4)ij}(t)\equiv \chi _{(4)ij}(\mathbf {x}_{a'}(t),\mathbf {p}_{a'}(t))$. We also define $s_{abc}\equiv r_{ab}+r_{bc}+r_{ca}$, $s_{aa'b'}\equiv r_{aa'} + r_{ab'} + r_{a'b'}$, and $s_{aba'}\equiv r_{ab}+r_{aa'}+r_{ba'}$. The building blocks are then defined as follows^15^:

E.3 $$\begin{aligned}&\chi _{(4)ij}({\mathbf {x}}_a,{\mathbf {p}}_a) \equiv \frac{8}{15}\frac{1}{16\pi } \sum \limits _a \frac{1}{m_a} \left( {\mathbf {p}}_a^2 \delta _{ij} - 3 p_{ai} p_{aj} \right) \nonumber \\&\quad + \frac{4}{15}\frac{1}{(16\pi )^2} \sum \limits _a\sum \limits _{b\ne a} \frac{m_a m_b}{r_{ab}} (3 n_{ab}^i n_{ab}^j - \delta _{ij}), \end{aligned}$$

E.4 $$\begin{aligned}&\varPi _{1ij}\left( {\mathbf {x}}_a,{\mathbf {p}}_a\right) \equiv \frac{4}{15}\frac{1}{16\pi } \sum \limits _a\frac{{\mathbf {p}}_a^2}{m_a^3} \left( -{\mathbf {p}}_a^2\delta _{ij} + 3p_{ai}p_{aj} \right) \nonumber \\&\quad + \frac{8}{5} \frac{1}{(16\pi )^2} \sum \limits _a\sum \limits _{b\ne a} \frac{m_b}{m_a r_{ab}} \left( - 2 {\mathbf {p}}_a^2\delta _{ij} + 5p_{ai}p_{aj} + {\mathbf {p}}_a^2 n_{ab}^i n_{ab}^j \right) \nonumber \\&\quad + \frac{1}{5} \frac{1}{(16\pi )^2} \sum \limits _a\sum \limits _{b\ne a} \frac{1}{r_{ab}} \Big \{ \Big [19 ({\mathbf {p}}_a\cdot \mathbf {p}_b) - 3 (\mathbf {n}_{ab}\cdot {\mathbf {p}}_a) (\mathbf {n}_{ab}\cdot \mathbf {p}_b)\Big ]\delta _{ij} \nonumber \\&\quad - 42 {p_{ai}p_{bj}} -3\Big [5({\mathbf {p}}_a\cdot \mathbf {p}_b) + (\mathbf {n}_{ab}\cdot {\mathbf {p}}_a) (\mathbf {n}_{ab}\cdot \mathbf {p}_b) \Big ] n_{ab}^i n_{ab}^j\nonumber \\&\quad + 6 (\mathbf {n}_{ab}\cdot \mathbf {p}_b) \left( n_{ab}^i p_{aj} + n_{ab}^j p_{ai} \right) \Big \} \nonumber \\&\quad + \frac{41}{15} \frac{1}{(16\pi )^3} \sum \limits _a\sum \limits _{b\ne a} \frac{m_a^2 m_b}{r_{ab}^2} \left( \delta _{ij} - 3 n_{ab}^i n_{ab}^j \right) \nonumber \\&\quad + \frac{1}{45} \frac{1}{(16\pi )^3} \sum \limits _a\sum \limits _{b\ne a}\sum \limits _{c\ne a,b} m_a m_b m_c \bigg \{ \frac{18}{r_{ab}r_{ca}} \left( \delta _{ij} - 3 n_{ab}^i n_{ab}^j \right) \nonumber \\&\quad - \frac{180}{s_{abc}} \left[ \left( \frac{1}{r_{ab}} + \frac{1}{s_{abc}} \right) n_{ab}^i n_{ab}^j + \frac{1}{s_{abc}} n_{ab}^i n_{bc}^j \right] \nonumber \\&\quad + \frac{10}{s_{abc}} \left[ 4 \left( \frac{1}{r_{ab}} + \frac{1}{r_{bc}} + \frac{1}{r_{ca}} \right) - \frac{r_{ab}^2 + r_{bc}^2 + r_{ca}^2}{r_{ab}r_{bc}r_{ca}} \right] \delta _{ij} \bigg \}, \end{aligned}$$

E.5 $$\begin{aligned}&\varPi _{2ij}({\mathbf {x}}_a,{\mathbf {p}}_a) \equiv \frac{1}{5}\frac{1}{(16\pi )^2} \sum \limits _a\sum \limits _{b\ne a} \frac{m_b}{m_a r_{ab}} \left\{ \Big [5(\mathbf {n}_{ab}\cdot {\mathbf {p}}_a)^2 - {\mathbf {p}}_a^2 \Big ] \delta _{ij} - 2p_{ai}p_{aj}\right. \nonumber \\&\left. \quad + \Big [5{\mathbf {p}}_a^2 - 3(\mathbf {n}_{ab}\cdot {\mathbf {p}}_a)^2 \Big ] n_{ab}^i n_{ab}^j - 6(\mathbf {n}_{ab}\cdot {\mathbf {p}}_a)(n_{ab}^i p_{aj} + n_{ab}^j p_{ai}) \right\} \nonumber \\&\quad + \frac{6}{5} \frac{1}{(16\pi )^3} \sum \limits _a\sum \limits _{b\ne a} \frac{m_a^2 m_b}{r_{ab}^2} \left( 3 n_{ab}^i n_{ab}^j - \delta _{ij} \right) \nonumber \\&\quad +\frac{1}{10}\frac{1}{(16\pi )^3} \sum \limits _a\sum \limits _{b\ne a}\sum \limits _{c\ne a,b} m_a m_b m_c \left\{ \left[ 5\frac{r_{ca}}{r_{ab}^3}\left( 1-\frac{r_{ca}}{r_{bc}}\right) +\frac{13}{r_{ab}r_{ca}} -\frac{40}{r_{ab}s_{abc}} \right] \delta _{ij}\right. \nonumber \\&\quad +\left[ 3\frac{r_{ab}}{r_{ca}^3} +\frac{r_{bc}^2}{r_{ab}r_{ca}^3} -\frac{5}{r_{ab}r_{ca}} +\frac{40}{s_{abc}}\left( \frac{1}{r_{ab}}+\frac{1}{s_{abc}}\right) \right] n_{ab}^i n_{ab}^j \nonumber \\&\left. \quad +\left[ 2 \frac{(r_{ab} + r_{ca})}{r_{bc}^3} - 16 \left( \frac{1}{r_{ab}^2} + \frac{1}{r_{ca}^2} \right) + \frac{88}{s_{abc}^2} \right] n_{ab}^i n_{ca}^j \right\} , \end{aligned}$$

E.6 $$\begin{aligned}&\varPi _{3ij}\left( {\mathbf {x}}_a,{\mathbf {p}}_a\right) \equiv \frac{1}{5} \frac{1}{(16\pi )^2} \sum \limits _a\sum \limits _{b\ne a} m_b \left\{ - 5(\mathbf {n}_{ab}\cdot {\mathbf {p}}_a) \delta _{ij}\right. \nonumber \\&\left. \quad + (\mathbf {n}_{ab}\cdot {\mathbf {p}}_a) n_{ab}^i n_{ab}^j + 7( n_{ab}^i p_{aj} + n_{ab}^j p_{ai}) \right\} , \end{aligned}$$

E.7 $$\begin{aligned}&\widetilde{\varPi }_{2ij}({\mathbf {x}}_a,t) \equiv \frac{1}{5}\frac{1}{(16\pi )^2} \sum \limits _{a}\sum \limits _{a'}\frac{m_a}{m_{a'}r_{aa'}} \left\{ \big (5(\mathbf {n}_{a{a'}}\cdot \mathbf {p}_{a'})^2-\mathbf {p}_{a'}^2\big )\delta _{ij} - 2 p_{{a'}i} p_{{a'}j}\right. \nonumber \\&\left. \quad +\big (5\mathbf {p}_{a'}^2 - 3 (\mathbf {n}_{{aa'}}\cdot \mathbf {p}_{a'})^2\big ) n_{{aa'}}^i n_{{aa'}}^j - 6 (\mathbf {n}_{{aa'}}\cdot \mathbf {p}_{a'}) (n_{aa'}^i p_{{a'}j} + n_{aa'}^j p_{{a'}i})\right\} \nonumber \\&\quad +\frac{1}{10}\frac{1}{(16\pi )^3} \sum \limits _{a}\sum \limits _{a'}\sum \limits _{{b'}\ne {a'}} m_a m_{a'} m_{b'} \bigg \{ \frac{32}{s_{a{a'}{b'}}} \big (\frac{1}{r_{{a'}{b'}}} + \frac{1}{s_{a{a'}{b'}}}\big ) n_{{a'}{b'}}^i n_{{a'}{b'}}^j \nonumber \\&\quad + 16 \big ( \frac{1}{r_{{a'}{b'}}^2} - \frac{2}{s_{a{a'}{b'}}^2} \big ) ( n_{aa'}^i n_{{a'}{b'}}^j + n_{aa'}^j n_{{a'}{b'}}^i ) - 2 \big (\frac{r_{aa'} + r_{ab'}}{r_{{a'}{b'}}^3} + \frac{12}{s_{a{a'}{b'}}^2}\big ) n_{aa'}^i n_{a{b'}}^j \nonumber \\&\quad +\big [ \frac{r_{aa'}}{r_{{a'}{b'}}^3} \big (\frac{r_{aa'}}{r_{ab'}} + 3\big ) -\frac{5}{r_{{a'}{b'}}r_{aa'}} +\frac{8}{s_{a{a'}{b'}}} \big (\frac{1}{r_{aa'}} + \frac{1}{s_{a{a'}{b'}}}\big ) \big ] n_{aa'}^i n_{{aa'}}^j \nonumber \\&\quad +\big [ 5\frac{r_{aa'}}{r_{{a'}{b'}}^3} \big ( 1 - \frac{r_{aa'}}{r_{ab'}} \big ) + \frac{17}{r_{{a'}{b'}}r_{aa'}} - \frac{4}{r_{aa'} r_{ab'}} - \frac{8}{s_{a{a'}{b'}}} \big ( \frac{1}{r_{aa'}} + \frac{4}{r_{{a'}{b'}}} \big ) \big ]\delta _{ij} \bigg \}, \end{aligned}$$

E.8 $$\begin{aligned}&Q'_{ij}({\mathbf {x}}_a,{\mathbf {p}}_a,t) \equiv -\frac{1}{16}\frac{1}{16\pi } \sum \limits _{a}\sum \limits _{a'}\frac{m_{a'}}{m_{a} r_{aa'}} \big \{ 2p_{ai}p_{aj}+12(\mathbf {n}_{aa'}\cdot {\mathbf {p}}_a)n_{aa'}^i p_{aj} \nonumber \\&\quad - 5{\mathbf {p}}_a^2 n_{aa'}^i n_{aa'}^j + 3(\mathbf {n}_{aa'}\cdot {\mathbf {p}}_a)^2 n_{aa'}^i n_{aa'}^j \big \}, \end{aligned}$$

E.9 $$\begin{aligned}&Q''_{ij}({\mathbf {x}}_a,t) \equiv \frac{1}{32} \frac{1}{(16\pi )^2} \sum \limits _{a}\sum \limits _{b\ne a}\sum \limits _{a'} m_a m_b m_{a'} \bigg \{ \frac{32}{s_{aba'}} \big ( \frac{1}{r_{ab}} + \frac{1}{s_{aba'}} \big ) n_{ab}^i n_{ab}^j \nonumber \\&\quad + \big [ 3 \frac{r_{aa'}}{r_{ab}^3} - \frac{5}{r_{ab}r_{aa'}} +\frac{r_{ba'}^2}{r_{ab}^3r_{aa'}} + \frac{8}{s_{aba'}} \big ( \frac{1}{r_{aa'}} + \frac{1}{s_{aba'}} \big ) \big ] n_{aa'}^in_{aa'}^j \nonumber \\&\quad - 2 \big ( \frac{r_{aa'}+r_{ba'}}{r_{ab}^3} + \frac{12}{s_{aba'}^2} \big ) n_{aa'}^i n_{ba'}^j - 32 \big ( \frac{1}{r_{ab}^2} - \frac{2}{s_{aba'}^2} \big ) n_{ab}^i n_{aa'}^j \bigg \}, \end{aligned}$$

E.10 $$\begin{aligned}&R'({\mathbf {x}}_a,{\mathbf {p}}_a,t) \equiv \frac{2}{105} \frac{1}{16\pi } \sum \limits _{a}\sum \limits _{a'} \frac{r_{aa'}^2}{m_{a}m_{a'}} \big \{ - 5 {\mathbf {p}}_a^2 \mathbf {p}_{a'}^2 + 11 ({\mathbf {p}}_a\cdot \mathbf {p}_{a'})^2 \nonumber \\&\quad + 4(\mathbf {n}_{aa'}\cdot \mathbf {p}_{a'})^2 {\mathbf {p}}_a^2 + 4 (\mathbf {n}_{aa'}\cdot {\mathbf {p}}_a)^2 \mathbf {p}_{a'}^2 - 12 (\mathbf {n}_{aa'}\cdot \mathbf {p}_{a'}) (\mathbf {n}_{aa'}\cdot {\mathbf {p}}_a) ({\mathbf {p}}_a\cdot \mathbf {p}_{a'}) \big \} \nonumber \\&\quad - \frac{1}{105} \frac{1}{(16\pi )^2} \sum \limits _{a}\sum \limits _{a'}\sum \limits _{b'\ne a'} \frac{m_{a'}m_{b'}}{m_a} \bigg \{ \big ( 2 \frac{r_{aa'}^4}{r_{a'b'}^3} - 2 \frac{r_{aa'}^2 r_{ab'}^2}{r_{a'b'}^3} - 5 \frac{r_{aa'}^2}{r_{a'b'}} \big ) {\mathbf {p}}_a^2 \nonumber \\&\quad + 4 \frac{r_{aa'}^2}{r_{a'b'}} (\mathbf {n}_{aa'}\cdot {\mathbf {p}}_a)^2 + 17 \big ( \frac{r_{aa'}^2}{r_{a'b'}} + r_{a'b'} \big ) (\mathbf {n}_{a'b'}\cdot {\mathbf {p}}_a)^2 \nonumber \\&\quad + 2 \big ( 6 \frac{r_{aa'}^3}{r_{a'b'}^2} + 17 r_{aa'} \big ) (\mathbf {n}_{aa'}\cdot {\mathbf {p}}_a) (\mathbf {n}_{a'b'}\cdot {\mathbf {p}}_a) \bigg \}, \end{aligned}$$

E.11 $$\begin{aligned}&R''({\mathbf {x}}_a,t) \equiv \frac{1}{105} \frac{1}{(16\pi )^2} \sum \limits _{a}\sum \limits _{b\ne a}\sum \limits _{a'} \frac{m_a m_b}{m_{a'}} \bigg \{ \big ( 5 \frac{r_{aa'}^2}{r_{ab}} + 2 \frac{r_{aa'}^2r_{ba'}^2}{r_{ab}^3} - 2 \frac{r_{aa'}^4}{r_{ab}^3} \big ) \mathbf {p}_{a'}^2 \nonumber \\&\qquad \qquad \qquad - 17 \big ( \frac{r_{aa'}^2}{r_{ab}} + r_{ab} \big ) (\mathbf {n}_{ab}\cdot \mathbf {p}_{a'})^2 - 4 \frac{r_{aa'}^2}{r_{ab}} (\mathbf {n}_{aa'}\cdot \mathbf {p}_{a'})^2 \nonumber \\&\qquad \qquad \quad + 2 \big ( \frac{6r_{aa'}^3}{r_{ab}^2} + 17 r_{aa'} \big ) (\mathbf {n}_{ab}\cdot \mathbf {p}_{a'}) (\mathbf {n}_{aa'}\cdot \mathbf {p}_{a'}) \bigg \} \nonumber \\&\quad \qquad \qquad + \frac{1}{210} \frac{1}{(16\pi )^3} \sum \limits _{a}\sum \limits _{b\ne a}\sum \limits _{a'}\sum \limits _{b'\ne a'} m_a m_b m_{a'} m_{b'} \bigg \{ 2 \frac{r_{aa'}^2}{r_{ab}r_{a'b'}^3}\left( r_{aa'}^2 - r_{ab'}^2\right) \nonumber \\&\quad + 2 \frac{r_{aa'}^2}{r_{ab}^3r_{a'b'}}\big (r_{aa'}^2 - r_{ba'}^2 \big ) + 4 \frac{r_{ab}r_{aa'}^2}{r_{a'b'}^3} - 5 \frac{r_{aa'}^2}{r_{ab}r_{a'b'}} - 2 \big ( \frac{r_{ab}^3}{r_{a'b'}^3} + \frac{r_{ab}}{r_{a'b'}} \big ) \nonumber \\&\quad - 4 \frac{r_{ab}r_{aa'}r_{bb'}}{r_{a'b'}^3} (\mathbf {n}_{aa'}\cdot \mathbf {n}_{bb'}) + 17 \big ( \frac{r_{ab}}{r_{a'b'}} + \frac{r_{a'b'}}{r_{ab}} + \frac{r_{aa'}^2}{r_{ab}r_{a'b'}} \big ) (\mathbf {n}_{ab}\cdot \mathbf {n}_{a'b'})^2 \nonumber \\&\quad + 6 \frac{r_{aa'}^4}{r_{ab}^2 r_{a'b'}^2} (\mathbf {n}_{ab}\cdot \mathbf {n}_{a'b'}) +34r_{aa'}^2\big (\frac{1}{r_{ab}^2}+\frac{1}{r_{a'b'}^2}\big ) (\mathbf {n}_{ab}\cdot \mathbf {n}_{a'b'}) \bigg \}. \end{aligned}$$

The leading-order Hamiltonian for systems made of any number of spinning bodies was derived by Wang et al. (2011). It reads^16^

E.12 $$\begin{aligned}&H^{\text {spin}}_{\text {3.5PN}}({\mathbf {x}}_a,{\mathbf {p}}_a,{\mathsf {S}}_a,t) = 5\pi \Big ( \chi _{(4)ij}({\mathbf {x}}_a,{\mathbf {p}}_a) \big (\dot{\varPi }_{1 ij}^{\text {spin}}(t) + \dot{\varPi }_{2 ij}^{\text {spin}}(t) + \ddot{\varPi }_{3 ij}^{\text {spin}}(t)\big ) \nonumber \\&\quad + \dot{\chi }_{(4)ij}(t) \big ( \varPi _{1ij}^{\text {spin}}({\mathbf {x}}_a,{\mathbf {p}}_a,{\mathsf {S}}_a) + \widetilde{\varPi }_{2ij}^{\text {spin}}({\mathbf {x}}_a,t) \big ) \nonumber \\&\quad - \ddot{\chi }_{(4)ij}(t) \varPi _{3 ij}^{\text {spin}}({\mathbf {x}}_a,{\mathsf {S}}_a) \Big ) + \dot{\chi }_{(4)ij}(t) Q'^{\,\text {spin}}_{ij}({\mathbf {x}}_a,{\mathbf {p}}_a,{\mathsf {S}}_a,t) \nonumber \\&\quad + \frac{\partial ^3}{\partial t^3} \Big ( R'^{\,\text {spin}}({\mathbf {x}}_a,{\mathbf {p}}_a,{\mathsf {S}}_a,t) + R''^{\,\text {spin}}({\mathbf {x}}_a,t) \Big ) \nonumber \\&\quad - \frac{\mathrm {d}}{\mathrm {d}t}\Big (\dot{\chi }_{(4)ij}(t) O_{ij}^{\text {spin}}({\mathbf {p}}_a,{\mathsf {S}}_a)\Big ), \end{aligned}$$

where ${\mathsf {S}}_a$ is the spin tensor associated with *a*th body, with components $S_{a(i)(j)}$. The function $\chi _{(4)ij}$ is defined in Eq. (E.3) above and the functions ${\varPi }_{1ij}^{\text {spin}}$, ${\varPi }_{2ij}^{\text {spin}}$, ${\varPi }_{3ij}^{\text {spin}}$, $\widetilde{\varPi }_{2ij}^{\text {spin}}$, $Q'^{\,\text {spin}}_{ij}$, $R'^{\,\text {spin}}$, $R''^{\,\text {spin}}$, and $O_{ij}^{\text {spin}}$ are given by

E.13 $$\begin{aligned}&\varPi ^{\mathrm{spin}}_{1ij}({\mathbf {x}}_a,{\mathbf {p}}_a,{\mathsf {S}}_a) \equiv \frac{4}{5(16\pi )^2}\sum _a\sum _{b \ne a} \bigg \{ \frac{1}{r_{ab}^2} \Big [ 3({\mathbf {n}}_{ab}\cdot {\mathbf {p}}_b) n_{ab}^k (n_{ab}^j S_{a(i)(k)} + n_{ab}^iS_{a(j)(k)}) \nonumber \\&\quad - 3 p_{bk} ( n_{ab}^j S_{a(i)(k)} + n_{ab}^i S_{a(j)(k)} ) - 3 n_{ab}^k ( p_{bj} S_{a(i)(k)} + p_{bi} S_{a(j)(k)} ) \nonumber \\&\quad + 4 (3n_{ab}^i n_{ab}^j - \delta _{ij}) n_{ab} ^k p_{bl} S_{a(k)(l)} \Big ] + \frac{m_b}{m_a} \frac{1}{r_{ab}^2} \Big [p_{ak} (n_{ab}^j S_{a(i)(k)} + n_{ab}^i S_{a(j)(k)}) \nonumber \\&\quad + (4 \delta _{ij} - 6 n_{ab}^i n_{ab}^j ) n_{ab}^k p_{al} S_{a(k)(l)} + 4n_{ab}^k (p_{aj} S_{a(i)(k)} + p_a {}_{i} S_{a(j)(k)} ) \Big ] \nonumber \\&\quad - \frac{S_{a(k)(l)}}{r_{ab}^3} \Big [ (3n_{ab}^i n_{ab}^j - \delta _{ij}) S_{b(k)(l)} + 3n_{ab}^k (n_{ab}^j S_{b(i)(l)} + n_{ab}^i S_{b(j)(l)})\nonumber \\&\quad + 3 (\delta _{ij} - 5 n_{ab}^i n_{ab}^j ) { n_{ab}^k n_{ab}^{n}S_{b(n)(l)} } \Big ] \bigg \}, \end{aligned}$$

E.14 $$\begin{aligned}&\varPi ^{\mathrm{spin}}_{2ij}({\mathbf {x}}_a,{\mathbf {p}}_a,{\mathsf {S}}_a) \equiv -\frac{4}{5(16\pi )^2} \sum _a\sum _{b\ne a} \frac{m_b}{m_a} \frac{1}{r_{ab}^2} \Big \{ -2p_{ak} (n_{ab}^i S_{a(j)(k)} + n_{ab}^j S_{a(i)(k)}) \nonumber \\&\quad + n_{ab}^k (p_{ai} S_{a(j)(k)} + p_{aj} S_{a(i)(k)}) + 3 ({\mathbf {n}}_{ab}\cdot {\mathbf {p}}_a) n_{ab}^k (n_{ab}^i S_{a(j)(k)} + n_{ab}^j S_{a(i)(k)}) \nonumber \\&\quad + (\delta _{ij} + 3 n_{ab}^i n_{ab}^j) n_{ab}^k p_{al} S_{a(k)(l)} \Big \}, \end{aligned}$$

E.15 $$\begin{aligned}&\varPi ^{\mathrm{spin}}_{3ij}({\mathbf {x}}_a,{\mathbf {p}}_a,{\mathsf {S}}_a) \equiv \frac{4}{5(16\pi )^2} \sum _a\sum _{b\ne a} \frac{m_b}{r_{ab}} n_{ab}^k (n_{ab}^j S_{a(i)(k)} + n_{ab}^i S_{a(j)(k)}), \end{aligned}$$

E.16 $$\begin{aligned}&{{\widetilde{\varPi }}}_{2ij}^{\mathrm{spin}}({\mathbf {x}}_a,t) \equiv -\frac{4}{5(16\pi )^2} \sum _a\sum _{a'} \frac{m_a}{m_{a'}} \frac{1}{r_{{a}a'}^2} \Big \{ 2p_{a'k} (n_{{a}a'}^i S_{a'(j)(k)} + n_{{a}a'}^j S_{a'(i)(k)}) \nonumber \\&\quad - n_{{a}a'}^k (p_{a'i} S_{a'(j)(k)} + p_{a'j} S_{a'(i)(k)}) - (\delta _{ij} + 3 n_{{a}a'}^i n_{{a}a'}^j) n_{{a}a'}^k p_{a'l} S_{a'(k)(l)} \nonumber \\&\quad - 3 ({\mathbf {n}}_{aa'}\cdot {\mathbf {p}}_{a'}) n_{{a}a'}^k (n_{{a}a'}^i S_{a'(j)(k)} +n_{{a}a'}^j S_{a'(i)(k)}) \Big \}, \end{aligned}$$

E.17 $$\begin{aligned}&Q'^{\,\text {spin}}_{ij}({\mathbf {x}}_a,{\mathbf {p}}_a,{\mathsf {S}}_a,t) \equiv \frac{1}{4(16\pi )} \sum _a\sum _{a'} \frac{m_{a'}}{m_a} \frac{1}{r_{{a}a'}^2} \Big \{ 2 p_{ak} (n_{{a}a'}^i S_{a(j)(k)} + n_{{a}a'}^j S_{a(i)(k)}) \nonumber \\&\quad - n_{{a}a'}^k (p_{ai} S_{a(j)(k)} + p_{aj} S_{a(i)(k)}) - (\delta _{ij} + 3n_{{a}a'}^i n_{{a}a'}^j) n_{{a}a'}^k p_{al} S_{a(k)(l)} \nonumber \\&\quad - 3 ({\mathbf {n}}_{aa'}\cdot {\mathbf {p}}_a) n_{{a}a'}^k (n_{{a}a'}^i S_{a(j)(k)} + n_{{a}a'}^j S_{a(i)(k)}) \Big \}, \end{aligned}$$

E.18 $$\begin{aligned}&R'^{\,\text {spin}}({\mathbf {x}}_a,{\mathbf {p}}_a,{\mathsf {S}}_a,t) \equiv \frac{1}{15(16\pi )} \sum _a\sum _{a'} S_{a(i)(j)} \bigg \{ \frac{4 r_{a'a}}{m_{a'} m_a} \big ( {\mathbf {p}}_{a'}^2 n_{a'a}^i p_{aj} \nonumber \\&\quad - ({\mathbf {n}}_{a'a}\cdot {\mathbf {p}}_{a'}) p_{a'i} p_{aj} - 2 ({\mathbf {p}}_{a'}\cdot {\mathbf {p}}_a) n_{a'a}^i p_{a'j} \big ) \nonumber \\&\quad + \frac{1}{7(16\pi )} \sum _{b'\ne a'}\frac{m_{a'}m_{b'}}{m_a} \bigg ( 17 n_{a'b'}^i p_{aj} - \frac{2r_{a'a}}{r_{a'b'}} \big ( 17 ({\mathbf {n}}_{a'b'}\cdot {\mathbf {p}}_a) n_{a'b'}^i n_{a'a}^j \nonumber \\&\quad + 7 n_{a'a}^i p_{aj} \big ) + \frac{6 r_{a'a}^2}{r_{a'b'}^2} \big ( n_{a'b'}^i p_{aj} + 2 ({\mathbf {n}}_{a'a}\cdot {\mathbf {p}}_a) n_{a'b'}^i n_{a'a}^j \big ) \nonumber \\&\quad + \frac{8 r_{a'a}}{r_{a'b'}^3} \big ( r_{a'a}^2 n_{a'a}^i p_{aj} - r_{b'a}^2 n_{a'a}^i p_{aj} \big ) \bigg ) \bigg \} \nonumber \\&\quad + \frac{4}{15(16\pi )} \sum _a\sum _{a'} \frac{r_{aa'}}{m_{a'} m_a} S_{a'(i)(j)} \Big ( {\mathbf {p}}_a^2 n_{a a'}^i p_{a'j} - 2({\mathbf {p}}_{a'}\cdot {\mathbf {p}}_a) n_{a a'}^i p_{aj} \nonumber \\&\quad + ({\mathbf {n}}_{aa'}\cdot {\mathbf {p}}_a) p_{a'i} p_{aj} \Big ) + \frac{2}{15(16\pi )} \sum _a\sum _{a'\ne a} \frac{1}{m_{a'} m_a} S_{a(i)(j)} \Big ( 3 p_{a'k} p_{ai} S_{a'(k)(j)} \nonumber \\&\quad - 2 ({\mathbf {p}}_{a'}\cdot {\mathbf {p}}_a) S_{a'(i)(j)} - 2 p_{a'i} p_{ak} S_{a'(k)(j)} \Big ), \end{aligned}$$

E.19 $$\begin{aligned}&R''^{\,\text {spin}}({\mathbf {x}}_a,t) \equiv \frac{2}{15(16\pi )^2} \sum _a\sum _{b\ne a}\sum _{a'} \frac{m_a m_b}{m_{a'}} \frac{r_{a'a}}{r_{ab}} S_{a'(i)(j)} \Big ( n_{a'a}^i p_{a'j} \nonumber \\&\quad - 2 ({\mathbf {n}}_{ab}\cdot {\mathbf {p}}_{a'}) n_{a'a}^i n_{ab}^j - ({\mathbf {n}}_{a'a}\cdot {\mathbf {n}}_{ab}) n_{ab}^i p_{a'j} \Big ), \end{aligned}$$

E.20 $$\begin{aligned}&O_{ij}^{\text {spin}}({\mathbf {p}}_a,{\mathsf {S}}_a) \equiv \sum _a \frac{1}{8m_a^2} p_{ak} \big (p_{ai} S_{a(k)(j)} + p_{aj} S_{a(k)(i)}\big ). \end{aligned}$$

## F Closed-form 1PM Hamiltonian for point-mass systems

The first post-Minkowskian (1PM) closed-form Hamiltonian for point-mass systems has been derived by Ledvinka et al. (2008). The starting point is the ADM reduced Hamiltonian describing *N* gravitationally interacting point masses with positions ${\mathbf {x}}_a$ and linear momenta $\mathbf{p}_a$ ($a=1,\ldots ,N)$. The 1PM Hamiltonian is, by definition, accurate through terms linear in *G* and it reads (setting $c=1$)

F.1 $$\begin{aligned} H_{\mathrm{lin}}&= \sum _a {\overline{m}}_a - \frac{1}{2}G\sum _{a,b\ne a} \frac{{\overline{m}}_a {\overline{m}}_b}{ r_{ab} } \left( 1+ \frac{p_a^2}{{\overline{m}}_a^2}+\frac{p_b^2}{{\overline{m}}_b^2}\right) \nonumber \\&\quad + \frac{1}{4}G\sum _{a,b\ne a} \frac{1}{r_{ab}}\left( 7\, \mathbf{p}_a \cdot \mathbf{p}_b + (\mathbf{p}_a \cdot \mathbf{n}_{ab})(\mathbf{p}_b \cdot \mathbf{n}_{ab}) \right) \nonumber \\&\quad - \frac{1}{2}\sum _a \frac{p_{ai}p_{aj}}{{\overline{m}}_a}\,h_{ij}^{\mathrm{TT}}(\mathbf{x}=\mathbf{x}_a) + \frac{1}{16\pi G} \int \mathrm {d}^3x \left( \frac{1}{4} h_{ij,k}^{\mathrm{TT}}\, h_{ij,k}^{\mathrm{TT}} + \pi ^{ij}_{\mathrm{TT}} \pi ^{ij}_{\mathrm{TT}}\right) , \end{aligned}$$

where ${\overline{m}}_a\equiv \left( m_a^2+\mathbf{p}^2_a \right) ^\frac{1}{2}$ and $\mathbf{n}_{ab} r_{ab}\equiv \mathbf{x}_a-\mathbf{x}_b$ (with $|\mathbf{n}_{ab}|=1$). The independent degrees of freedom of the gravitational field, $h_{ij}^{\mathrm{TT}}$ and $\pi ^{ij}_{\mathrm{TT}}$, are treated to linear order in *G*. Denoting $\mathbf{x}-\mathbf{x}_a\equiv \mathbf{n}_a |\mathbf{x}-\mathbf{x}_a|$ and $\cos \theta _a\equiv ({\mathbf{n}_a \cdot {\dot{\mathbf{x}}}_a) /|{\dot{\mathbf{x}}}_a|}$, the solution for $h_{ij}^{\mathrm{TT}}(\mathbf{x})$ was found to be

F.2 $$\begin{aligned} h_{ij}^{\mathrm {TT}}(\mathbf{x}) = \delta _{ij}^{\mathrm {TT}\,kl} \sum _b \frac{4G}{{\overline{m}}_b} \frac{1}{|\mathbf{x}-\mathbf{x}_b|} \frac{p_{bk}p_{bl}}{\sqrt{1-{{\dot{\mathbf{x}}}_b}^2\sin ^2 \theta _b}}. \end{aligned}$$

An autonomous point-mass Hamiltonian needs the field part in the related Routhian,

F.3 $$\begin{aligned} R_f = \frac{1}{16\pi G} \int \mathrm {d}^3x\, \frac{1}{4}\left( h_{ij,k}^{\mathrm{TT}}\, h_{ij,k}^{\mathrm{TT}} - \dot{h}_{ij}^{\mathrm{TT}}\dot{h}_{ij}^{\mathrm{TT}} \right) , \end{aligned}$$

to be transformed into an explicit function of particle variables. Using the Gauss law in the first term and integrating by parts the term containing the time derivatives one arrives at

F.4 $$\begin{aligned} R_f= & {} -\frac{1}{16\pi G}\int \mathrm {d}^3x\, \frac{1}{4} h_{ij}^{\mathrm{TT}}\left( \varDelta h_{ij}^{\mathrm{TT}} - \partial _t^2 h_{ij}^{\mathrm{TT}} \right) + \frac{1}{64\pi G} \oint \mathrm {d}S_k (h_{ij}^{\mathrm{TT}} h_{ij,k}^{\mathrm{TT}}) \nonumber \\&\quad - \frac{1}{64\pi G} \frac{\mathrm {d}}{\mathrm {d}t} \int \mathrm {d}^3 x\,(h_{ij}^{\mathrm{TT}} \dot{h}_{ij}^{\mathrm{TT}}). \end{aligned}$$

The field equations imply that the first integral directly combines with the “interaction” term containing $\sum \, {\overline{m}}_a^{-1}\, p_{ai}\, p_{aj} \,h^{\mathrm{TT}}_{ij}(\mathbf{x}_a)$, so only its coefficient gets changed. The remaining terms in $R_f$, the surface integral and the total time derivative, do not modify the dynamics of the system since in our approximation of unaccelerated field-generating particles, the surface integral vanishes at large $|\mathbf{x}|$. The reduced Routhian thus takes the form, now referred to as *H* because it is a Hamiltonian for the particles,

F.5 $$\begin{aligned} H_{\mathrm{lin}}(\mathbf{x}_c,\mathbf{p}_c,{\dot{\mathbf{x}}}_c)&= \sum _a {\overline{m}}_a - \frac{1}{2}G\sum _{a,b\ne a} \frac{{\overline{m}}_a {\overline{m}}_b }{ r_{ab}} \left( 1+ 2\frac{p_a^2}{{\overline{m}}_a^2}\right) \nonumber \\&\quad + \frac{1}{4}G\sum _{a,b\ne a} \frac{1}{r_{ab}}\left( 7\, (\mathbf{p}_a \cdot \mathbf{p}_b) + (\mathbf{p}_a \cdot \mathbf{n}_{ab})(\mathbf{p}_b \cdot \mathbf{n}_{ab}) \right) \nonumber \\&\quad - \frac{1}{4}\sum _a \frac{p_{ai}p_{aj} }{{\overline{m}}_a}\,h_{ij}^{\mathrm{TT}}(\mathbf{x}=\mathbf{x}_a;\mathbf{x}_b,\mathbf{p}_b,{\dot{\mathbf{x}}}_b). \end{aligned}$$

Though dropping a total time derivative, which implies a canonical transformation, the new canonical coordinates keep their names. A further change of coordinates has to take place to eliminate the velocities ${\dot{\mathbf{x}}}_a$ in the Hamiltonian. This can be achieved by simply putting ${\dot{\mathbf{x}}}_a = \mathbf{p}_a / {\overline{m}}_a$ (again without changing names of the variables). Using the shortcut $y_{ba}\equiv {\overline{m}}_b^{-1} [ m_b^2+ \left( \mathbf{n}_{ba} \cdot \mathbf{p}_b\right) ^2]^\frac{1}{2}$, the Hamiltonian comes out in the final form (Ledvinka et al. 2008)

F.6 $$\begin{aligned} H_{\mathrm{lin}}&= \sum _a {\overline{m}}_a - \frac{1}{2}G\sum _{a,b\ne a} \frac{{\overline{m}}_a {\overline{m}}_b }{ r_{ab}} \left( 1+ \frac{p_a^2}{ {\overline{m}}_a^2}+\frac{p_b^2}{{\overline{m}}_b^2}\right) \nonumber \\&\quad + \frac{1}{4}G\sum _{a,b\ne a} \frac{1}{r_{ab}}\left( 7\, (\mathbf{p}_a \cdot \mathbf{p}_b) + (\mathbf{p}_a \cdot \mathbf{n}_{ab})(\mathbf{p}_b \cdot \mathbf{n}_{ab}) \right) \nonumber \\&\quad - \frac{1}{4} G \sum _{a,b\ne a} \frac{1}{r_{ab}} \frac{({\overline{m}}_a {\overline{m}}_b)^{-1}}{ (y_{ba}+1)^2 y_{ba}} \Bigg \{ 2\Big (2 (\mathbf{p}_a \cdot \mathbf{p}_b)^2 (\mathbf{p}_b \cdot \mathbf{n}_{ba})^2 \nonumber \\&\quad - 2 (\mathbf{p}_a \cdot \mathbf{n}_{ba}) (\mathbf{p}_b \cdot \mathbf{n}_{ba}) (\mathbf{p}_a \cdot \mathbf{p}_b) \mathbf{p}_b^2 +(\mathbf{p}_a \cdot \mathbf{n}_{ba})^2 \mathbf{p}_b^4 -(\mathbf{p}_a \cdot \mathbf{p}_b)^2 \mathbf{p}_b^2 \Big ) \frac{1}{{\overline{m}}_b^2} \nonumber \\&\quad + 2 \Big [ (\mathbf{p}_a \cdot \mathbf{p}_b)^2 - \mathbf{p}_a^2 (\mathbf{p}_b \cdot \mathbf{n}_{ba})^2 + (\mathbf{p}_a \cdot \mathbf{n}_{ba})^2 (\mathbf{p}_b \cdot \mathbf{n}_{ba})^2 \nonumber \\&\quad + 2 (\mathbf{p}_a \cdot \mathbf{n}_{ba}) (\mathbf{p}_b \cdot \mathbf{n}_{ba}) (\mathbf{p}_a \cdot \mathbf{p}_b) - (\mathbf{p}_a \cdot \mathbf{n}_{ba})^2 \mathbf{p}_b^2\Big ] \nonumber \\&\quad + \Big [ \mathbf{p}_a^2 \mathbf{p}_b^2 - 3 \mathbf{p}_a^2 (\mathbf{p}_b \cdot \mathbf{n}_{ba})^2 +(\mathbf{p}_a \cdot \mathbf{n}_{ba})^2 (\mathbf{p}_b \cdot \mathbf{n}_{ba})^2 \nonumber \\&\quad + 8 (\mathbf{p}_a \cdot \mathbf{n}_{ba}) (\mathbf{p}_b \cdot \mathbf{n}_{ba}) (\mathbf{p}_a \cdot \mathbf{p}_b) - 3 (\mathbf{p}_a \cdot \mathbf{n}_{ba})^2 \mathbf{p}_b^2 \Big ]y_{ba} \Bigg \}. \end{aligned}$$

This is the Hamiltonian for a many-point-mass system through 1PM approximation, i.e., including all terms linear in *G*. It is given in closed form and entirely in terms of the canonical variables of the particles.

The usefulness of that Hamiltonian has been proved in several applications (see, e.g., Foffa and Sturani 2011, 2013a; Jaranowski and Schäfer 2012; Damour 2016; Feng et al. 2018). Especially in Jaranowski and Schäfer (2012) it was checked that the terms linear in *G* in the 4PN-accurate ADM Hamiltonian derived there, are, up to adding a total time derivative, compatible with the 4PN-accurate Hamiltonian which can be obtained from the exact 1PM Hamiltonian (F.6). Let us also note that Damour (2016) has shown that, after a suitable canonical transformation, the rather complicated Hamiltonian (F.6) is equivalent (modulo the EOB energy map) to the much simpler Hamiltonian of a test particle moving in a (linearized) Schwarzschild metric. The binary centre-of-mass 2PM Hamiltonian has been derived most recently by Damour (2018) in an EOB-type form and also the gravitational spin–orbit coupling in binary systems has been achieved at 2PM order by Bini and Damour (2018) (for other 2PM results see, e.g., Bel et al. 1981; Westpfahl 1985).

## G Skeleton Hamiltonian for binary black holes

The skeleton approach to GR developed by Faye et al. (2004), is a truncation of GR such that an analytic PN expansion exists to arbitrary orders which, at the same time, is explicitly calculable. The approach imposes the conformal flat condition for the spatial three-metric for all times (not only initially as for the Brill–Lindquist solution), together with a specific truncation of the field-momentum energy density. It exactly recovers the general relativity dynamical equations in the limits of test-body and 1PN dynamics. The usefulness of the skeleton approach in the construction of initial data needed for numerical solving binary black hole dynamics was studied by Bode et al. (2009).

The conformally flat metric

G.1 $$\begin{aligned} \gamma _{ij} = \left( 1+\frac{1}{8}\phi \right) ^4 \delta _{ij} \end{aligned}$$

straightforwardly results in maximal slicing, using the ADM coordinate conditions,

G.2 $$\begin{aligned} \pi ^{ij}\gamma _{ij} = 2 \sqrt{\gamma } \gamma ^{ij} K_{ij} = 0. \end{aligned}$$

Our coordinates fit to the both ADM and Dirac coordinate conditions. The momentum constraint equations now become

G.3 $$\begin{aligned} \pi ^j_{i,\,j} = -\frac{8 \pi G}{c^3} \sum _a p_{ai}\delta _a. \end{aligned}$$

The solution of these equations is constructed under the condition that $\pi ^j_{i}$ is purely longitudinal, i.e.,

G.4 $$\begin{aligned} \pi ^j_{i} = \partial _i V_j + \partial _j V_i - \frac{2}{3} \delta _{ij} \partial _l V_l. \end{aligned}$$

This condition is part of the definition of the skeleton model.

Furthermore, in the Hamiltonian constraint equation, which in our case reads

G.5 $$\begin{aligned} \varDelta \phi = - \frac{ \pi ^j_i \pi ^i_j }{(1+\frac{1}{8}\phi )^{7} } -\frac{16\pi G}{c^2}\sum _a \frac{ m_a\delta _a}{ (1+\frac{1}{8}\phi )}\, \biggl ( 1+ \frac{p_a^2}{(1+\frac{1}{8}\phi )^4m_a^2c^2} \biggr )^{1/2}, \end{aligned}$$

a truncation of the numerator of the first term is made in the following form

G.6 $$\begin{aligned} \pi ^j_i \pi ^i_j \equiv -2 V_j\partial _i\pi ^i_j + \partial _i(2V_j\pi ^i_j) \, \rightarrow -2 V_j\partial _i\pi ^i_j=\frac{16\pi G}{c^3}\sum _a p_{aj}V_j\delta _a, \end{aligned}$$

i.e., dropping from $\pi ^j_i \pi ^i_j$ the term $\partial _i(2V_j\pi ^i_j)$. This is the second crucial truncation condition additional to the conformal flat one. Without this truncation neither an explicit analytic solution can be constructed nor a PN expansion is feasible. From Jaranowski and Schäfer (1998, 2000c), it is known that at the 3PN level the $h^{\mathrm{TT}}_{ij}$-field is needed to make the sum of the corresponding terms from $\pi ^j_i \pi ^i_j$ analytic in 1 / *c*.

With the aid of the ansatz

G.7 $$\begin{aligned} \phi = \frac{4G}{c^2}\sum _a \frac{\alpha _a}{r_a} \end{aligned}$$

and by making use of dimensional regularization, the energy and momentum constraint equations result in an algebraic equation for $\alpha _a$ of the form (Faye et al. 2004),

G.8 $$\begin{aligned} \alpha _a = \frac{m_a}{\displaystyle 1+\frac{G\alpha _b}{2r_{ab}c^2}} \left[ 1 + \frac{p_a^2/(m_a^2 c^2)}{\displaystyle \left( 1+\frac{G\alpha _b}{2r_{ab}c^2} \right) ^{4}} \right] ^{1/2} + \frac{p_{ai} V_{ai}/c}{\displaystyle \left( 1+\frac{G\alpha _b}{2r_{ab}c^2}\right) ^{7}}, \quad b \ne a.\nonumber \\ \end{aligned}$$

With these inputs the skeleton Hamiltonian for binary black holes results in

G.9 $$\begin{aligned} H_{\mathrm{sk}} = -\frac{c^4}{16\pi G}\int \mathrm {d}^3x \,\varDelta \phi = \sum _a \alpha _ac^2. \end{aligned}$$

The Hamilton equations of motion read

G.10 $$\begin{aligned} \dot{\mathbf{x}}_a = \frac{\partial H_{\mathrm{sk}}}{\partial {\mathbf {p}}_a}, \quad {\dot{{\mathbf {p}}}}_a = -\frac{\partial H_{\mathrm{sk}}}{\partial {\mathbf {x}}_a}. \end{aligned}$$

We will present the more explicit form of the binary skeleton Hamiltonian in the centre-of-mass reference frame of the binary, which is defined by the equality ${\mathbf {p}}_1+{\mathbf {p}}_2=\mathbf {0}$. We define

G.11 $$\begin{aligned} {\mathbf {p}}\equiv {\mathbf {p}}_1 = -{\mathbf {p}}_2, \quad {\mathbf {r}}\equiv \mathbf {x}_1 - \mathbf {x}_2, \quad r \equiv |{\mathbf {r}}|. \end{aligned}$$

It is also convenient to introduce dimensionless quantities^17^ (here $M\equiv m_1+m_2$ and $\mu \equiv m_1m_2/M$)

G.12 $$\begin{aligned} {\hat{{\mathbf {r}}}} \equiv \frac{{\mathbf {r}}c^2}{GM}, \quad \hat{{\mathbf {p}}} \equiv \frac{{\mathbf {p}}}{\mu c}, \quad {{\hat{\mathbf {p}}}}^2 = {{\hat{p}}}_r^2 + {{\hat{j}}}^2/{{\hat{r}}}^2 \quad \text {with}\quad {{\hat{p}}}_r \equiv \frac{p_r}{\mu c} \quad \text {and}\quad {{\hat{j}}} \equiv \frac{Jc}{GM\mu }, \end{aligned}$$

where $p_r\equiv \mathbf {p} \cdot \mathbf {r}/r$ is the radial linear momentum and $\mathbf {J}\equiv \mathbf {r} \times \mathbf {p}$ is the orbital angular momentum in the centre-of-mass frame. The reduced binary skeleton Hamiltonian ${{{\hat{H}}}}_{\mathrm{sk}}\equiv H_{\mathrm{sk}}/(\mu c^2)$ [it defines equations of motion with respect to dimensionless time ${{\hat{t}}}\equiv t c^3/(GM)$] can be put into the following form (Gopakumar and Schäfer 2008):

G.13 $$\begin{aligned} {{{\hat{H}}}}_{\mathrm{sk}} = 2\,{\hat{r}}(\psi _1 + \psi _2 - 2), \end{aligned}$$

where the functions $\psi _1$ and $\psi _2$ are solutions of the following system of coupled equations

G.14 $$\begin{aligned} \psi _{1}&= 1+\frac{ \chi _{-} }{4\, {{\hat{r}}}\, \psi _2} \, \sqrt{1+ \frac{ 4\,{\nu }^{2} \left( {{{{\hat{p}}}_r}}^{2}+{{{\hat{j}}}}^2/{{{\hat{r}}}}^{2} \right) }{ \chi _{-}^2\, \psi _2^4}} - \frac{ \left( 8\,{{ {{\hat{p}}}_r}}^{2}+7{{{\hat{j}}}}^2/{{{\hat{r}}}}^2 \right) {\nu }^{2}}{8\, {{{\hat{r}}}}^{2}\psi _2^7 }, \end{aligned}$$

G.15 $$\begin{aligned} \psi _{2}&= 1+\frac{ \chi _{+} }{4\, {{\hat{r}}}\, \psi _1} \, \sqrt{1+ \frac{ 4\,{\nu }^{2} \left( {{{{\hat{p}}}_r}}^{2}+{{{\hat{j}}}}^2/{{{\hat{r}}}}^2 \right) }{ \chi _{+}^2\, \psi _1^4}} - \frac{ \left( 8\,{{ {{\hat{p}}}_r}}^{2}+7{{{\hat{j}}}}^2/{{{\hat{r}}}}^2 \right) {\nu }^{2}}{8\, {{{\hat{r}}}}^{2}\psi _1^7}, \end{aligned}$$

where $\chi _{-}\equiv 1-\sqrt{1-4\,\nu }$ and $\chi _+\equiv 1+\sqrt{1-4\,\nu }$, with $\nu \equiv \mu /M$.

Beyond the properties mentioned in the beginning, the conservative skeleton Hamiltonian reproduces the Brill–Lindquist initial-value solution. It is remarkable that the skeleton Hamiltonian allows a PN expansion in powers of $1/c^2$ to arbitrary orders. The skeleton Hamiltonian thus describes the evolution of a kind of black holes under both conformally flat condition and the condition of analyticity in $1/c^2$. Along circular orbits the two-black-hole skeleton solution is quasistationary and it satisfies the property of the equality of Komar and ADM masses (Komar 1959, 1963). Of course, gravitational radiation emission is not included. It can, however, be added to some reasonable extent, see Gopakumar and Schäfer (2008).

Restricting to circular orbits and defining $x\equiv (GM\omega /c^3)^{2/3}$, where $\omega$ is the orbital angular frequency, the skeleton Hamiltonian reads explicitly to 3PN order,

G.16 $$\begin{aligned} {{{\hat{H}}}}_{\mathrm{sk}}&= -\frac{x}{2} + \bigg (\frac{3}{8}+\frac{\nu }{24}\bigg ) x^2 + \bigg (\frac{27}{16}+\frac{29}{16}\nu -\frac{17}{48}\nu ^2\bigg ) x^3 \nonumber \\&\quad + \bigg (\frac{675}{128}+\frac{8585}{384}\nu -\frac{7985}{192}\nu ^2 +\frac{1115}{10368}\nu ^3\bigg ) x^4 + \mathcal {O}(x^{5}). \end{aligned}$$

In Faye et al. (2004), the coefficients of this expansion are given to the order $x^{11}$ inclusively. We recall that the 3PN-accurate result of general relativity reads [cf. Eq. (6.65)],

G.17 $$\begin{aligned} {{{\hat{H}}}}_{\le \mathrm 3PN}&= -\frac{x}{2} + \bigg ( \frac{3}{8} + \frac{\nu }{24} \bigg ) x^2 + \bigg ( \frac{27}{16} - \frac{19}{16}\nu + \frac{1}{48}\nu ^2 \bigg ) x^3 \nonumber \\&\quad + \Bigg ( \frac{675}{128} + \bigg (\frac{205}{192}\pi ^2-\frac{34445}{1152}\bigg )\nu + \frac{155}{192} \nu ^2 + \frac{35}{10368} \nu ^3 \Bigg ) x^4. \end{aligned}$$

In the Isenberg–Wilson–Mathews approach to general relativity only the conformal flat condition is employed. Through 2PN order, the Isenberg–Wilson–Mathews energy of a binary is given by

G.18 $$\begin{aligned} {{{\hat{H}}}}_{\mathrm{IWM}} = -\frac{x}{2} + \bigg ( \frac{3}{8} + \frac{\nu }{24} \bigg ) x^2 + \bigg ( \frac{27}{16} - \frac{39}{16}\nu - \frac{17}{48} \nu ^2 \bigg ) x^3. \end{aligned}$$

The difference between ${{{\hat{H}}}}_{\mathrm{IWM}}$ and ${{{\hat{H}}}}_{\mathrm{sk}}$ shows the effect of truncation in the field-momentum part of ${{{\hat{H}}}}_{\mathrm{sk}}$ through 2PN order and the difference between ${{{\hat{H}}}}_{\mathrm{IWM}}$ and ${{{\hat{H}}}}_{\le \mathrm 3PN}$ reveals the effect of conformal flat truncation. In the test-body limit, $\nu =0$, the three Hamiltonians coincide.

## Footnotes

1 In such a particle Hamiltonian, the field degrees of freedom are treated as independent from the particle variables, rendering the particle Hamiltonian an explicit function of time.

2 The incompatibility of the extended Hadamard regularization with distribution theory and dimensional regularization is serious and can not be expressed in terms of several constant ambiguity parameters. This can be clearly seen from the paper by Blanchet et al. (2004) on deriving the 3PN equations of motion in harmonic coordinates: see the paragraph containing Eq. (1.8) and Eq. (3.55) there.

3 Equations (3.29) and (3.30) are taken from Jaranowski and Schäfer (1998, 2000c) and they are enough to calculate 3PN-accurate two-point-mass Hamiltonian. In Jaranowski and Schäfer (2015) one can find higher-order PN expansion of constraint equations, performed in *d* dimensions, necessary to compute 4PN Hamiltonian.

4 This approach is described in Appendix A3 a of Jaranowski and Schäfer (2015), where Eqs. (A40)–(A42) are misprinted: $(r/s)^B\ddot{h}^\text {TT}_{(4)ij}$ should be replaced by $\big [(r/s)^B\ddot{h}^\text {TT}_{(4)ij}\big ]^\text {TT}$. The Eq. (3.6) in Damour et al. (2014) is the correct version of Eq. (A40) in Jaranowski and Schäfer (2015).

5 Here $\mathrm {e}=2.718\ldots$ should be distinguished from the eccentricity *e*.

6 The 4PN value of the ISCO frequency parameter given here, 0.236599, is slightly different from the value 0.236597 published in Jaranowski and Schäfer (2013). The reason is that in Jaranowski and Schäfer (2013) the only then known approximate value 153.8803 of the linear-in-$\nu$ coefficient in the 4PN-order term in Eq. (6.65) was used, whereas the numerical exact value of this coefficient reads $153.8837968\ldots$. From the same reason the 4PN ISCO frequency parameter determined by the *j*-method described below in this section, is equal 0.242967, whereas the value published in Jaranowski and Schäfer (2013) reads 0.247515.

7 For more details about SSCs, see Sect. 3.3 of our review.

8 Especially Eq. (12.5.35) there.

9 Slightly earlier a fully dynamical calculation of that dynamics was made by Porto and Rothstein (2008a). This result turned out to be incomplete due to incorrect treatement of a specific Feynman diagram.

10 The final spin precession equation of the paper [Porto and Rothstein 2008a deviates from the corresponding one in Steinhoff et al. (2008c)]. A detailed inspection has shown that the last term in Eq. (60) of Porto and Rothstein (2008a) has wrong sign (Steinhoff and Schäfer 2009b). Using the correct sign, after redefinition of the spin variable, agreement with the Hamiltonian of Steinhoff et al. (2008c) is achieved.

11 The $H_{S_1^4}$ and $H_{S_2^4}$ terms were incorrectly claimed to be zero by Hergt and Schäfer (2008a).
